# Nitrilium ion trapping as a strategy to access structurally diverse heterobiaryl-containing peptide macrocycles†

**DOI:** 10.1039/d3sc03058j

**Published:** 2023-08-09

**Authors:** Matthew Diamandas, Nicholas W. Heller, Andrei K. Yudin

**Affiliations:** a Department of Chemistry, University of Toronto Toronto ON M5S 3H6 Canada

## Abstract

Biaryl and heterobiaryl-containing cyclic peptides represent promising scaffolds for the development of bioactive molecules. The incorporation of heterobiaryl motifs continues to pose synthetic challenges, which is partially due to the difficulties in effecting late-stage metal-catalyzed cross-couplings. We report a new strategy to form heterobiaryls that is based on trapping nitrilium ions. The sequence is exemplified using oxadiazole- and oxazole-containing biaryl linkages. NMR analysis and molecular dynamics simulations reveal structural control elements common to each member of the heterobiaryl containing peptide family in this study. Strategic substitutions on the C-terminal aminobenzoic acid moiety paired with installation of oxadiazole or oxazole heterobiaryl backbone linkages allow for the modulation of peptide backbone conformation, which should assist efforts to optimize the biophysical properties of peptide macrocycles.

## Abstract 2

Biaryl and heterobiaryl-containing cyclic peptides represent promising scaffolds for the development of bioactive molecules.

## Graphical abstract

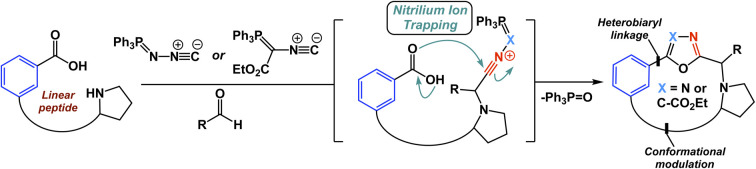

## Introduction

**Fig. 1:**
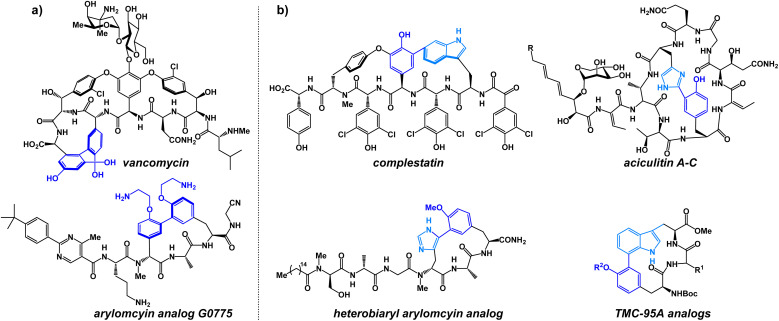
Medicinally relevant (a) biaryl and (b) heterobiaryl-containing peptide macrocycles.

In the past decade, a large number of target-specific, biaryl-containing peptide macrocycles have attracted the attention of researchers for the treatment of bacterial and fungal infections.^1^ As a result of their target selectivity and strong binding affinity, biaryl cyclic peptides represent promising scaffolds in the development of novel bioactive compounds.^2–4^ Especially noteworthy are the antibiotics vancomycin and the arylomycin analog G0775, each possessing potent antibacterial activity (Fig. 1a).^2^ Heterobiaryl peptides have also garnered attention in recent years. The heterobiaryl-containing peptide macrocycle complestatin (also known as chloropeptin II) possesses activity against HIV. Heterobiaryl linkages can be found in aciculitin A-C, TMC-95A as well as a synthetic heterobiaryl analog of arylomycin (Fig. 1b).^4,5^ More recently, a novel PCSK9 inhibitor produced by Merck contains a peptide bridge involving a heterobiaryl linkage between a triazole and an aryl ring. Synthetically, heterobiaryl and biaryl linkages in peptide macrocycles are typically constructed from building blocks and then incorporated into the peptide backbone *via* a conventional amidation reaction. Alternatively, metal-catalyzed ring-closing macrocyclization reactions are used towards heterobiaryl construction.^4,5^ Metal-catalyzed heterobiaryl formation has been utilized in many synthetic routes; however, it is typically limited to peptide sequences that do not contain metal-coordinating amino acids (cysteine, methionine, histidine) and relies on the use of pre-functionalized boron- or tin-containing starting materials, many of which are sensitive to both strongly basic and strongly acidic conditions. Yet another limitation is that biaryl-bearing compounds assembled using metal-catalyzed cross-coupling reactions can be contaminated with trace amounts of transition-metal impurities, making them insufficiently pure to meet stringent drug safety standards.^6^ Currently, no method exists that allows for the assembly of heterobiaryl scaffolds during peptide macrocyclization without the reliance on transition-metal catalysis.

**Fig. 2:**
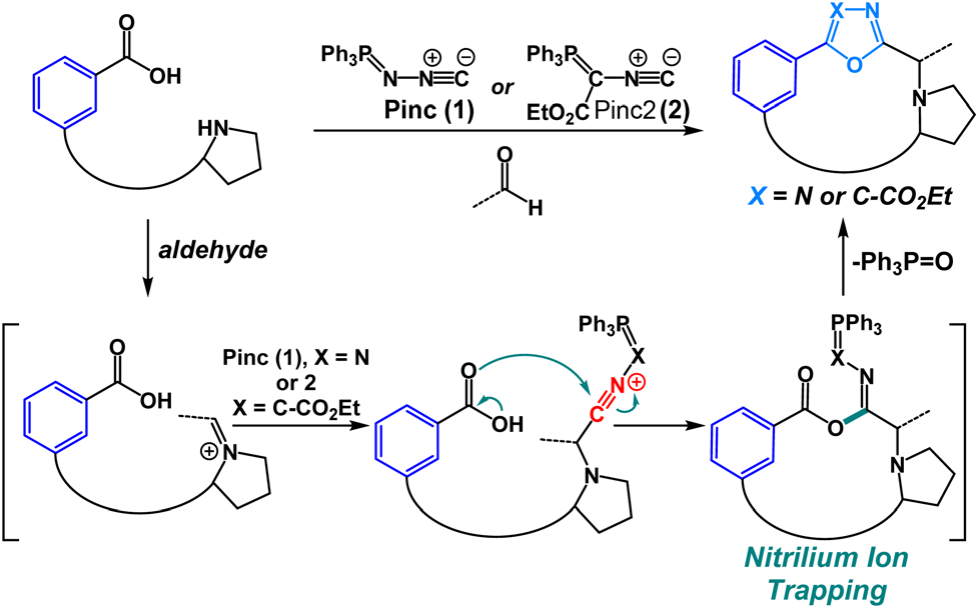
Heterobiaryl forming isocyanide reagents Pinc (1) and closely related isocyanide Pinc2 (2).

We have previously shown that oxadiazole-grafted peptide macrocycles prepared from (*N*-isocyanoimino)triphenylphosphorane (Pinc) possess predictable secondary structure.^7,8^ This is a result of the beta-turn stabilizing effect that can be observed in most oxadiazole-grafted macrocycles. Our lab has also previously utilized nitrilium ion intermediates in peptide macrocycles to effect an efficient macrocyclic ring-contraction strategy.^9^ Given our group's emerging interest in the synthesis and the structural features of heterobiaryl and bis(heteroaryl) containing peptides, we were keen to develop a new macrocyclization strategy that would allow for facile synthesis of heterobiaryl-containing peptide macrocycles without the reliance on transition-metal catalysis or the need to prepare building blocks that are suitably protected for Fmoc Solid-Phase Peptide Synthesis (SPPS).^9–13^ The isocyanide reagent Pinc has proven to be a powerful reagent for macrocyclization.^7,8,11^ Given these advances, we were motivated to utilize Pinc and closely related ethyl 2-isocyano-2-(triphenyl-λ^5^-phosphanylidene)acetate (Pinc2) to generate a series of oxadiazole and oxazole heterobiaryl linkages as part of a macrocyclization protocol enabled by the nucleophilic trapping of nitrilium intermediates (Fig. 2).^14^

## Results and discussion

**Fig. 3:**
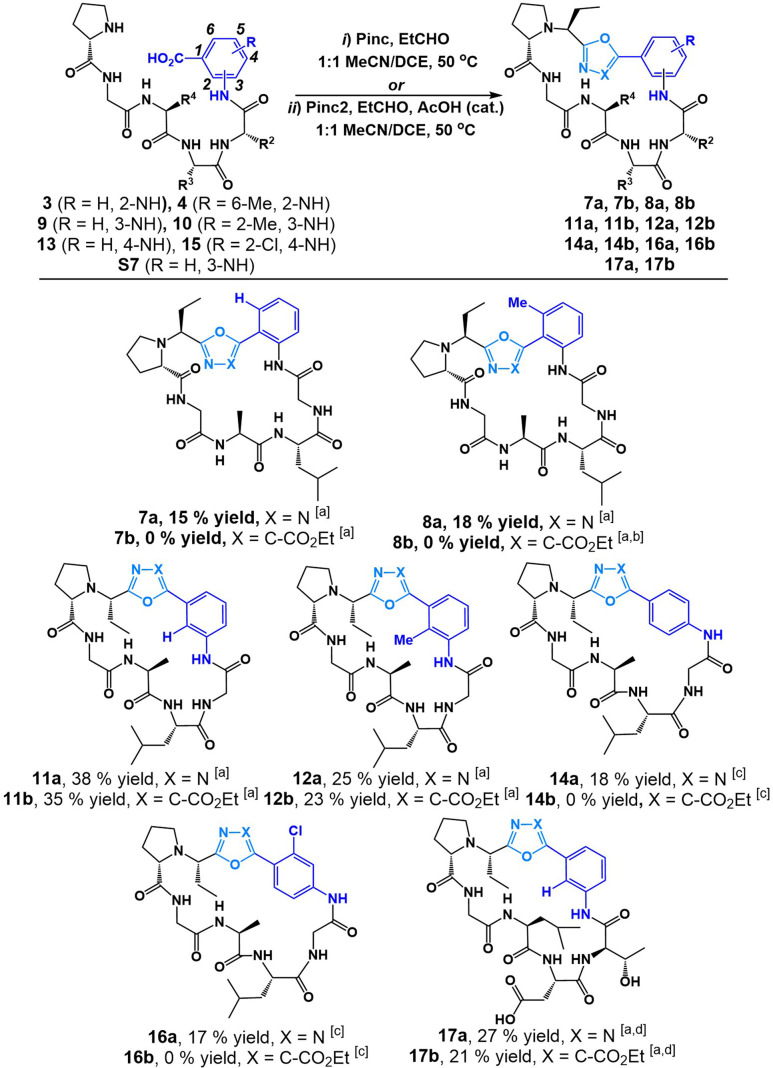
Cyclization of linear peptides with isocyanide reagents Pinc and Pinc2. [a] Standard reaction conditions: Pinc (1.2 equiv.), EtCHO (1.5 equiv.) in 1 : 1 MeCN/DCE (25 mM) at 50 °C or Pinc2 (1.5 equiv.), EtCHO (1.5 equiv.), AcOH (0.1 equiv.) in 1 : 1 MeCN/DCE (25 mM) at 50 °C. [b] Cyclic phosphorane 6b observed (see ESI†). [c] Alternative reaction conditions: Pinc (1.5 equiv.), EtCHO (2 equiv.) in 1 : 1 MeCN/DCE (5 mM) at 60 °C or Pinc2 (1.5 equiv.), EtCHO (1.5 equiv.), AcOH (0.1 equiv.) in 1 : 1 MeCN/DCE (5 mM) at 60 °C. [d] After cyclization the peptide was globally deprotected with 3 : 7 TFA/DCM.

We began our investigation into the utility of Pinc and Pinc2 in generating heterobiaryl linkages during a macrocyclization protocol with C-terminal 2-aminobenzoic acid (anthranilic acid, or Ant)–bearing linear peptide sequences. We recognized that typical head-to-tail cyclization at Ant residues are difficult as a result of unavoidable side reactions upon activation of the C-terminus carboxylate. We were keen to see whether our method would circumvent this reactivity and allow for efficient heterobiaryl forming macrocyclization at the Ant C-terminus. Linear Ant-containing peptide 3 and methylated Ant-containing peptide 4 were prepared *via* routine Fmoc SPPS employing mild Fmoc removal conditions (6% piperazine in a 0.1 M 6-ClHOBt DMF solution for 2 × 10 min). Gratifyingly, linear peptides 3 and 4 smoothly underwent cyclization with Pinc and EtCHO in 1 : 1 DCE/MeCN (25 mM) at 50 °C to afford heterobiarylcontaining cyclic peptides 7a (15% yield) and 8a (18% yield) respectively (Fig. 3). We found that the reaction of 3 and 4 with isocyanide reagent Pinc2 did not result in desired peptides 7b and 8b. Instead, in the case of linear peptide 4, a cyclic phosphorane intermediate (6b in ESI†) was detected by LCMS. This suggested that the ylide intermediate produced by Pinc2 is noticeably less nucleophilic than the imino-phosphorane intermediate afforded by Pinc. We concluded that the electron-rich carbonyl group of Ant-containing peptide sequences was not suitable for cyclization with Pinc2. In stark contrast, we found that cyclization of analogous 3-aminobenzoic acid sequences 9 and 10 produced both oxadiazole heterobiaryl 11a (38% yield) and 12a (25% yield) as well as oxazole heterobiaryl 11b (35% yield) and 12b (23% yield). No cyclic phosphorane was detected in either case. Finally, we tested the reaction on 4-aminobenzoic acidcontaining peptides 13. We recognized that this reaction was challenging as a result of the electronrich nature of the C-terminus carbonyl as well as the inflexible 4-aminobenzoic acid moiety. Indeed, under the standard reaction conditions, no desired peptide was observed using both isocyanide reagents Pinc and Pinc2. In both reactions, an enamine intermediate could be observed and isolated (see ESI†). We noticed that 13 was poorly soluble under our standard reaction conditions. The cyclization was thus conducted at 5 mM while heating to 60 °C with additional portions of Pinc and EtCHO added. This variation led to full conversion of peptide 13 and afforded the desired oxadiazole biaryl 14a (18% yield). Much like with the 2-aminobenzoic acid peptides, the reaction of 13 with isocyanide Pinc2 failed; although, no cyclic phosphorane was detected in this instance. We were content with the applicability of the reaction towards electron-rich, methylated aminobenzoic acid-containing linear peptides. However, we were unsure how the reaction would perform on aminobenzoic acid derivatives bearing electron-withdrawing group. Thus, we tested the reaction on 4-amino-2-chlorobenzoic acid peptide 15 and were found the cyclization to proceed smoothly with Pinc at 5 mM reaction dilution conditions to afford 16a (17% yield). In all cases, the *S*-isomer (at the Et group) was the major diastereomer generated during macrocyclization.

As previously mentioned, macrocycle embedded oxadiazole-grafts have historically proven to be unique and effective structural control elements in peptide macrocycles.^7,8^ A predictable and invariable beta-turn stabilizing effect can be observed in most oxadiazole-grafted macrocycles. In 2018, our group showed that a hydrogen bond between the endocyclic amine and the amide proton of the subsequent amino acid residue is common to every peptide of this group that has been made thus far.^7^ Given this, we were keen to examine the degree to which the peptide backbone could be modulated using the structurally diverse and synthetically accessible heterobiaryl-grafted peptide macrocycles of Fig. 3. We suspected that both alteration of the heterobiaryl *ϕ*-angle as well as the substitution pattern of the heterobiaryl linkage would lead to measurable downstream structural effects. To determine the conformation of each macrocycle, conformational searches were performed using restraints from NOE-derived interproton distances, and backbone dihedral angles extracted from the NH/α-proton scalar coupling constants in *d*_6_-DMSO to generate crude structures. These structures were then subjected to 100 ns of solvent explicit molecular dynamics (OPLS4) simulations, and the resulting trajectory was clustered to determine the most populated conformers. Variable-temperature (VT) ^1^H NMR analysis was also utilized to evaluate intramolecular hydrogen bonding (IMHB) networks between specific amide residues. Typically, temperature coefficients greater than 4 ppb K^−1^ indicate that an amide NH is non-solvent exposed or involved in IMHB. The most populated conformer that best matched the NOE distance restraints/VT ^1^H NMR was deemed the preferred solution structure.

**Fig. 4:**
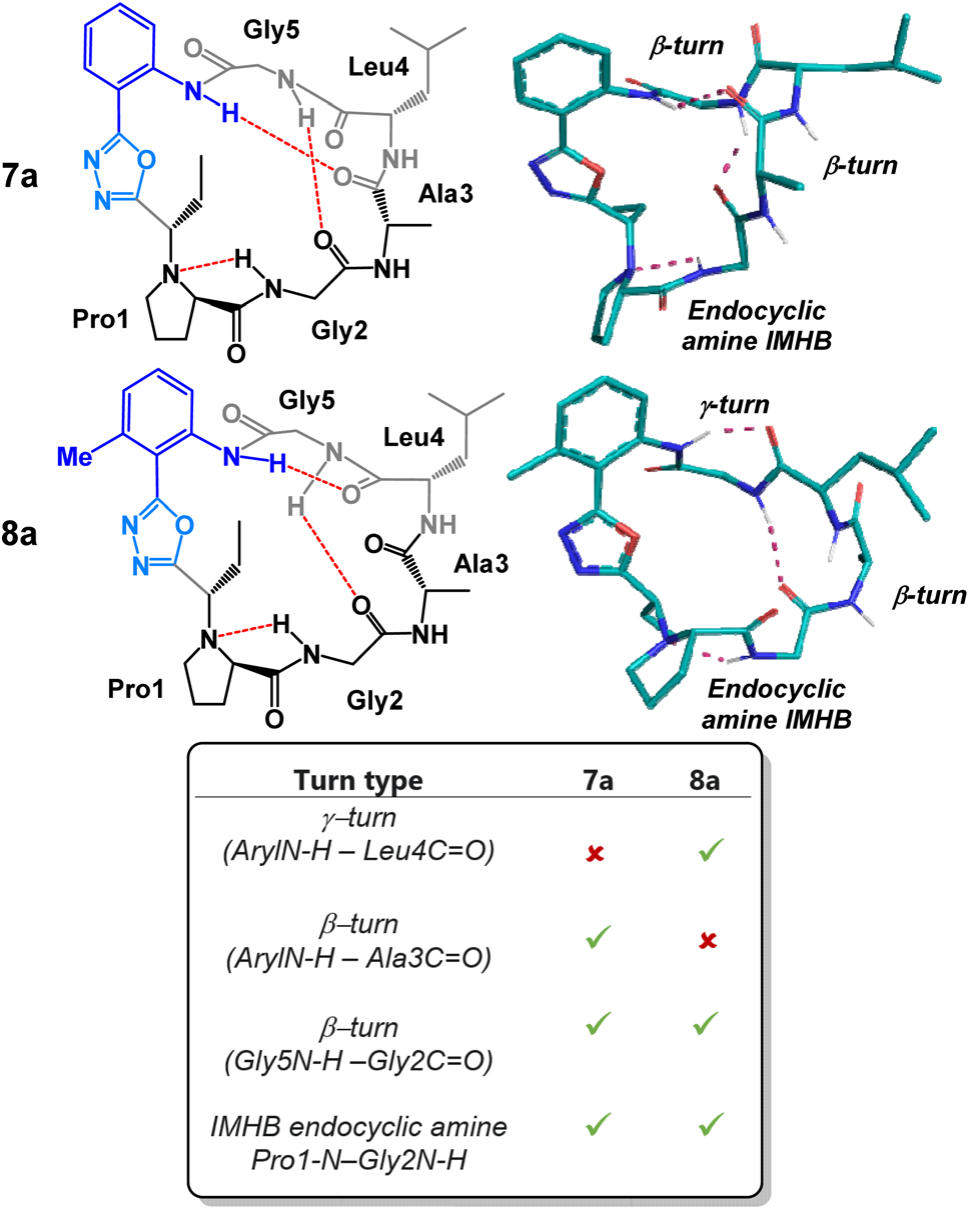
Structural analysis of 2-aminobenzoic acid derived peptides 7a and 8a.

**Fig. 5:**
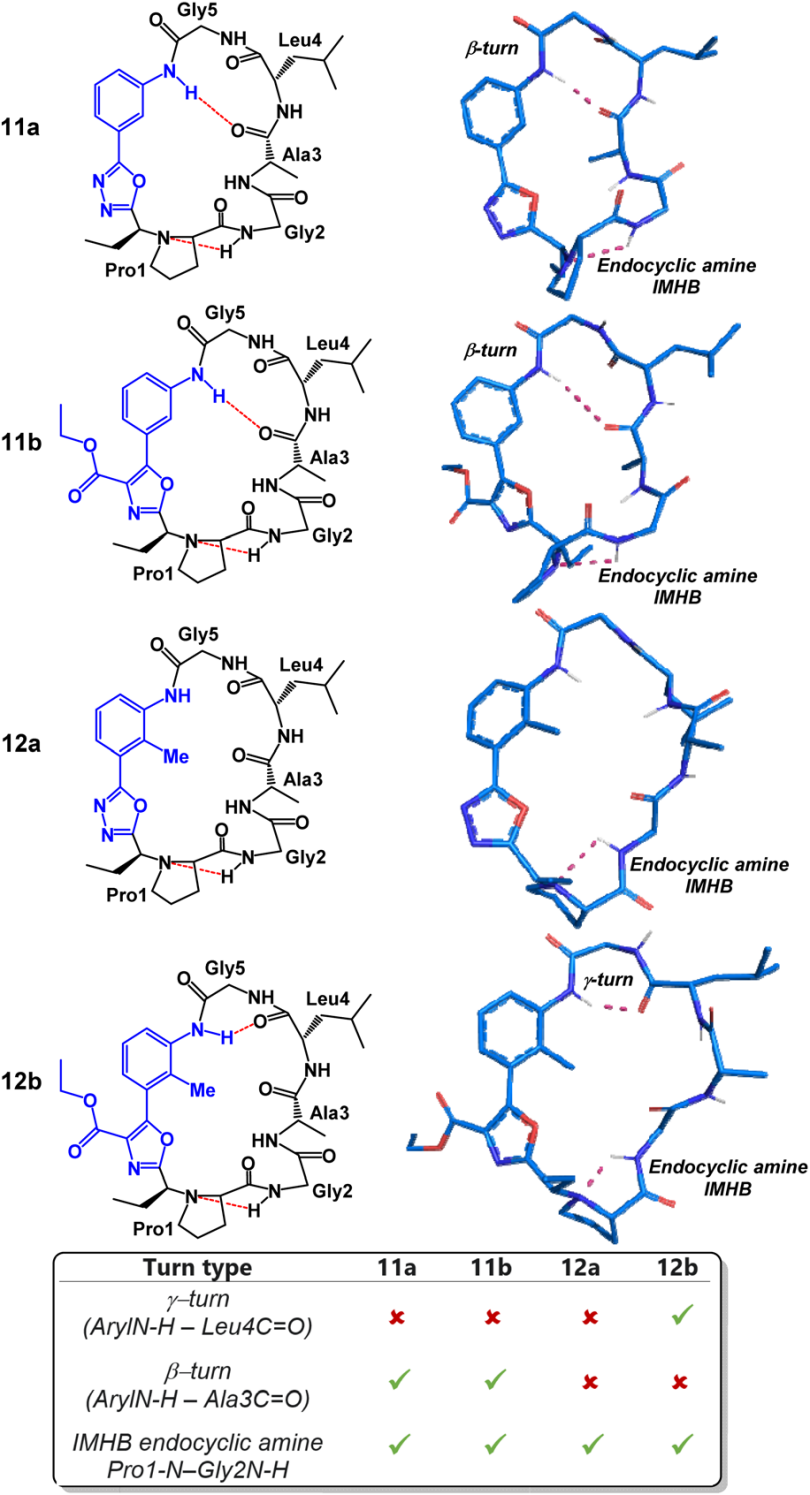
Structural analysis of 3-aminobenzoic acid derived peptides 11a, 11b, 12a and 12b.

We began our structural investigation with peptides 7a and 8a (Fig. 4). Both solution structures possessed key similarities and differences. Most notably, the characteristic endocyclic amine IMHB between the NH of Gly2 and the nitrogen of Pro1 was retained in both compounds. Likewise, both structures fostered β-turns between the NH of Gly5 and the carbonyl of Gly2. The noticeable difference between the two structures was a γ-turn found in peptide 8a in place of a β-turn at the anilide NH in 7a. To examine the structural effects of altering the heterobiaryl *ϕ*-angle as well as the substitution pattern of the heterobiaryl linkage further, we turned our attention toward peptide 11a, 11b, 12a and 12b. As previously illustrated, this group of peptides were made using both reagents Pinc and 2. Indeed, a high degree of structural modulation was obtained in this group of peptides (Fig. 5). The anilide NH of 11a and 11b were both involved in formation of well-defined β-turns. However, slight variations were measured in both the *ϕ* and *ψ* dihedral angles of the *i* + 1 and *i* + 2 residues. In peptide 12a, substitution of a methyl group on the aryl ring resulted in complete disruption of any turn motif at the anilide NH. In contrast, methylated oxazole-containing 12b was found to contain a tight γ-turn in place of the β-turn observed in 11a and 12b. This trend indicated that a bulky methyl substituent pointing into core of the macrocycle leads to disruption of β-turn-like transannular IMHB and fosters the formation of tighter/shorter turns. Much like with the peptides of Fig. 4, peptide 11a, 11b, 12a and 12b all possessed the classical endocyclic amine IMHB between the NH of Gly2 and the reduced amide of Pro1.

**Fig. 6:**
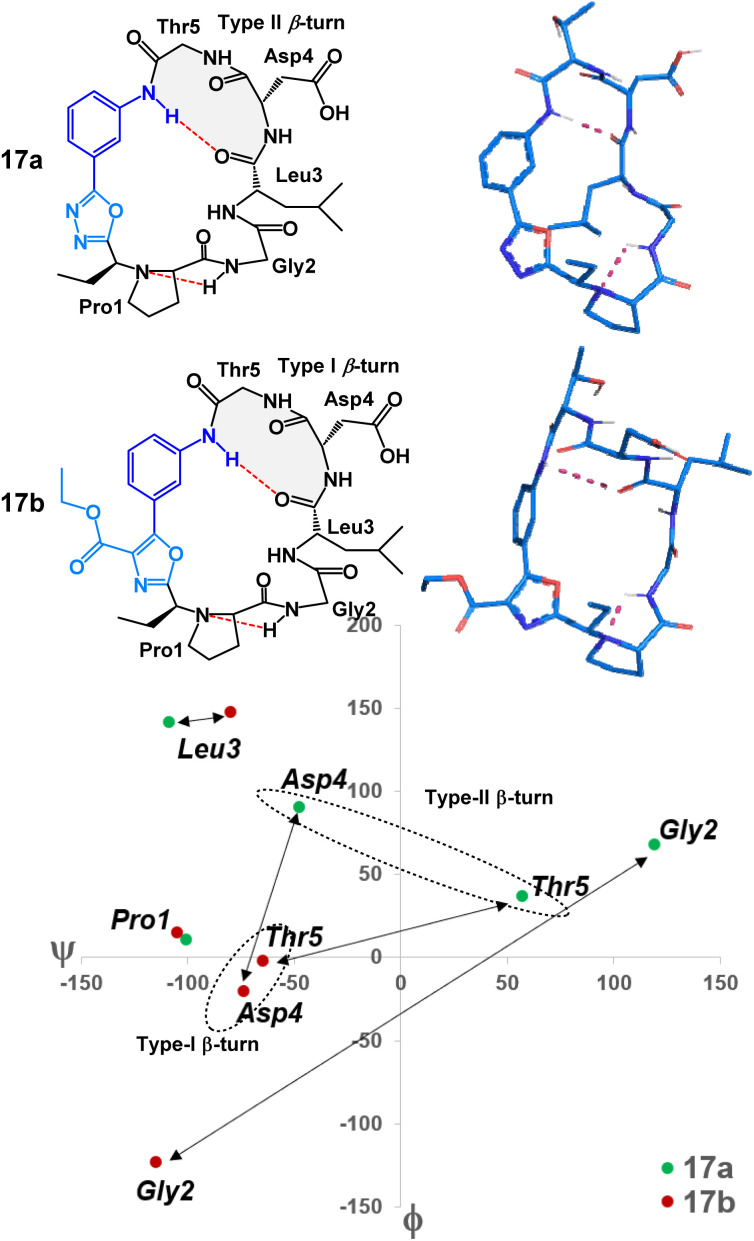
MCM structural analysis of LDT-containing 17a (green) and 17b (red). Double sided arrows reflect the difference in position of each peptide's residue on the Ramachandran plot. Residues within the doted circle compose each peptides β-turn moiety (highlighted in grey).

To examine the nuanced differences of oxazole- and oxadiazole-containing heterobiaryl sequences, we prepared two structural mimics of mucosal addressin cell adhesion molecule-1 (MAdCAM-1) – peptides 17a and 17b. Previous studies have shown that macrocyclic peptides with a Leu-Asp-Thr (LDT) motif as part of a β-turn were able to mimic the structural features of MAdCAM-1 and act as an antagonist for the α_4_β_7_ integrin receptor.^7^ We prepared peptides 17a and 17b (Fig. 3) with LDT containing sequences placed so that the Leu residue would theoretically be located at position *i* of the β-turn (Fig. 6). The cyclization of these 3-aminobenzoic acid derived peptides (S7) proceeded smoothly. Peptides 17a and 17b were obtained after acid-mediated removal of the side chain *tert*-butyl protecting groups in yields of 27% and 21%, respectively. Structurally, both peptides did in fact display the predicted LDTcontaining β-turn with the Leu at position *i*. However, each peptide displayed a unique conformation. This can be visualized through MCM analysis in Fig. 6.^17^ Peptide 17a occupies both positive and negative *ϕ*/*ψ* space whereas 17b occupies only negative *ϕ*/*ψ* space. The large differences in amino acid dihedral angles can likely be attributed to the *ϕ*-dihedral angle of the heterobiaryl linkage in both 17a and 17b. The relatively flat (*ϕ* = 2.2°) of 17a is contrasted by the non-planar 17b (*ϕ* = 38.0°). The differences in amino acid and heterobiaryl dihedral angles of 17a and 17b manifest themselves in the β-turn of the LDT motif with a type II-β-turn in 17a and a type I-β-turn in 17b. Further, we found that the relative polarity of either peptide could be controlled based on the heterobiaryl-graft. We found that the relative reversed-phase HPLC retention times of 17a and 17b differed by over 0.6 minutes on a 15 minute gradient (5.71 minutes and 6.34 minutes respectively). In other studies, relative reversed-phase HPLC retention times have proven to be a useful metric in determining the permeability of peptide macrocycles, as longer retention times typically equate to lower aqueous solubility, lower polarity and increased lipophilicity.^10,14–16^ This suggests that, despite possessing a similar conformation to 17a, the oxazole heterobiaryl 17b is noticeably more non-polar and likely lipophilic when compared to oxadiazole heterobiaryl 17a.

**Fig. 7:**
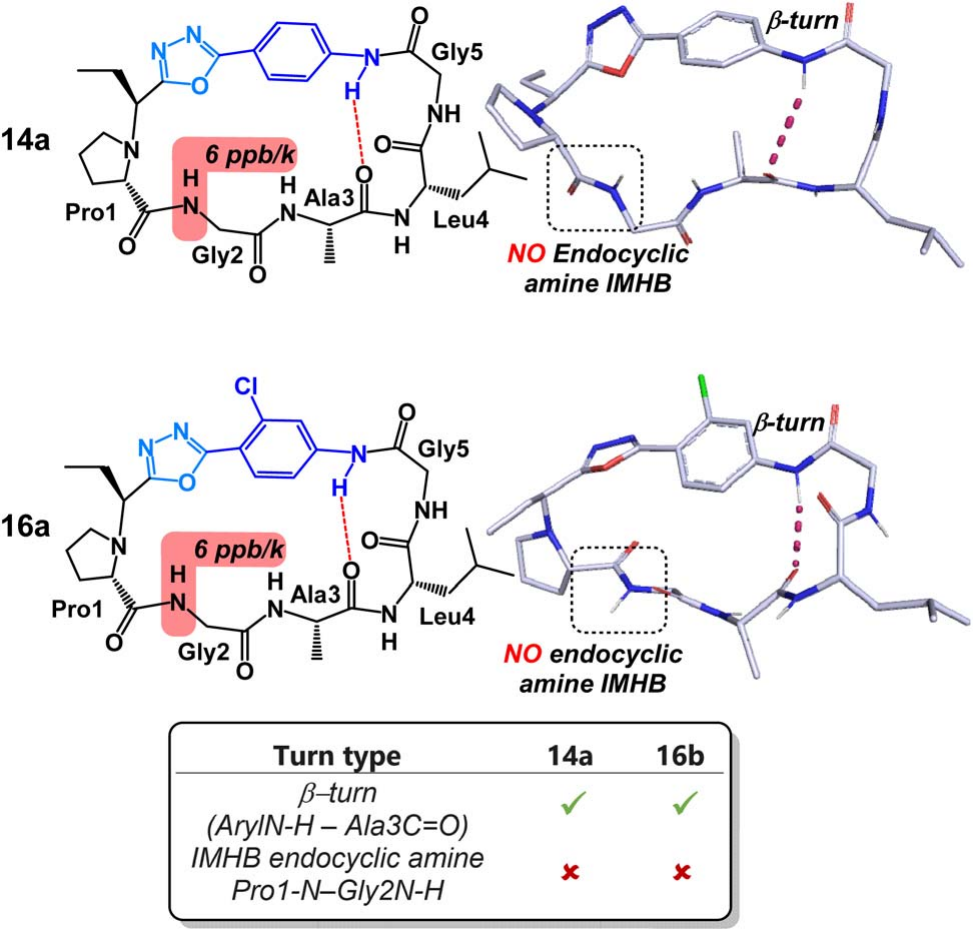
Structural analysis of 4-aminobenzoic acid derived peptides 14a and 16a.

Content with our ability to tune peptide conformation using reagents Pinc and 2, we finally turned our attention towards the structures of 4-amino benzoic acid derived peptides 14a and 16a (Fig. 7). Unlike other peptides examined in this study, substituents on the aryl ring had little structural impact on the peptide conformation. Both 14a and 16a possessed near identical turn motifs and secondary structures. Perhaps more intriguingly, both peptides lacked the IMHB between the NH of Gly2 and the endocyclic amine of Pro1. As far as we can tell, this is the only example in which this rigid hydrogen bond has been disrupted through contortion of the peptide backbone. Disruption of this reduced amide IMHB was verified through our variable temperature NMR experiments. Typically, in the other peptides in this study, the temperature coefficient of the NH of Gly2 was determined to be <1 ppb K^−1^. Remarkably, in peptides 14a and 16a, this same temperature coefficient was determined to be 6 ppb K^−1^.

## Conclusions

In summary, we have developed a new cyclization strategy to prepare heterobiaryl-containing peptides using oxadiazole-forming isocyanide reagent Pinc as well as oxazole-forming isocyanide reagent 2. We have shown that two structurally distinct heterobiaryl peptide macrocycles (either oxadiazole- or oxazole-containing) can be prepared from a single linear peptide sequence. Through structural analysis, we have determined that structural modulation can be achieved through alteration of the *ϕ*-dihedral angle of the heterobiaryl linkage. We have demonstrated that oxazole heterobiaryl linkages produced from reagent Pinc2 prefer a non-planar orientation that leads to downstream structural effects unlike those observed in oxadiazole heterobiaryl peptides produced from reagent 1. We have also found that the lipophilicity of heterobiaryl-containing peptides can be altered depending on the nature of the linkage where the oxazole heterobiaryl-containing peptides is noticeably more non-polar to the analogous oxadiazole heterobiaryl-containing peptide. In addition, in 4-aminobenzoic acid derived peptides, we have shown that incorporation of an oxadiazole heterobiaryl linkage results in an unprecedented disruption of the reduced amide IMHB in products 14a and 16a, respectively. To the best of our knowledge, this is the only example in which an otherwise rigid reduced amide IMHB has been entirely perturbed as a result of peptide backbone contortion. Our work serves as a framework for the rapid synthesis of heterobiaryl-containing peptide libraries with distinct and tuneable secondary structures which has potential significance in the development of bioactive peptide macrocycles.

## Author contributions

M. D. and A. K. Y. conceived the project and wrote the manuscript. M. D. and N. W. H. designed and conducted the experiments. All authors have given approval to the final version of the manuscript.

## Conflicts of interest

A. K. Y. is an associate editor of Chemical Science.

## Supplementary Material

SC-014-D3SC03058J-s001
