# Supplementary material for: Nitrilium ion trapping as a strategy to access structurally diverse heterobiaryl-containing peptide macrocycles
Source: Chem Sci. 2023 Aug 9;14(35):9482–7. doi: 10.1039/d3sc03058j (PMC10498670; doi:10.1039/d3sc03058j)
Supplement: SC-014-D3SC03058J-s001 [file SC-014-D3SC03058J-s001.pdf]

Electronic Supplementary Material (ESI) for Chemical Science.
This journal is © The Royal Society of Chemistry 2023

SUPPORTING INFORMATION

Nitrilium Ion Trapping as a Strategy to Access Structurally Diverse Heterobiaryl-containing Peptide Macrocycles

Matthew Diamandas, Nicholas W. Heller, Andrei K. Yudin*

Table of Contents

General Experimental Method	3
Experimental Procedure: Building block syntheses	4-5
Building Block NMR Spectra	6-11
Experimental Procedure: Solid-Phase Peptide Synthesis	12-13
Full Characterization of compound 7a-17b	14-63
Isolated Enamine Byproduct (compound S8)	64
LC Traces	65-69
LC Trace of Compound 6b	70
Conformational Analysis	71-76
Macrocycle Conformation Maps	77
References	78

General Experimental Method

All reagents were utilized as received from commercial sources unless otherwise noted. All solvents were of reagent grade quality and freshly distilled prior to use. Dichloromethane was distilled over CaH_2 indicator under an atmosphere of nitrogen. Chromatography: Flash-column chromatography was performed using Merck silica gel 60 (40- 63 µm). Thin-layer chromatography (TLC) was performed using Merck silica gel 60 F 254 plates, with UV (254 nm) detection followed by $KMnO_4$ or Ninhydrin stain. NMR Spectrometry: All NMR spectra were recorded on either a Bruker DPX300, Bruker AV 300, Bruker AV 400 at 300 K, Agilent 500 MHz DD2 NMR Spectrometer at 298 K or a Varian 600 Unity spectrometer at 298 K. 1H NMR spectra chemical shifts (δ) are reported in parts per million (ppm) referenced to residual protonated solvent peak (DMSO-*d*6 δ = 2.50). Spectral data is reported as follows: chemical shift, multiplicity (s = singlet, d = doublet, t = triplet, q = quartet, dd = doublet of doublets, dt = doublet of triplets, ddt = doublet of doublet of triplets, dtd = doublet of triplet of doublets, m = multiplet, br = broad, h = heptet, dddd = doublet of doublet of doublet of doublets, qd = quartet of doublets, td = triplet of doublets, tt = triplet of triplets), coupling constant (J) in Hertz (Hz), and integration. 13C NMR spectra chemical shifts (δ) are reported in parts per million (ppm) and were referenced to carbon resonances in the NMR solvent (DMSO-*d*6 δ = 39.5). Mass Spectrometry: High-resolution mass spectrometry (HRMS) ESI (m/z) spectra were recorded on a Bruker MicroTof or an Orbitrap LTQ XL (Nanospray) of Thermo Scientific. At the University of Toronto high resolution mass spectra were obtained on a VG 70-250S (double focusing) mass spectrometer at 70 eV or on an ABI/Sciex Qstar mass spectrometer with ESI source, MS/MS and accurate mass capabilities or on JEOL AccuTOF-DART instrument. RP-HPLC/MS: Low-resolution mass spectra (ESI) were collected on an Agilent Technologies 1200 series HPLC paired to a 6130 Mass Spectrometer. Compounds were resolved on Phenomenex's Kinetex 2.6µ C18 50x4.6mm column at room temperature with a flow of 1 mL/min. The gradient consisted of eluents A (0.1% formic acid in double distilled water) and B (0.1% formic acid in HPLC-grade acetonitrile). LCMS method: A linear gradient starting from 5% of B to 95% over 15 min or 6 min at a flow rate of 1.0 mL/min.

Experimental Procedure

Building block syntheses

2-,3- and 4-aminobenzoic acid derivatives used to prepare peptides **7a, 7b, 8a, 8b 11a, 11b, 12a, 12b, 14a** and **16a** were loaded onto 2′-Cl-TrtCl polystyrene resin as dipeptides in order to avoid poor acylation of the first aniline NH_2. The general method to prepare each of these dipeptides (**S1**-**S6**) is depicted in Scheme S1.

Scheme S1. General method to prepare compounds S1-S6.

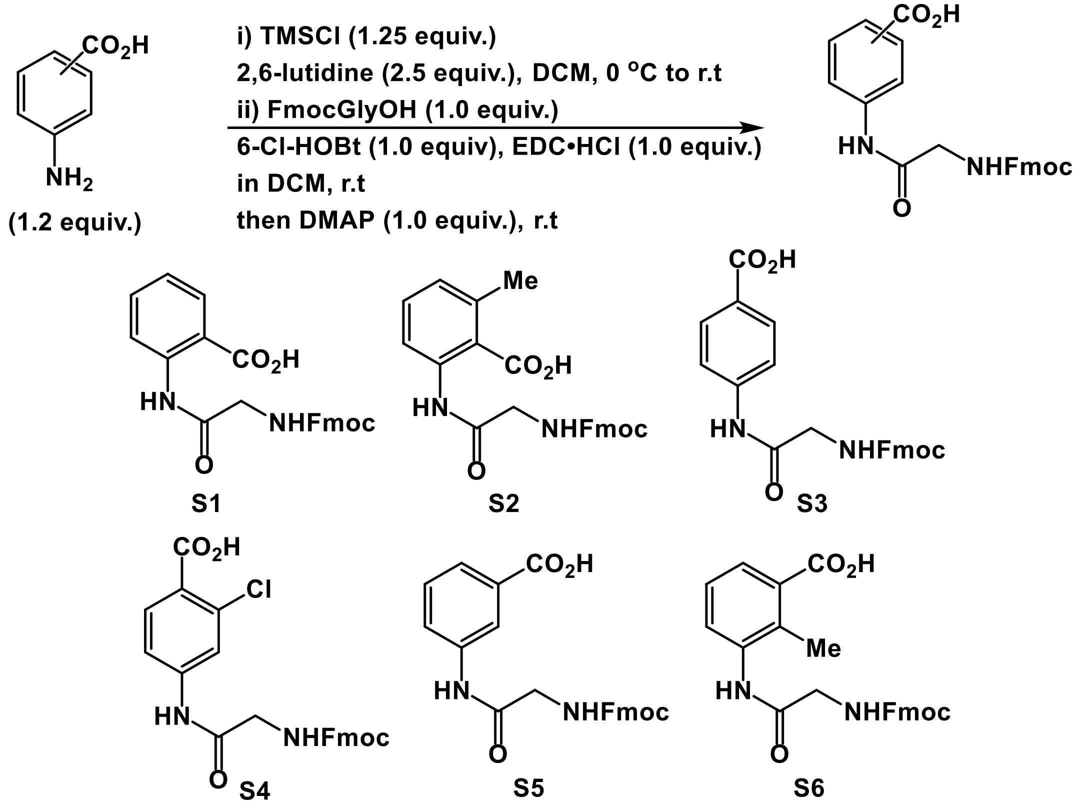

General Procedure for Compounds S1-2. A suspension of 2-aminobenoic acid (14.5 mmol, 1.2 equiv.) in dry DCM (50 mL) at 0 °C was treated dropwise with 2,6-lutidine (3.52 mL, mmol, 2.5 equiv.) followed by TMSCl (1.91 mL, mmol, 1.25 equiv.). The subsequent homogeneous mixture was removed from cooling and stirred at room temperature for 10 min. In a separate flask, EDC•HCl (2.32 g, 12.1 mmol, 1.0 equiv.) was added to a suspension of FmocGlyOH (3.60 g, 12.1 mmol, 1.0 equiv.) and 6-Cl-HOBt (2.05 g, 12.1 mmol, 1.0 equiv.) in dry DCM (50 mL). This mixture was gently agitated at room temperature for 5 min at which point it became clear and homogeneous. Next, the solution of FmocGlyOH/EDC•HCl/6-Cl-HOBt in dry DCM was added to the initial solution of carboxylic acid/2,6-lutidine/TMSCl in dry DCM. The resulting solution was stirred at room temperature for 10 min before solid DMAP (1.48 g, 12.1 mmol, 1.0 equiv.) was added. The resulting solution was allowed to stir at room temperature. After 19 h at room temperature, the reaction was evaporated in vacuo to remove the DCM. The mixture was then diluted with EtOAc (250 mL) and 1 M HCl (250 mL). The organic layer was separated, and the aqueous phase was extracted with EtOAc (3 x 250 mL). Then, the combined organic layers were washed with brine (100 mL), dried (Na_2SO_4), filtered and evaporated in vacuo. Each crude solid was then recrystallized from a mixture of EtOH/H_2O or EtOH/MeOH/H_2O to give each title compound.

General Procedure for Compounds S3-6. A suspension of 2-, 3- or 4-aminobenoic acid (2.00 mmol, 1.2 equiv.) in dry DCM (10 mL) at 0 °C was treated dropwise with 2,6-lutidine (0.49 mL, mmol, 2.5 equiv.) followed by TMSCl (0.26 mL, mmol, 1.25 equiv.). The subsequent homogeneous mixture was removed from cooling and stirred at room temperature for 10 min. In a separate flask, EDC•HCl (320 mg, 1.67 mmol, 1.0 equiv.) was added to a suspension of FmocGlyOH (467 mg, 1.67 mmol, 1.0 equiv.) and 6-Cl-HOBt (284 mg, 1.67 mmol, 1.0 equiv.) in dry DCM (10 mL). This mixture was gently agitated at room temperature for 5 min at which point it became clear and homogeneous. Next, the solution of FmocGlyOH/EDC•HCl/6-Cl-HOBt in dry DCM (10 mL) was added to the initial solution of carboxylic acid/2,6-lutidine/TMSCl in dry DCM (10 min). The resulting solution was stirred at

room temperature for 10 min before solid DMAP (204 mg, 1.67 mmol, 1.0 equiv.) was added. The resulting solution was allowed to stir at room temperature. After 19 h at room temperature, the reaction was evaporated in vacuo to remove the DCM. The mixture was then diluted with EtOAc (50 mL) and 1 M HCl (50 mL). The organic layer was separated, and the aqueous phase was extracted with EtOAc (3 x 50 mL). Then, the combined organic layers were washed with brine (100 mL), dried (Na_2SO_4), filtered and evaporated in vacuo. Each crude solid was then recrystallized from a mixture of EtOH/H_2O or MeOH/H_2O to give each title compound.

Compound S1. The crude solid was recrystallized from 2:1:2 EtOH/MeOH/H_2O (ca. 100 mL), rising with ice cold 1:4 EtOH/H_2O, to give **S1** as a fluffy off-white solid (4.00 g, 71 % yield). ^{1}H NMR (500 MHz, DMSO-*d*6) δ 13.63 (s, 1H), 11.63 (s, 1H), 8.60 (d, *J* = 8.4 Hz, 1H), 8.10 – 7.99 (m, 2H), 7.90 (d, *J* = 7.6 Hz, 2H), 7.74 (d, *J* = 7.5 Hz, 2H), 7.65 – 7.58 (m, 1H), 7.42 (t, *J* = 7.4 Hz, 2H), 7.34 (t, *J* = 7.2 Hz, 2H), 7.20 – 7.14 (m, 1H), 4.36 – 4.23 (m, 3H), 3.83 (d, *J* = 6.0 Hz, 2H). ^{13}C NMR (126 MHz, DMSO-*d*6) δ 169.4, 168.8, 156.8, 143.8, 140.7, 140.5, 134.2, 131.2, 127.7, 127.1, 125.3, 122.8, 120.1, 119.6, 116.2, 66.1, 46.7, 45.5. LC-MS (ESI+) m/z calculated for $C_{25}H_{23}N_2O_5^+$ $[M+H]^+$ = 417.1, found = 417.0.

Compound S2. The crude solid was recrystallized from 2:1:2 EtOH/MeOH/H_2O (ca. 100 mL), rising with ice cold 1:4 EtOH/H_2O, to give **S2** as an off-white solid (4.03 g, 69 % yield). ^{1}H NMR (500 MHz, DMSO-*d*6) δ 13.50 (s, 1H), 9.93 (s, 1H), 7.90 (d, *J* = 7.5 Hz, 2H), 7.73 (d, *J* = 7.3 Hz, 4H), 7.42 (t, *J* = 7.3 Hz, 2H), 7.37 – 7.30 (m, 3H), 7.06 (d, *J* = 7.6 Hz, 1H), 4.39 – 4.14 (m, 3H), 3.79 (d, *J* = 6.1 Hz, 2H), 2.38 (s, 3H). ^{13}C NMR (126 MHz, DMSO-*d*6) δ 169.1, 168.3, 156.6, 143.8, 140.7, 136.9, 136.0, 130.2, 127.6, 127.1, 126.7, 125.3, 120.6, 120.1, 65.9, 46.6, 44.5, 20.9. LC-MS (ESI+) m/z calculated for $C_{25}H_{23}N_2O_5^+$ $[M+H]^+$ = 431.2, found = 431.2.

Compound S3. The crude solid was recrystallized from 1:1 EtOH/H_2O (ca. 20 mL), rising with ice cold 1:4 EtOH/H_2O, to give **S3** as an off-white solid (467.6 mg, 56 % yield). ^{1}H NMR (500 MHz, DMSO-*d*6) δ 12.69 (s, 1H), 10.29 (s, 1H), 7.92 – 7.87 (m, 4H), 7.76 – 7.65 (m, 5H), 7.45 – 7.39 (m, 2H), 7.34 (td, *J* = 7.5, 1.2 Hz, 2H), 4.31 (d, *J* = 7.4 Hz, 2H), 4.25 (t, *J* = 7.0 Hz, 1H), 3.83 (d, *J* = 6.2 Hz, 2H). ^{13}C NMR (126 MHz, DMSO-*d*6) δ 168.54, 166.90, 156.63, 143.85, 142.93, 140.74, 130.45, 127.65, 127.10, 125.26, 125.12, 120.14, 118.33, 65.76, 46.63, 44.13, 40.11, 40.02, 39.94, 39.85, 39.78, 39.69, 39.61, 39.52, 39.44, 39.35, 39.19, 39.02. LC-MS (ESI+) m/z calculated $C_{25}H_{23}N_2O_5^+$ $[M+H]^+$ = 417.1, found = 417.2.

Compound S4. The crude solid was recrystallized from 1:1 EtOH/H_2O (ca. 20 mL), rising with ice cold 1:4 EtOH/H_2O, to give **S4** as an off-white solid (435.6 mg, 48 % yield). ^{1}H NMR (500 MHz, DMSO-*d*6) δ 13.10 (s, 1H), 10.40 (s, 1H), 7.92 – 7.86 (m, 3H), 7.84 (d, *J* = 8.6 Hz, 1H), 7.76 – 7.67 (m, 3H), 7.54 (dd, *J* = 8.6, 2.1 Hz, 1H), 7.45 – 7.40 (m, 2H), 7.34 (td, *J* = 7.4, 1.2 Hz, 2H), 4.34 – 4.30 (m, 3H), 3.83 (d, *J* = 6.1 Hz, 2H). ^{13}C NMR (126 MHz, DMSO-*d*6) δ 168.88, 165.89, 156.64, 143.84, 142.52, 140.75, 133.08, 132.49, 127.66, 127.10, 125.25, 124.53, 120.14, 116.97, 65.78, 46.63, 44.16, 40.11, 40.02, 39.94, 39.85, 39.78, 39.69, 39.61, 39.52, 39.35, 39.19, 39.02. LC-MS (ESI+) m/z calculated $C_{24}H_{20}ClN_2O_5^+$ $[M+H]^+$ = 451.1, found = 451.0.

Compound S5. The crude solid was recrystallized from 1:1 EtOH/H_2O (ca. 20 mL), rising with ice cold 1:4 EtOH/H_2O, to give **S5** as an off-white solid (584.2 mg, 70 % yield). ^{1}H NMR (500 MHz, DMSO-*d*6) δ 12.98 (s, 1H), 10.18 (s, 1H), 8.27 – 8.23 (m, 1H), 7.89 (d, *J* = 7.5 Hz, 2H), 7.85 – 7.81 (m, 1H), 7.74 (d, *J* = 7.5 Hz, 2H), 7.69 – 7.62 (m, 2H), 7.48 – 7.38 (m, 4H), 7.34 (td, *J* = 7.4, 1.2 Hz, 2H), 4.38 – 4.17 (m, 4H), 3.83 (d, *J* = 6.1 Hz, 2H). (126 MHz, DMSO-*d*6) δ 168.29, 167.17, 156.65, 143.88, 140.76, 140.63, 139.14, 131.34, 129.06, 127.66, 127.11, 127.00, 125.27, 124.06, 123.20, 120.13, 119.88, 65.77, 56.06, 46.66, 44.09, 40.11, 40.02, 39.94, 39.85, 39.78, 39.69, 39.61, 39.52, 39.35, 39.19, 39.02, 18.58. LC-MS (ESI+) m/z calculated $C_{25}H_{23}N_2O_5^+$ $[M+H]^+$ = 417.1, found = 417.0.

Compound S6. The crude solid was recrystallized from 1:1 MeOH/H_2O (ca. 20 mL), rising with ice cold 1:MeOH EtOH/H_2O, to give **S6** as an off-white solid (310.6 mg, 30 % yield). ^{1}H NMR (500 MHz, DMSO-*d*6) δ 12.92 (s, 1H), 9.49 (s, 1H), 7.89 (d, *J* = 7.5 Hz, 2H), 7.73 (d, *J* = 7.5 Hz, 2H), 7.67 (t, *J* = 6.2 Hz, 1H), 7.58 (d, *J* = 7.7 Hz, 1H), 7.49 (d, *J* = 7.9 Hz, 1H), 7.42 (t, *J* = 7.5 Hz, 2H), 7.33 (t, *J* = 7.4 Hz, 2H), 7.26 (t, *J* = 7.8 Hz, 1H), 4.32 (d, *J* = 7.1 Hz, 2H), 4.25 (d, *J* = 6.9 Hz, 1H), 3.86 (d, *J* = 6.1 Hz, 2H), 2.33 (s, 3H). ^{13}C NMR (126 MHz, DMSO-*d*6) δ 169.10, 168.31, 156.59, 143.85, 140.72, 136.97, 132.79, 132.70, 128.70, 127.62, 127.06, 126.78, 125.50, 125.24, 120.11, 65.73, 46.63, 43.76, 40.00, 39.92, 39.83, 39.76, 39.67, 39.50, 39.33, 39.17, 39.00, 15.05. LC-MS (ESI+) m/z calculated for $C_{25}H_{23}N_2O_5^+$ $[M+H]^+$ = 431.2, found = 431.2.

Figure S1:1H13C-NMR Compound **S1** (500 MHz, DMSO-*d*6)

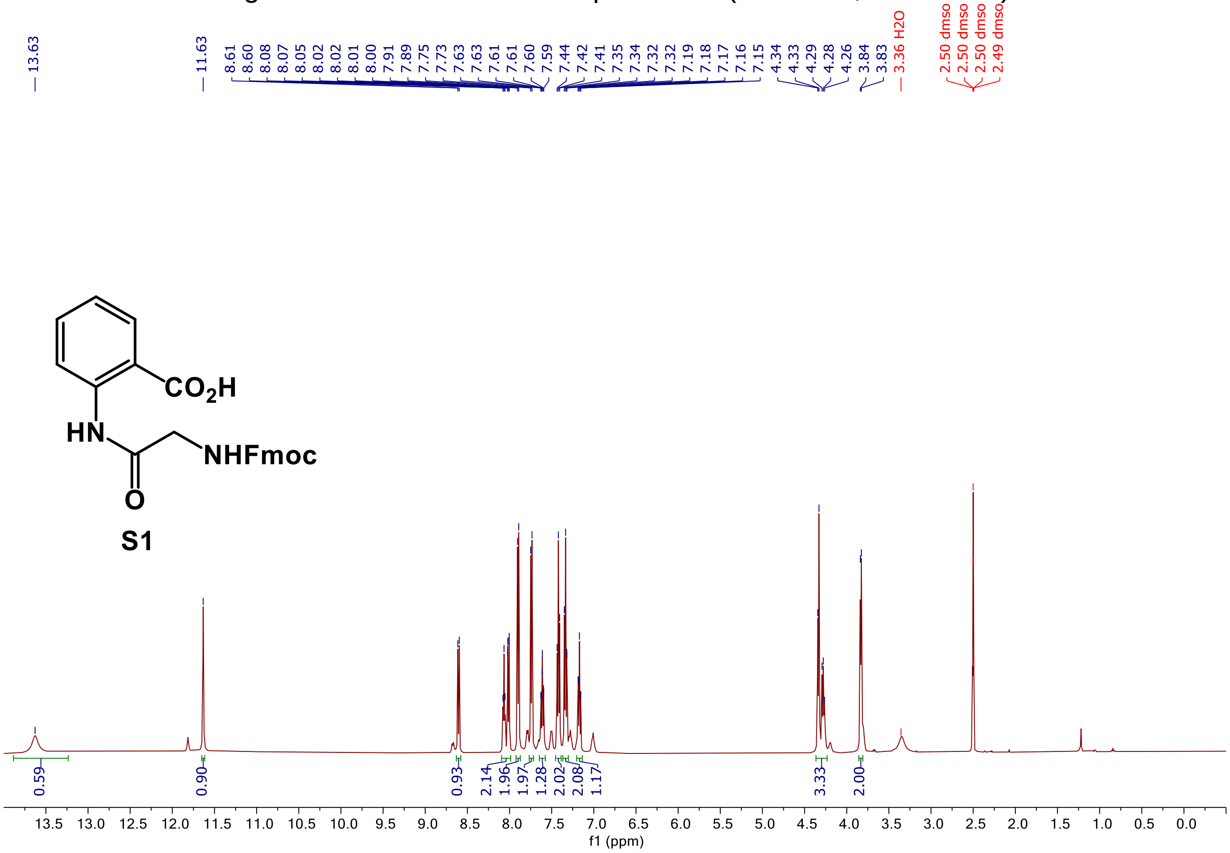

Figure S2: 13C-NMR Compound **S1** (126 MHz, DMSO-*d*6)

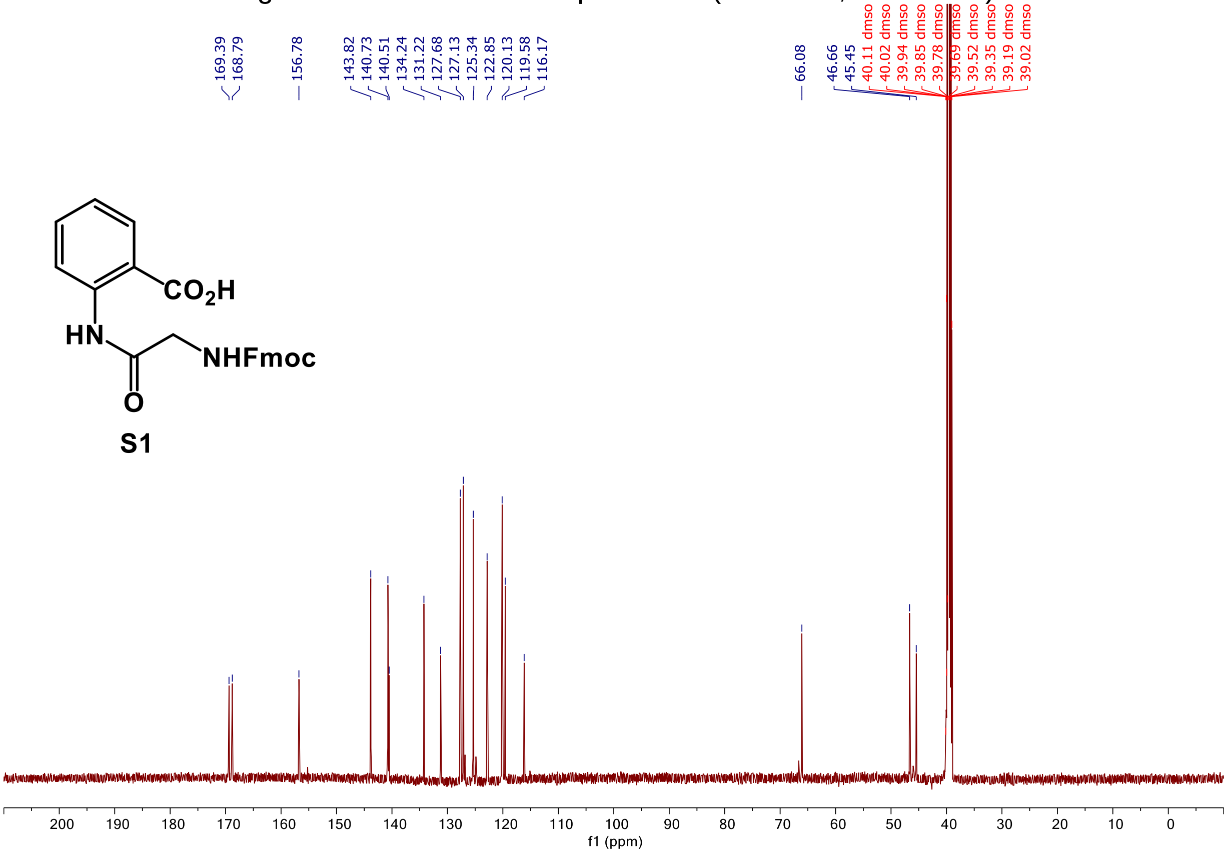

Figure S3: 1H-NMR Compound **S2** (500 MHz, DMSO-*d6*)

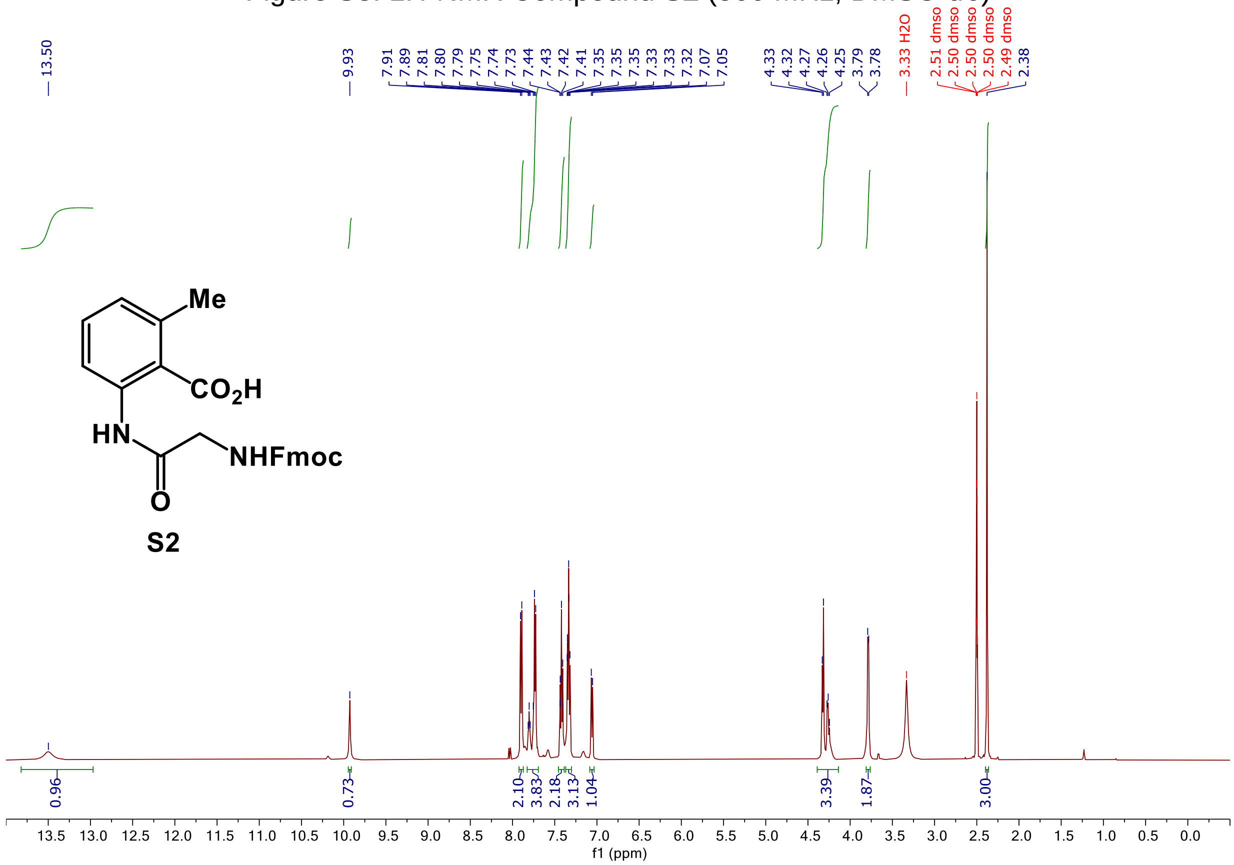

Figure S4: 13C-NMR Compound **S2** (126 MHz, DMSO-*d6*)

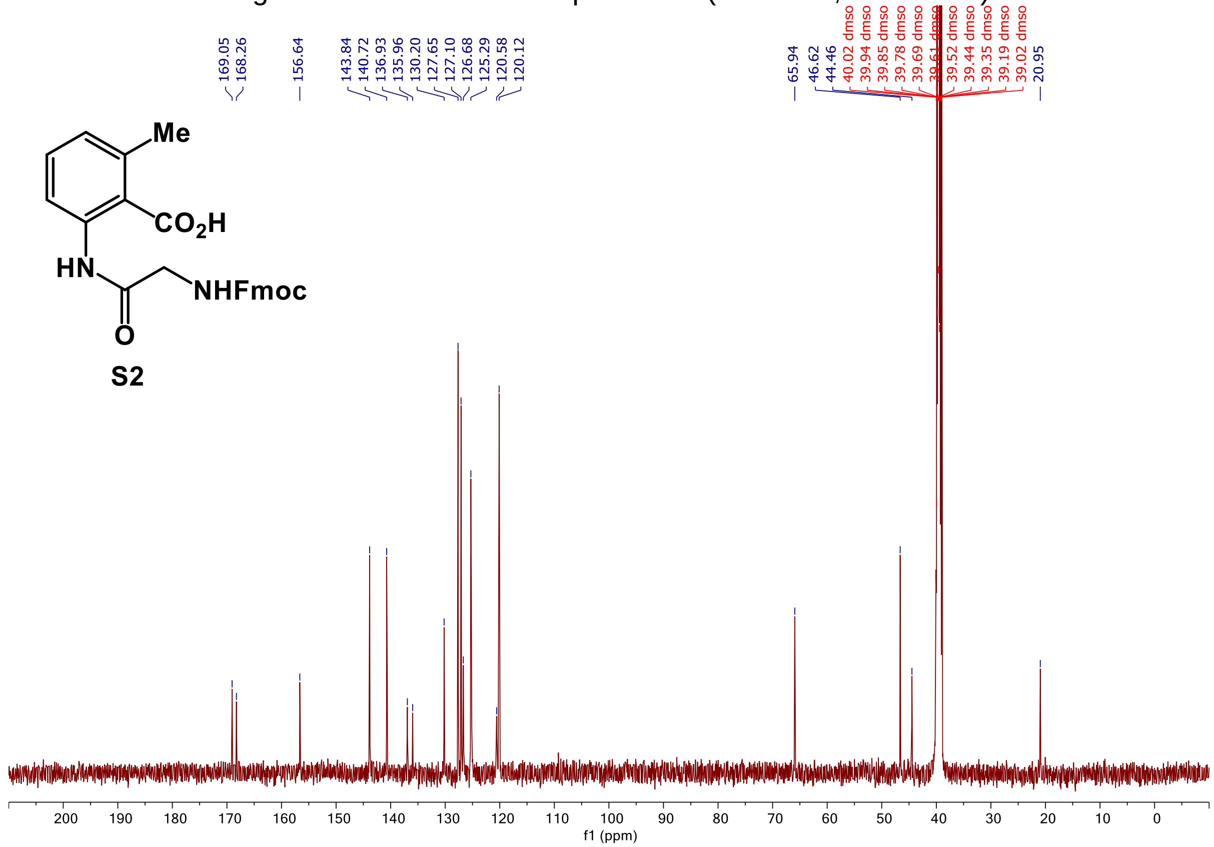

Figure S5: 1H-NMR Compound **S3** (500 MHz, DMSO-*d*6)

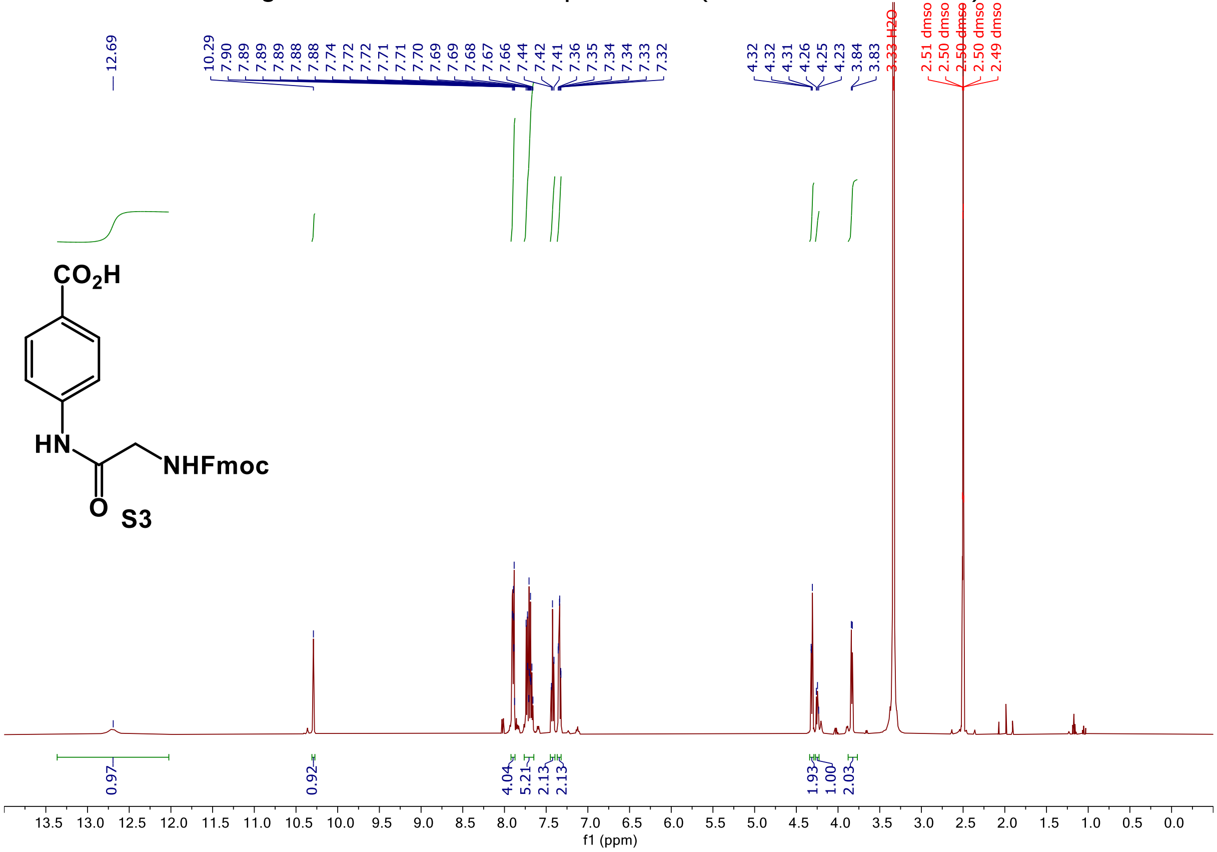

Figure S6: 13C-NMR Compound **S3** (126 MHz, DMSO-*d*6)

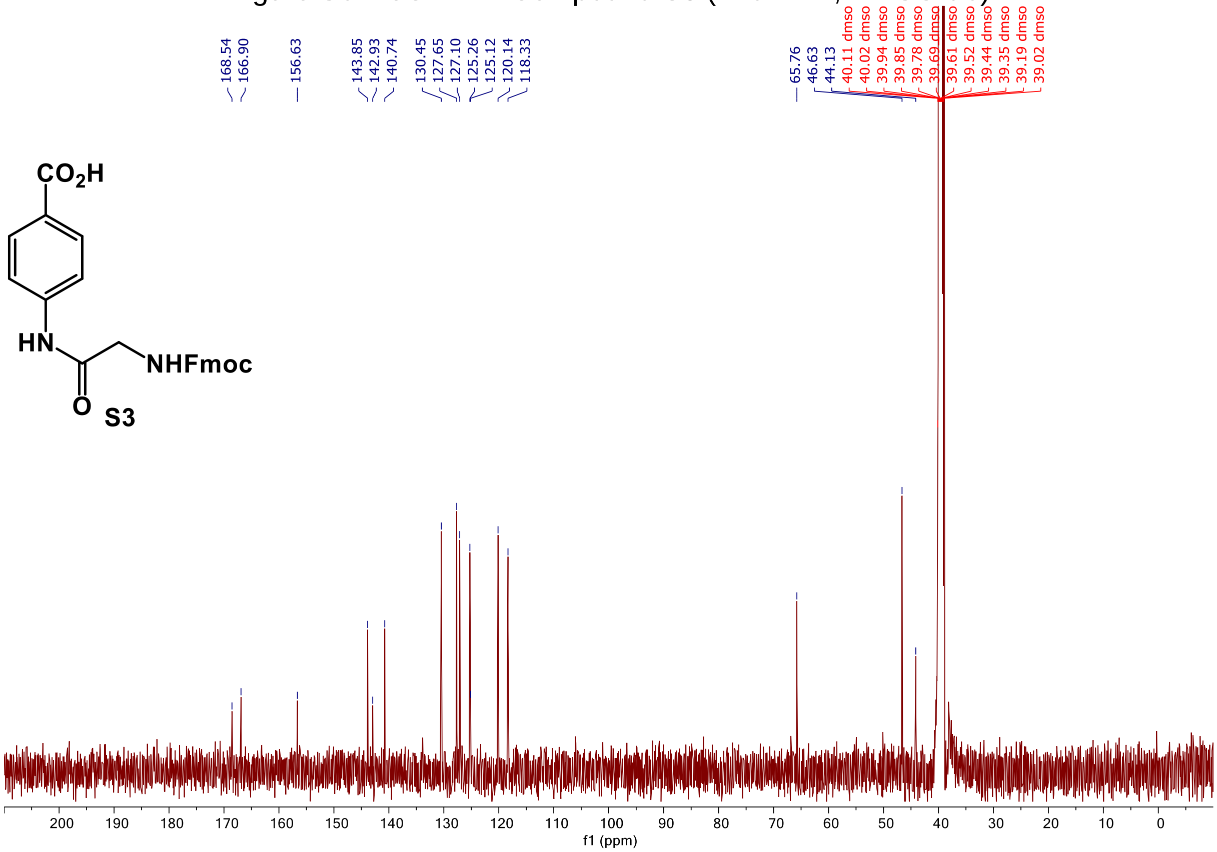

Figure S7: 1H-NMR Compound **S4** (500 MHz, DMSO-*d*6)

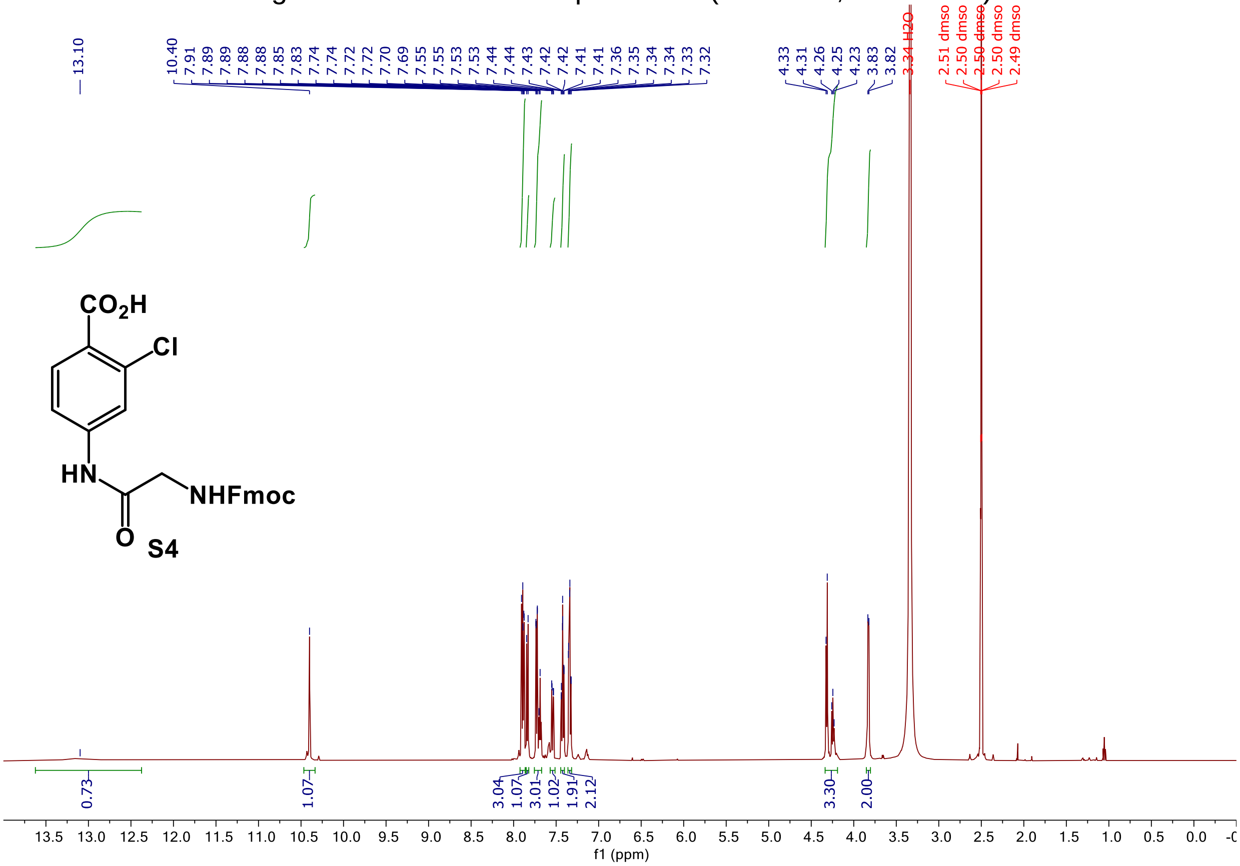

Figure S8: 13C-NMR Compound **S4** (126 MHz, DMSO-*d*6)

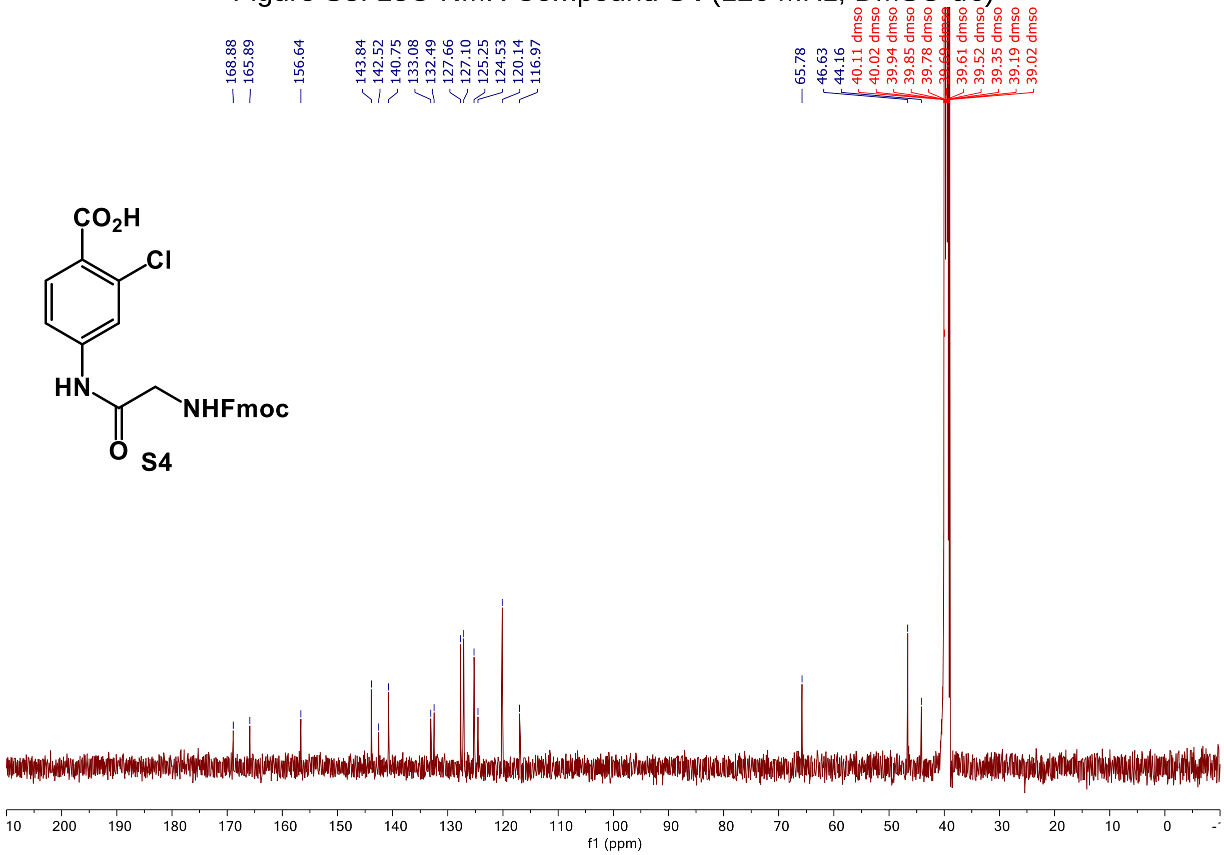

Figure S9: 1H-NMR Compound **S5** (500 MHz, DMSO-*d*6)

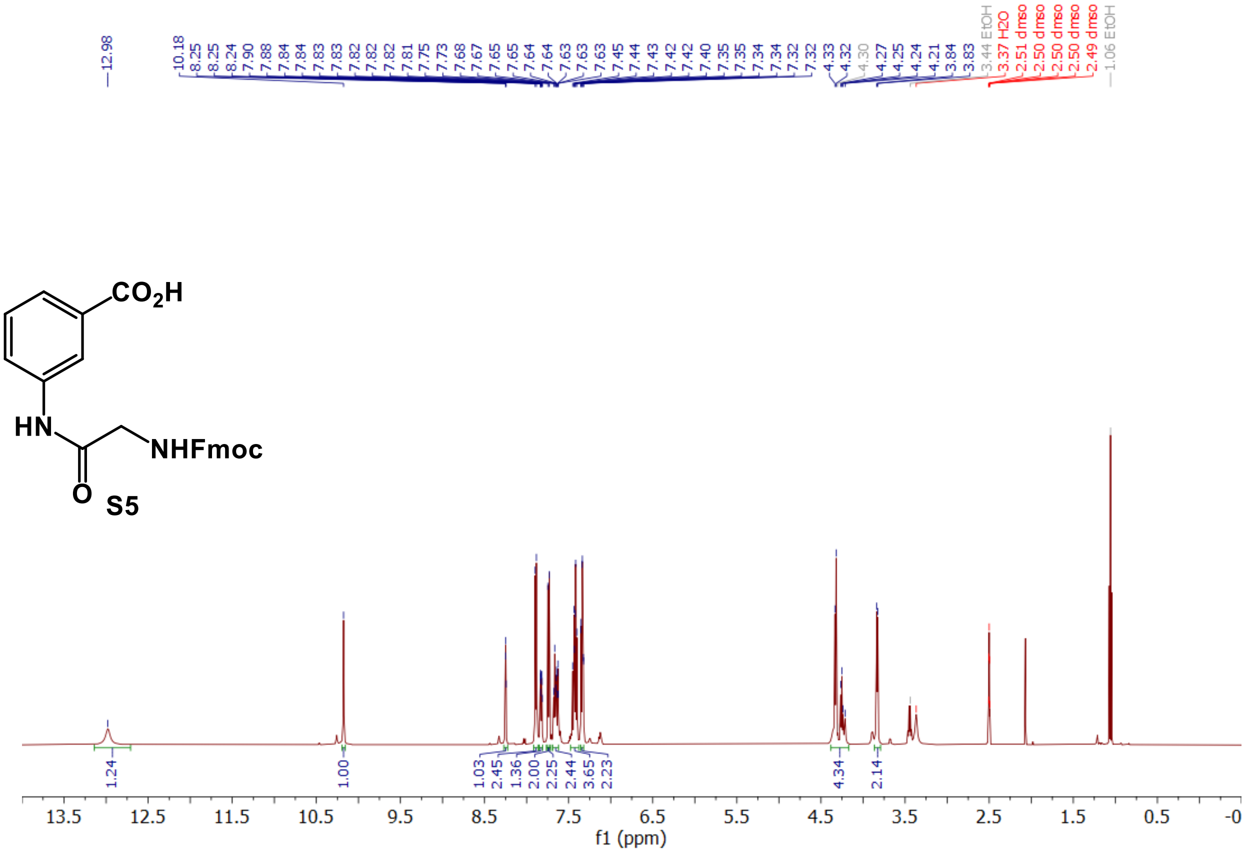

Figure S10: 13C-NMR Compound **S5** (500 MHz, DMSO-*d*6)

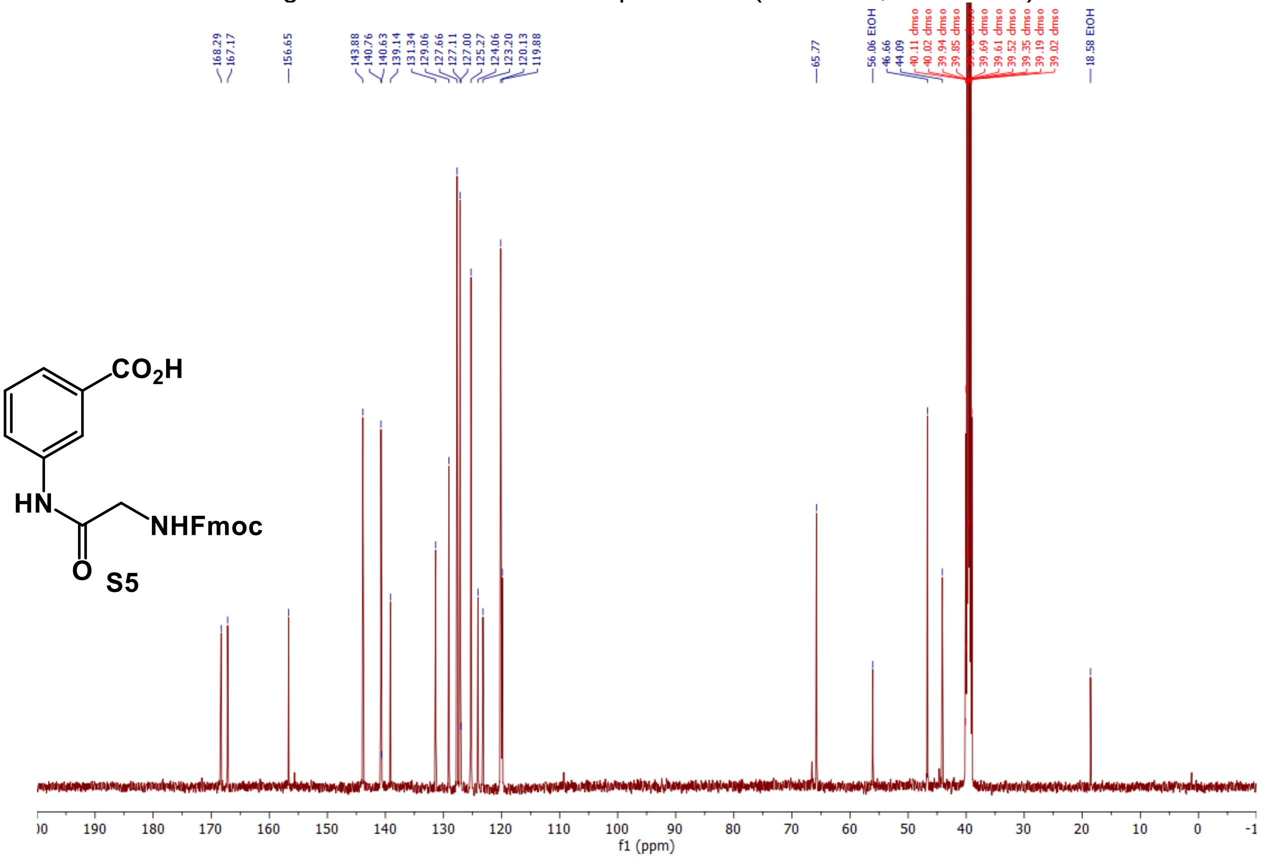

Figure S11: 1H-NMR Compound **S6** (500 MHz, DMSO-*d*6)

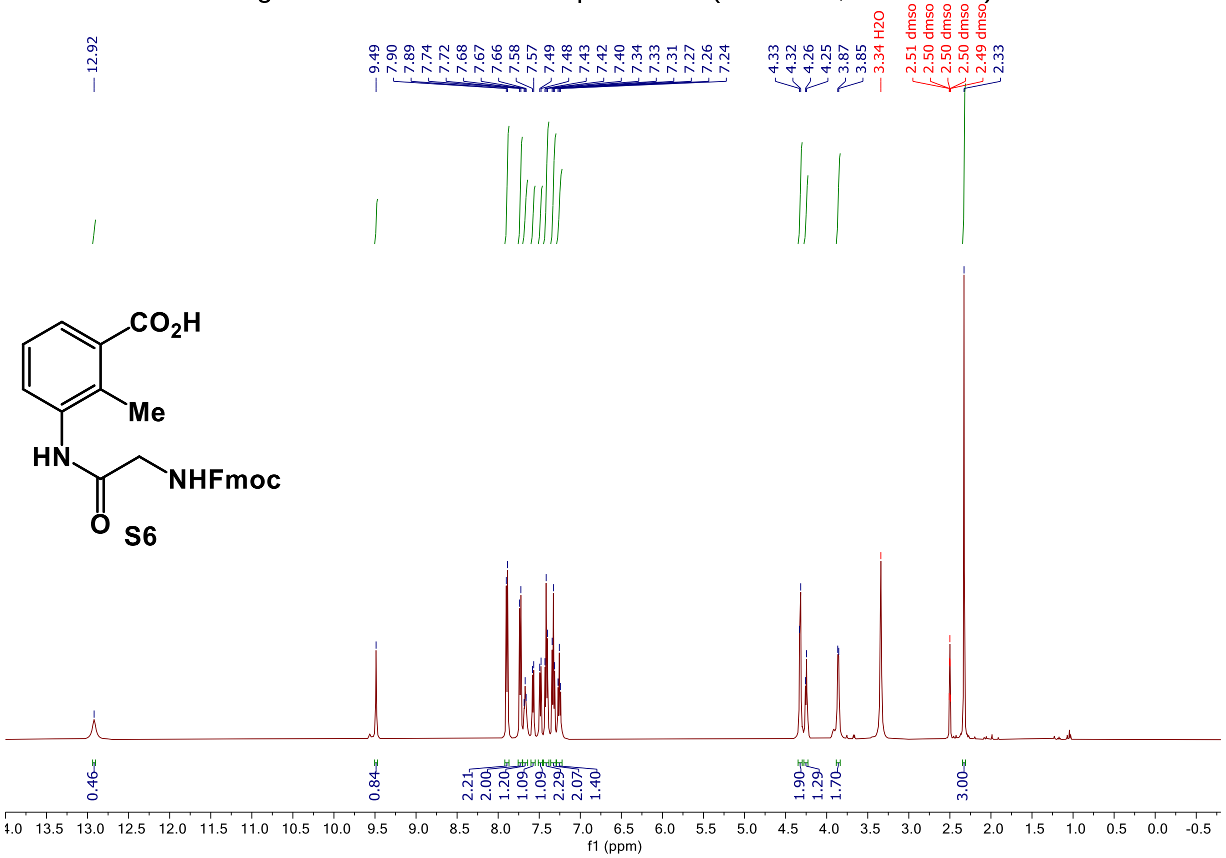

Figure S12: 13C-NMR Compound **S6** (500 MHz, DMSO-*d*6)

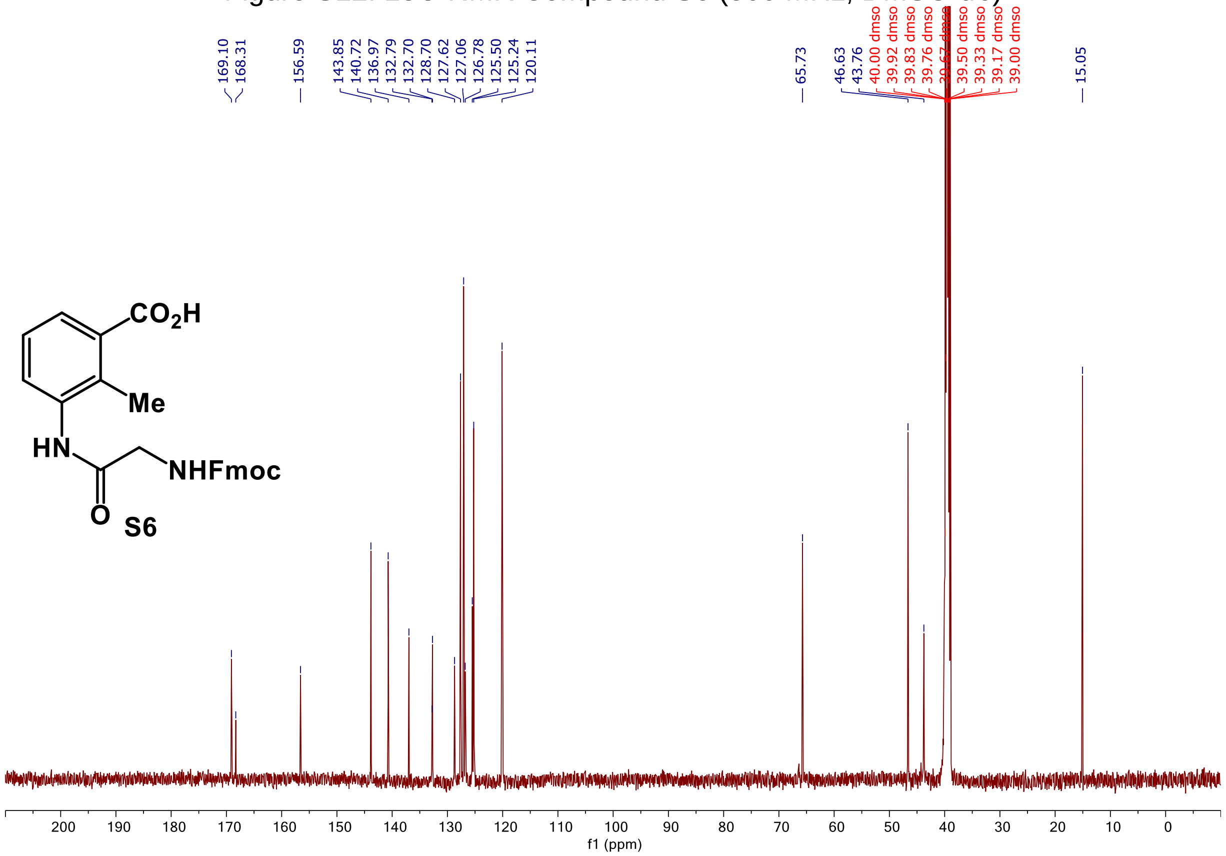

<u>**Solid-Phase Peptide Synthesis**</u>

General Procedure for Resin Loading and Fmoc SPPS (peptides 3, 4, 9, 10, 13, 15)

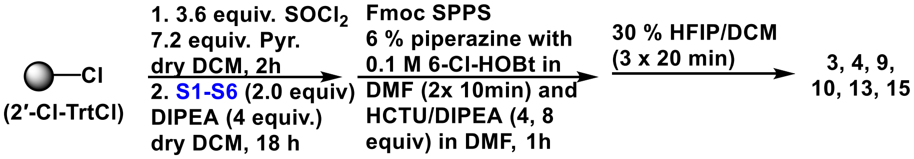

The SPPS synthesis was performed manually beginning from 2′-Cl-TrtCl polystyrene resin (theoretical substitution = 1.1 mmol/g, 181.8 mg, 0.2 mmol, 1 equiv). The 2′-Cl-TrtCl polystyrene resin was preactivated in dry DCM (20 mL) with $SOCl_2$ (26 µL, 3.6 equiv) and pyridine (58 µL, 7.2 equiv) under reflux for 2 h. The resin was then transferred to a disposable peptide cartridge and rinsed with dry DCM, followed by loading with **S1-S6**(2.0 equiv) and DIPEA (4.0 equiv) in dry DCM (2.0 mL) (18 h). The resin was capped with 17:2:1 DCM/MeOH/DIPEA (3 × 10 min. All Fmoc-amino acids (4 equiv) were activated for 5 min using HCTU (4 equiv) and DIPEA (8 equiv) in DMF (2.0 mL) and then coupled for 1 h. All Fmoc groups were removed using 6 % piperazine with 0.1 M 6-Cl-HOBt in DMF (2.0 mL) (2 x 10 min). The global deprotection was executed by treating the resin bound peptide with 30 % hexafluoroisopropanol (HFIP) in DCM (3 x 20 min). The resin was then rinsed with DCM (2x) and then 1 % MeOH in DCM. The cleavage cocktail was condensed under a stream of N_2 to remove volatiles. The peptide residue was then precipitated with ice cold Et_2O. The Et_2O was carefully removed using a stream of N_2 to afford each linear peptide. These linear peptides were subjected to macrocyclization using Pinc and reagent **2**.

General Procedure for Resin Loading and Fmoc SPPS for LDT containing linear peptide S7

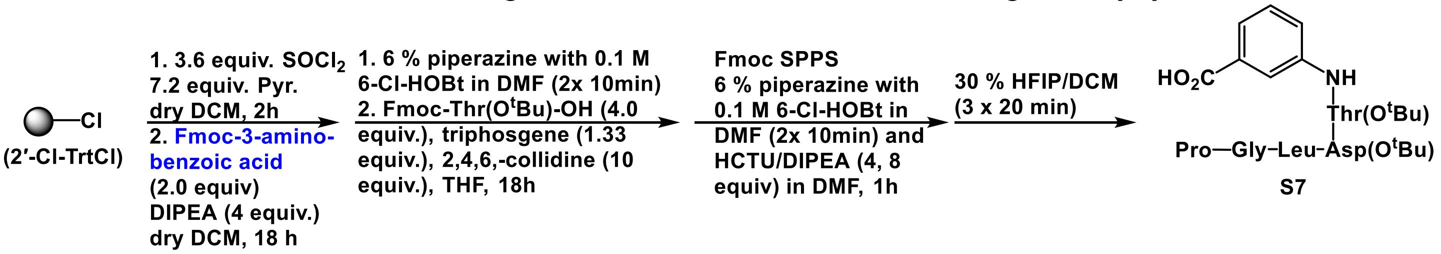

The SPPS synthesis was performed manually beginning from 2′-Cl-TrtCl polystyrene resin (theoretical substitution = 1.1 mmol/g, 181.8 mg, 0.2 mmol, 1 equiv). The 2′-Cl-TrtCl polystyrene resin was preactivated in dry DCM (20 mL) with $SOCl_2$ (26 µL, 3.6 equiv) and pyridine (58 µL, 7.2 equiv) under reflux for 2 h. The resin was then transferred to a disposable peptide cartridge and rinsed with dry DCM, followed by loading with **Fmoc-3-aminobenzoic acid** (2.0 equiv) and DIPEA (4.0 equiv) in dry DCM (2.0 mL) (18 h). The resin was capped with 17:2:1 DCM/MeOH/DIPEA (3 × 10 min. The first Fmoc group was removed using 6 % piperazine with 0.1 M 6-ClH-OBt in DMF (2.0 mL) (2 x 10 min) and then the subsequent Fmoc-amino acid was coupled as follows (to form the aryl amide bond): a solution of Fmoc-Thr(O^{t}Bu)-OH (4.0 equiv) and triphosgene (1.33 equiv) in dry THF (1.5 mL) was added 2,4,6-colidine (10 equiv). Upon the addition of the 2,4,6-collidine a suspension immediately formed. This suspension was gently shaken for 1 minute before it was added to the peptide (previously washed with dry THF x 3). The reaction was gently agitated for 18 h before it was carefully drained. The peptide was then rinsed with THF (1.0 mL x 2), DMF (1.0 mL x 3), 3:1 THF/H2O (1.0 mL x 3), MeOH (1.0 mL x 3) and then DMF (1.0 mL x 3).[1] All subsequent Fmoc-amino acids (4 equiv) were activated for 5 min using HCTU (4 equiv) and DIPEA (8 equiv) in DMF (2.0 mL) and then coupled for 1 h. All Fmoc groups were removed using 6 % piperazine with 0.1 M 6-ClH-OBt in DMF (2.0 mL) (2 x 10 min). The global deprotection was executed by treating the resin bound peptide with 30 % hexafluoroisopropanol (HFIP) in DCM (3 x 20 min). The resin was then rinsed with DCM (2x) and then 1 % MeOH in DCM. The cleavage cocktail was condensed under a stream of N_2 to remove volatiles. The peptide residue was then precipitated with ice cold Et_2O. The Et_2O was carefully removed using a stream of N_2 to afford **S7**. These linear peptides were subjected to macrocyclization using Pinc and reagent **2.**

General Procedure for Peptide Macrocyclization Reactions for peptides 3, 4, 9 and 10

Pinc: A suspension of linear peptide in 1:1 DCE/MeCN (25 mM) at r.t was treated with EtCHO (1.5 equiv.). After 10 min this mixture was treated with Pinc (1.2 equiv.). The dark orange reaction mixture was then heated to 50

^{o}C and stirred for 18 h. After 18 h the reaction mixture was evaporated, and the crude peptide was purified by C18-reversed-phase chromatography and then peak fractions were pooled and lyophilized to afford the desired peptides. In each case the major diastereomer was separated from the minor.

*Reagent **2**:* A suspension of linear peptide in 1:1 DCE/MeCN (25 mM) at r.t was treated with EtCHO (1.5 equiv.) followed by catalytic AcOH (0.1 equiv.). After 10 min this mixture was treated with **2** (1.5 equiv.). The dark orange reaction mixture was then heated to 50 ^{o}C and stirred for 18 h. After 18 h the reaction mixture was evaporated, and the crude peptide was purified by C18-reversed-phase chromatography and then peak fractions were pooled and lyophilized to afford the desired peptides. In each case the major diastereomer was separated from the minor.

General Procedure for Peptide Macrocyclization Reactions for peptides 13 and 15

Pinc: A suspension of linear peptide in 1:1 DCE/MeCN (5 mM) at r.t was treated with EtCHO (2.0 equiv.). After 10 min this mixture was treated with Pinc (1.5 equiv.). The dark orange reaction mixture was then heated to 60 ^{o}C and stirred for 18 h. After 18 h the reaction mixture was evaporated, and the crude peptide was purified by C18-reversed-phase chromatography and then peak fractions were pooled and lyophilized to afford the desired peptides. In each case the major diastereomer was separated from the minor.

Macrocyclization and Global Deprotection of Linear Peptide S7

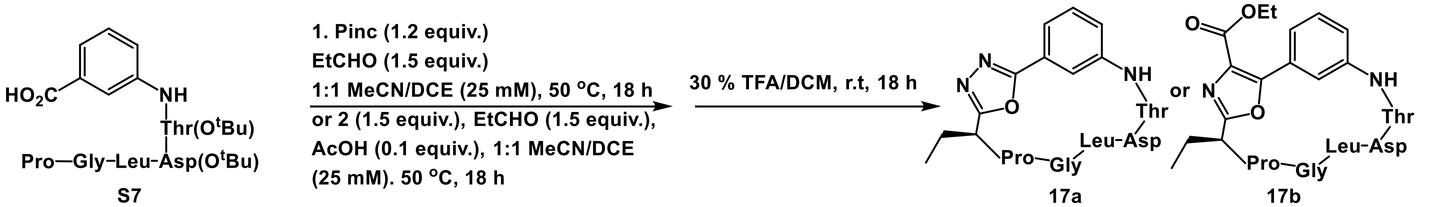

Pinc: A suspension of linear peptide in 1:1 DCE/MeCN (25 mM) at r.t was treated with EtCHO (1.5 equiv.). After 10 min this mixture was treated with Pinc (1.2 equiv.). The dark orange reaction mixture was then heated to 50 ^{o}C and stirred for 18 h. After 18 h the reaction mixture was evaporated, and the crude peptide was taken up in DCM (0.7 mL) at 0 ^{o}C and then was treated dropwise with TFA (0.3 mL). The resulting solution was stirred at r.t for 18 h. After 18 h the reaction was concentrated *in vacuo* and the residue was azeotroped with heptane (3x) and chloroform (3x). The crude peptide was purified by C18-reversed-phase chromatography and then peak fractions were pooled and lyophilized to afford **17a**. The major diastereomer was separated from the minor.

*Reagent **2**:* A suspension of linear peptide in 1:1 DCE/MeCN (25 mM) at r.t was treated with EtCHO (1.5 equiv.) followed by catalytic AcOH (0.1 equiv.). After 10 min this mixture was treated with **2** (1.5 equiv.). The dark orange reaction mixture was then heated to 50 ^{o}C and stirred for 18 h. After 18 h the reaction mixture was evaporated, and the crude peptide was taken up in DCM (0.7 mL) at 0 ^{o}C and then was treated dropwise with TFA (0.3 mL). The resulting solution was stirred at r.t for 18 h. After 18 h the reaction was concentrated *in vacuo* and the residue was azeotroped with heptane (3x) and chloroform (3x). The crude peptide was purified by C18-reversed-phase chromatography and then peak fractions were pooled and lyophilized to afford **17b**. The major diastereomer was separated from the minor.

Table 1: Full Characterization of compound **7a**

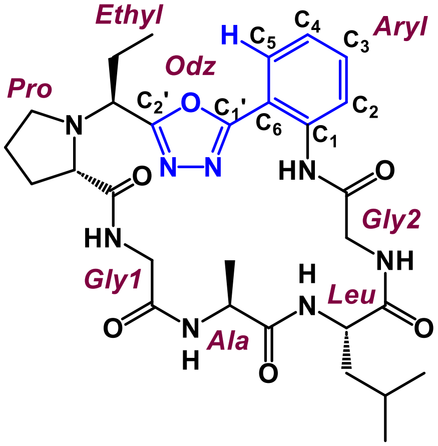

White solid obtained in a 15 % overall yield (based on 100 % resin loading).^{1}H NMR (600 MHz, DMSO-*d*6) δ 10.26 (s, 1H), 8.46 (d, *J* = 5.7 Hz, 1H), 8.11 (s, 1H), 8.07 (s, 1H), 7.94 (d, *J* = 7.8 Hz, 1H), 7.86 – 7.81 (m, 2H), 7.63 (t, *J* = 7.8 Hz, 1H), 7.38 (t, *J* = 7.5 Hz, 1H), 4.35 (q, *J* = 8.6 Hz, 1H), 4.17 – 4.12 (m, 1H), 4.02 (dd, *J* = 9.8, 5.7 Hz, 1H), 3.94 – 3.83 (m, 2H), 3.72 (d, *J* = 16.7 Hz, 2H), 3.40 (dd, *J* = 9.6, 3.3 Hz, 1H), 2.91 (d, *J* = 7.0 Hz, 1H), 2.64 (d, *J* = 7.3 Hz, 1H), 2.04 (dd, *J* = 12.6, 9.7 Hz, 1H), 1.94 – 1.86 (m, 2H), 1.76 – 1.65 (m, 5H), 1.61 - 1.59 (m, 1H), 1.30 (d, *J* = 7.2 Hz, 3H), 0.88 (d, *J* = 7.4 Hz, 3H), 0.84 (d, *J* = 7.3 Hz, 3H), 0.76 (t, *J* = 7.4 Hz, 3H). HRMS (ESI+) m/z calc. for $C_{29}H_{41}N_8O_6^+$ $[M+H]^+$ = 597.3136, found 597.3144.

	**1H Shifts**
Ethyl	CH_3 (0.76), CH_2 (1.94 – 1.86), αCH (4.02)
Gly2	αCH_2 (3.72, 3.94 – 3.83), NH (8.11)
Leu	αCH (4.35), βCH_2 (1.66 – 1.65), γCH (1.61 – 1.59), CH_3s (0.88, 0.84) NH (7.86 - 7.81)
Ala	αCH (4.17 – 4.12), CH_3 (1.30), NH (8.46)
Gly1	αCH_2 (3.72, 3.85), NH (8.07)
Pro	αCH (3.40), βCH_2 (2.04, 1.72), γCH_2 (1.71), δCH_2 (2.91, 2.64)
Aryl	NH (10.26), C_2H (7.84), C_3H (7.63), C_4H (7.38), C_5H (7.94)
Odz	N/A

	**C13 Shifts**
Ethyl	CH_3 (19.5), CH_2 (24.7), αCH (59.2)
Gly2	αC (42.7), C=O (172.6)
Leu	αC (51.2), βC (39.1), γC (24.1), CH_3s (22.6, 20.7), C=O (172.4)
Ala	αC (50.0), CH_3 (34.7), C=O (169.6)
Gly1	αC (41.9), C=O (174.1)
Pro	αC (63.0), βC (29.9), γC (23.1), δC (50.3), C=O (n.d)
Aryl	C_1 (n.d), C_2 (124.3), C_3 (132.2), C_4 (124.9), C_5 (129.3), C_6 (135.8). C=O (168.2)
Odz	C_1' (164.2), C_2' (165.7)

Figure S13:1H-NMR **7a** (600 MHz, DMSO-*d*6)

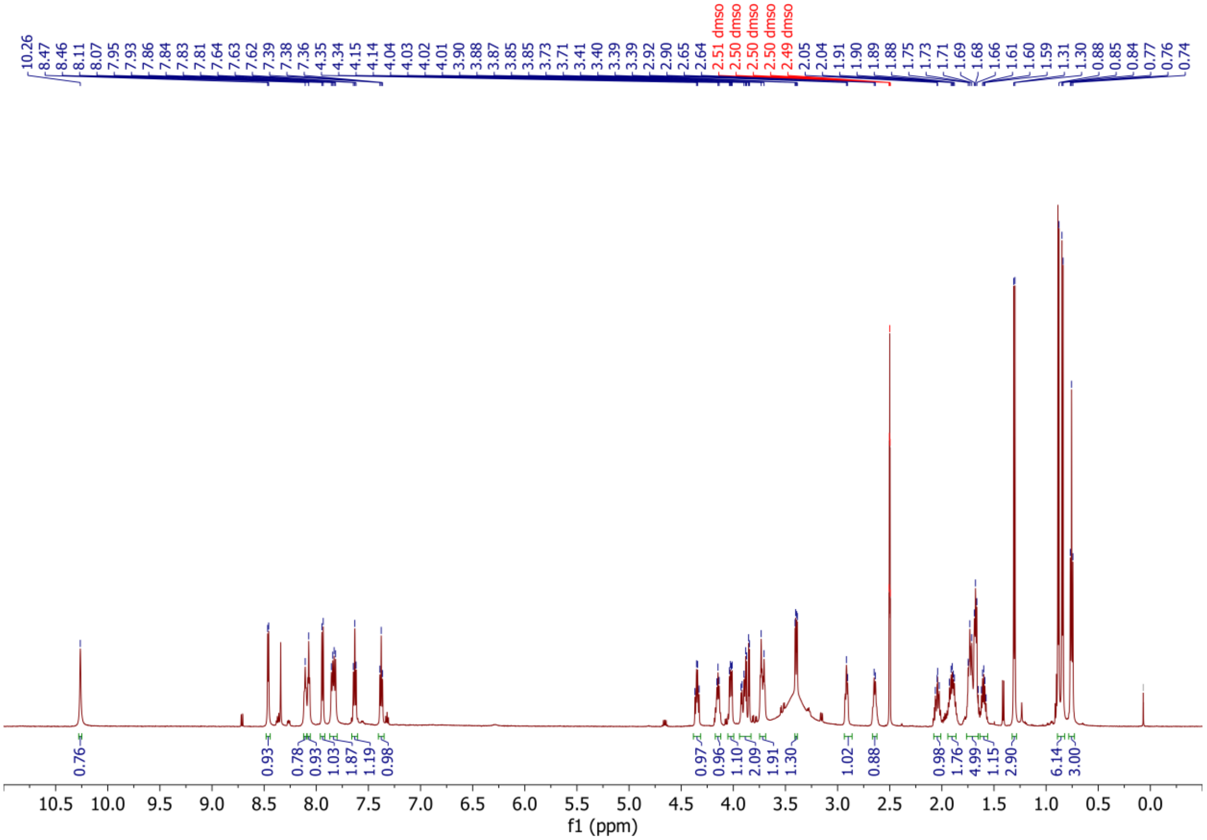

Figure S14: 2D-COSY Compound **7a** (600 MHz, DMSO-*d*6)

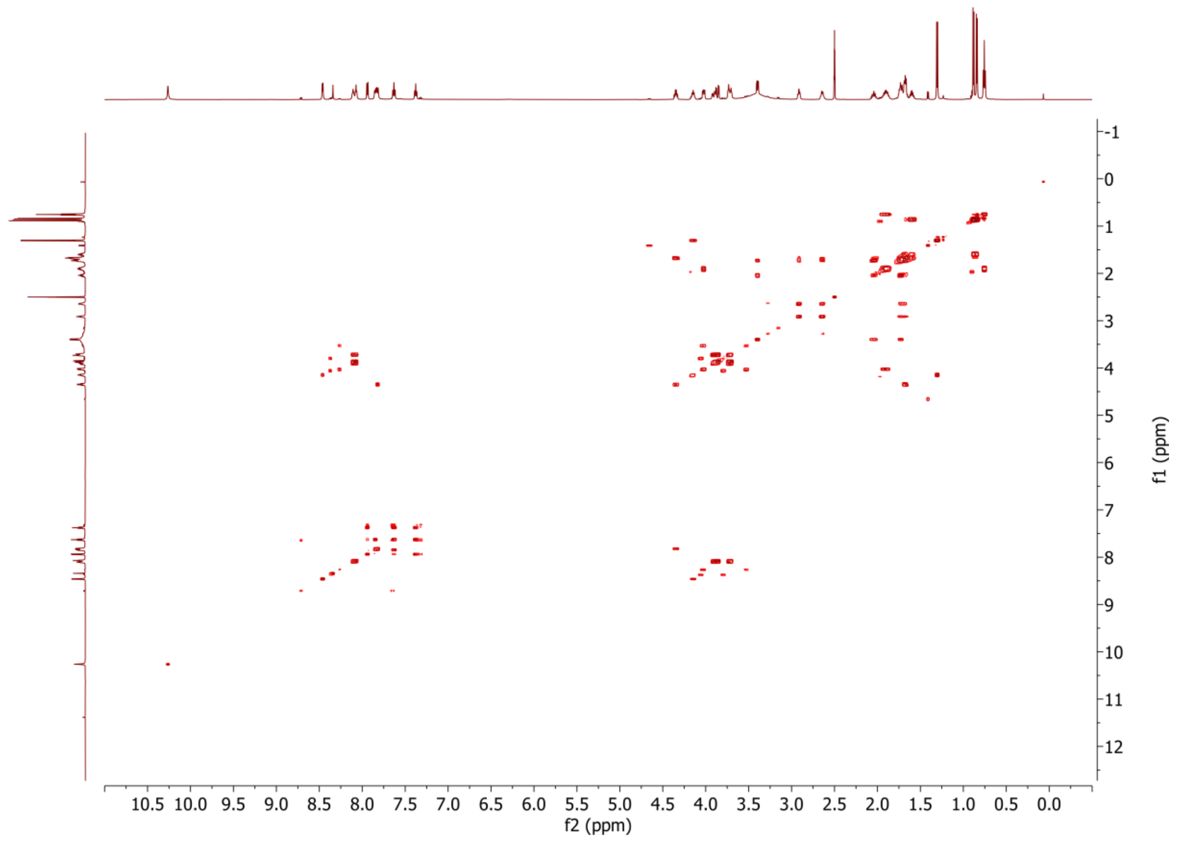

Figure S15: 2D-TOCSY Compound **7a** (600 MHz, DMSO-*d*6)

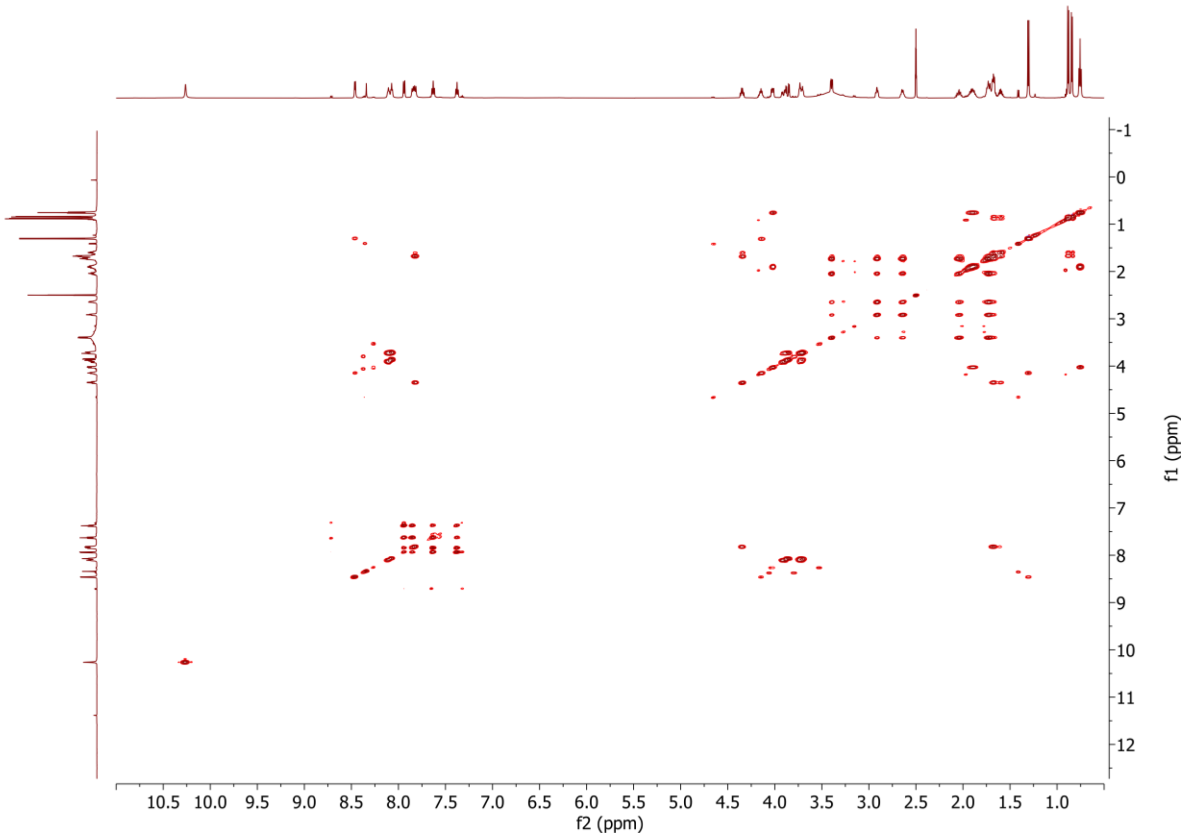

Figure S16: 2D-ROESY Compound **7a** (600 MHz, DMSO-*d*6)

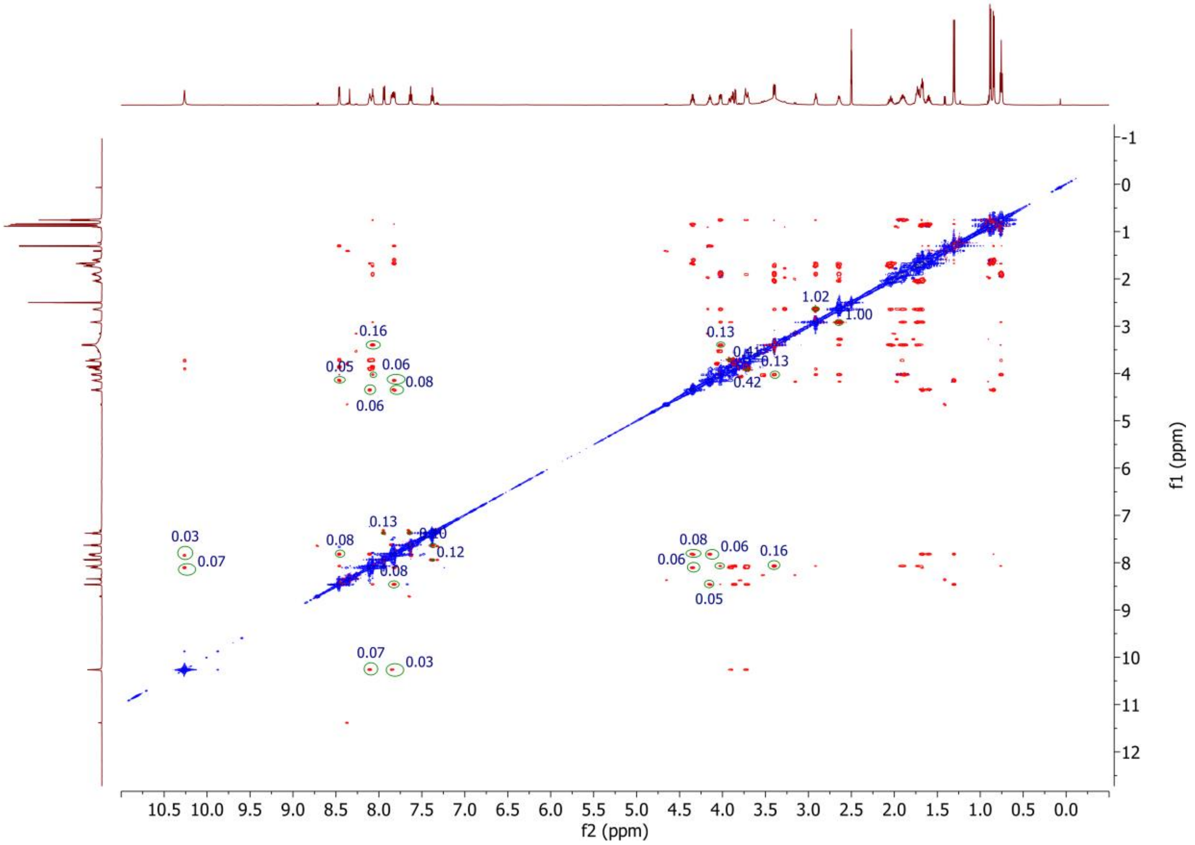

Figure S17: 2D-HSQC Compound **7a** (600 MHz, DMSO-*d6*)

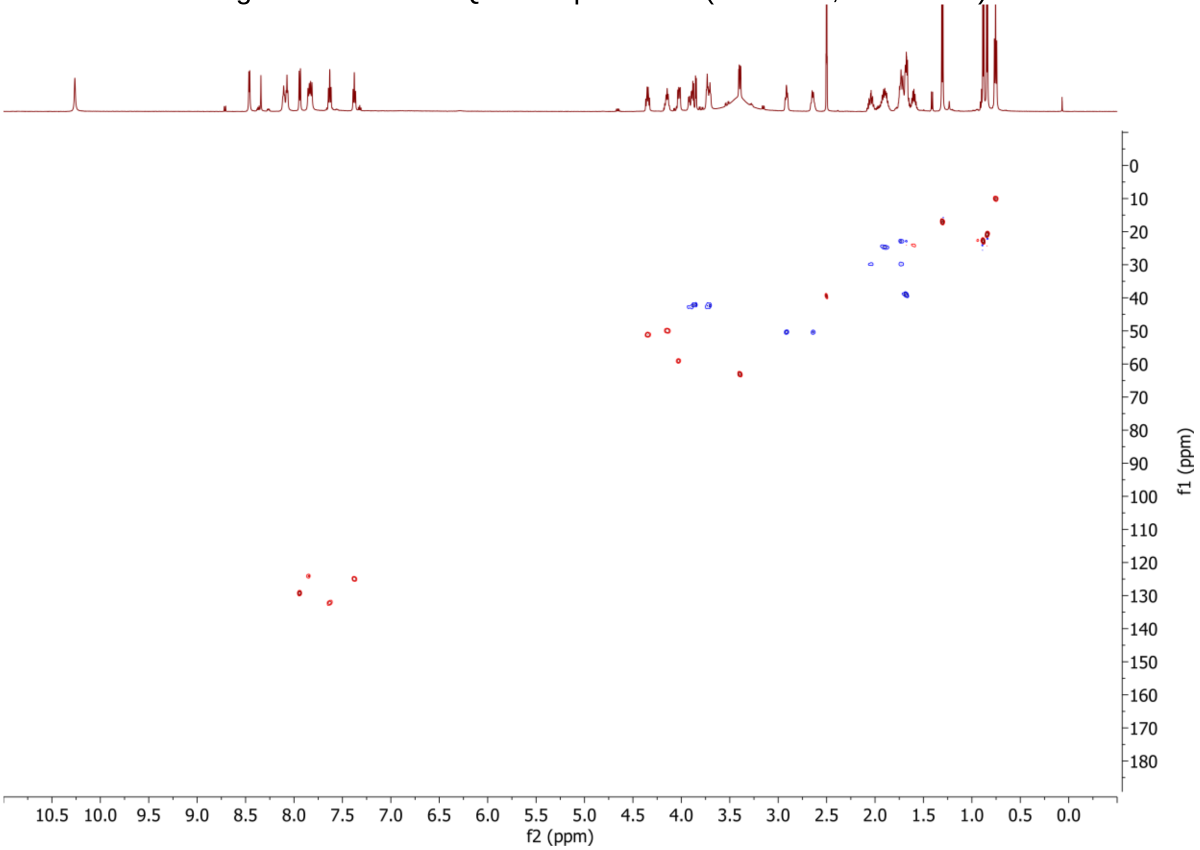

Figure S18: 2D-HMBC Compound **7a** (600 MHz, DMSO-*d6*)

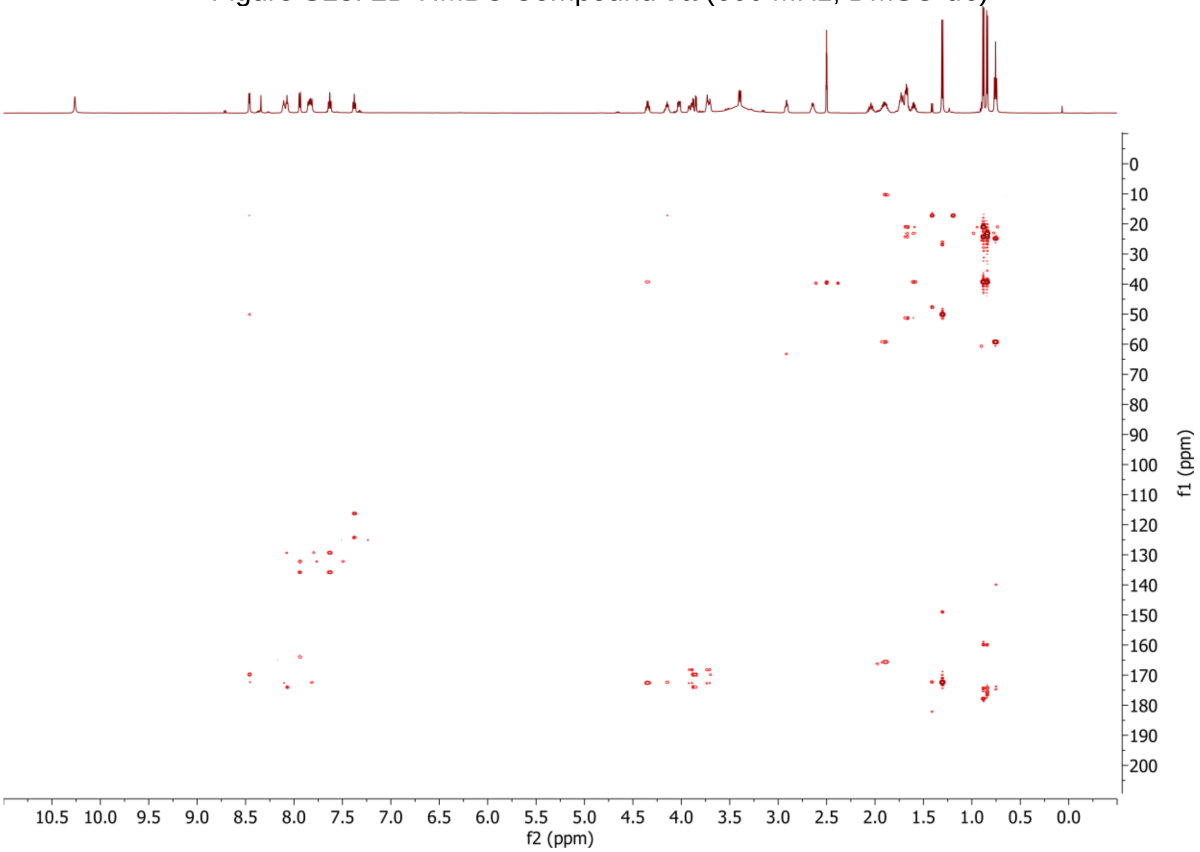

Figure S19: VT 1H-NMR Compound **7a** (600 MHz, DMSO-*d*6)

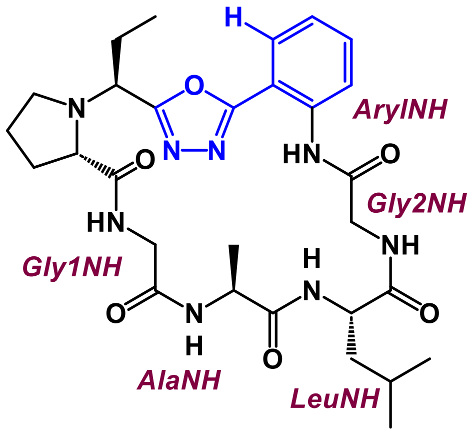

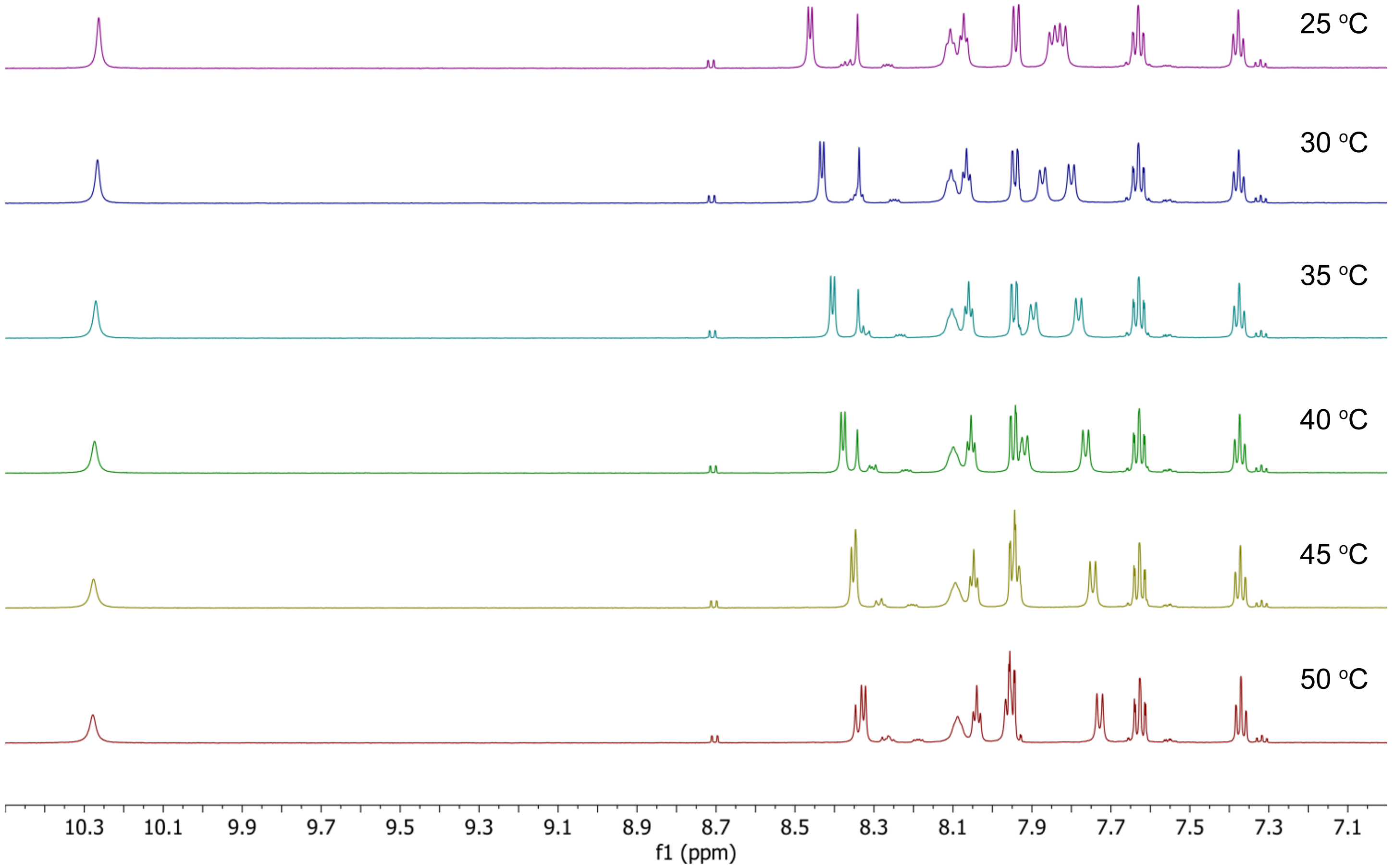

	Shifts (ppm)				
K	*ArylNH*	*Gly2NH*	*LeuNH*	*AlaNH*	*Gly1NH*
**298**	10.26	8.10	7.81	8.46	8.07
**323**	10.28	8.09	7.71	8.33	8.04
Δppb/k					
**298-323**	**0.8**	**0.4**	**4**	**5.2**	**1.2**

Table 2: Full Characterization of compound **8a**

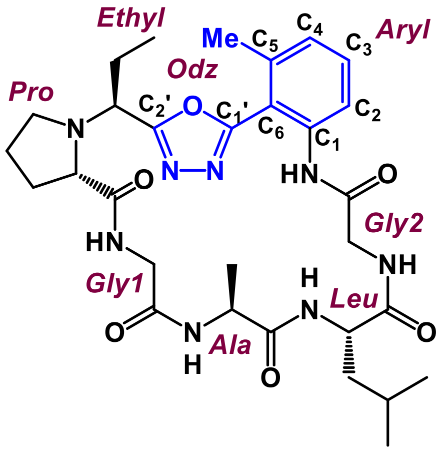

White solid obtained in an 18 % overall yield (based on 100 % resin loading). ^{1}H NMR (600 MHz, DMSO-*d*6) δ 9.74 (s, 1H), 8.43 (d, J = 5.1 Hz, 1H), 8.02 – 7.98 (m, 1H), 7.90 – 7.84 (m, 2H), 7.44 (t, J = 7.9 Hz, 1H), 7.34 (d, J = 8.1 Hz, 1H), 7.23 (d, J = 7.6 Hz, 1H), 4.30 (td, J = 10.1, 4.0 Hz, 1H), 3.99 (p, J = 5.4 Hz, 1H), 3.92 – 3.86 (m, 2H), 3.84 – 3.80 (m, 2H), 3.57 (dd, J = 16.7, 5.6 Hz, 1H), 3.39 – 3.33 (m, 1H), 2.83 - 2.79 (m, 1H), 2.60 – 2.56 (m, 1H), 2.13 – 2.04 (m, 1H), 1.99 – 1.93 (m, 1H), 1.88 – 1.83 (m, 1H), 1.78 – 1.70 (m, 3H), 1.68 – 1.64 (m, 2H), 1.63 – 1.59 (m, 1H), 1.29 (d, J = 7.3 Hz, 3H), 0.90 (d, J = 6.4 Hz, 3H), 0.84 (d, J = 6.4 Hz, 3H), 0.79 (t, J = 7.4 Hz, 3H). HRMS (ESI+) m/z calc. for $C_{30}H_{43}N_8O_6^+$ [M+H]$^+$ = 611.3299, found 611.3300.

	**1H Shifts**
Ethyl	CH_3 (0.79), CH_2 (1.68 – 1.64), αCH (3.92 – 3.86)
Gly2	αCH_2 (3.92 – 3.86, 3.57), NH (7.90 – 7.84)
Leu	αCH (4.30), βCH_2 (1.78 – 1.70), γCH (1.88 – 1.83), CH_3s (0.90, 0.84) NH (7.90 – 7.84)
Ala	αCH (3.99), CH_3 (1.29), NH (8.43)
Gly1	αCH_2 (3.84 – 3.80), NH (8.02 – 7.98)
Pro	αCH (3.35), βCH_2 (2.83 - 2.79, 2.60 – 2.56), γCH_2 (1.78 – 1.70, 1.63 – 1.59), δCH_2 (2.13 – 2.04, 1.99 – 1.93)
Aryl	NH (9.74), C_4H (7.23), C_3H (7.44), C_2H (7.34), CH_3 (2.40)
Odz	N/A

Figure S20:1H-NMR **8a** (500 MHz, DMSO-*d6*)

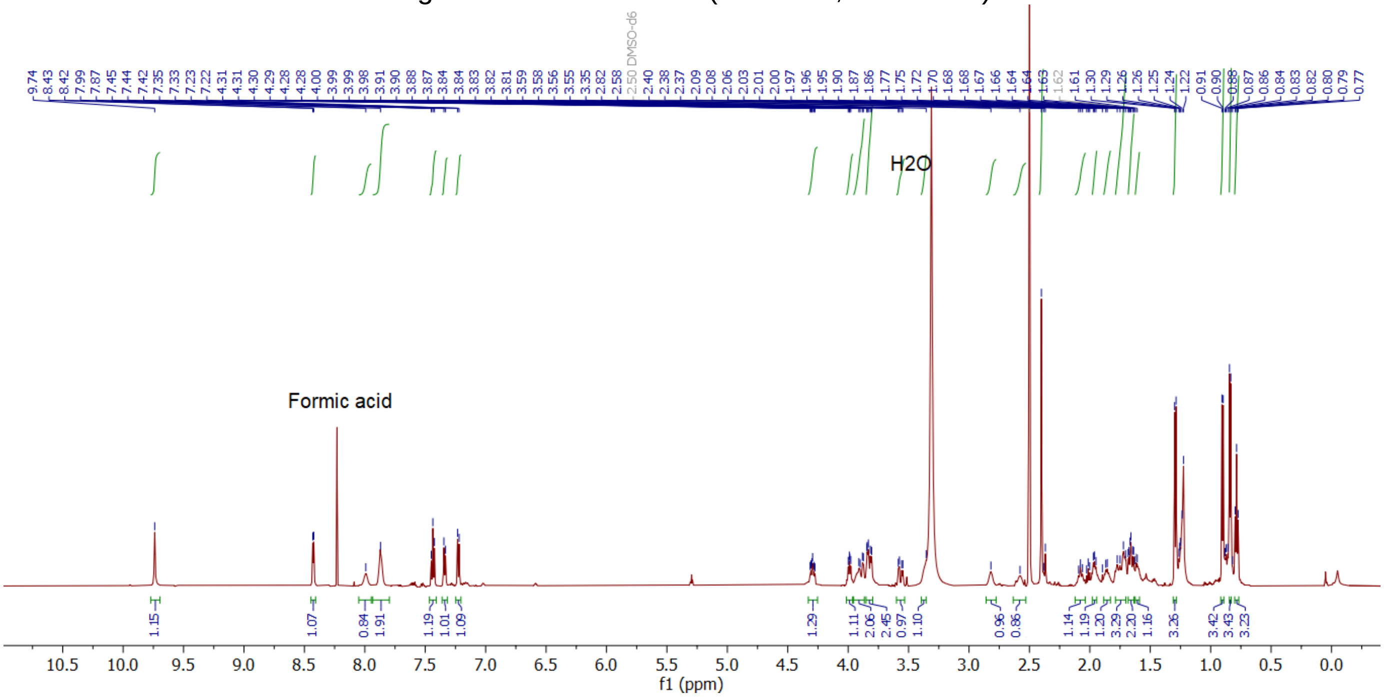

Figure S21: 2D-COSY Compound **8a** (500 MHz, DMSO-*d6*)

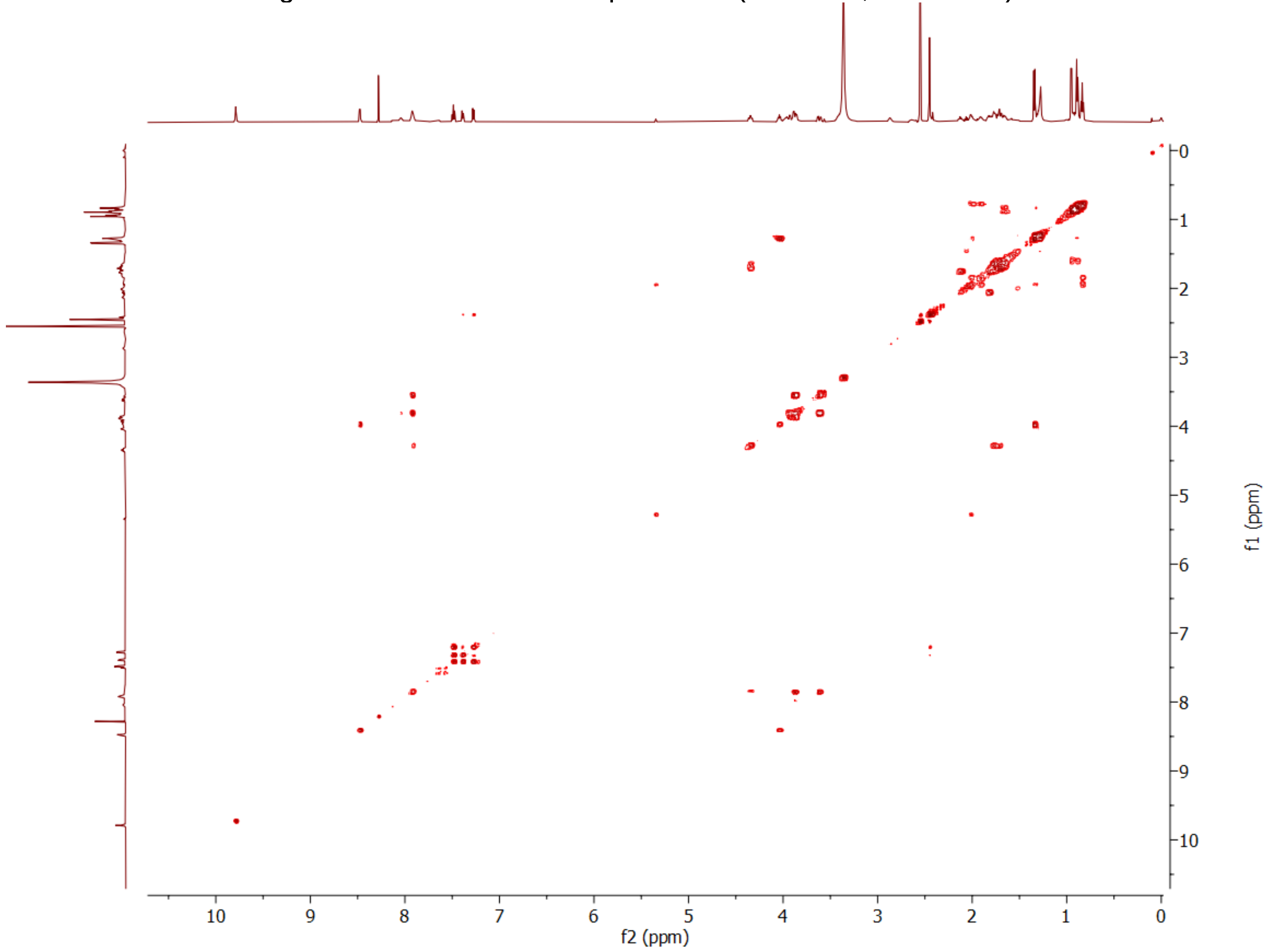

Figure S22: 2D-TOCSY Compound **8a** (500 MHz, DMSO-*d*6)

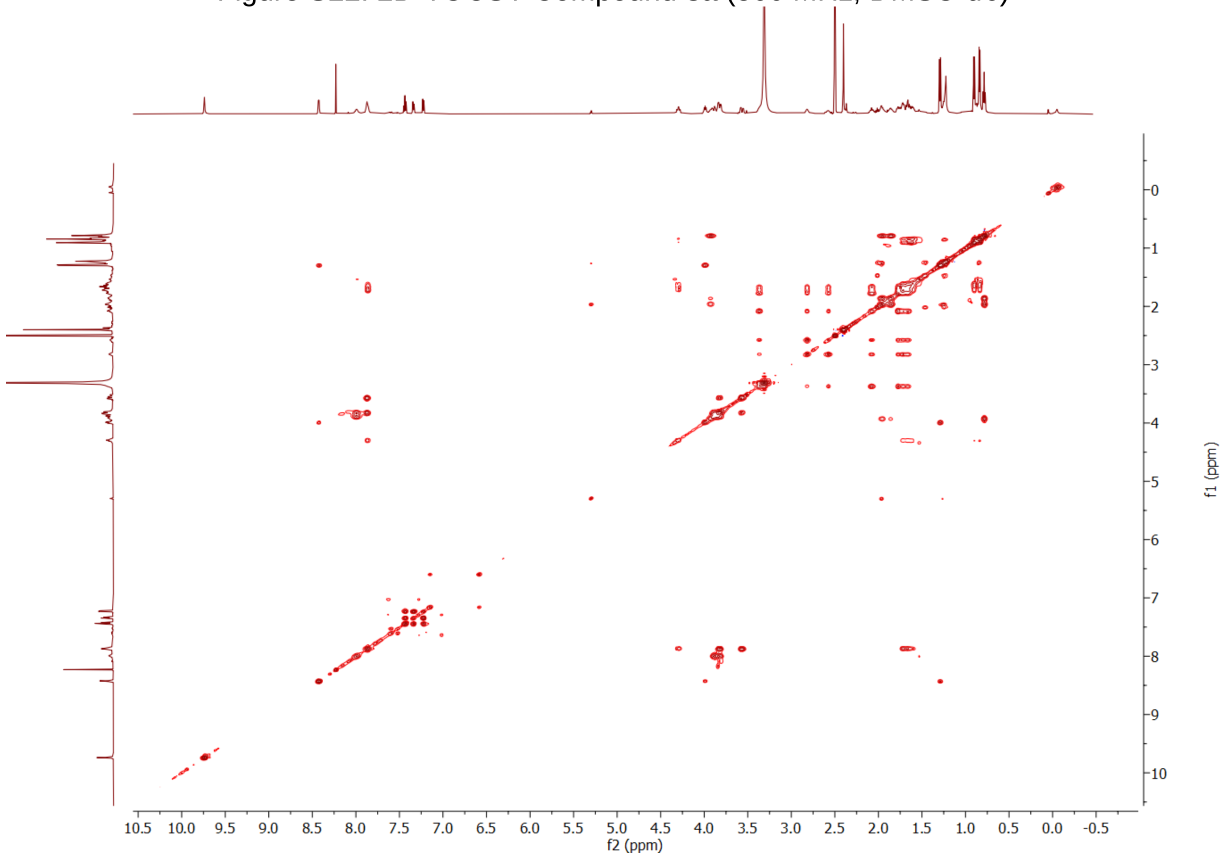

Figure S23: 2D-ROESY Compound **8a** (500 MHz, DMSO-*d*6)

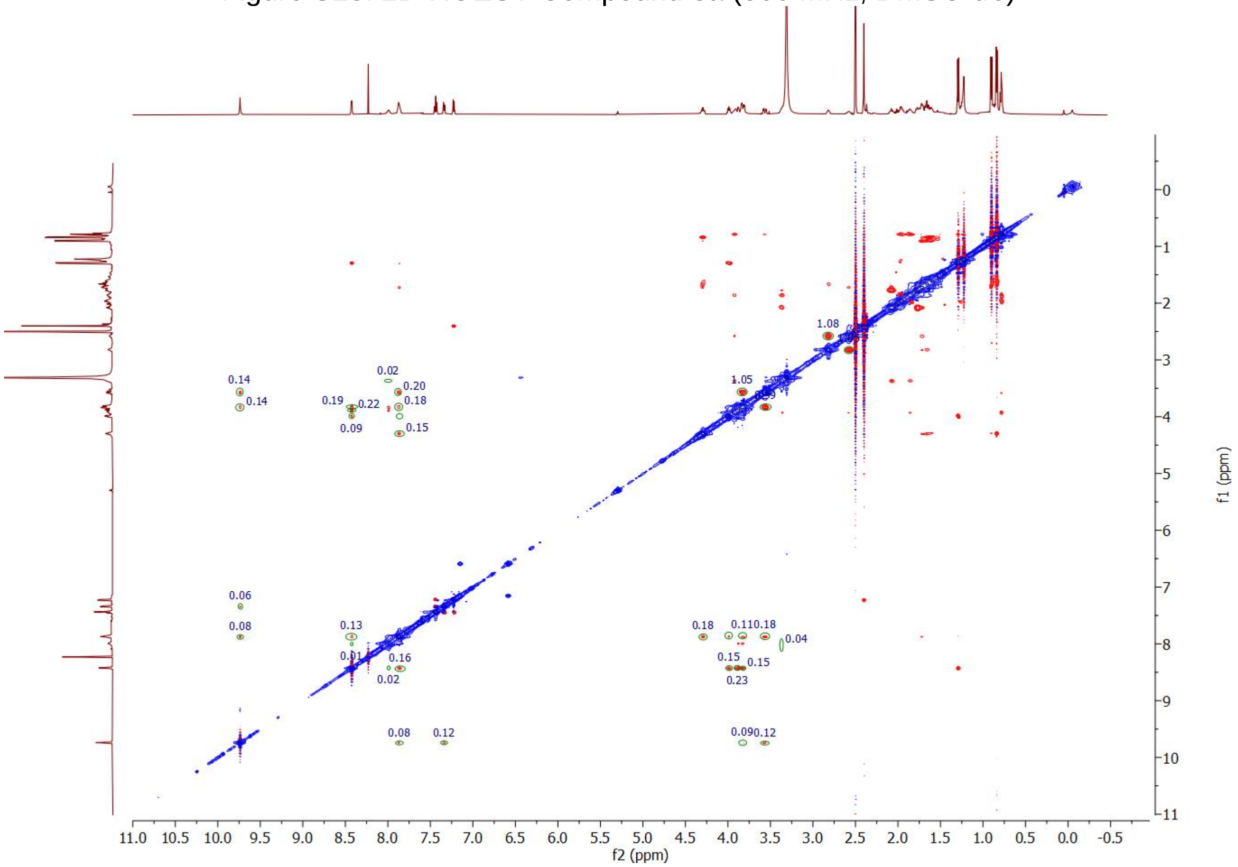

Figure S24: 2D-HSQC Compound **8a** (500 MHz, DMSO-*d*6)

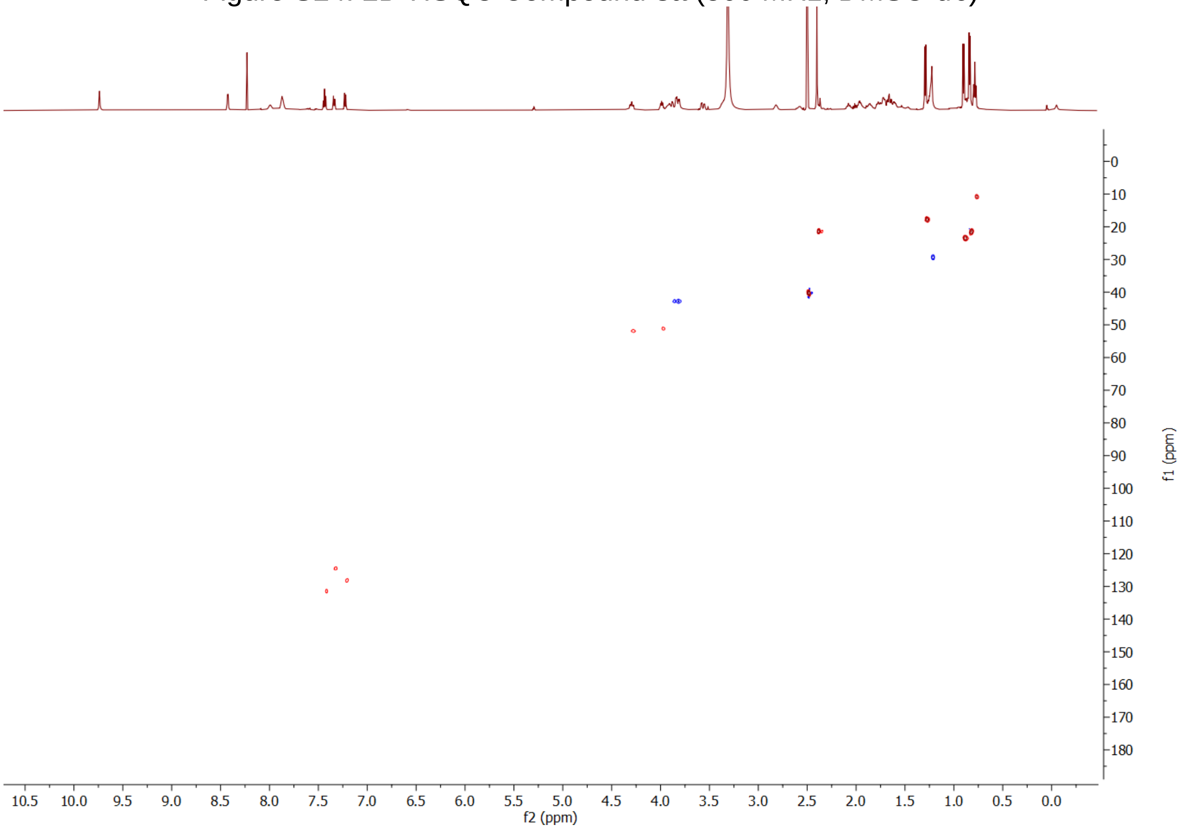

Figure S25: 2D-HMBC Compound **8a** (500 MHz, DMSO-*d*6)

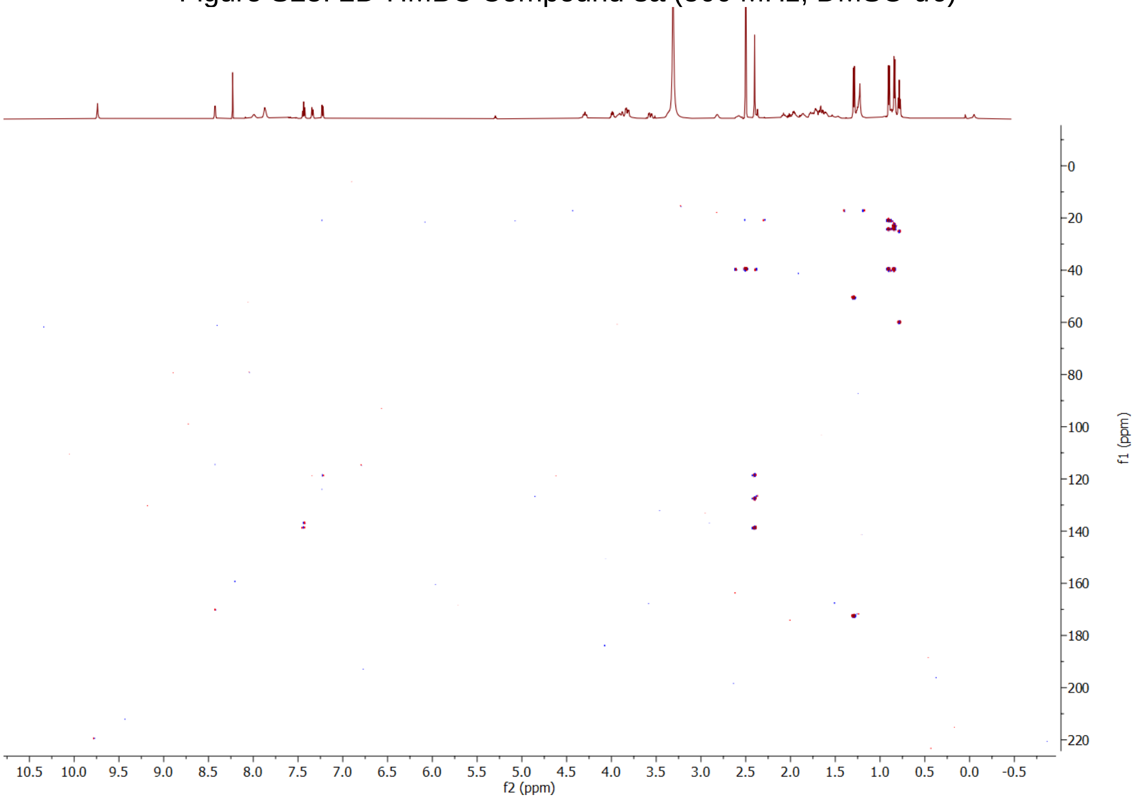

Figure S26: VT 1H-NMR Compound **8a** (500 MHz, DMSO-*d*6)

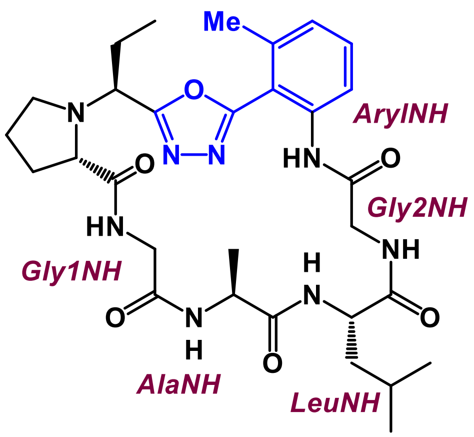

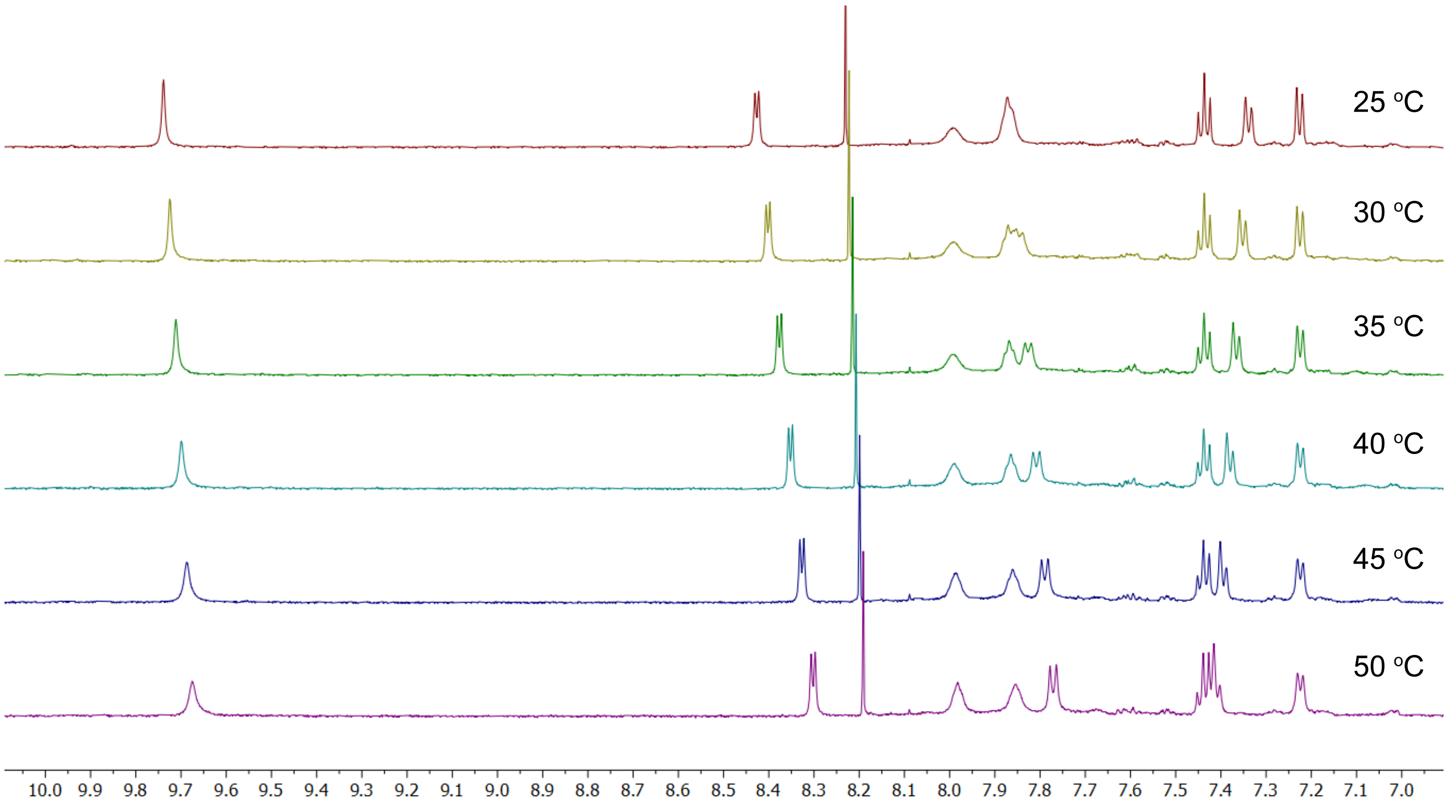

	Shifts (ppm)				
K	*ArylNH*	*Gly2NH*	*LeuNH*	*AlaNH*	*Gly1NH*
**298**	9.74	7.87	7.87	8.42	7.99
**323**	9.67	7.85	7.77	8.30	7.98
Δppb/k					
**298-323**	**2.8**	**0.8**	**4**	**4.8**	**0.4**

Table 3: Full Characterization of compound **11a**

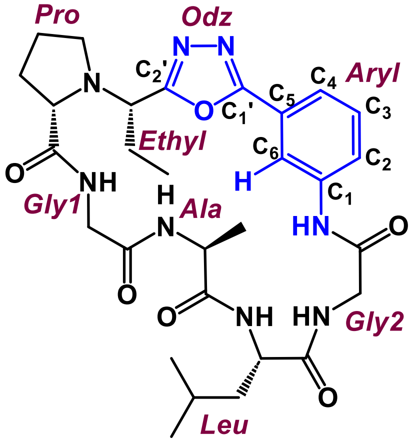

White solid obtained in a 38 % overall yield (based on 100 % resin loading). ^{1}H NMR (600 MHz, DMSO-*d6*) δ 9.73 (s, 1H), 8.45 – 8.39 (m, 2H), 8.28 (dd, *J* = 7.3, 4.8 Hz, 1H), 8.23 (t, *J* = 1.9 Hz, 1H), 7.96 (dt, *J* = 8.1, 1.6 Hz, 1H), 7.86 (dt, *J* = 7.8, 1.4 Hz, 1H), 7.56 (t, *J* = 8.0 Hz, 1H), 7.52 (d, *J* = 8.0 Hz, 1H), 4.46 (p, *J* = 7.2 Hz, 1H), 4.14 (dt, *J* = 7.9, 6.5 Hz, 1H), 4.08 – 4.01 (m, 2H), 3.92 (dd, *J* = 9.7, 5.9 Hz, 1H), 3.74 (dd, *J* = 17.1, 4.8 Hz, 1H), 3.67 (dd, *J* = 16.4, 4.7 Hz, 1H), 3.07 (dd, *J* = 8.5, 5.5 Hz, 1H), 2.95 (td, *J* = 8.0, 2.9 Hz, 1H), 2.68 (q, *J* = 8.1 Hz, 1H), 2.03 (ddq, *J* = 14.6, 9.5, 7.2 Hz, 1H), 1.98 – 1.86 (m, 1H), 1.73 – 1.66 (m, 3H), 1.59 (dq, *J* = 8.1, 6.0 Hz, 1H), 1.57 – 1.50 (m, 2H), 1.28 (d, *J* = 7.0 Hz, 3H), 0.92 (d, *J* = 6.4 Hz, 3H), 0.86 (d, *J* = 6.3 Hz, 3H), 0.80 (t, *J* = 7.3 Hz, 3H). HRMS (ESI+) m/z calc. for $C_{29}H_{41}N_8O_6^+$ $[M+H]^+$ = 597.3138, found 597.3144.

	**1H Shifts**
Ethyl	CH_3 (0.81), CH_2 (2.03, 1.90), αCH (3.92)
Gly2	αCH_2 (3.74, 4.08 – 4.01), NH (8.45 – 8.39)
Leu	αCH (4.14), βCH_2 (1.57 – 1.50), γCH (1.59), CH_3s (0.92, 0.86) NH (8.45 – 8.39)
Ala	αCH (4.46), CH_3 (1.28), NH (7.52)
Gly1	αCH_2 (3.67, 4.08 – 4.01), NH (8.28)
Pro	αCH (3.07), βCH_2 (1.73 – 1.66), γCH_2 (1.98 – 1.96, 1.73 – 1.66), δCH_2 (2.68, 2.95)
Aryl	NH (9.73), C_2H (7.86), C_3H (7.56), C_4H (7.96), C_6H (8.23)
Odz	N/A

	**C13 Shifts**
Ethyl	CH_3 (11.3), CH2 (25.7), αCH (57.4)
Gly2	αC (42.7), C=O (168.0)
Leu	αC (52.2), βC (39.5), γC (24.1), CH_3s (22.4), C=O (172.2)
Ala	αC (47.9), CH_3 (17.8), C=O (173.0)
Gly1	αC (42.3), C=O (168.7)
Pro	αC (63.7), βC (29.3), γC (29.3), δC (47.4), C=O (173.2)
Aryl	C_1 (139.1), C_2 (121.5), C_3 (129.7), C_4 (122.2), C_5 (123.6), C_6 (117.1). C=O (168.1)
Odz	C_1' (163.6), C_2' (165.2)

Figure S27:1H-NMR **11a** (500 MHz, DMSO-*d*6)

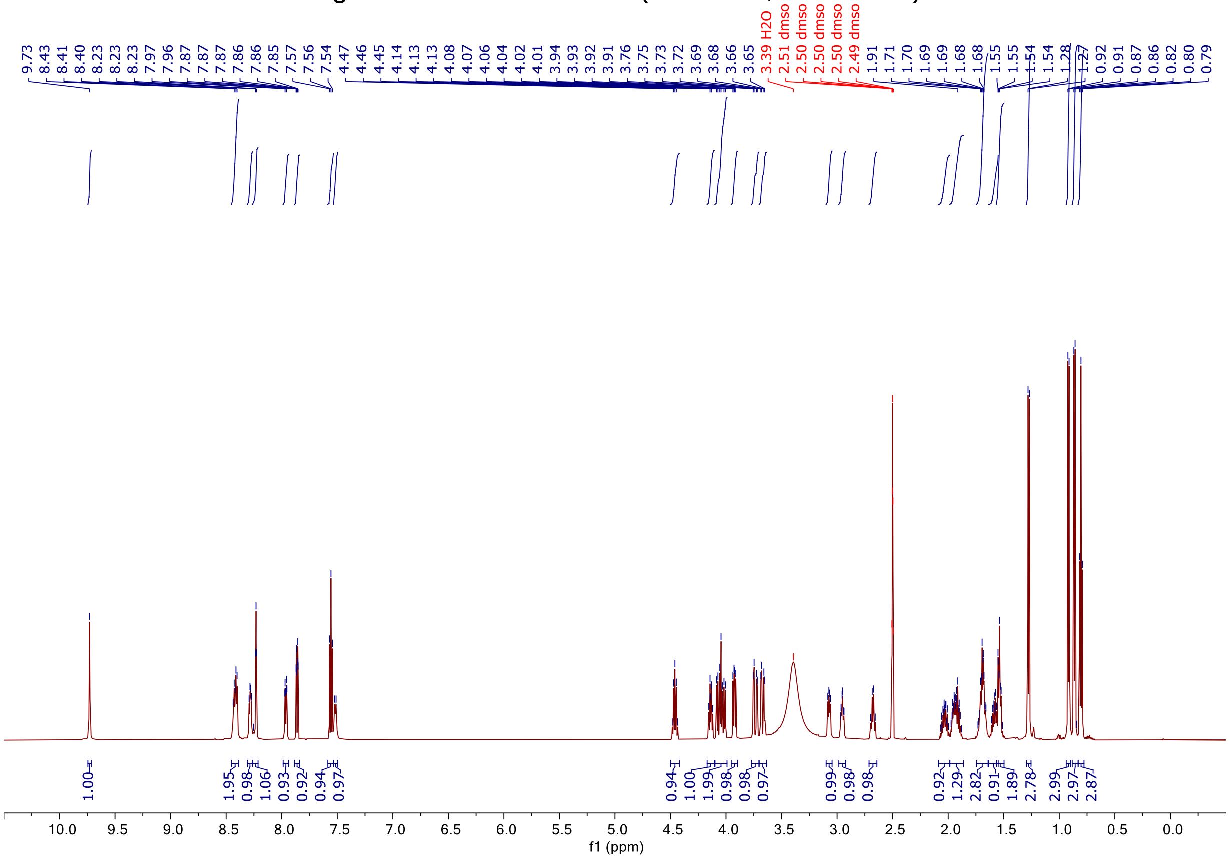

Figure S28: 2D-COSY Compound **11a** (500 MHz, DMSO-*d*6)

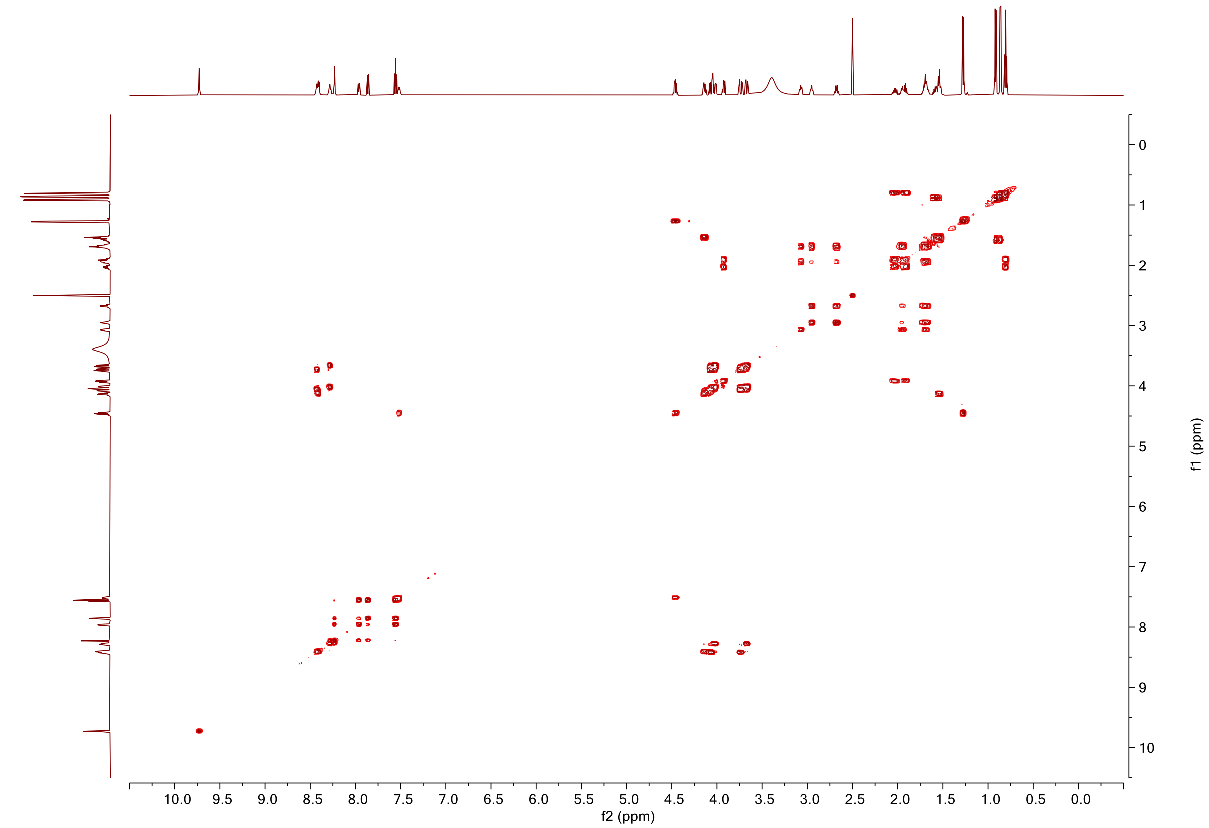

Figure S29: 2D-TOCSY Compound **11a** (500 MHz, DMSO-*d6*)

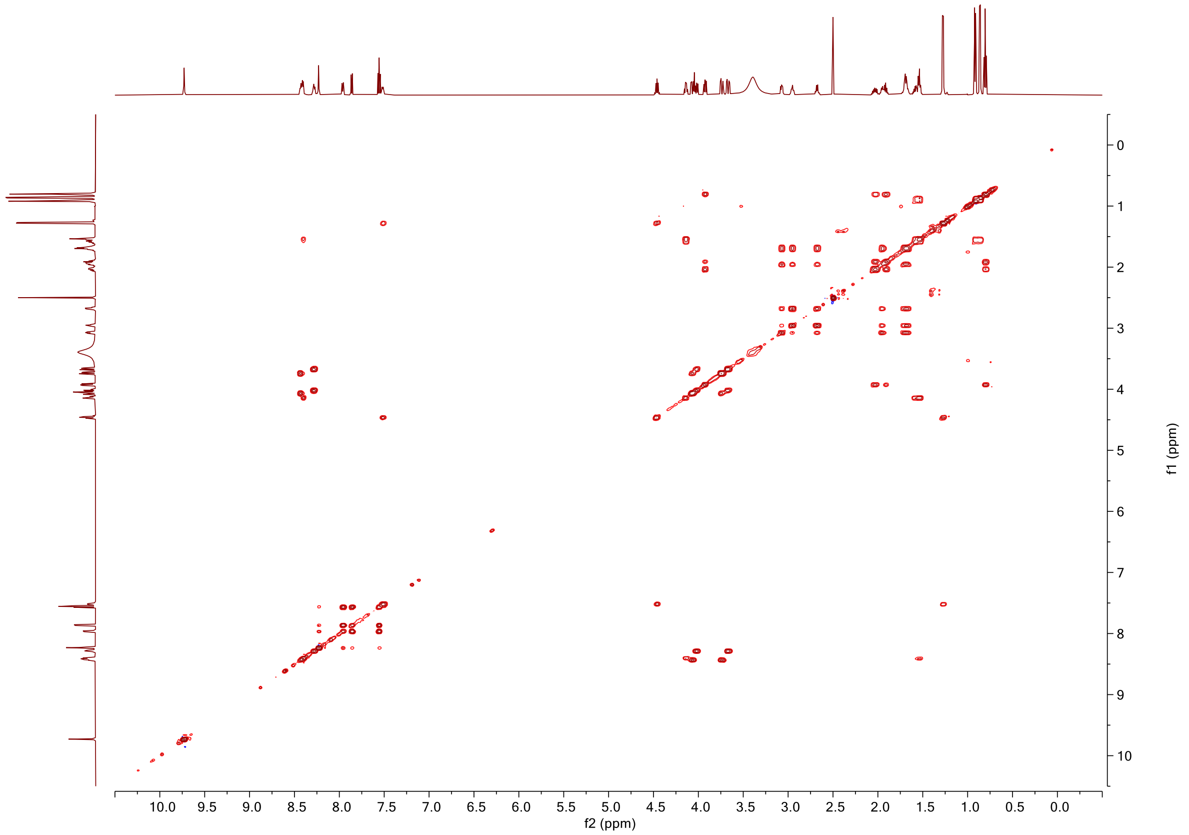

Figure S30: 2D-ROESY Compound **11a** (500 MHz, DMSO-*d6*)

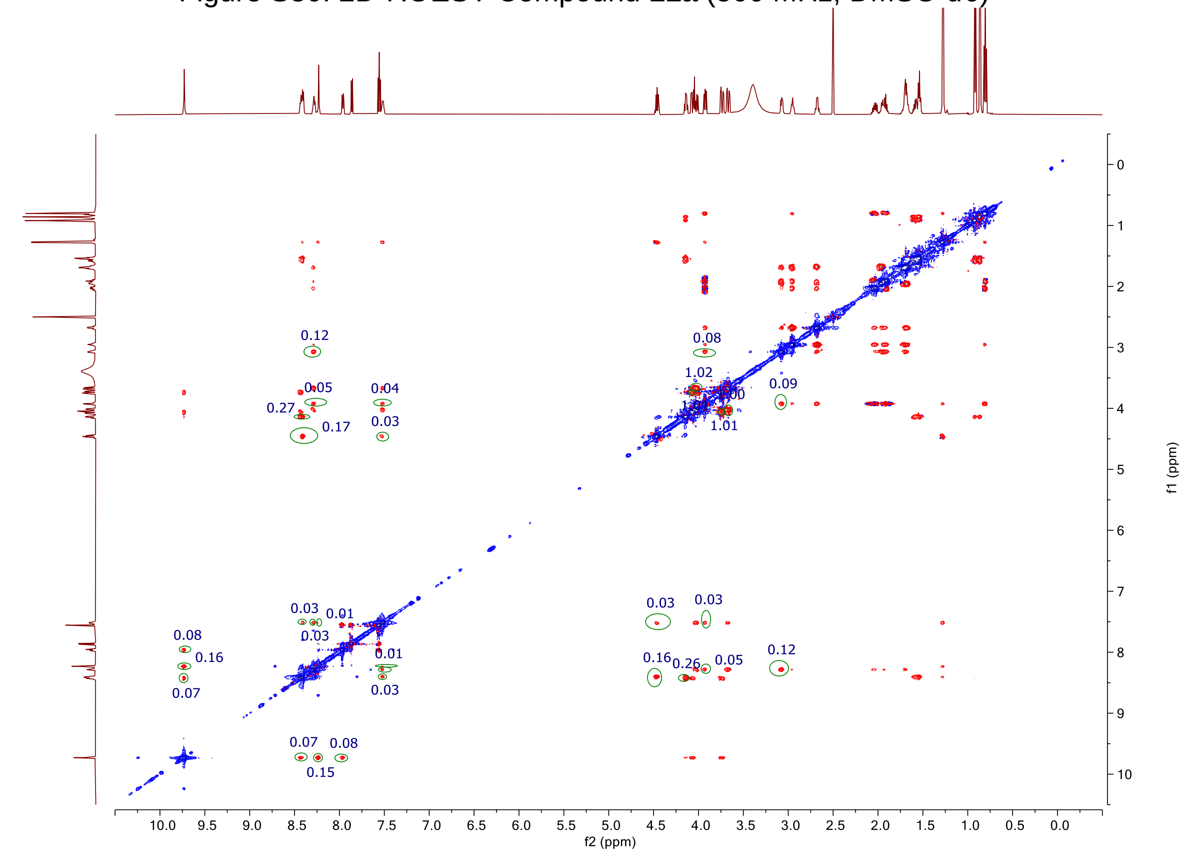

Figure S31: 2D-HSQC Compound **11a** (500 MHz, DMSO-*d*6)

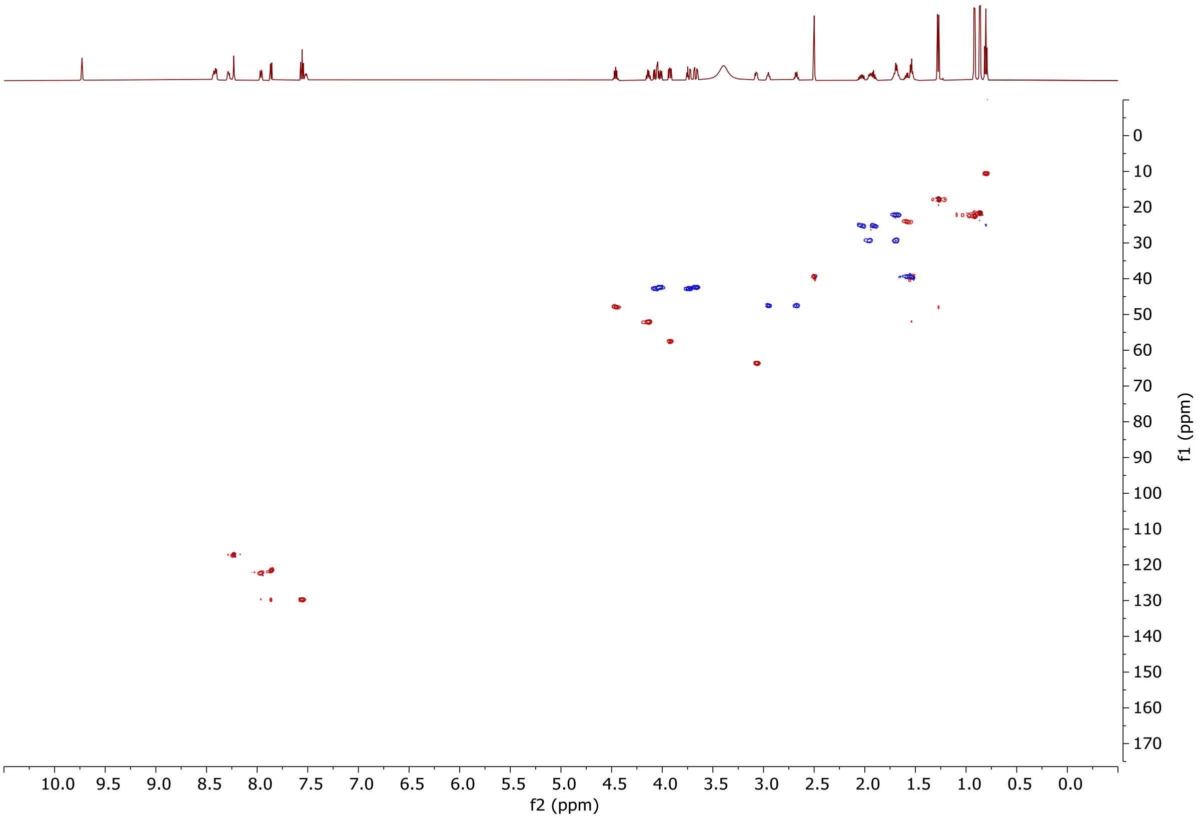

Figure S32: 2D-HMBC Compound **11a** (500 MHz, DMSO-*d*6)

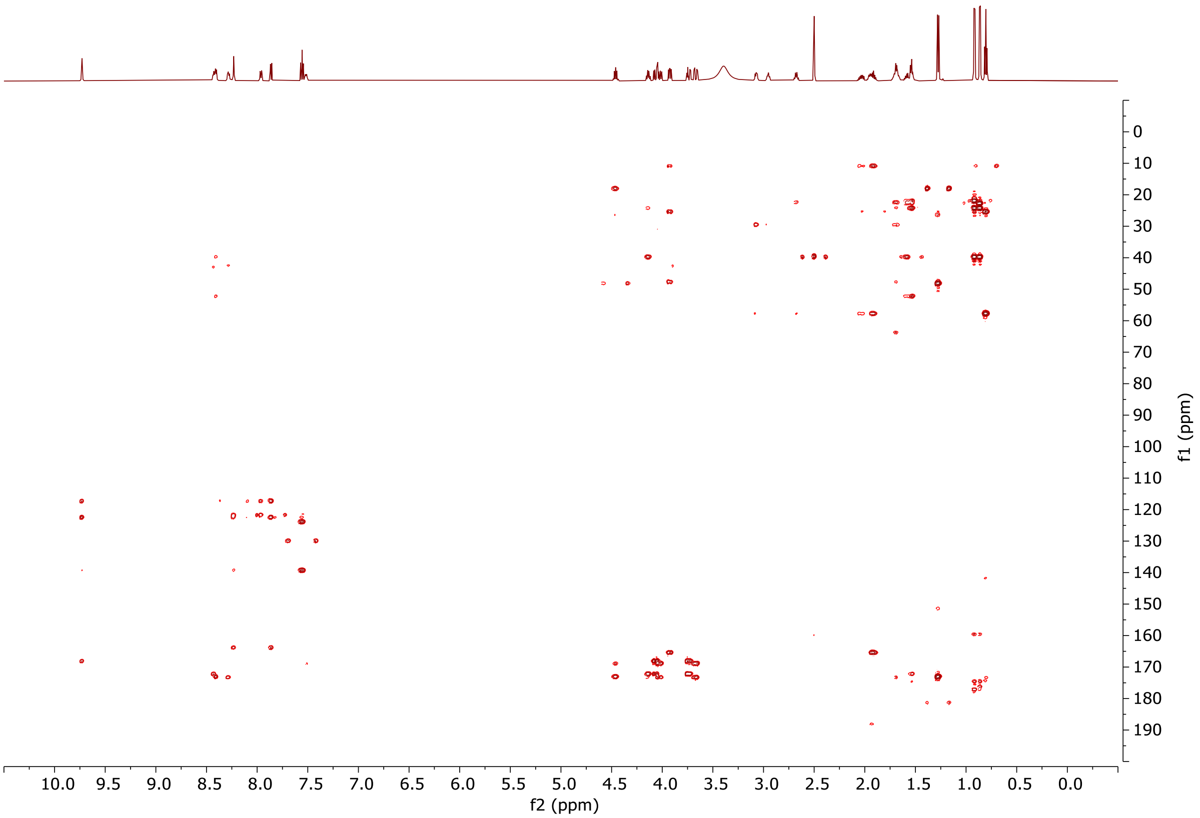

Figure S33: VT 1H-NMR Compound **11a** (500 MHz, DMSO-*d*6)

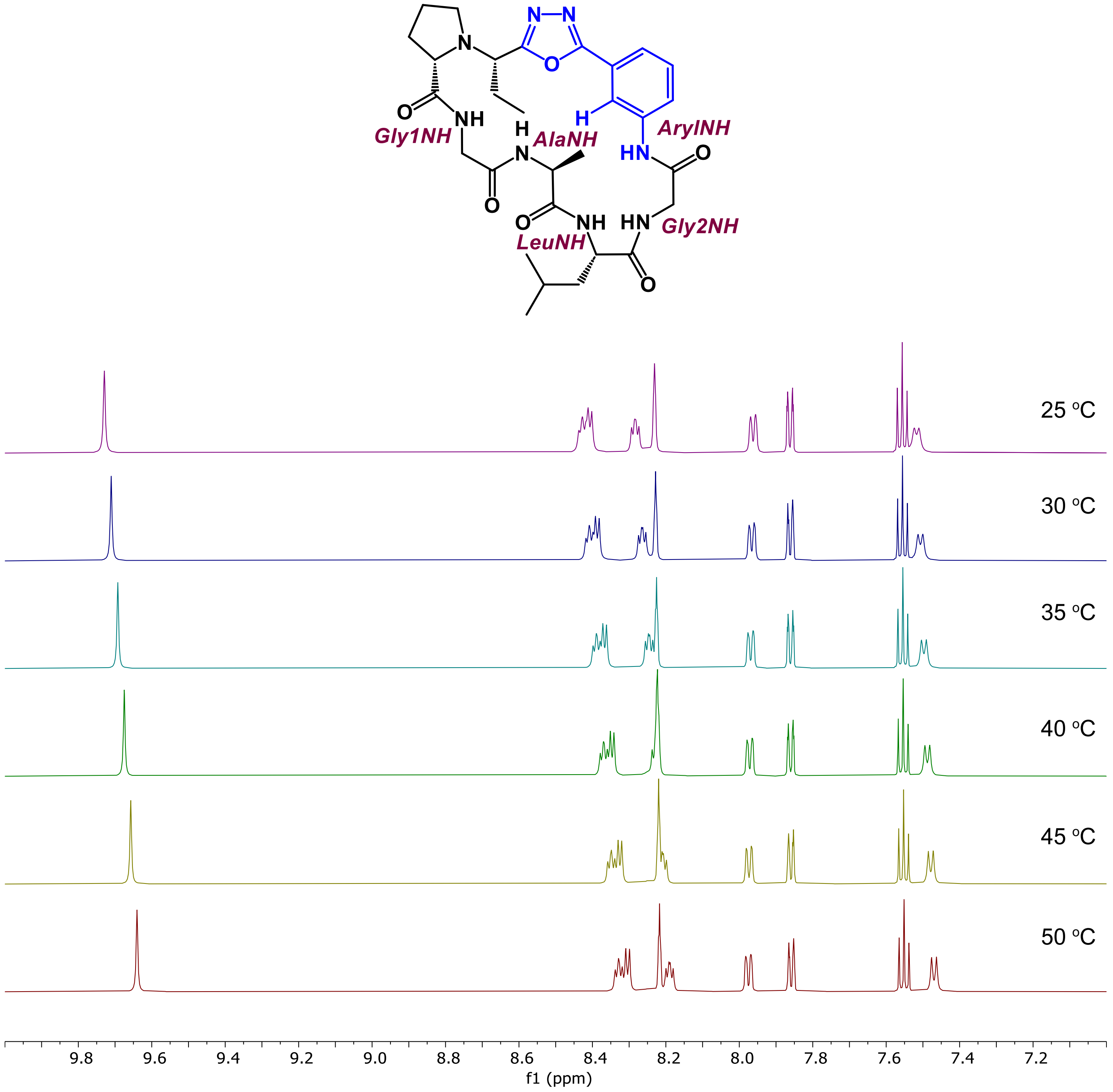

	Shifts (ppm)				
K	*ArylNH*	*Gly2NH*	*LeuNH*	*AlaNH*	*Gly1NH*
**298**	9.73	8.43	8.41	7.52	8.28
**323**	9.64	8.33	8.30	7.47	8.19
Δppb/k					
**298-323**	**3.6**	**4**	**4.4**	**2**	**3.6**

Table 4: Full Characterization of compound **11b**

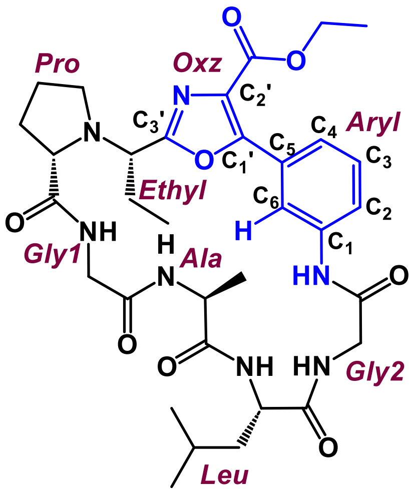

White solid obtained in 38 % overall yield (based on 100 % resin loading). ^{1}H NMR (500 MHz, DMSO-*d6*) δ 9.78 (s, 1H), 8.27 – 8.20 (m, 1H), 8.20 – 8.16 (m, 2H), 8.06 (dd, *J* = 7.2, 4.5 Hz, 1H), 7.97 (d, *J* = 7.9 Hz, 1H), 7.74 (d, *J* = 7.1 Hz, 1H), 7.64 (d, *J* = 8.2 Hz, 1H), 7.46 (t, *J* = 8.0 Hz, 1H), 4.35 – 4.26 (m, 3H), 4.21 – 4.02 (m, 3H), 3.81 – 3.73 (m, 2H), 3.65 (dd, *J* = 16.3, 4.3 Hz, 1H), 3.30 – 3.24 (m, 1H), 2.92 – 2.88 (m, 1H), 2.61 – 2.57 (d, *J* = 8.1 Hz, 1H), 2.02 – 1.98 (m, 2H), 1.89 – 1.82 (m, 1H), 1.72 – 1.65 (m, 2H), 1.57 - 1.53 (m, 3H), 1.31 – 1.21 (m, 6H), 0.90 (d, *J* = 5.9 Hz, 3H), 0.87 – 0.78 (m, 6H). HRMS (ESI+) m/z calc. for $C_{30}H_{43}N_8O_6^+$ $[M+H]^+$ = 611.3292, found 61.3300.

	**1H Shifts**
Ethyl	CH_3 (0.87 – 0.78), CH_2 (1.89 – 1.82, 2.02 – 1.98), αCH (3.81 – 3.73)
Gly2	αCH_2 (3.81 – 3.73, 4.21 – 4.02), NH (8.20 – 8.16)
Leu	αCH (4.21 – 4.02), βCH_2 (1.57 - 1.53), γCH (1.57 - 1.53), CH_3s (0.90, 0.87 – 0.78) NH (8.27 – 8.20)
Ala	αCH (4.35 – 4.26), CH_3 (1.31 – 1.21), NH (7.74)
Gly1	αCH_2 (3.65, 4.21 – 4.02), NH (8.06)
Pro	αCH (3.30 – 3.24), βCH_2 (1.72 – 1.65, 2.02 – 1.98), γCH_2 (1.72 – 1.65), δCH_2 (2.61 – 2.57, 2.92 – 2.88)
Aryl	NH (9.78), C_2H (7.64), C_3H (7.46), C_4H (7.97), C_6H (8.20 – 8.16)
Oxz	CH_2 (4.35 – 4.26), CH_3 (1.31 – 1.21)

	**C13 Shifts**
Ethyl	CH_3 (10.5), CH_2 (24.8), αCH (59.5)
Gly2	αC (42.8), C=O (171.2)
Leu	αC (51.6), βC (39.6), γC (24.0), CH_3s (21.2, 22.5), C=O (172.2)
Ala	αC (48.5), CH_3 (17.7), C=O (172.9)
Gly1	αC (42.2), C=O (173.4)
Pro	αC (63.7), βC (29.7), γC (22.6), δC (47.9), C=O (n.d)
Aryl	C_1 (138.5), C_2 (121.2), C_3 (128.7), C_4 (124.0), C_5 (n.d), C_6 (118.0). C=O (167.6)
Odz	C_1' (n.d), C_2' (n.d), C_3' (161.5), CH_2 (60.5), CH_3 (13.7), C=O (161.6)

Figure S34:1H-NMR **11b** (500 MHz, DMSO-*d*6)

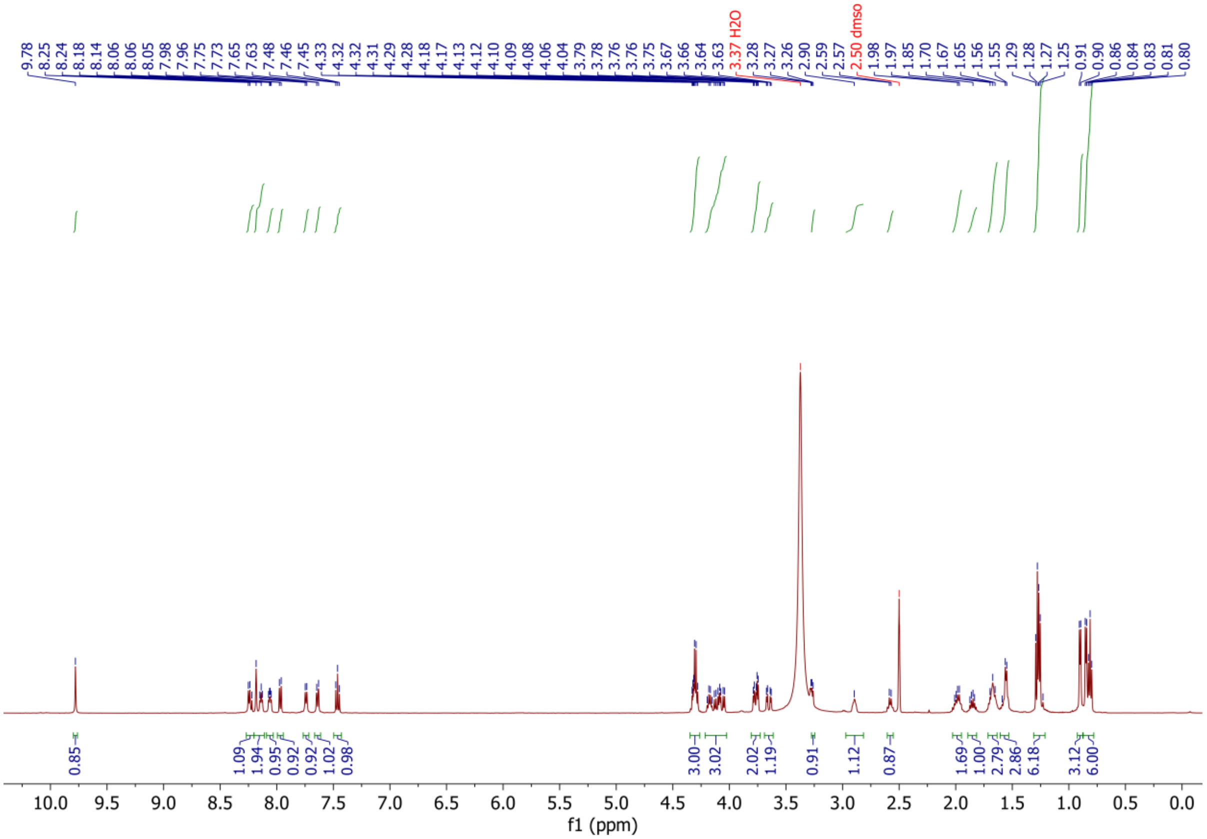

Figure S35: 2D-COSY Compound **11b** (500 MHz, DMSO-*d*6)

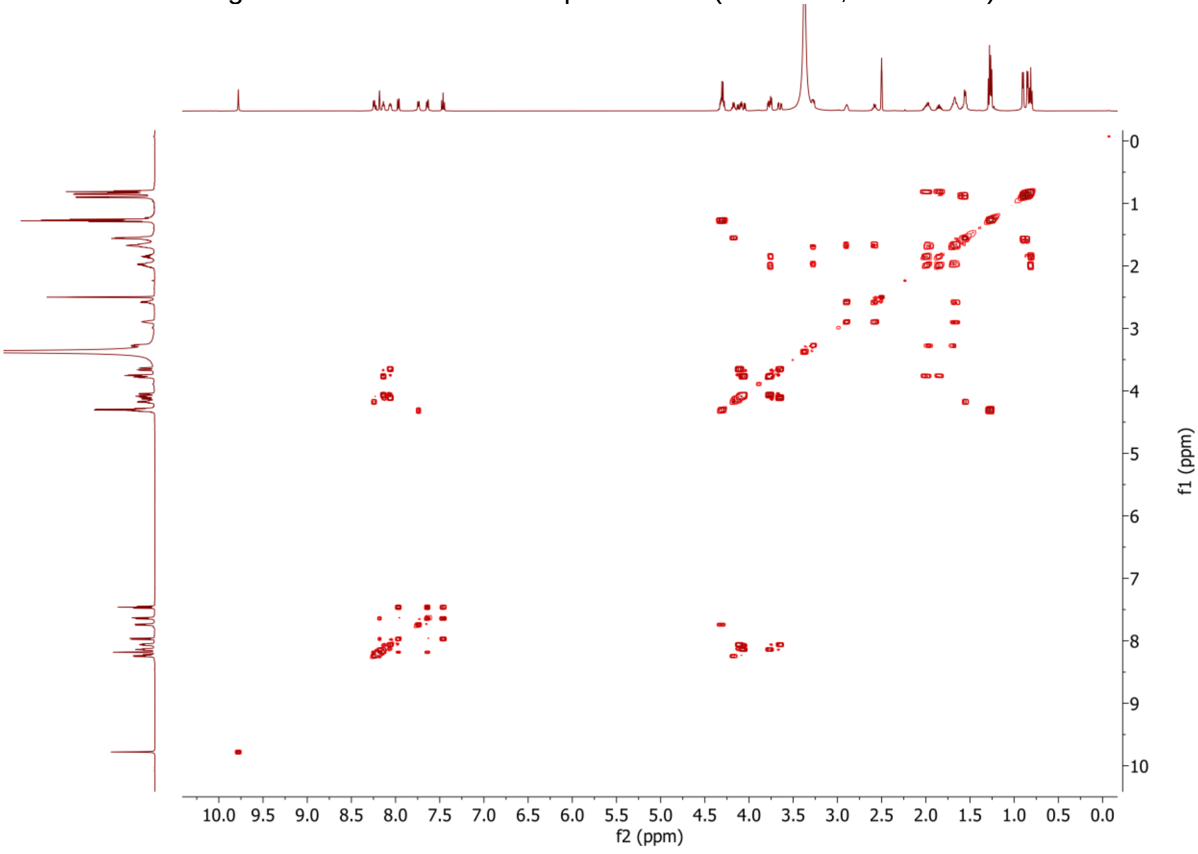

Figure S36: 2D-TOCSY Compound **11b** (500 MHz, DMSO-*d6*)

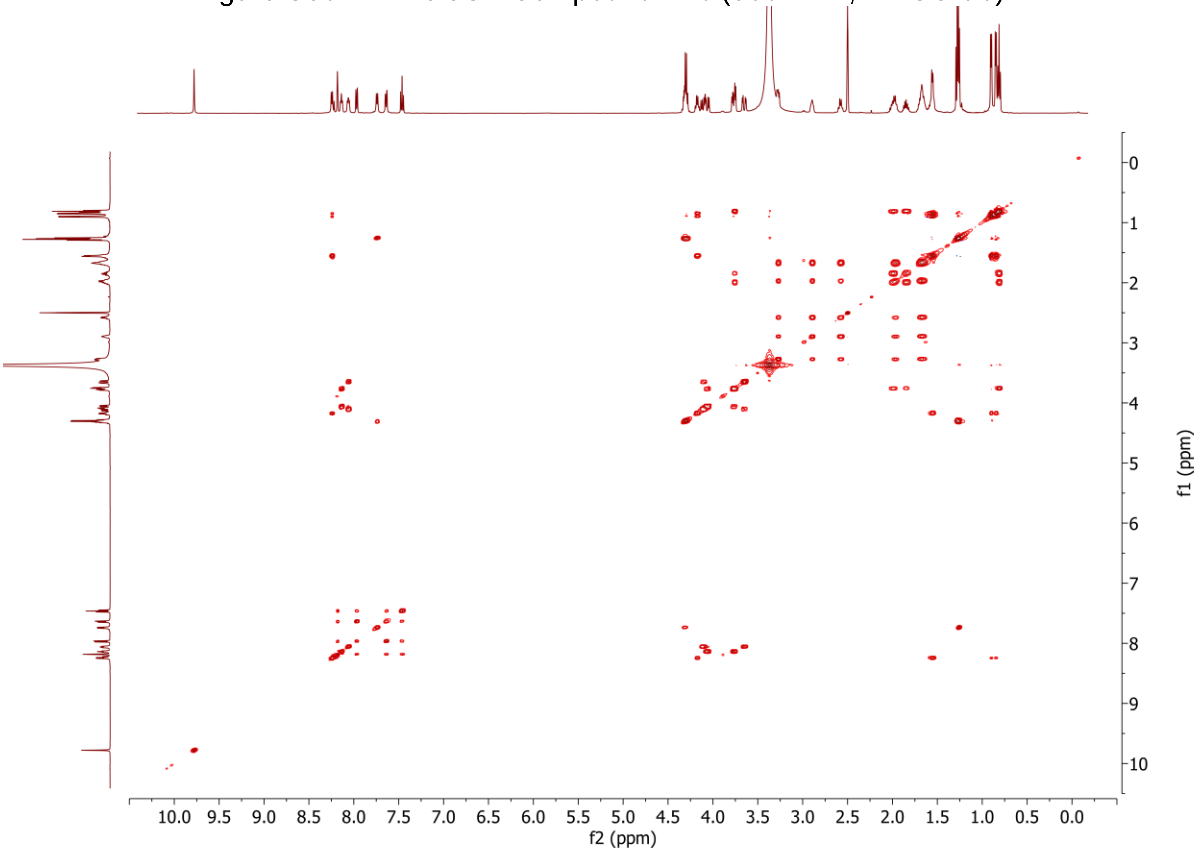

Figure S37: 2D-ROESY Compound **11b** (500 MHz, DMSO-*d6*)

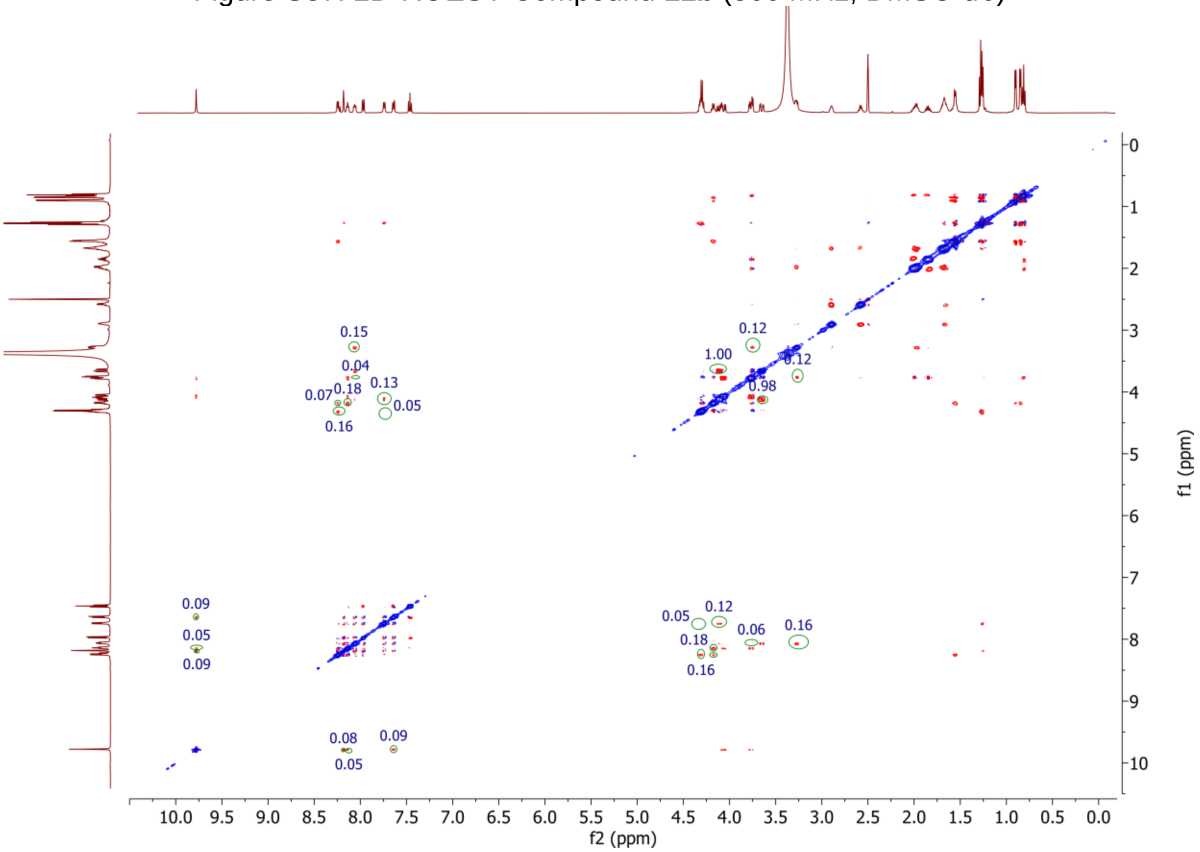

Figure S38: 2D-HSQC Compound **11b** (500 MHz, DMSO-*d*6)

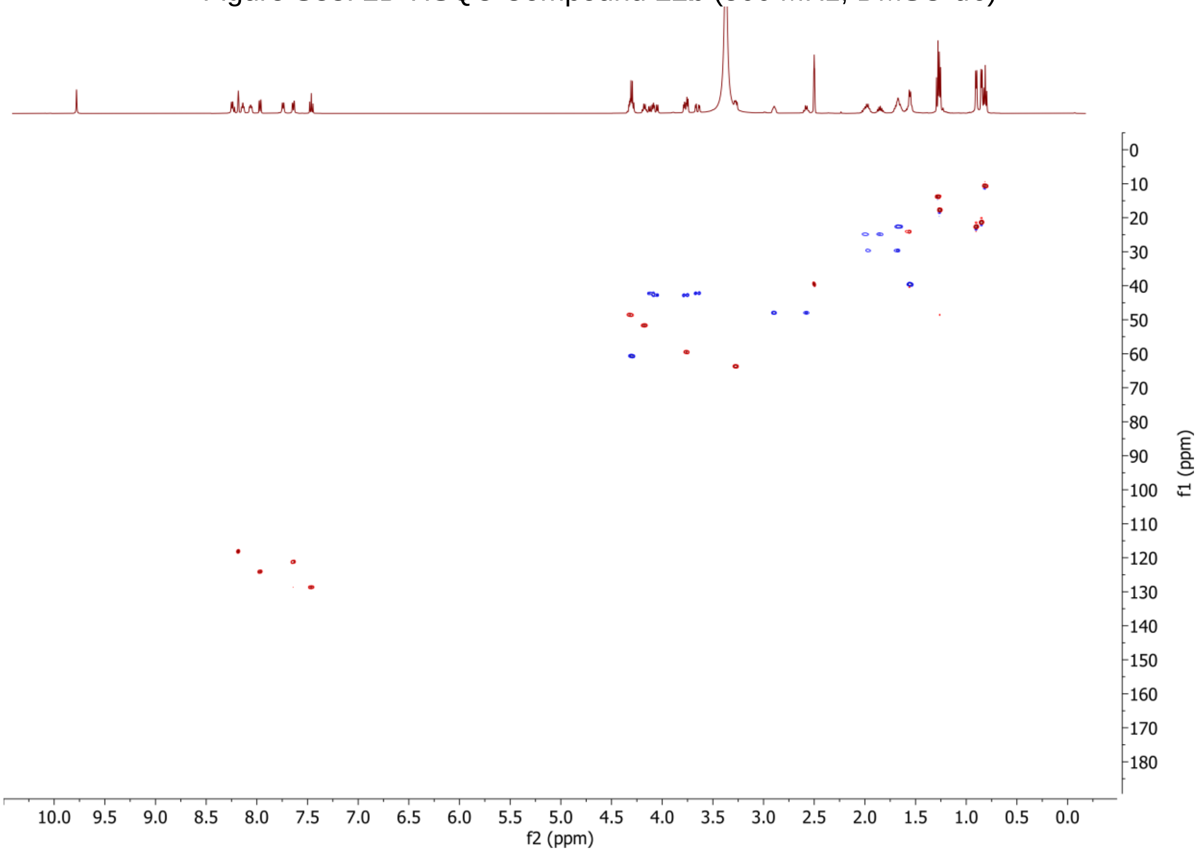

Figure S39: 2D-HMBC Compound **11b** (400 MHz, DMSO-*d*6)

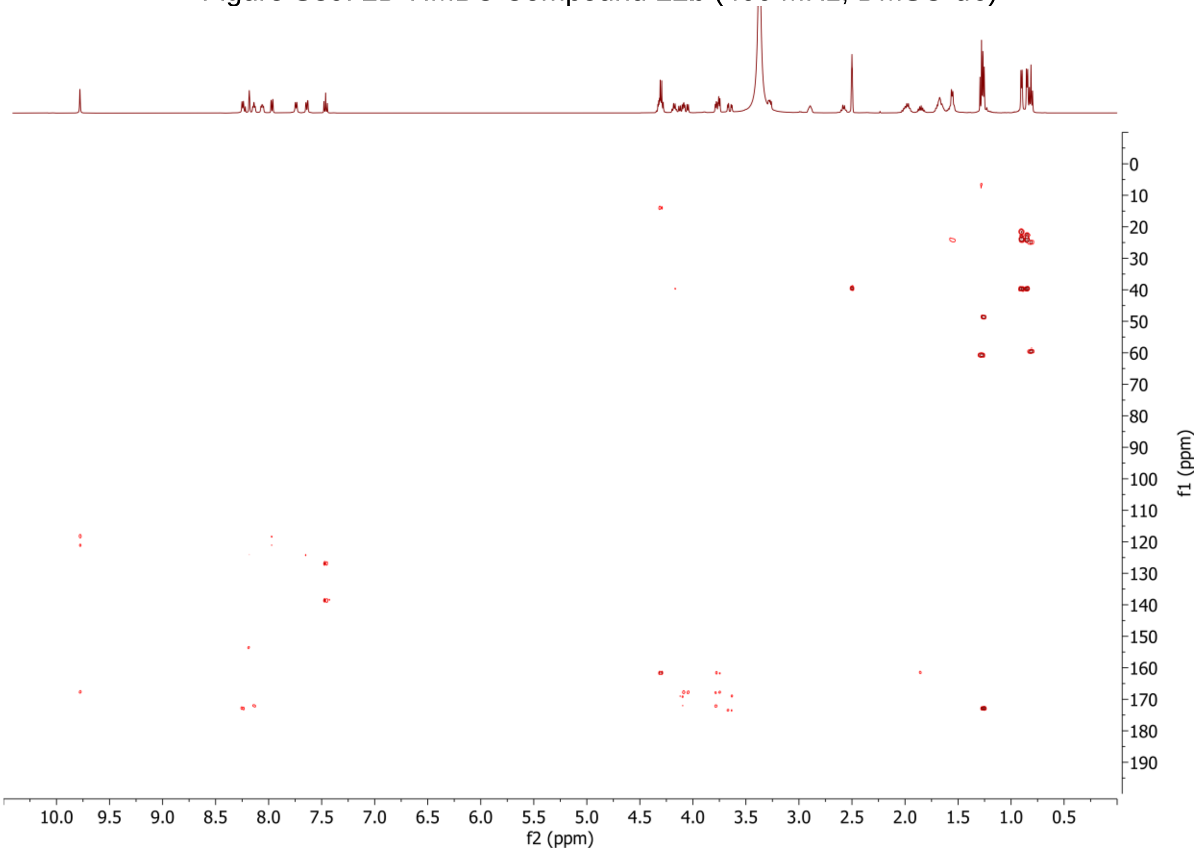

Figure S40: VT 1H-NMR Compound **11b** (500 MHz, DMSO-*d*6)

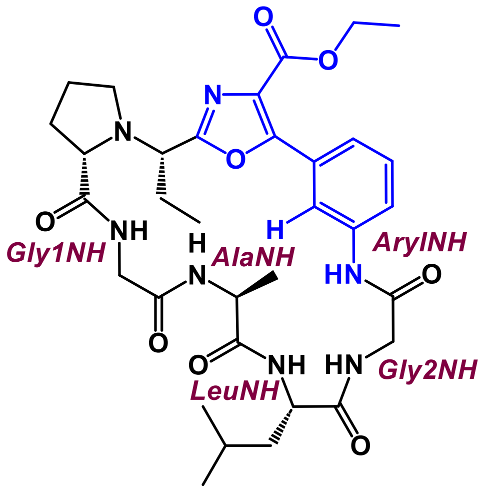

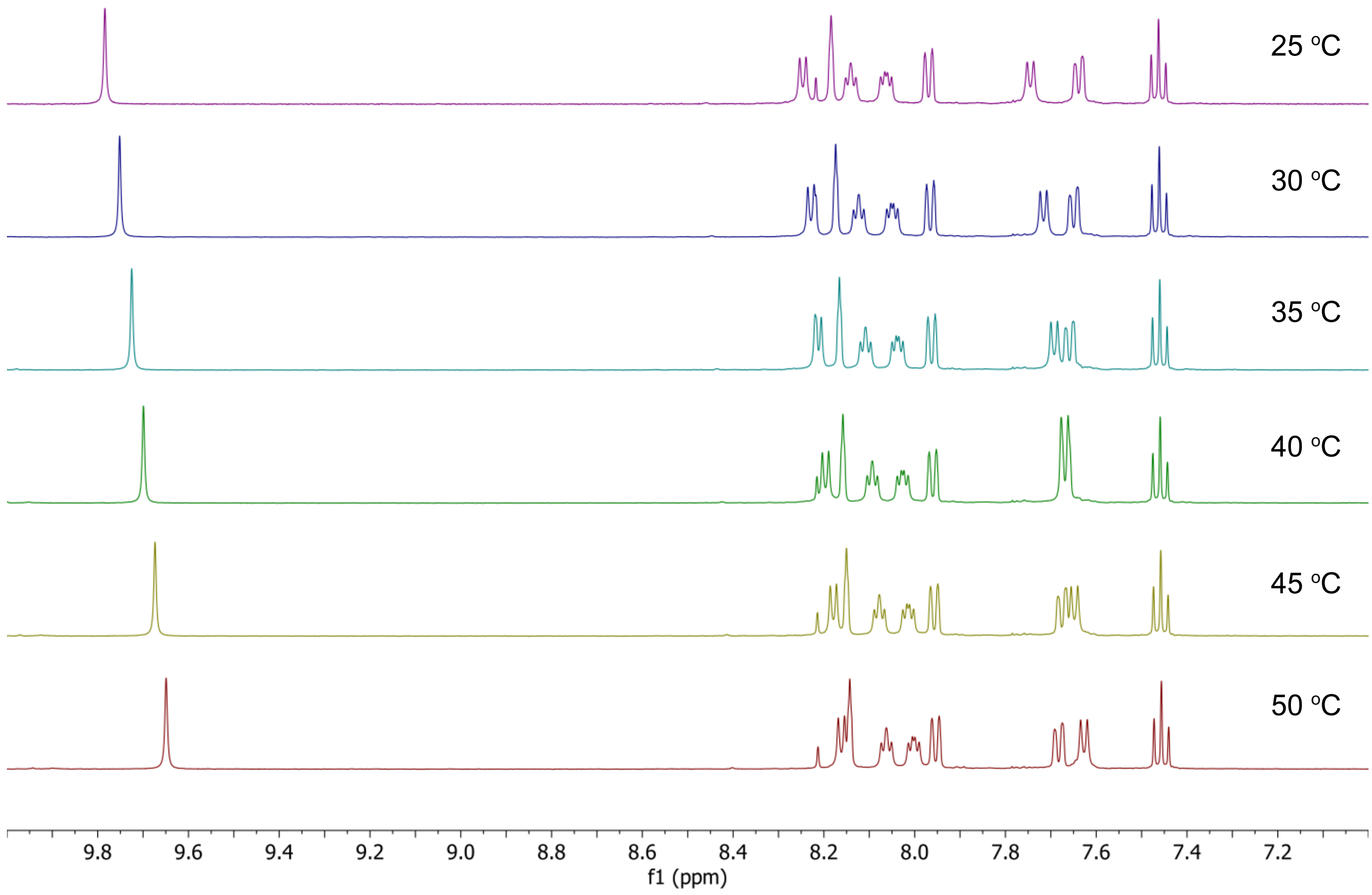

	Shifts (ppm)				
K	*ArylNH*	*Gly2NH*	*LeuNH*	*AlaNH*	*Gly1NH*
**298**	9.78	8.18	8.25	7.75	8.06
**323**	9.67	8.14	8.16	7.62	8.00
Δppb/k					
**298-323**	**4.4**	**1.6**	**3.6**	**2.4**	**2.4**

Table 5: Full Characterization of compound **12a**

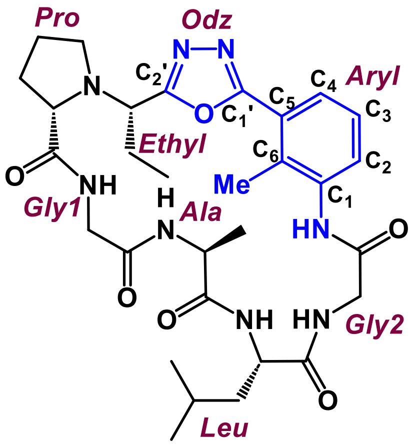

White solid obtained in 23 % overall yield (based on 100 % resin loading). ^{1}H NMR (500 MHz, DMSO-*d*6) δ 9.22 (s, 1H), 8.82 (t, *J* = 6.0 Hz, 1H), 8.15 (d, *J* = 7.6 Hz, 1H), 8.08 (dd, *J* = 7.2, 4.0 Hz, 1H), 7.82 (dd, *J* = 7.9, 1.3 Hz, 1H), 7.76 (d, *J* = 7.3 Hz, 1H), 7.67 (d, *J* = 7.9 Hz, 1H), 7.38 (t, *J* = 7.9 Hz, 1H), 4.35 (d, *J* = 7.2 Hz, 1H), 4.29 (t, *J* = 7.3 Hz, 1H), 4.14 – 4.03 (m, 2H), 3.96 (dd, *J* = 10.7, 4.9 Hz, 1H), 3.64 – 3.52 (m, 2H), 3.44 (dd, *J* = 9.8, 3.9 Hz, 2H), 2.78 (t, *J* = 7.4 Hz, 1H), 2.55 – 2.51 (m, 1H), 2.31 (s, 3H), 2.21 – 2.01 (m, 2H), 1.83 – 1.67 (m, 3H), 1.61 – 1.51 (m, 3H), 1.22 (d, *J* = 7.2 Hz, 3H), 0.91 – 0.87 (m, 6H), 0.72 (t, *J* = 7.3 Hz, 3H). HRMS (ESI+) m/z calc. for $C_{30}H_{43}N_8O_6^+$ $[M+H]^+$ = 611.3292, found 611.3300.

	1H Shifts
Ethyl	CH_3 (0.72), CH_2 (1.83 – 1.67, 2.21 – 2.01), αCH (3.96)
Gly2	αCH_2 (3.64 – 3.52, 4.14 – 4.03), NH (8.82)
Leu	αCH (4.35), βCH_2 (1.61 – 1.51), γCH (1.61 – 1.51), CH_3s (0.91 – 0.87) NH (7.76)
Ala	αCH (4.29), CH_3 (1.22), NH (8.15)
Gly1	αCH_2 (3.64 – 3.52, 4.14 – 4.03), NH (8.08)
Pro	αCH (3.44), βCH_2 (1.83 – 1.67, 2.21 – 2.01), γCH_2 (1.83 – 1.67, 2.21 – 2.01), δCH_2 (2.53, 2.77)
Aryl	NH (9.22), C_2H (7.67), C_3H (7.38), C_4H (7.82), C_6-CH_3 (2.31)
Odz	N/A

	C13 Shifts
Ethyl	CH_3 (10.4), CH_2 (24.3), αCH (59.8)
Gly2	αC (43.6), C=O (168.4)
Leu	αC (50.9), βC (40.7), γC (23.8), CH_3s (22.7, 21.6), C=O (173.0)
Ala	αC (48.1), CH_3 (17.8), C=O (172.1)
Gly1	αC (41.3), C=O (168.6)
Pro	αC (62.4), βC (30.1), γC (24.4), δC (51.5), C=O (173.9)
Aryl	C_1 (137.3), C_2 (128.3), C_3 (126.2), C_4 (126.5), C_5 (124.2), C_6 (132.2). C_6CH_3 (14.8), C=O (168.1)
Odz	C_1' (165.3), C_2' (166.5)

Figure S41:1H-NMR **12a** (500 MHz, DMSO-*d*6)

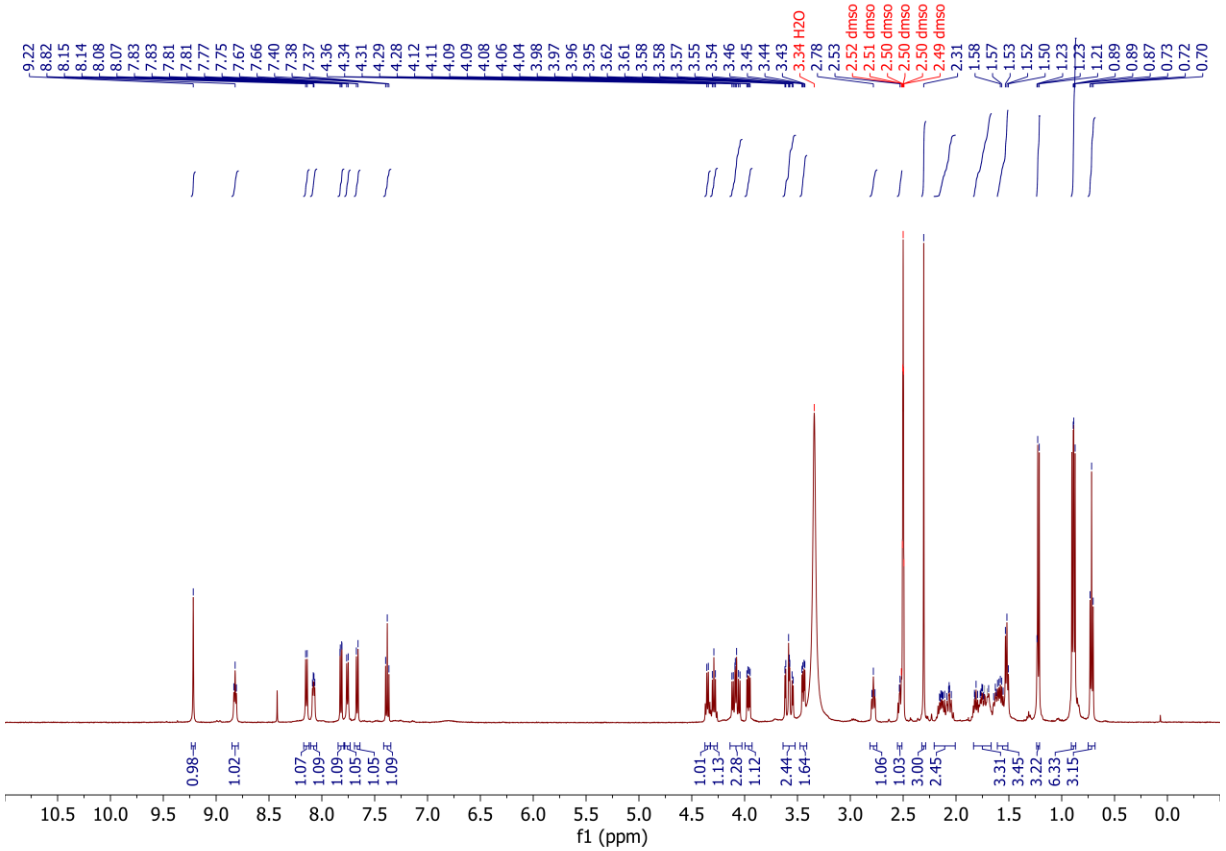

Figure S42: 2D-COSY Compound **12a** (500 MHz, DMSO-*d*6)

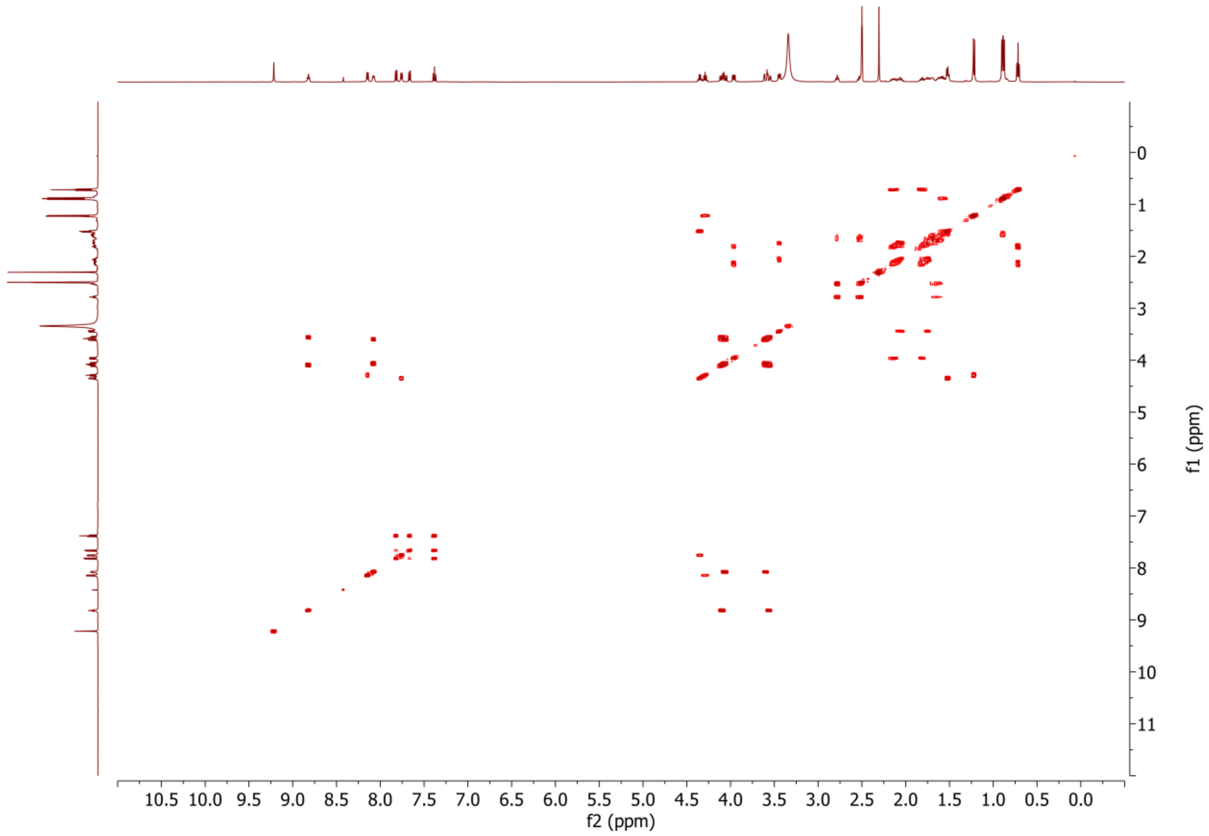

Figure S43: 2D-TOCSY Compound **12a** (500 MHz, DMSO-*d*6)

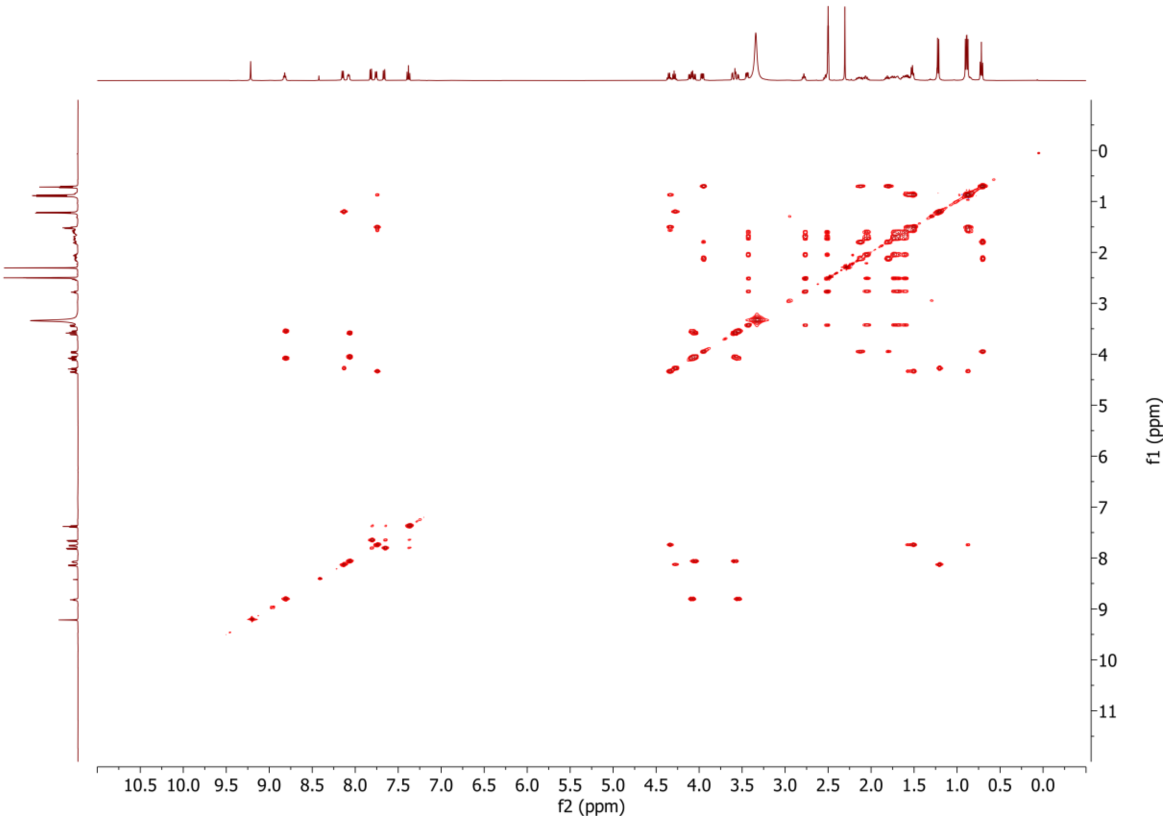

Figure S44: 2D-ROESY Compound **12a** (500 MHz, DMSO-*d*6)

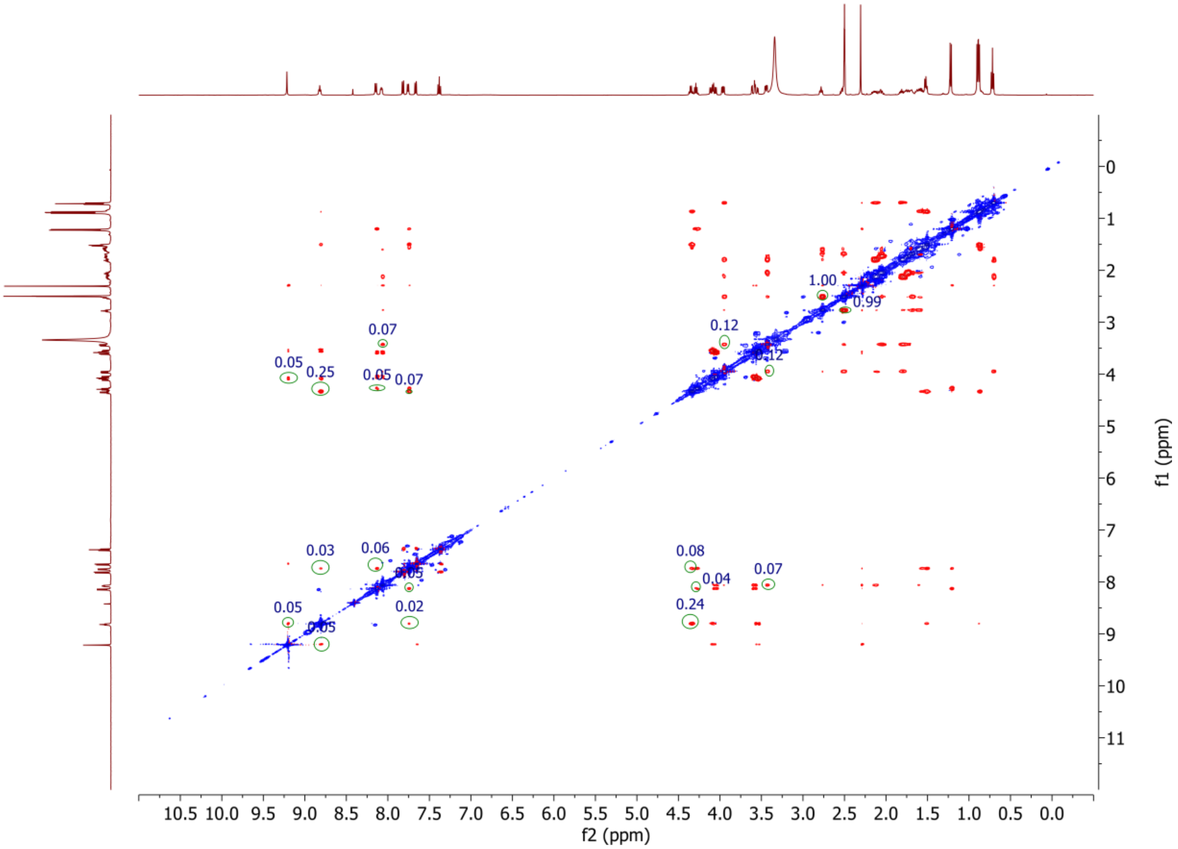

Figure S45: 2D-HSQC Compound **12a** (500 MHz, DMSO-*d*6)

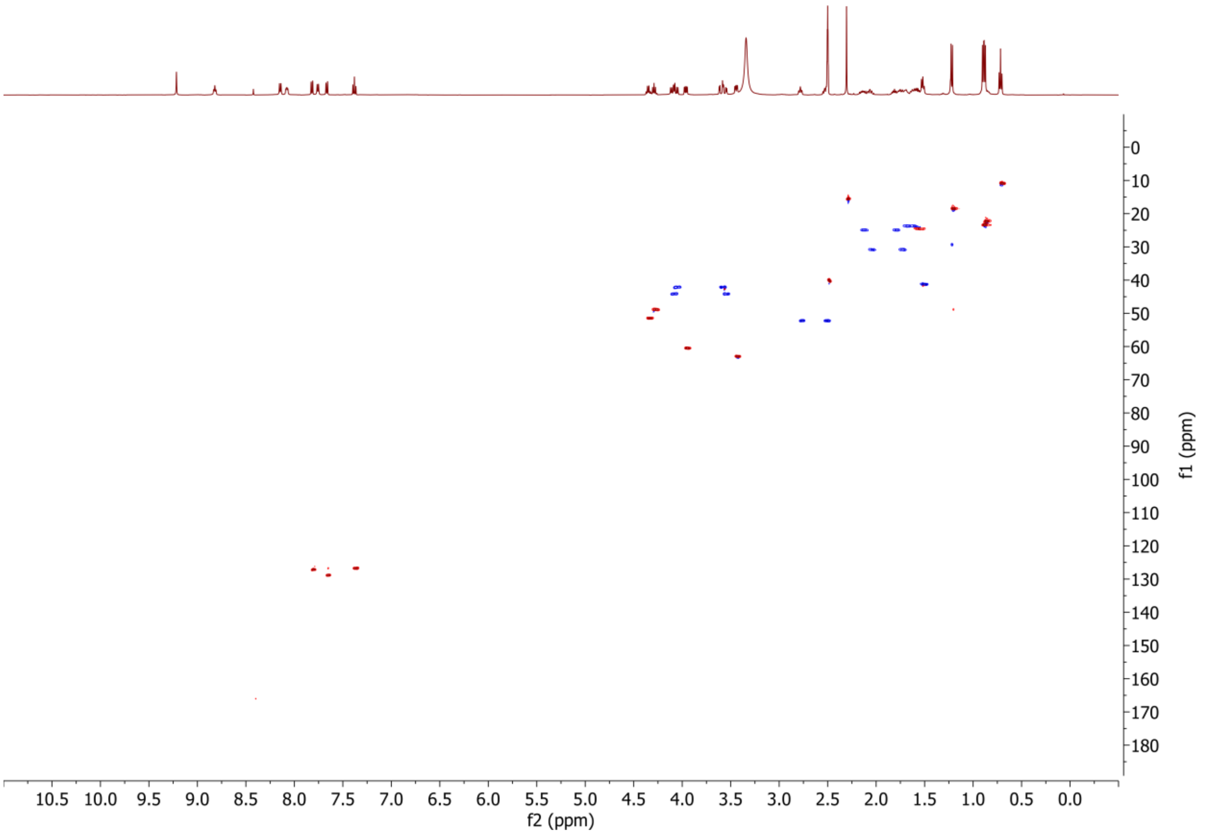

Figure S46: 2D-HMBC Compound **12a** (500 MHz, DMSO-*d*6)

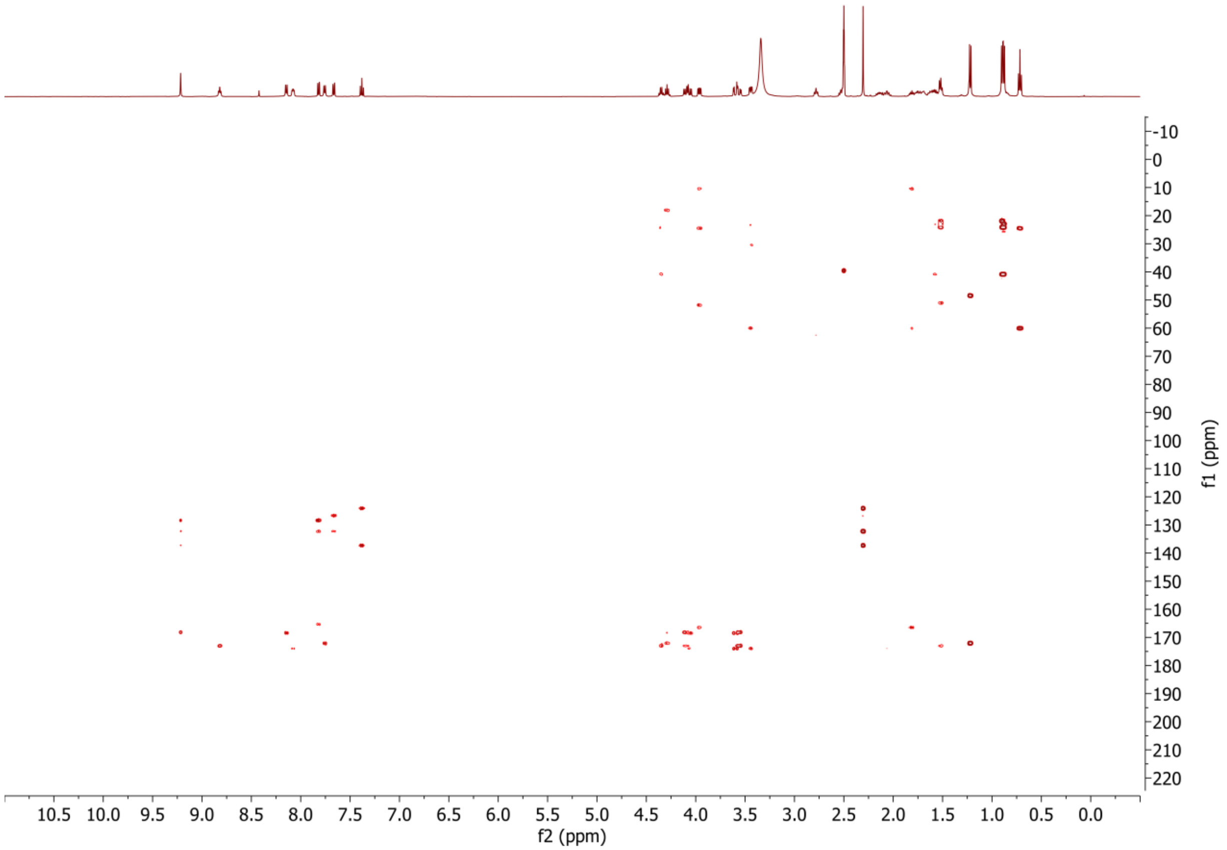

Figure S47: VT 1H-NMR Compound **12a** (500 MHz, DMSO-*d*6)

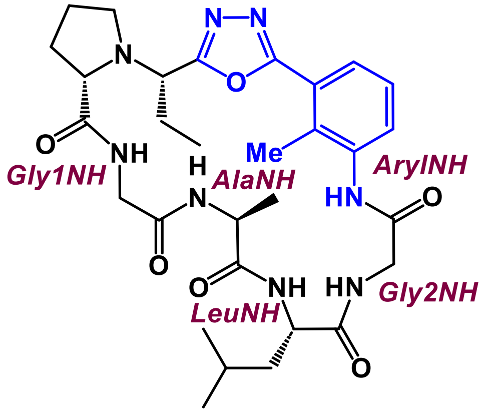

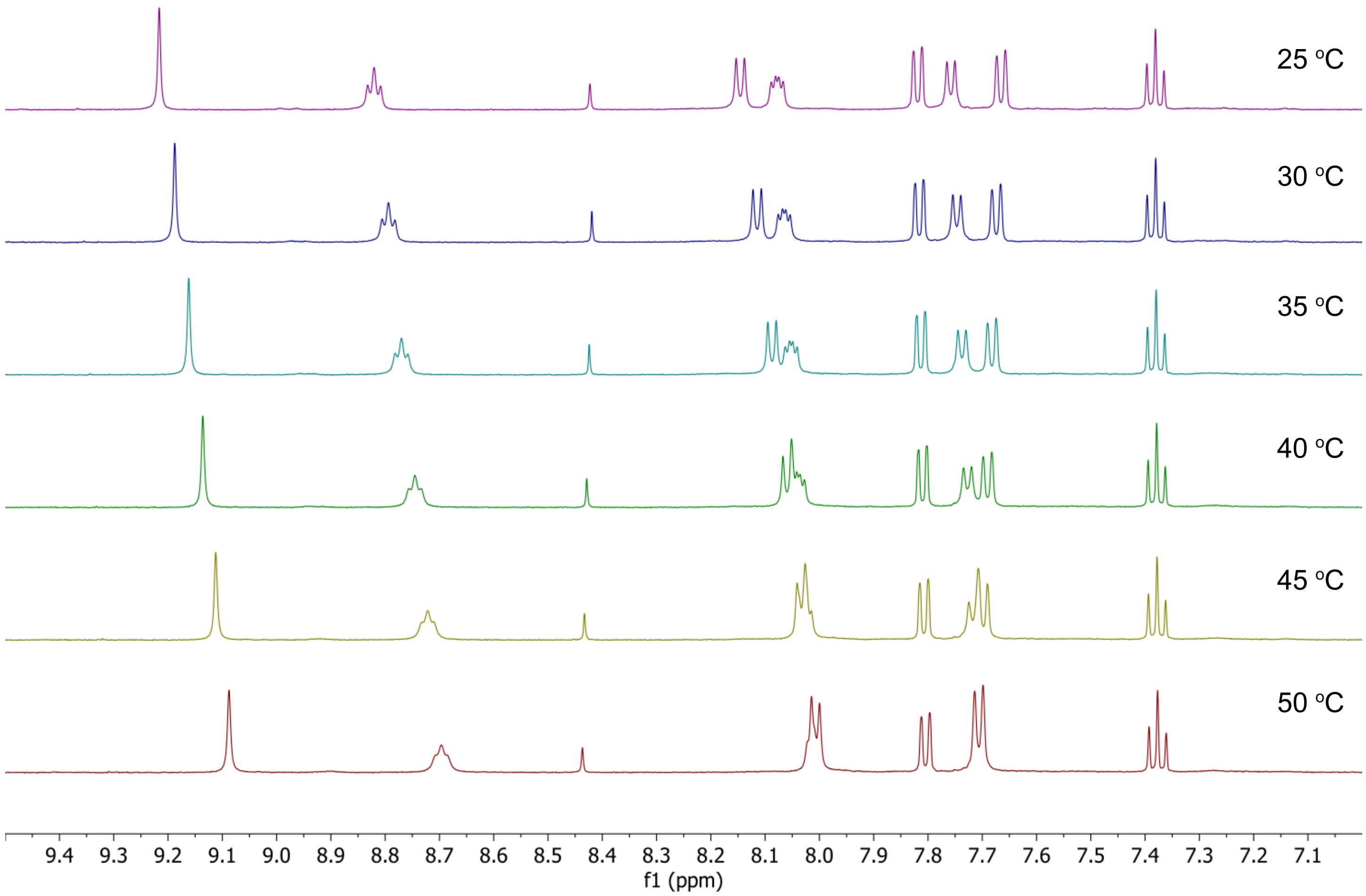

Shifts (ppm)					
K	*ArylNH*	*Gly2NH*	*LeuNH*	*AlaNH*	*Gly1NH*
**298**	9.22	8.82	7.76	8.15	8.08
**323**	9.07	8.68	7.69	7.98	8.04
Δppb/k					
**298-323**	**6**	**5.6**	**2.8**	**6.8**	**1.6**

Table 6: Full Characterization of compound **12b**

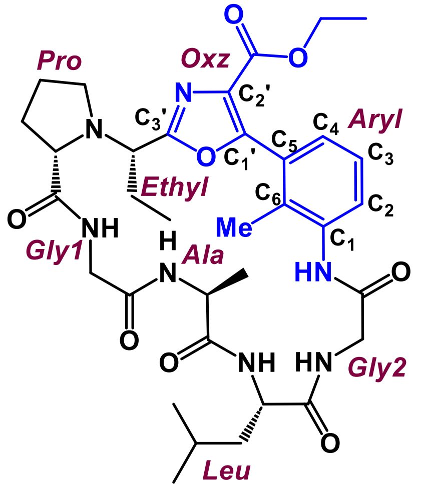

White solid obtained in a 23 % overall yield (based on 100 % resin loading).^{1}H NMR (500 MHz, DMSO-*d*6) δ 8.87 (s, 1H), 8.80 (t, *J* = 6.0 Hz, 1H), 8.12 – 8.05 (m, 2H), 7.86 (d, *J* = 7.6 Hz, 1H), 7.64 (d, *J* = 7.7 Hz, 1H), 7.41 (d, *J* = 7.6 Hz, 1H), 7.28 (t, *J* = 7.9 Hz, 1H), 4.36 – 4.29 (m, 1H), 4.26 – 4.17 (m, 3H), 4.13 (dd, *J* = 16.3, 7.0 Hz, 1H), 3.96 (dd, *J* = 16.5, 6.6 Hz, 1H), 3.79 (dd, *J* = 10.5, 4.9 Hz, 1H), 3.64 – 3.54 (m, 2H), 3.30 (dd, *J* = 9.6, 4.5 Hz, 1H), 2.81 – 2.75 (3, 1H), 2.61 – 2.55 (m, 1H), 2.12 (s, 3H), 2.02 (d, *J* = 6.9 Hz, 1H), 1.84 – 1.66 (m, 3H), 1.64 - 1.54 (m, 2H), 1.55 – 1.48 (m, 2H), 1.22 – 1.11 (m, 6H), 0.91 (d, *J* = 6.5 Hz, 3H), 0.87 (dd, 6.5 Hz, 6H), 0.73 (t, *J* = 7.4 Hz, 3H). HRMS (ESI+) m/z calc. for $C_{34}H_{48}N_7O_8^+$ $[M+H]^+$ = 682.3559, found 682.3559.

	**1H Shifts**
Ethyl	CH_3 (0.73), CH_2 (1.84 – 1.66, 2.00), αCH (3.79)
Gly2	αCH_2 (3.64 – 3.54, 4.132), NH (8.80)
Leu	αCH (4.26 – 4.17), βCH_2 (1.55 – 1.48, 1.64 - 1.54), γCH (1.55 – 1.48), CH_3s (0.86, 0.90) NH (8.12 – 8.05)
Ala	αCH (4.36 – 4.29), CH_3 (1.14), NH (7.86)
Gly1	αCH_2 (3.64 – 3.54, 3.96), NH (8.12 – 8.05)
Pro	αCH (3.30), βCH_2 (1.84 – 1.66, 2.02), γCH_2 (1.64 - 1.54, 1.84 – 1.66), δCH_2 (2.61 – 2.55, 2.81 – 2.75)
Aryl	NH (8.87), C_2H (7.41), C_3H (7.28), C_4H (7.64), C_6-CH_3 (2.12)
Oxz	CH_2 (4.26 – 4.17), CH_3 (1.18)

	**C13 Shifts**
Ethyl	CH_3 (10.5), CH_2 (25.0), αCH (61.3)
Gly2	αC (43.4), C=O (172.9)
Leu	αC (51.4), βC (23.7), γC (39.8), CH_3s (22.5, 21.4), C=O (172.5)
Ala	αC (47.8), CH_3 (17.9), C=O (168.4)
Gly1	αC (42.0), C=O (173.8)
Pro	αC (63.0), βC (29.7), γC (22.7), δC (50.4), C=O (173.8)
Aryl	C_1 (136.3), C_2 (126.3), C_3 (125.1), C_4 (128.5), C_5 (127.3), C_6 (131.3). C_6CH_3 (15.0), C=O (168.2)
Oxz	C_1' (155.3), C_2' (n.d), C_3' (162.3), CH_2 (60.3), CH_3 (13.7), C=O (161.0)

Figure S48:1H-NMR **12b** (500 MHz, DMSO-*d*6)

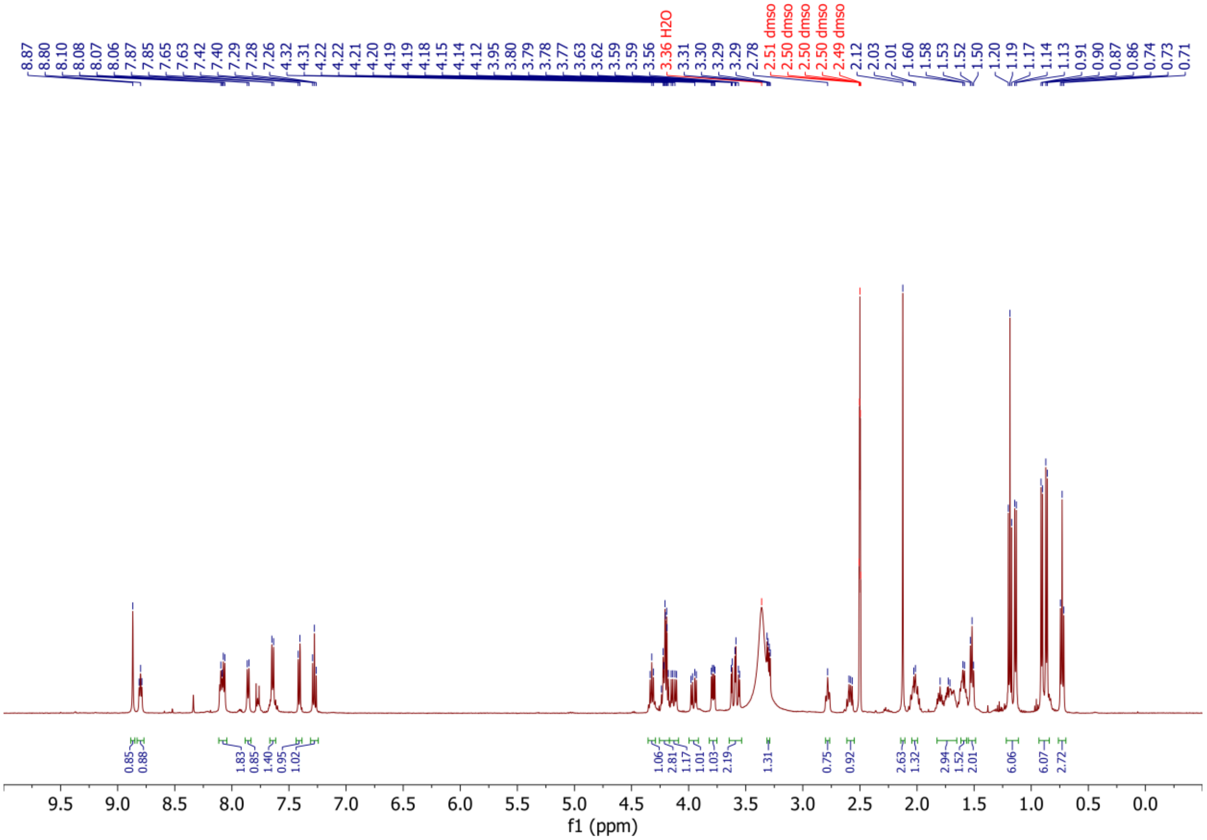

Figure S49: 2D-COSY Compound **12a** (500 MHz, DMSO-*d*6)

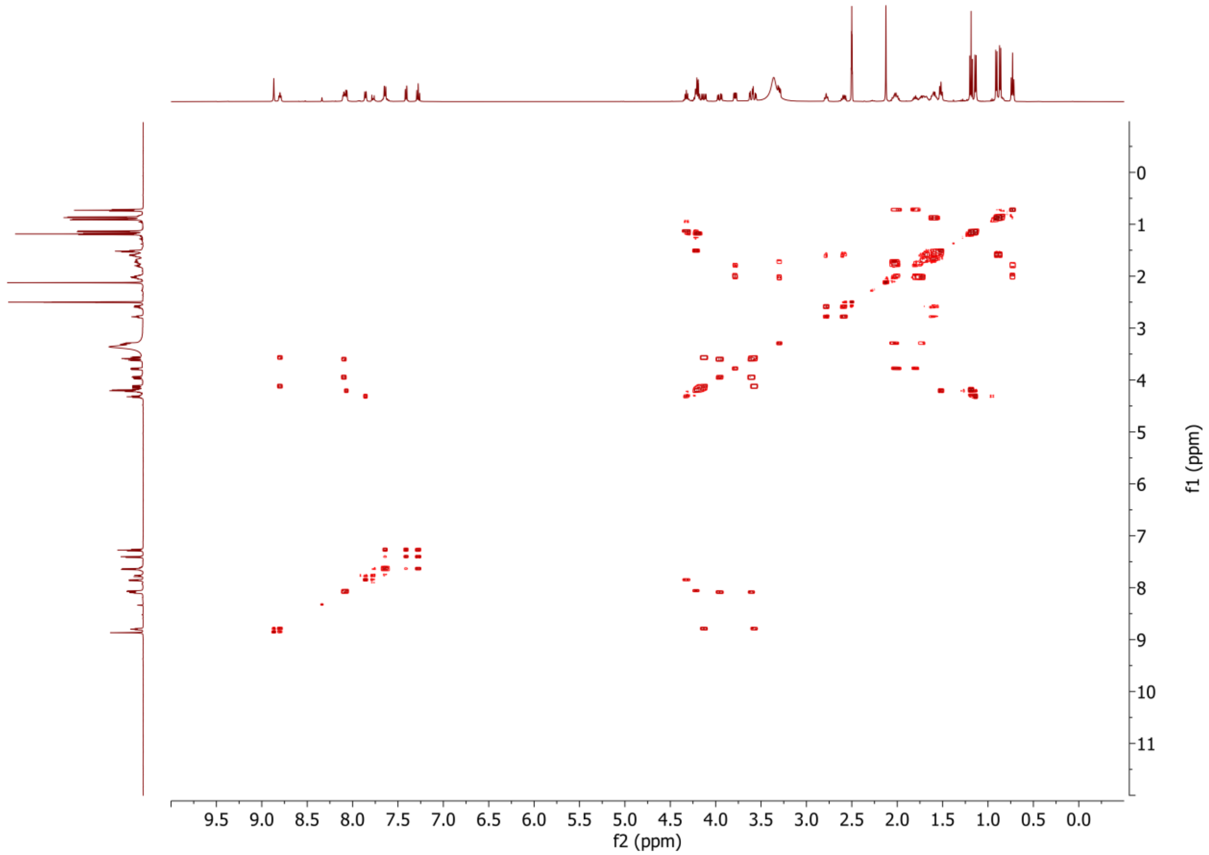

Figure S50: 2D-TOCSY Compound **12b** (500 MHz, DMSO-*d*6)

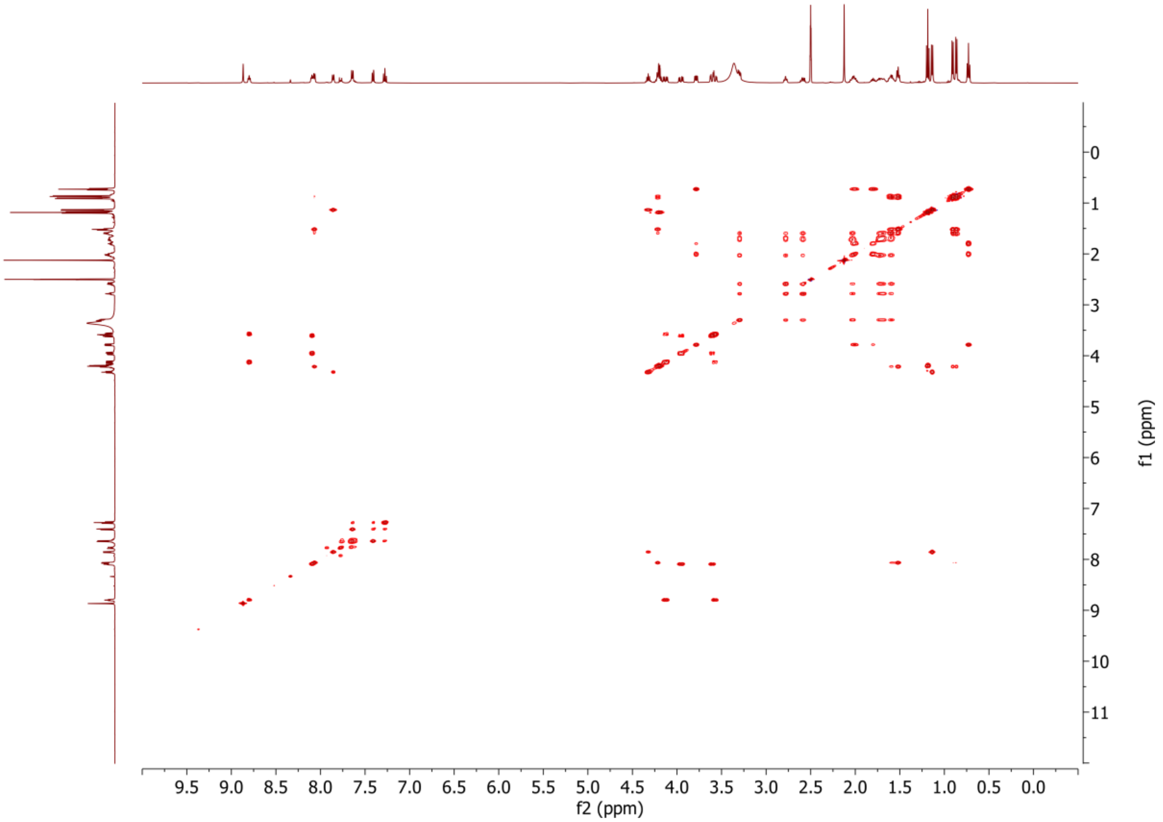

Figure S51: 2D-ROESY Compound **12b** (500 MHz, DMSO-*d*6)

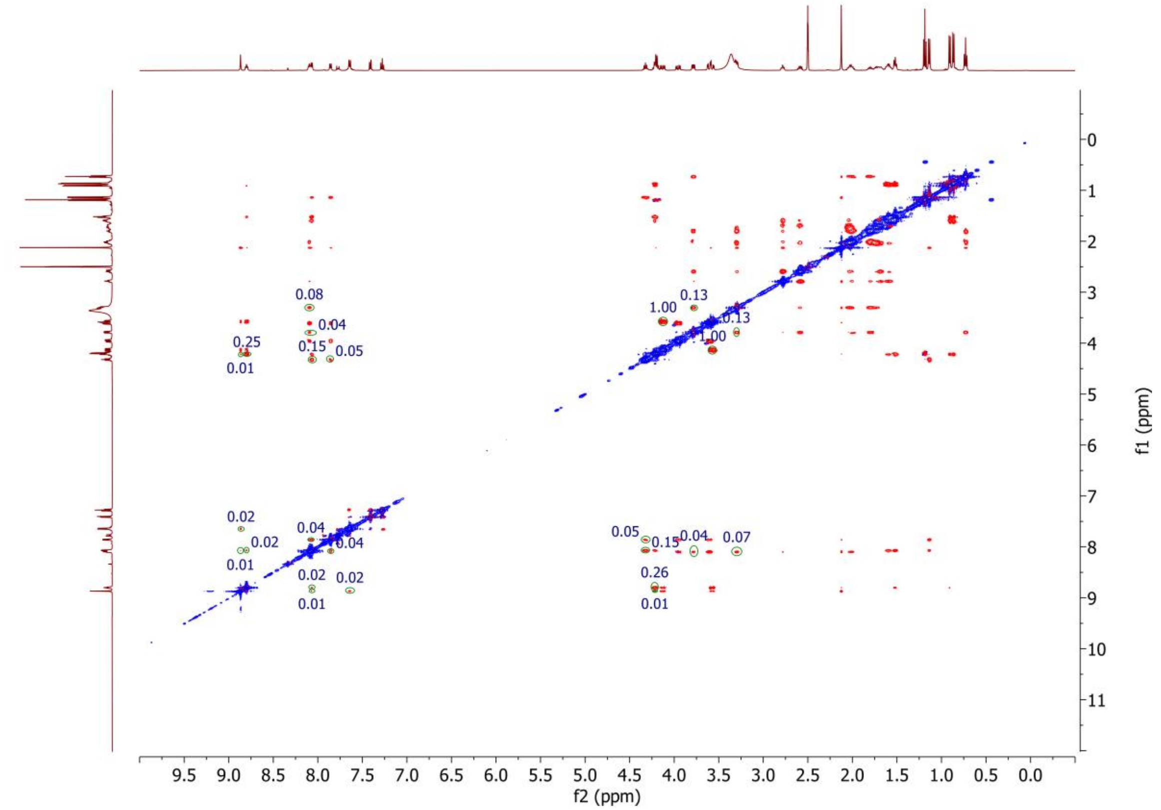

Figure S52: 2D-HSQC Compound **12b** (500 MHz, DMSO-*d*6)

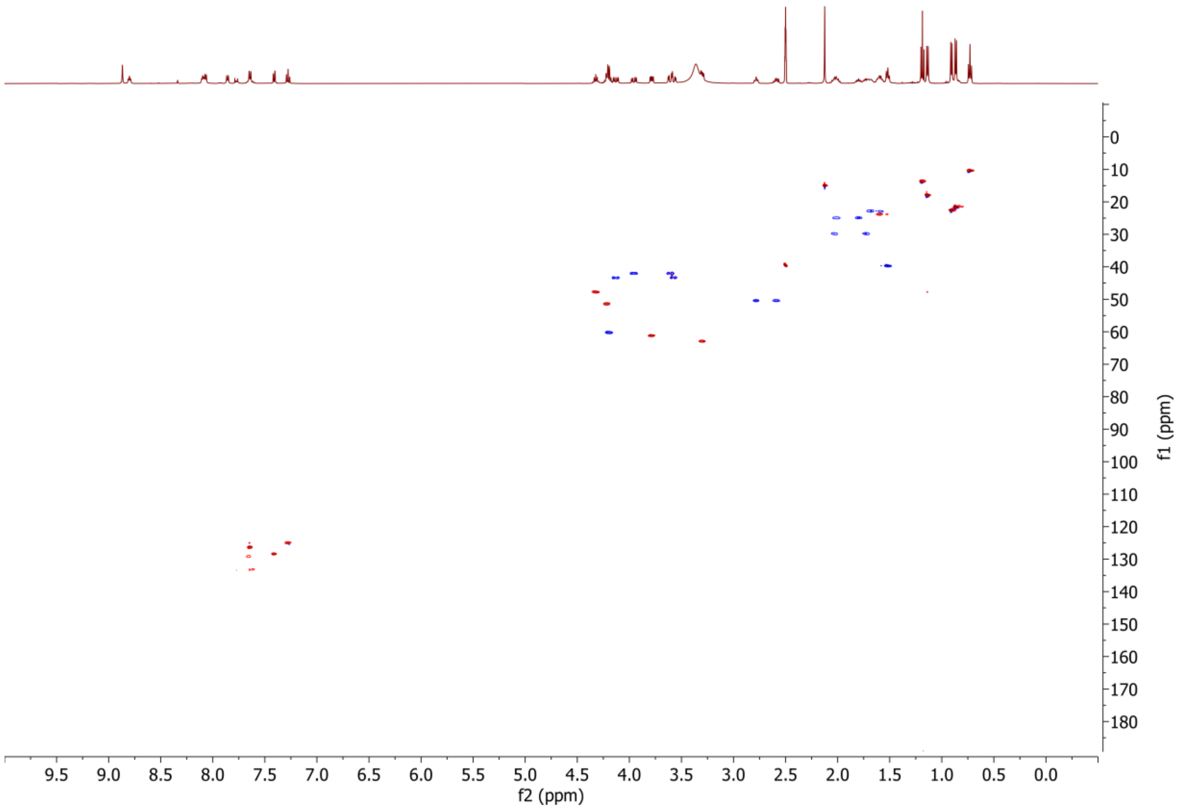

Figure S53: 2D-HMBC Compound **12b** (500 MHz, DMSO-*d*6)

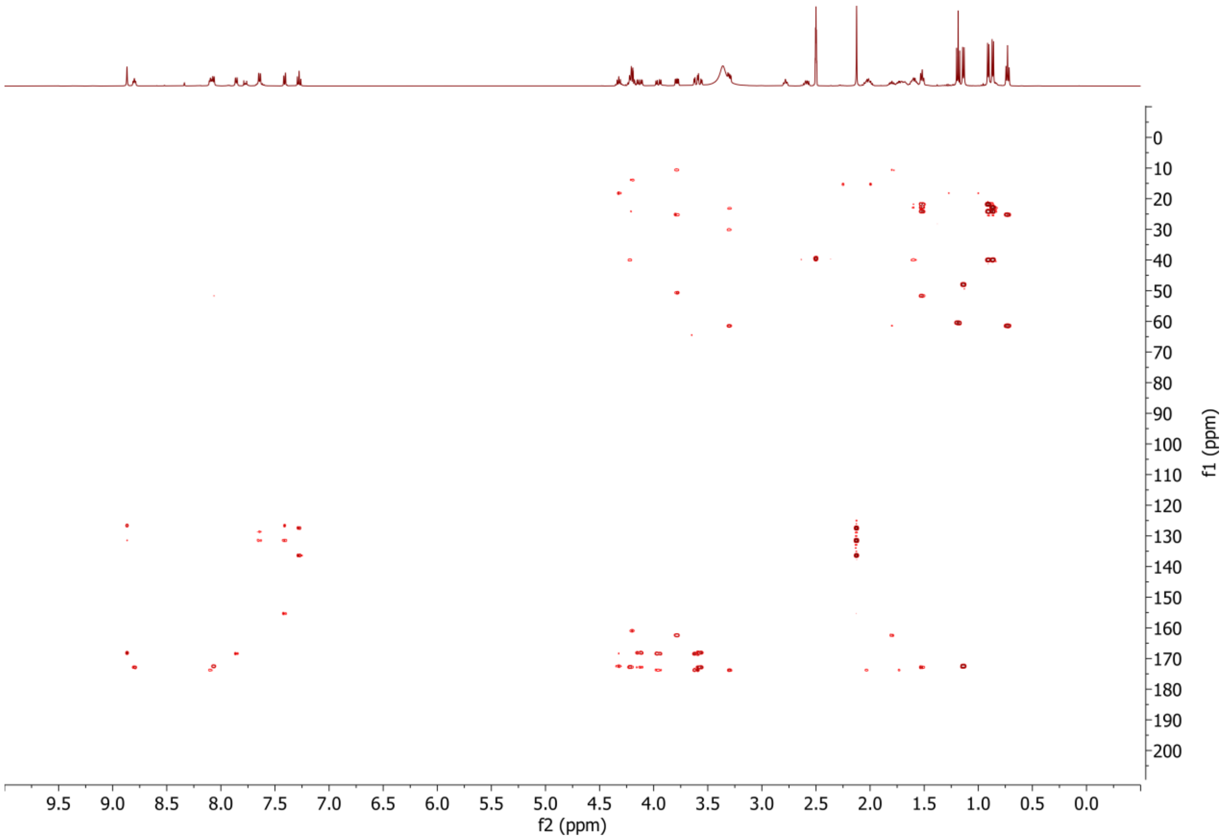

Figure S54: VT 1H-NMR Compound **12b** (500 MHz, DMSO-*d*6)

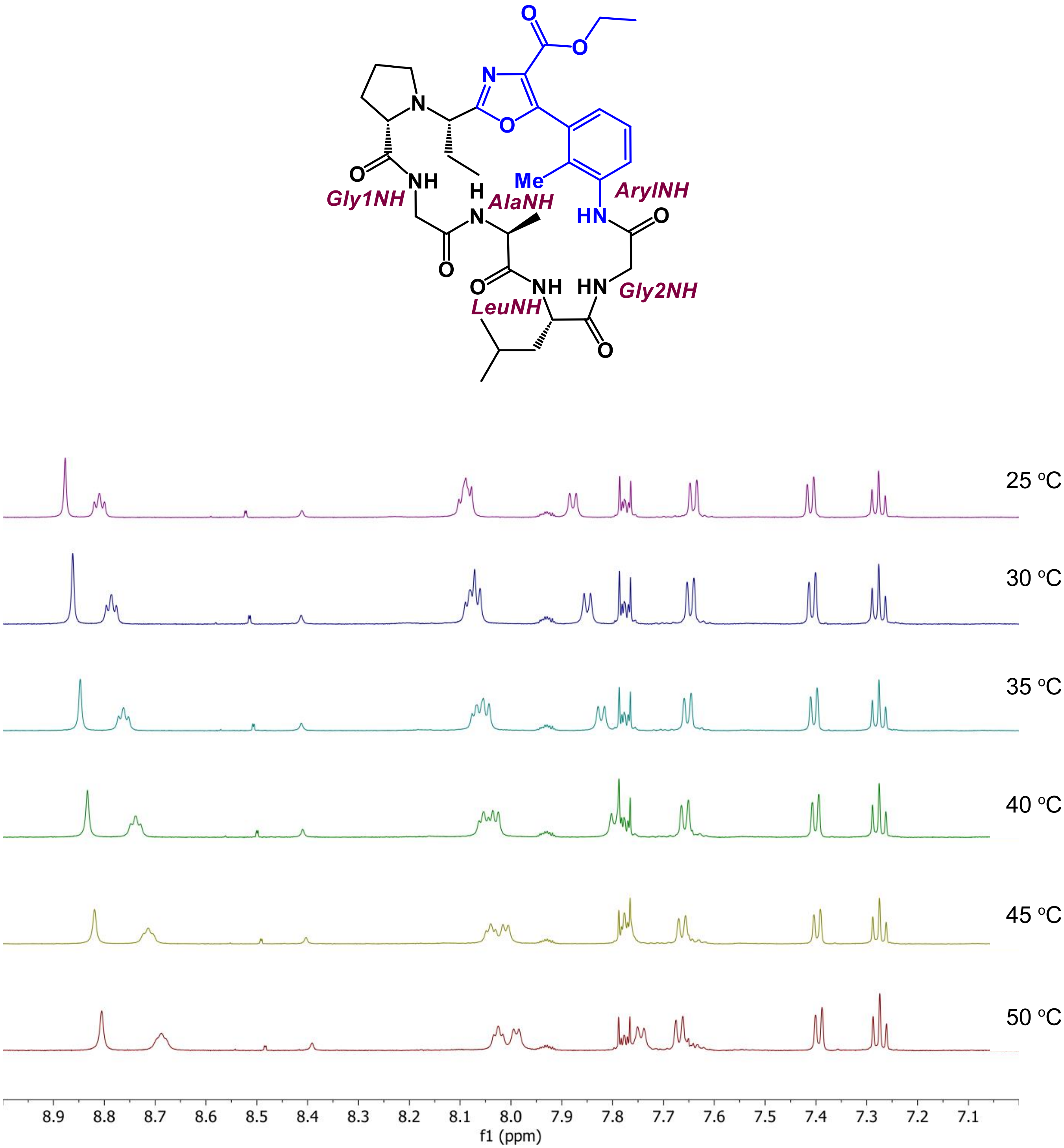

	Shifts (ppm)				
K	*ArylNH*	*Gly2NH*	*LeuNH*	*AlaNH*	*Gly1NH*
**298**	8.88	8.81	8.09	7.88	8.09
**323**	8.81	8.69	7.99	7.75	8.02
Δppb/k					
**298-323**	**2.5**	**2.7**	**2.4**	**2.4**	**2.3**

Table 7: Full Characterization of compound **14a**

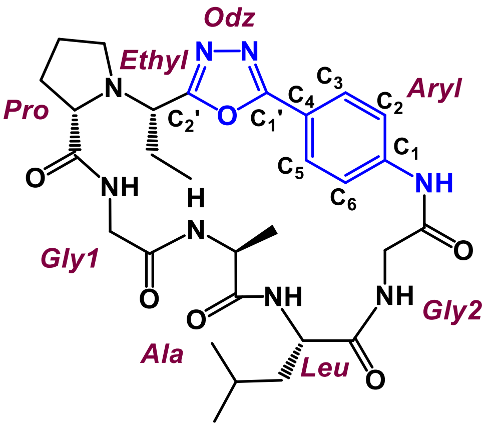

White solid obtained in a 18 % overall yield (based on 100 % resin loading).^{1}H NMR (600 MHz, DMSO-*d*6) δ 9.48 (s, 1H), 9.03 – 8.97 (m, 1H), 8.20 – 8.16 (m, 1H), 8.15 (d, *J* = 8.6 Hz, 1H), 7.97 – 7.91 (m, 2H), 7.85 (d, *J* = 8.5 Hz, 2H), 7.80 (s, 1H), 4.55 (p, *J* = 7.4 Hz, 1H), 4.16 (dd, J = 9.4, 5.9 Hz, 1H), 4.12 – 4.05 (m, 1H), 4.04 – 3.97 (m, 1H), 3.96 – 3.91 (m, 1H), 3.66 – 3.57 (m, 1H), 3.50 (m, 1H), 3.34 (m, 1H), 3.15 – 3.11 (m, 1H), 2.83 (q, *J* = 7.9 Hz, 1H), 1.99 – 1.90 (m, 2H), 1.70 – 1.66 (m, 3H), 1.62 – 1.55 (m, 3H), 1.54 – 1.46 (m, 1H), 1.27 (d, *J* = 7.2 Hz, 3H), 0.93 (d, *J* = 7.2 Hz, 3H), 0.87 (d, *J* = 7.2 Hz, 3H), 0.82 (t, *J* = 7.4 Hz, 3H). HRMS (ESI+) m/z calc. for $C_{29}H_{41}N_8O_8^+$ $[M+H]^+$ = 597.3155, found 597.3144.

	1H Shifts
Ethyl	CH_3 (0.82), CH_2 (1.99 – 1.90), αCH (4.16)
Gly2	αCH_2 (4.12 - 4.05, 3.66 – 3.57), NH (9.03 – 8.97)
Leu	αCH (4.04 – 3.97), βCH_2 (1.70 – 1.66, 1.62 – 1.55), γCH (1.54 – 1.46), CH_3s (0.93, 0.87) NH (8.20 – 8.16)
Ala	αCH (4.55), CH_3 (1.27), NH (8.15)
Gly1	αCH_2 (3.50, 3.34), NH (7.80)
Pro	αCH (3.96 – 3.91), βCH_2 (3.15 – 3.11, 2.83), γCH_2 (1.70 – 1.66), δCH_2 (1.62 – 1.55)
Aryl	NH (9.48), C_2H (7.97 - 7.91), C_3H (7.85), C_5H (7.85), C_6H (7.97 - 7.91)
Odz	N/A

Figure S55:1H-NMR **14a** (600 MHz, DMSO-*d*6)

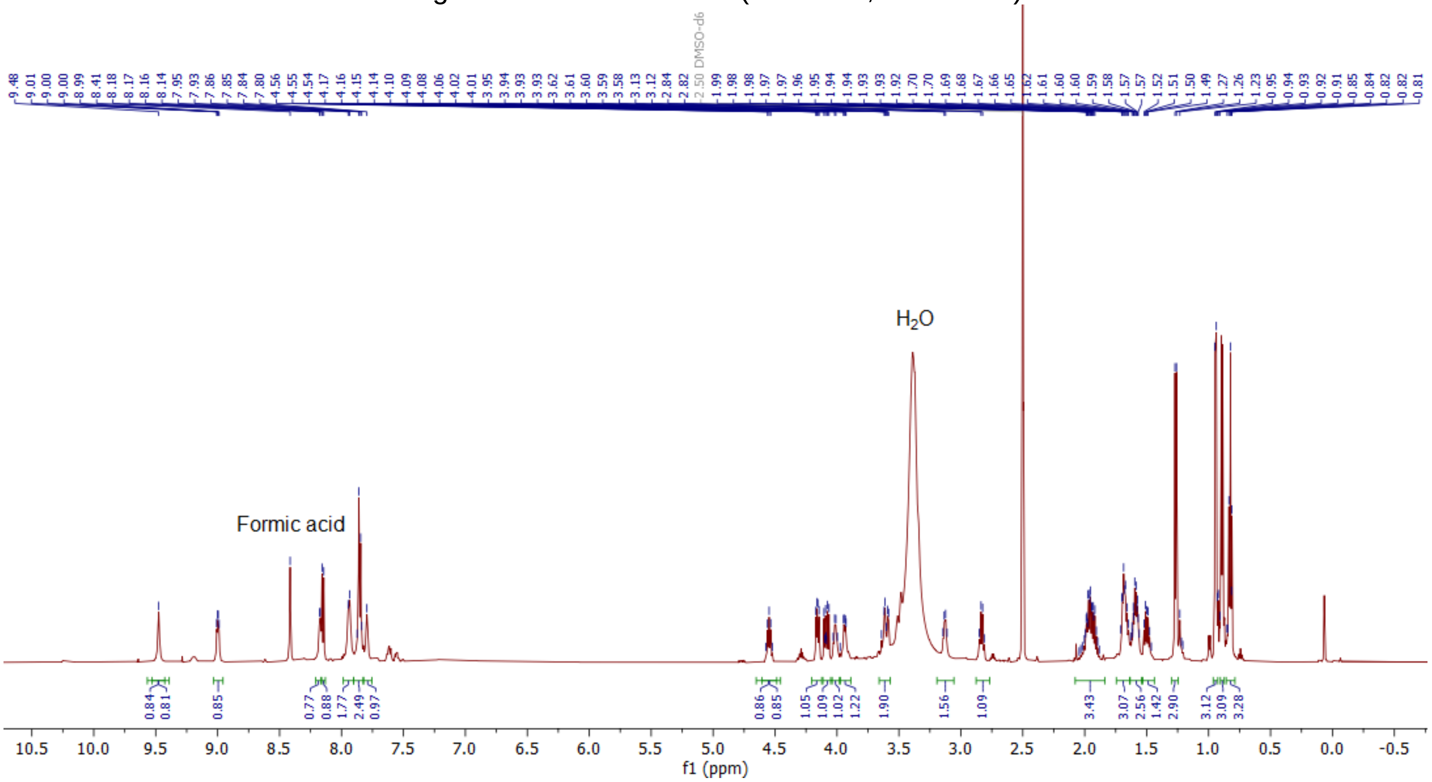

Figure S56: 2D-COSY Compound **14a** (600 MHz, DMSO-*d*6)

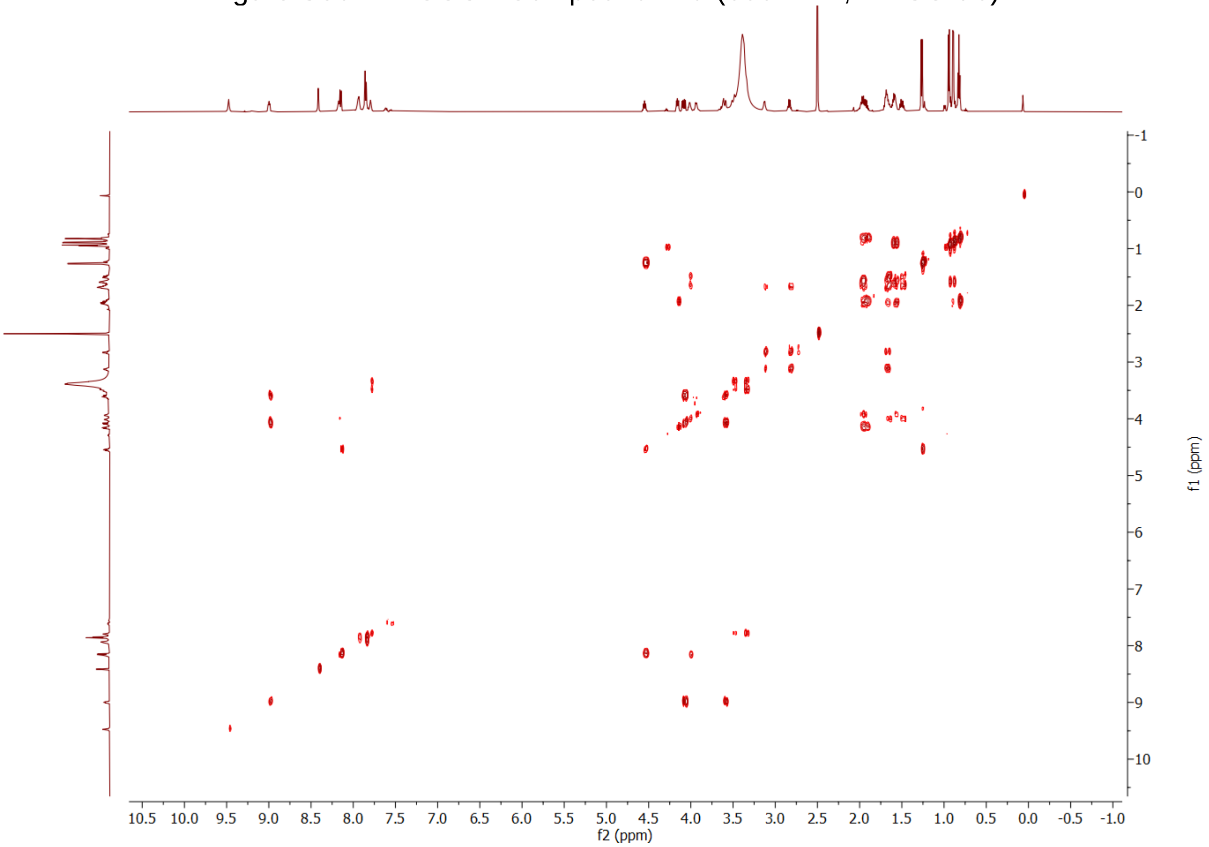

Figure S57: 2D-TOCSY Compound **14a** (600 MHz, DMSO-*d*6)

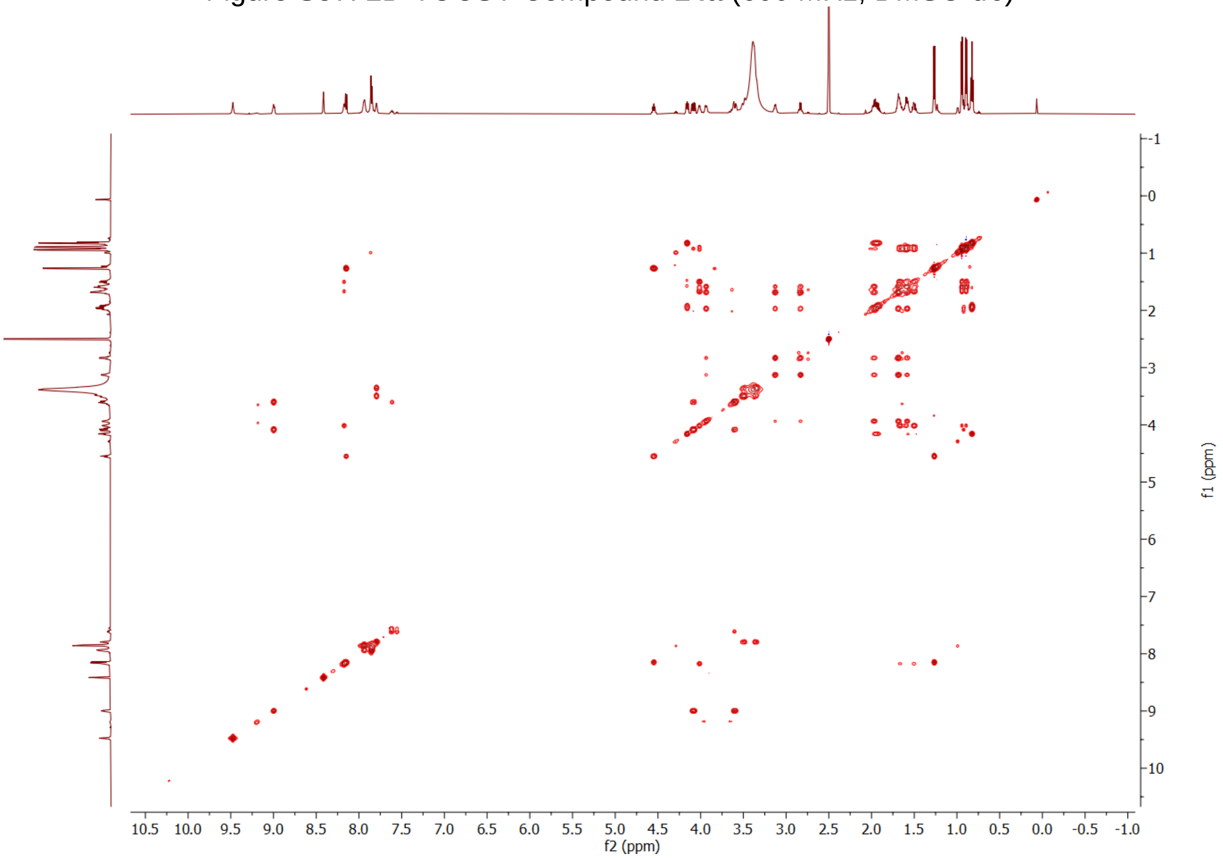

Figure S58: 2D-ROESY Compound **14a** (600 MHz, DMSO-*d*6)

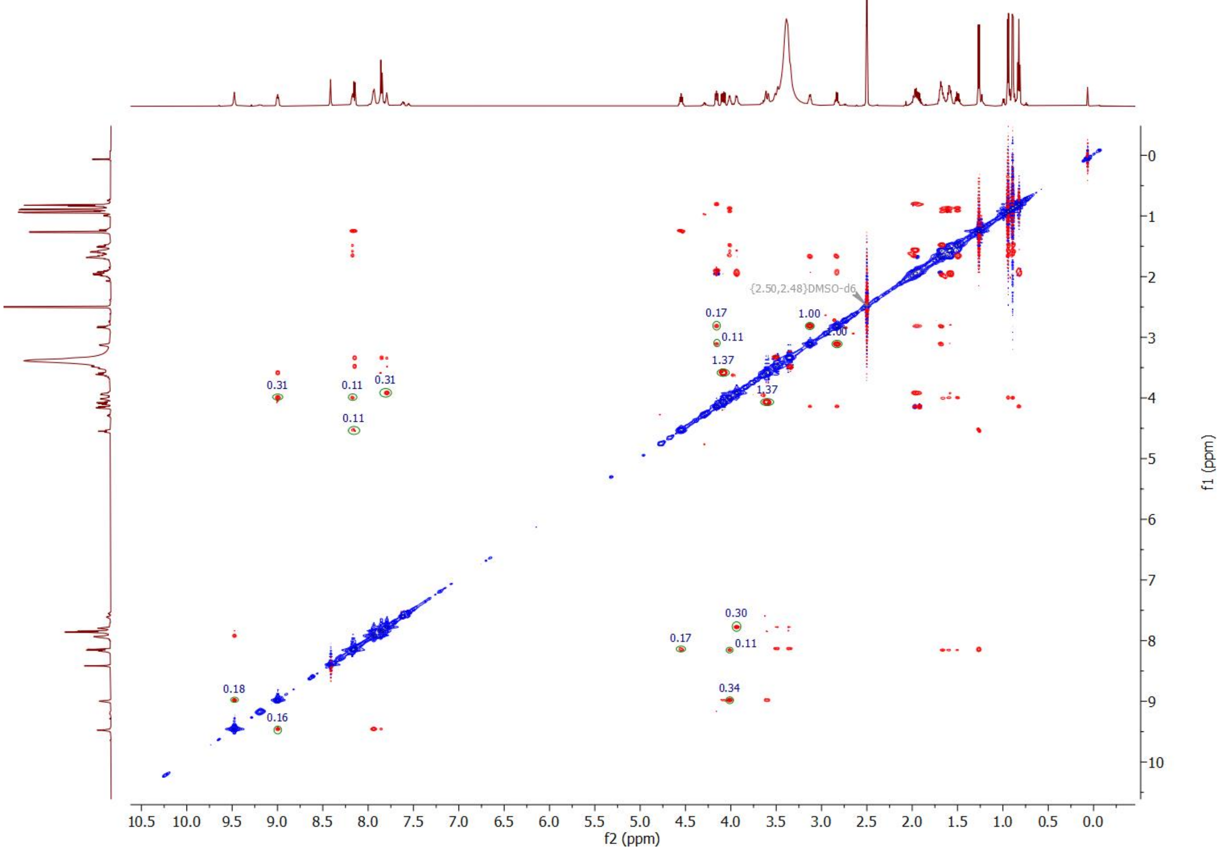

Figure S59: 2D-HSQC Compound **14a** (600 MHz, DMSO-*d*6)

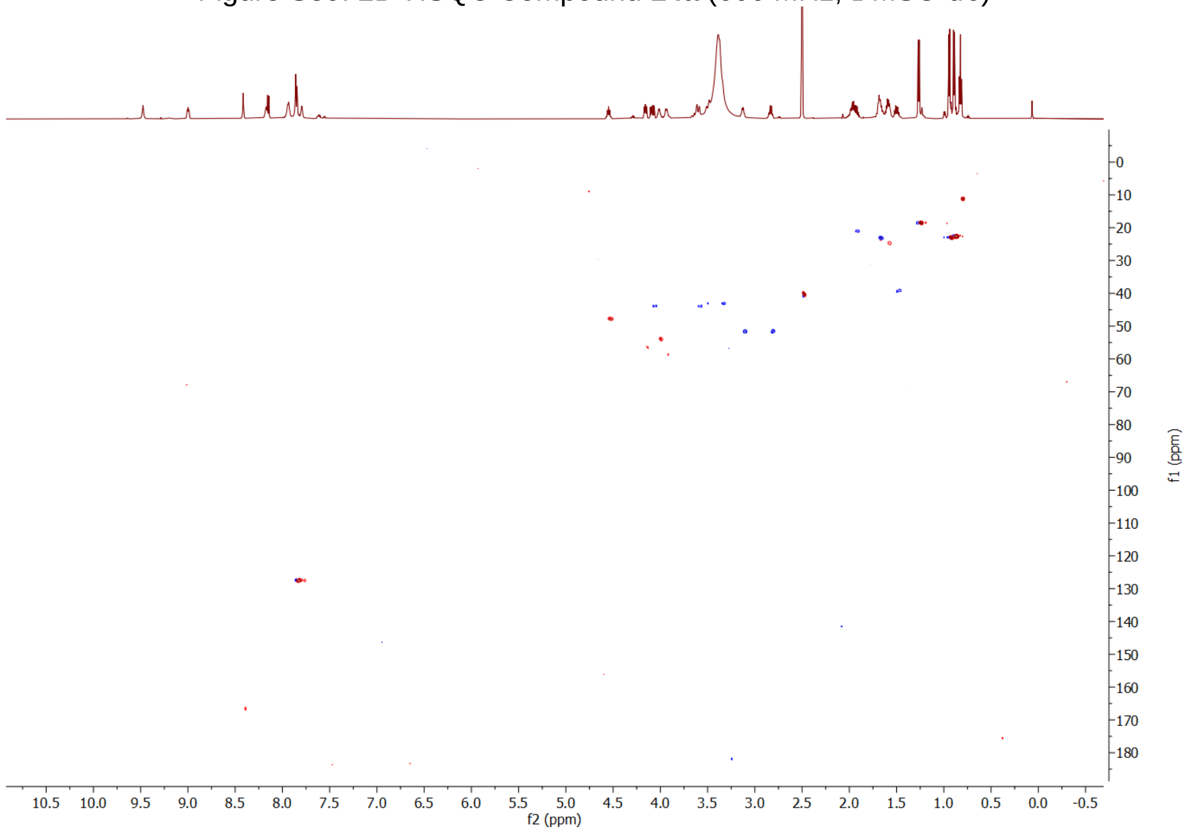

Figure S61: VT 1H-NMR Compound **14a** (600 MHz, DMSO-*d*6)

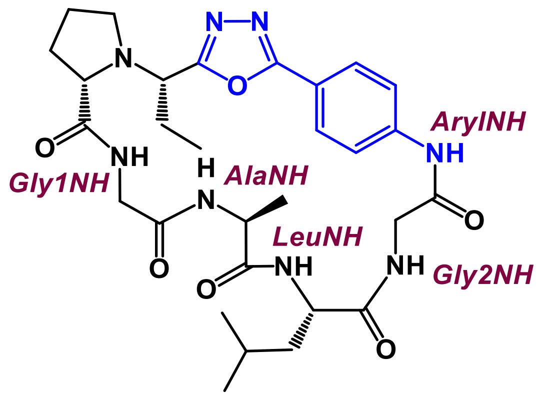

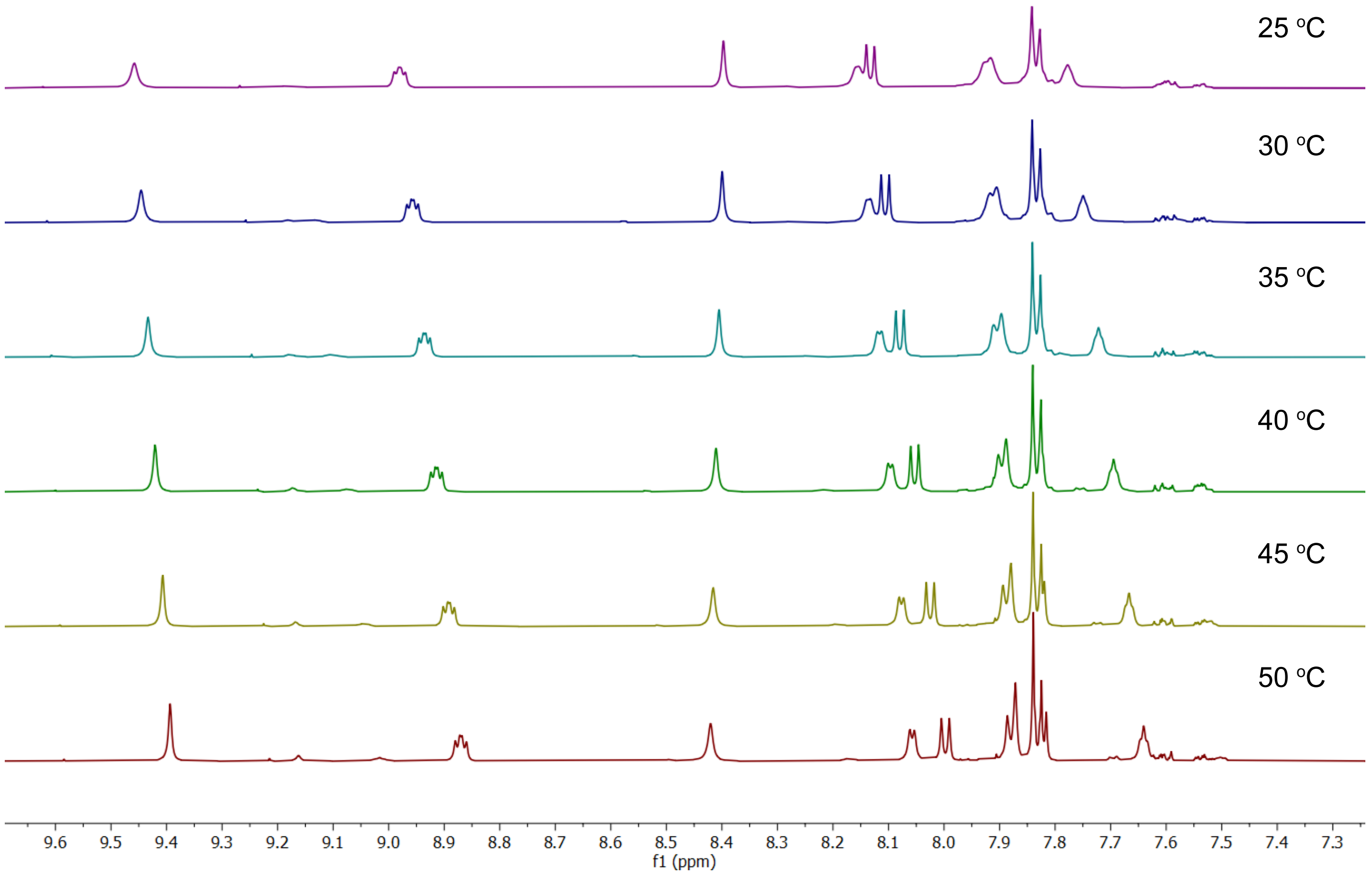

	Shifts (ppm)				
K	*ArylNH*	*Gly2NH*	*LeuNH*	*AlaNH*	*Gly1NH*
**298**	9.48	9.00	8.17	8.15	7.80
**323**	9.41	8.89	8.07	8.01	7.66
Δppb/k					
**298-323**	**2.8**	**4.4**	**4.0**	**5.6**	**5.6**

Table 8: Full Characterization of compound **16a**

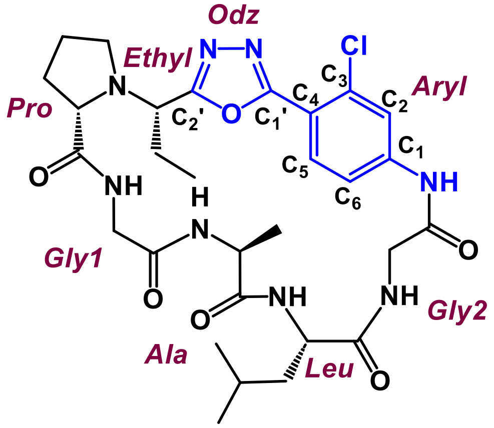

White solid obtained in a 17 % overall yield (based on 100 % resin loading).^{1}H NMR (500 MHz, DMSO-*d*6) δ 9.58 (s, 1H), 8.93 (s, 1H), 8.40 (s, 1H), 8.20 (s, 1H), 8.11 (d, *J* = 8.4 Hz, 1H), 7.86 – 7.78 (m, 2H), 7.62 (dd, *J* = 8.8, 2.1 Hz, 1H), 4.54 – 4.47 (m, 1H), 4.20 (dd, *J* = 9.1, 6.3 Hz, 1H), 4.11 (dd, *J* = 17.0, 7.6 Hz, 1H), 4.00 (d, *J* = 4.8 Hz, 1H), 3.77 (dd, *J* = 8.4, 3.8 Hz, 1H), 3.64 (dd, *J* = 17.0, 4.6 Hz, 1H), 3.57 (d, *J* = 4.0 Hz, 1H), 3.54 – 3.46 (m, 1H), 3.15 – 3.07 (m, 1H), 2.75 (d, *J* = 8.0 Hz, 1H), 1.99 – 1.90 (m, 3H), 1.74 – 1.66 (m, 2H), 1.63 – 1.57 (m, 3H), 1.53 – 1.47 (m, 1H), 1.24 (d, *J* = 7.2 Hz, 3H), 0.96 (d, *J* = 6.9 Hz, 3H), 0.93 (d, *J* = 6.9 Hz, 3H), 0.91 – 0.85 (m, 6H). HRMS (ESI+) m/z calc. for $C_{34}H_{48}N_7O_8^+$ [M+H]$^+$ = 631.2746, found 631.2754.

	1H Shifts
Ethyl	CH_3 (0.91 – 0.85), CH_2 (1.99 – 1.90), αCH (4.20)
Gly2	αCH_2 (3.64, 4.11), NH (8.93)
Leu	αCH (4.00), βCH_2 (1.53 – 1.47, 1.63 – 1.57), γCH (1.63 – 1.57), CH_3s (0.96, 0.93) NH (8.20)
Ala	αCH (4.54 – 4.47), CH_3 (0.91 – 0.85), NH (8.11)
Gly1	αCH_2 (3.54 – 3.46, 3.57), NH (7.86 – 7.78)
Pro	αCH (3.77), βCH_2 (1.99 – 1.90, 1.63 – 1.57), γCH_2 (1.74 – 1.66), δCH_2 (2.75, 3.15 – 3.07)
Aryl	NH (9.58), C_2H (8.40), C_5H (7.86 – 7.78), C_6H (7.62)
Odz	N/A

	C13 Shifts
Ethyl	CH_3 (11.0), CH_2 (21.7), αCH (43.8)
Gly2	αC (44.1), C=O (172.3)
Leu	αC (53.9), βC (39.2), γC (24.6), CH_3s (22.9, 22.4), C=O (172.4)
Ala	αC (47.7), CH_3 (18.5), C=O (172.6)
Gly1	αC (42.9), C=O (167.3)
Pro	αC (59.1), βC (31.7), γC (22.9), δC (50.6), C=O (174.0)
Aryl	C_1 (n.d), C_2 (119.7), C_3 (n.d), C_4 (141.2), C_5 (130.5), C_6 (118.7). C=O (168.8)
Odz	C_1' (160.9), C_2' (164.2)

Figure S62:1H-NMR **16a** (500 MHz, DMSO-*d*6)

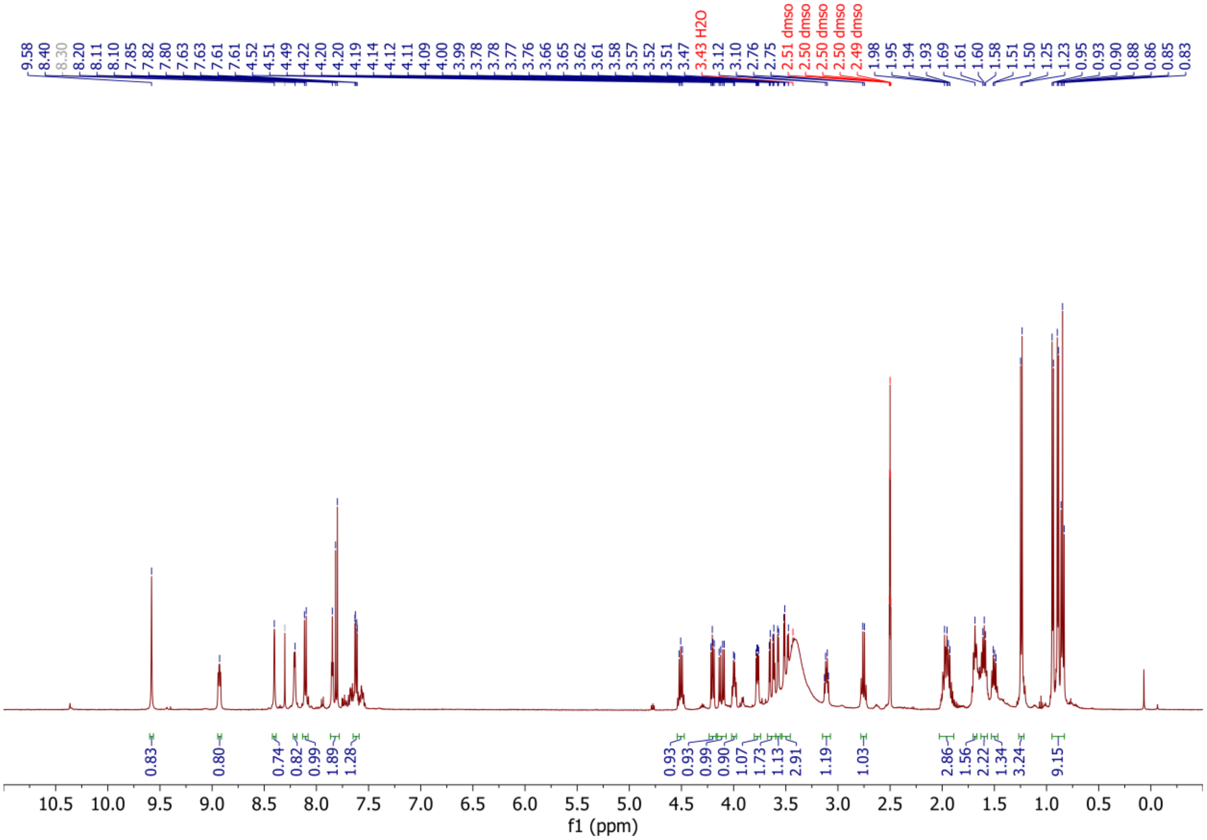

Figure S63: 2D-COSY Compound **16a** (500 MHz, DMSO-*d*6)

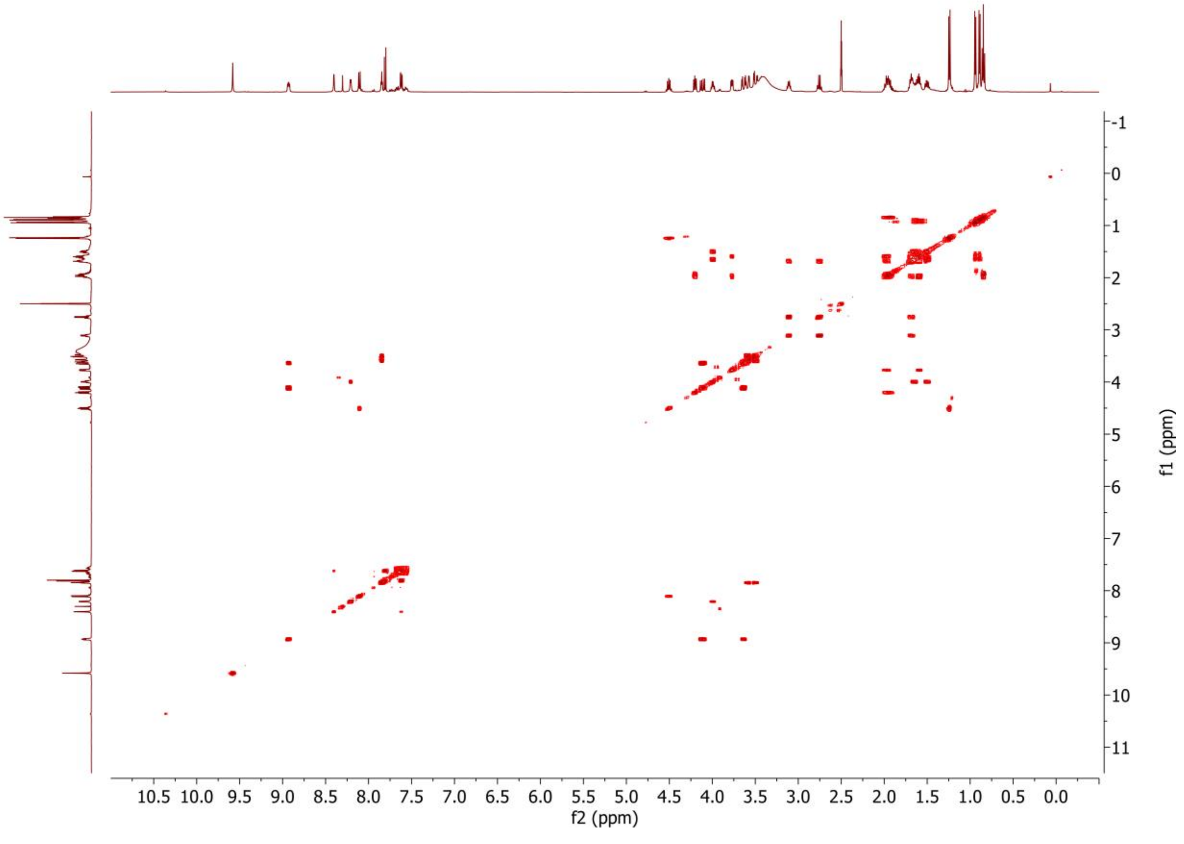

Figure S64: 2D-TOCSY Compound **16a** (500 MHz, DMSO-*d*6)

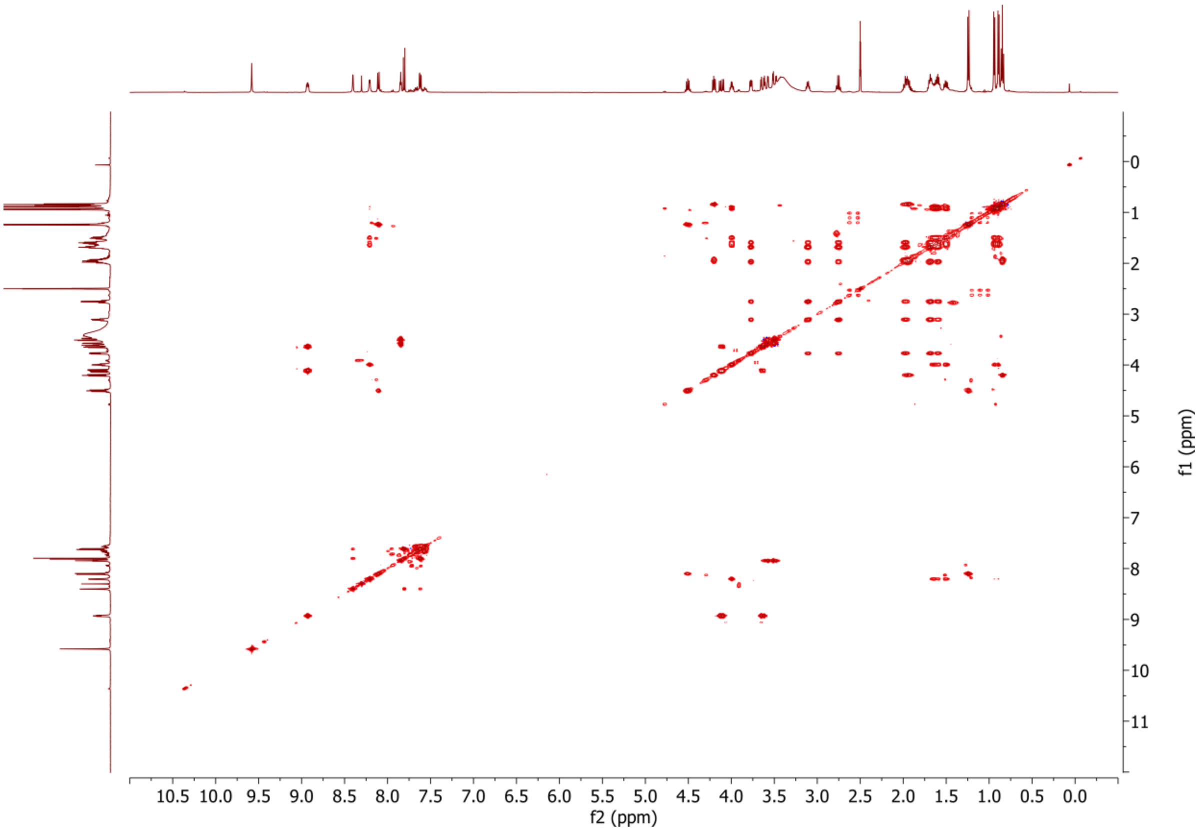

Figure S65: 2D-ROESY Compound **16a** (500 MHz, DMSO-*d*6)

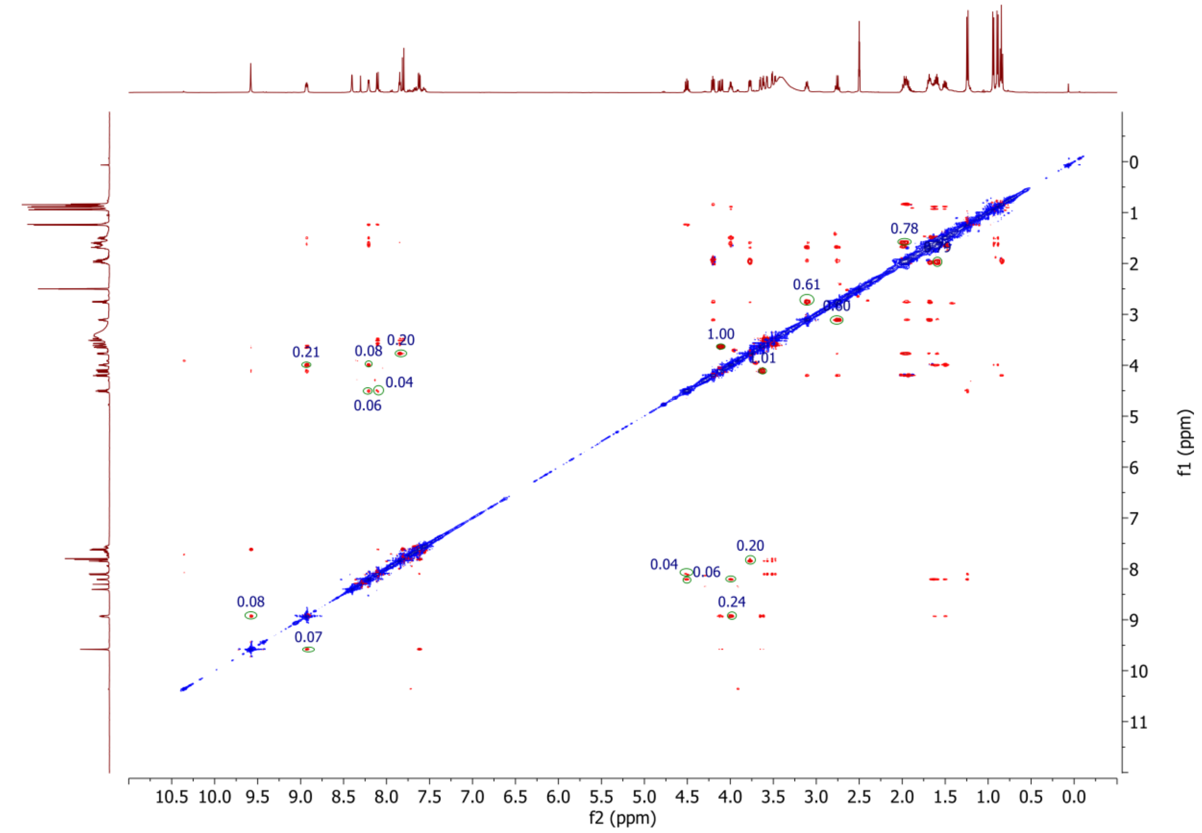

Figure S66: 2D-HSQC Compound **16a** (500 MHz, DMSO-*d*6)

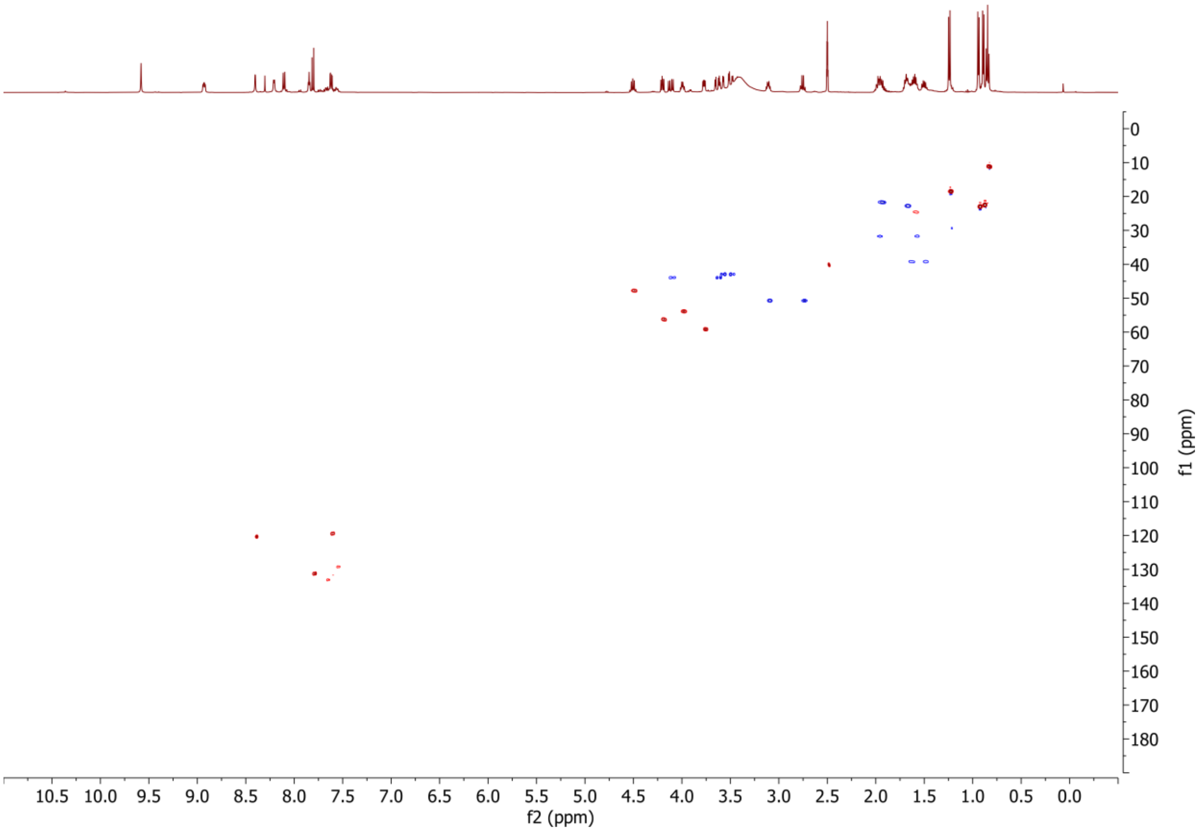

Figure S67: 2D-HMBC Compound **16a** (500 MHz, DMSO-*d*6)

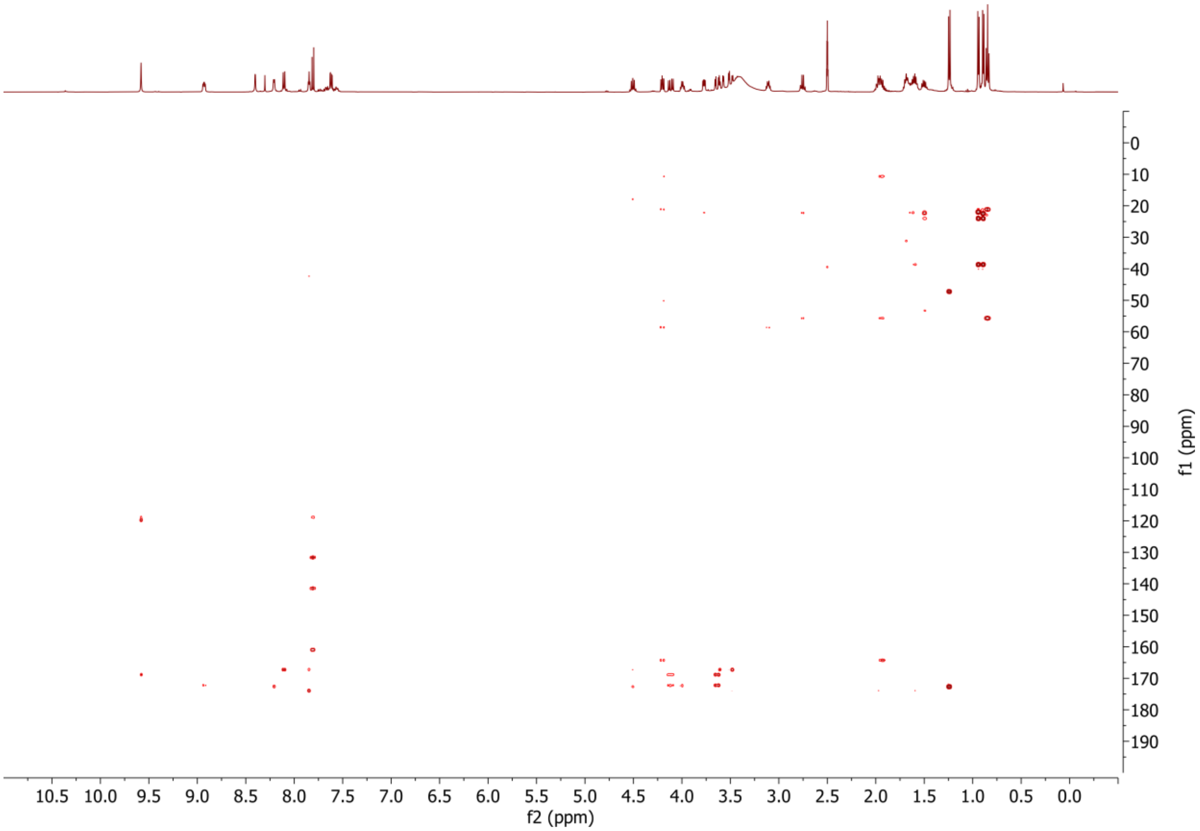

Figure S68: VT 1H-NMR Compound **16a** (500 MHz, DMSO-*d6*)

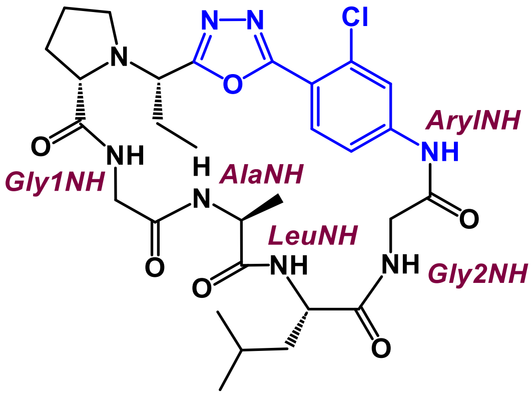

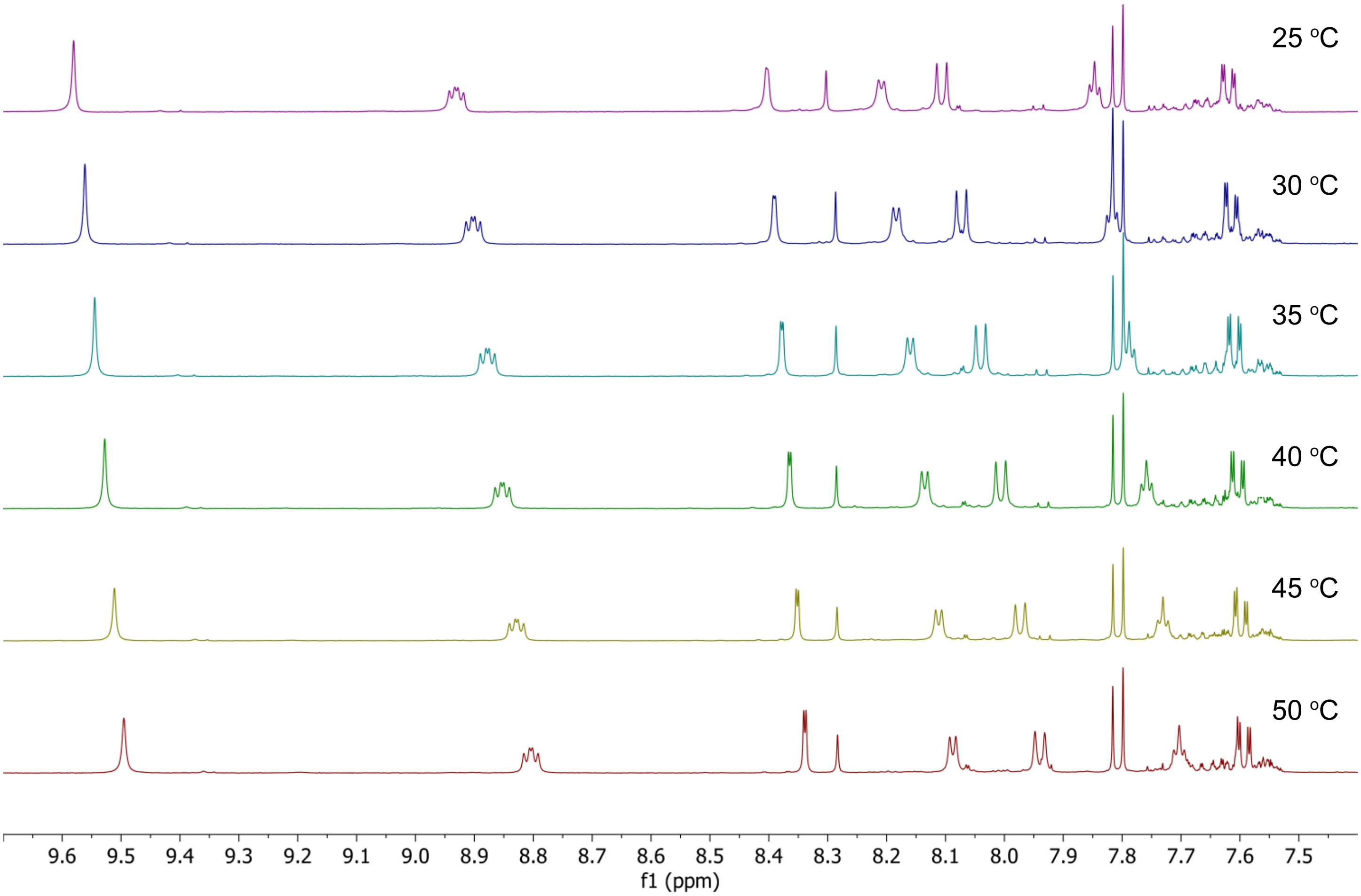

	Shifts (ppm)				
K	*ArylNH*	*Gly1NH*	*LeuNH*	*AlaNH*	*Gly2NH*
**298**	9.58	8.93	8.21	8.11	7.85
**323**	9.49	8.8	8.09	7.94	7.7
Δppb/k					
**298-323**	**3.6**	**5.2**	**4.8**	**6.8**	**6**

Table 9: Full Characterization of compound **17a**

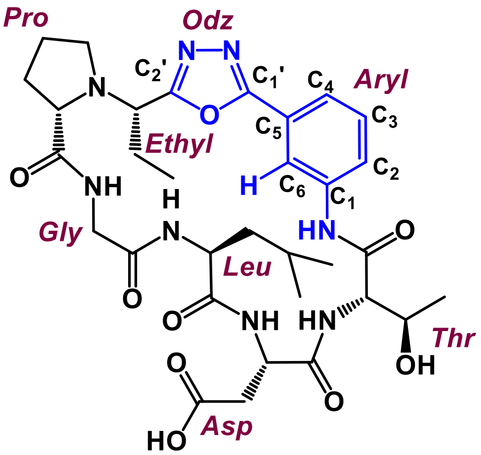

White solid obtained in a 27 % overall yield (based on 100 % resin loading). ^{1}H NMR (500 MHz, DMSO-*d*6) δ 9.88 (s, 1H), 8.72 (d, *J* = 6.3 Hz, 1H), 8.54 (s, 1H), 8.20 – 8.14 (m, 1H), 7.83 (d, *J* = 7.7 Hz, 1H), 7.65 (d, *J* = 8.4 Hz, 1H), 7.55 (t, *J* = 7.9 Hz, 1H), 7.31 (d, *J* = 8.3 Hz, 1H), 5.11 (s, 1H), 4.45 (q, *J* = 8.5 Hz, 1H), 4.41 – 4.33 (m, 2H), 4.22 – 4.10 (m, 2H), 3.93 (dd, *J* = 9.9, 5.7 Hz, 1H), 3.71 (dd, *J* = 16.1, 4.5 Hz, 1H), 3.25 – 3.19 (m, 1H), 2.90 (d, *J* = 3.9 Hz, 1H), 2.79 – 2.67 (m, 2H), 2.67 – 2.61 (m, 1H), 2.09 – 1.88 (m, 2H), 1.70 – 1.54 (m, 6H), 1.09 (d, *J* = 6.3 Hz, 3H), 0.88 (d, *J* = 6.2 Hz), 0.84 (d, *J* = 6.2 Hz), 0.78 (t, *J* = 7.3 Hz, 3H). HRMS (ESI+) m/z calc. for $C_{32}H_{45}N_8O_9^+$ $[M+H]^+$ = 685.3297, found 685.3304.

	1H Shifts
Ethyl	CH_3 (0.78), CH_2 (2.09 – 1.88), αCH (3.93)
Thr	αCH (4.41 – 4.33), βCH (4.22 – 4.10), CH_3 (1.09), NH (7.31), OH (5.11)
Asp	αCH (4.41 – 4.33), $βCH_2$ (2.79 – 2.67), NH (8.72), CO_2H (n.d)
Leu	αCH (4.45), $βCH_2$ (1.70 – 1.54), γCH (1.70 – 1.54) CH_3s (0.84, 0.88), NH (7.65)
Gly	$αCH_2$ (3.71, 4.22 – 4.10), NH (8.20 – 8.14)
Pro	αCH (3.25 – 3.19), $βCH_2$ (1.70 – 1.54, 1.97), $γCH_2$ (1.70 – 1.54), $δCH_2$ (2.67 – 2.61, 2.90)
Aryl	NH (9.88), C_2H (7.65), C_3H (7.55), C_4H (7.83), C_6H (8.54)
Odz	N/A

	C13 Shifts
Ethyl	CH_3 (10.5), CH_2 (24.7), αCH (56.0)
Thr	αC (58.0), βC (66.4), CH_3 (19.7), C=O (170.4)
Asp	αC (51.4), βC (35.9), γC=O (170.9), C=O (170.7)
Leu	αC (51.0), βC (22.4), γC (41.2), CH_3s (21.6, 22.5), C=O (173.0)
Gly	αC (42.0), C=O (173.7)
Pro	αC (62.7), βC (29.6), γC (24.1), δC (48.7), C=O (173.7)
Aryl	C_1 (139.2), C_2 (122.0), C_3 (129.7), C_4 (121.1), C_5 (117.6), C_6 (123.5). C=O (168.8)
Odz	C_1' (163.8), C_2' (165.8)

Figure S69:1H-NMR **17a** (500 MHz, DMSO-*d*6)

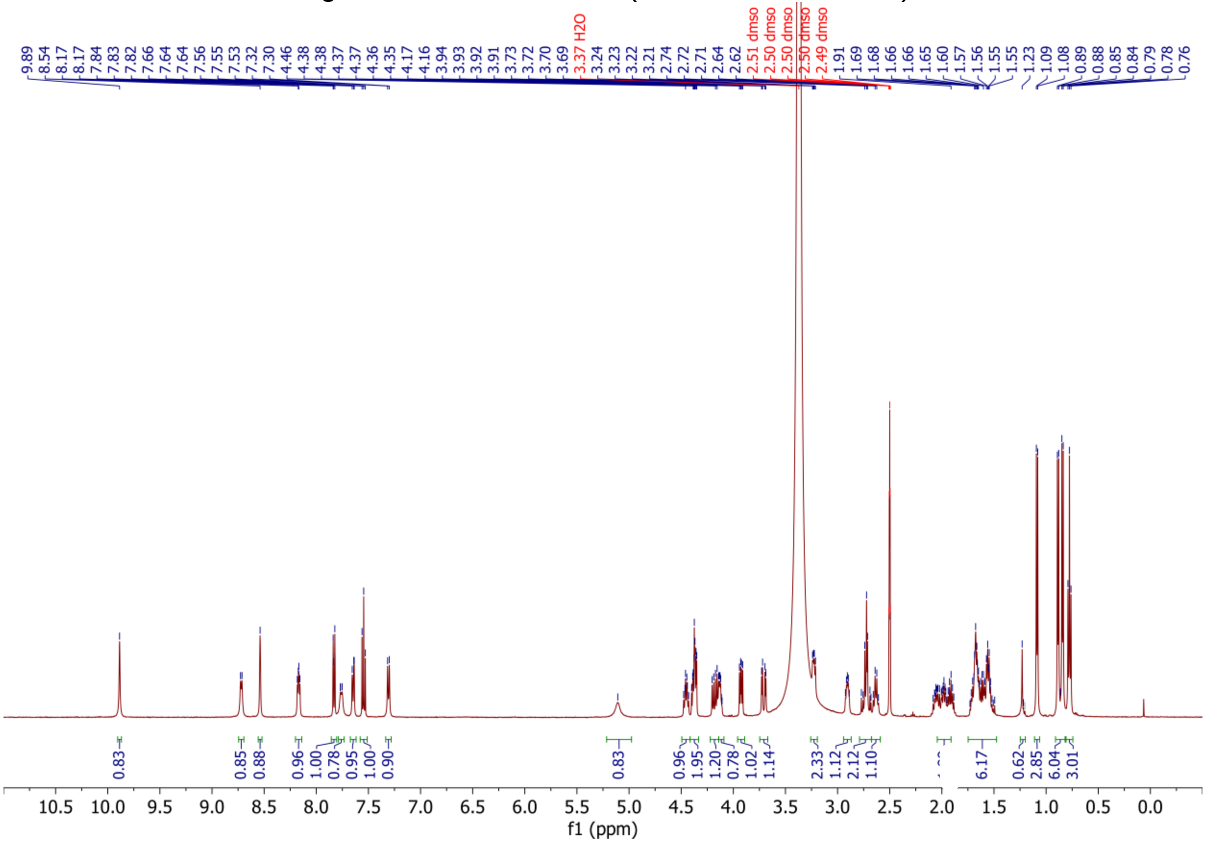

Figure S70: 2D-COSY Compound **17a** (500 MHz, DMSO-*d*6)

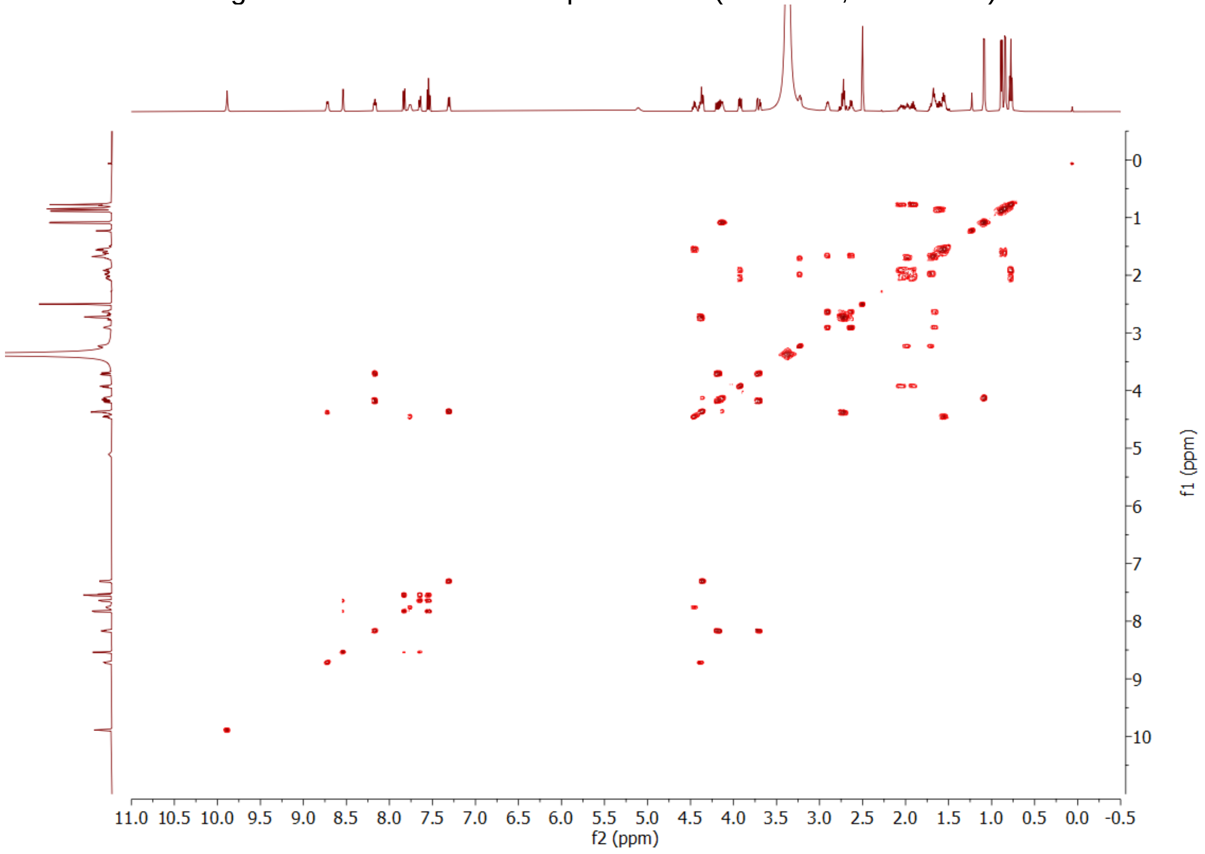

Figure S71: 2D-TOCSY Compound **17a** (500 MHz, DMSO-*d*6)

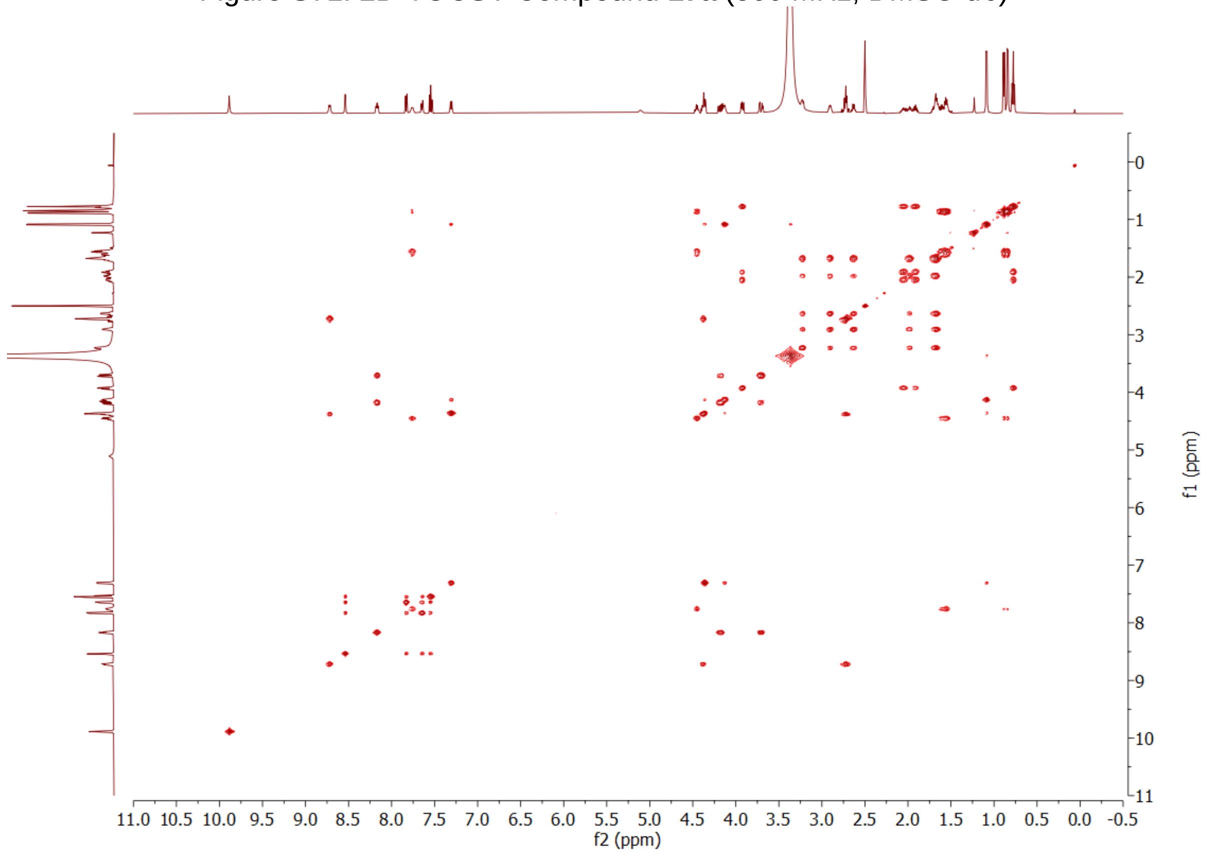

Figure S72: 2D-ROESY Compound **17a** (500 MHz, DMSO-*d*6)

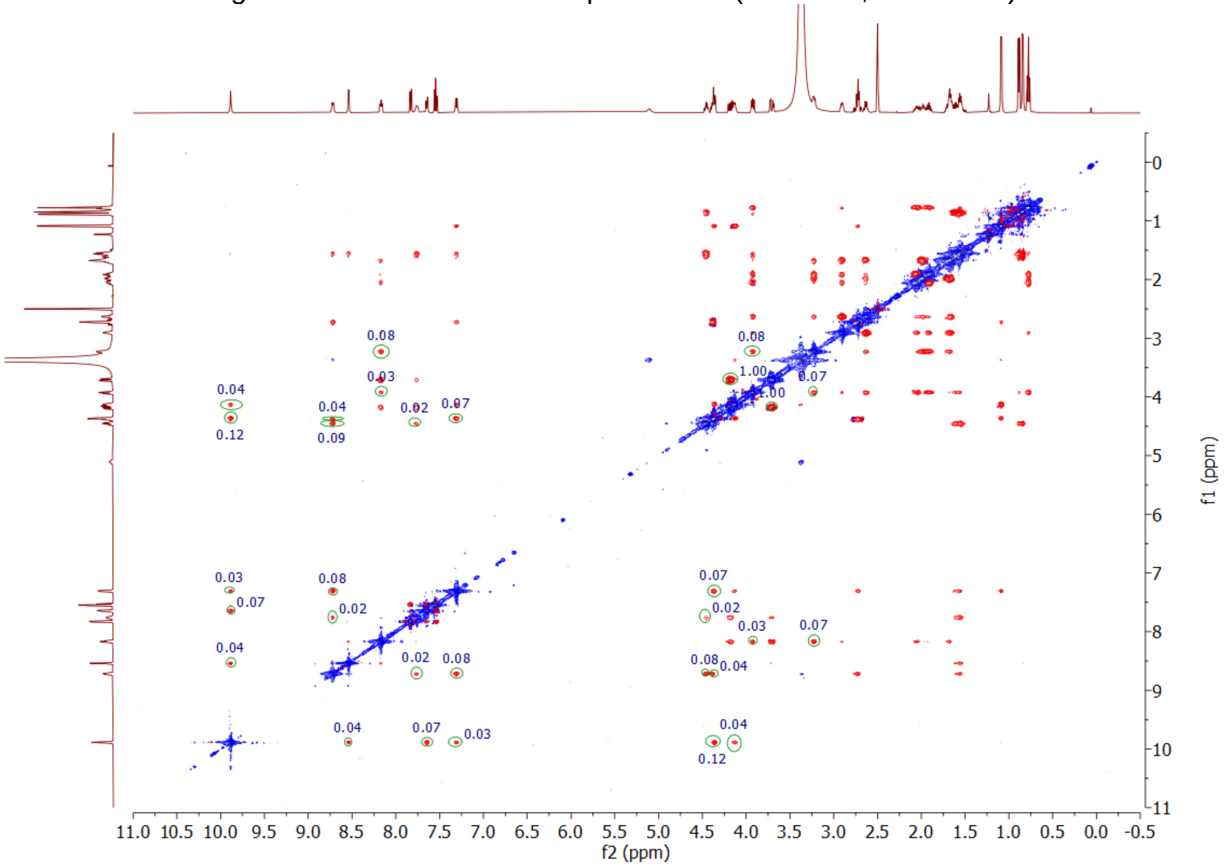

Figure S73: 2D-HSQC Compound **17a** (500 MHz, DMSO-*d*6)

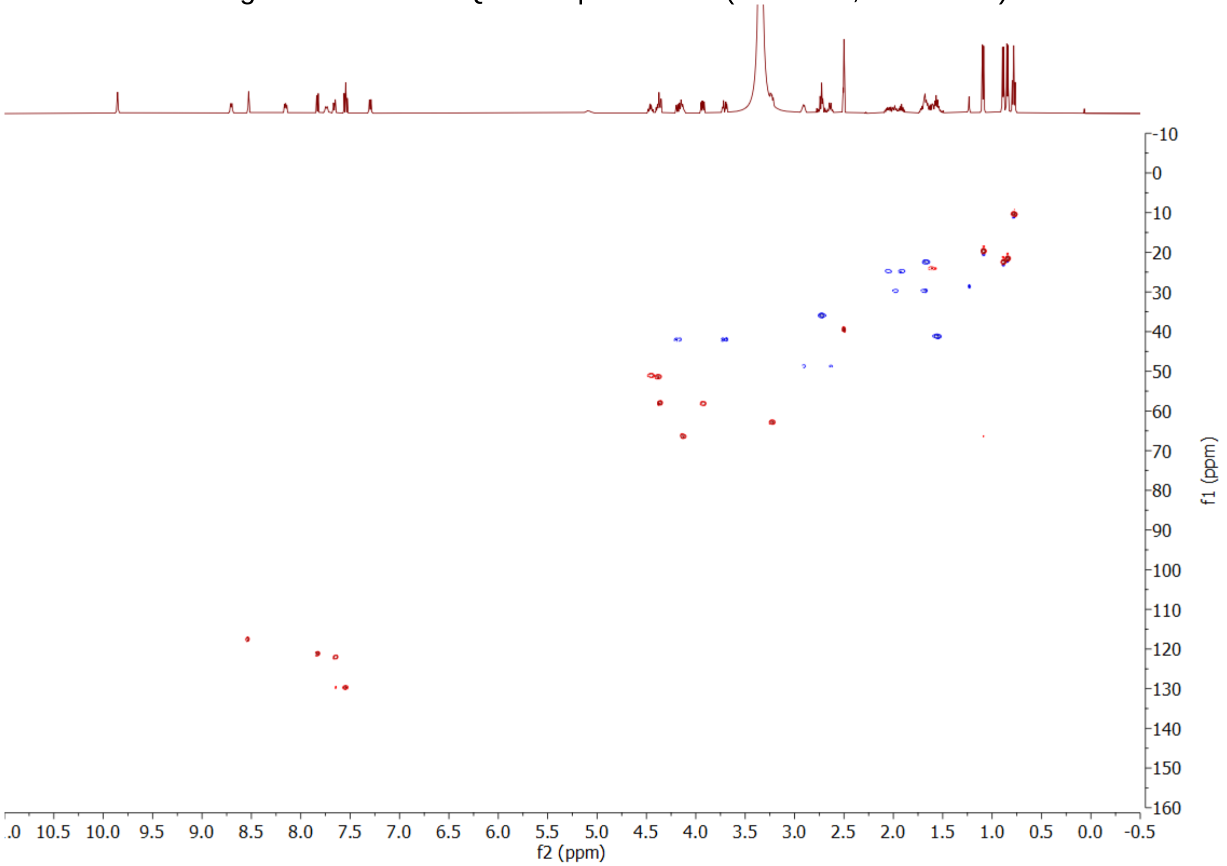

Figure S74: 2D-HMBC Compound **17a** (500 MHz, DMSO-*d*6)

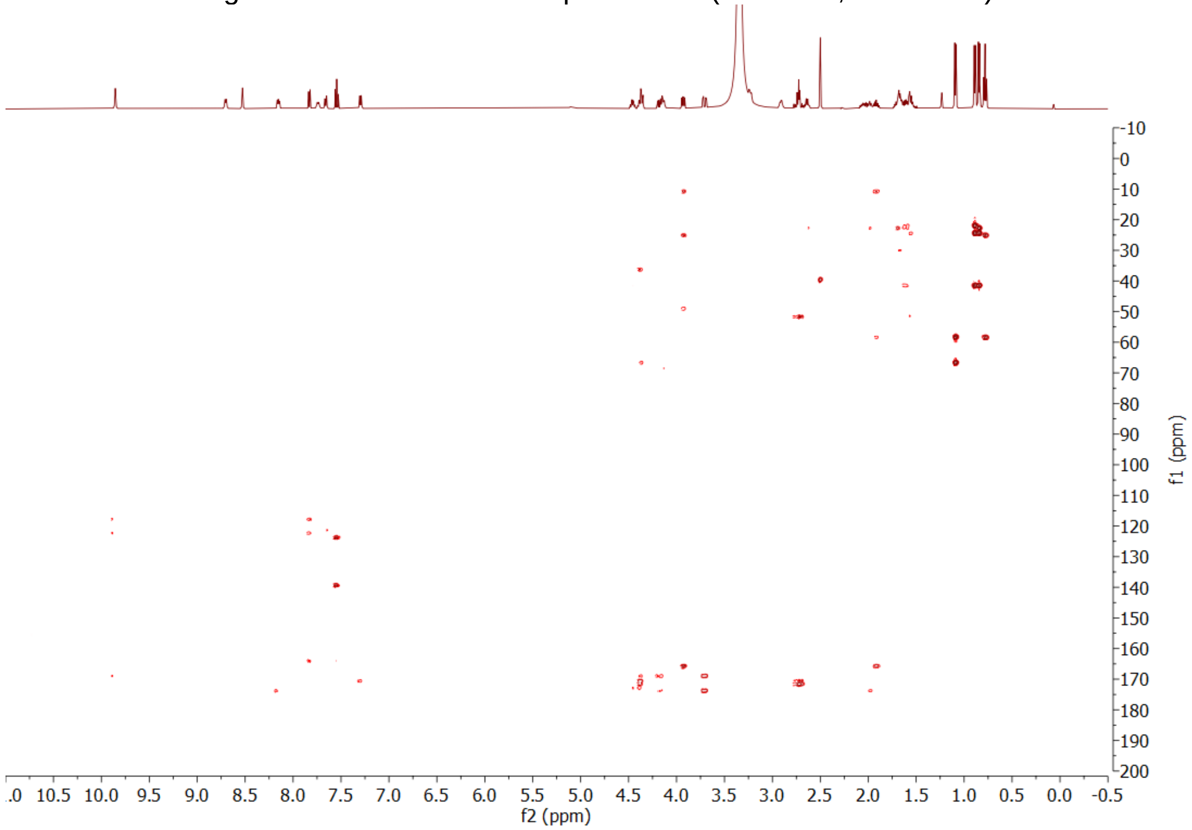

Figure S75: VT 1H-NMR Compound **17a** (500 MHz, DMSO-*d*6)

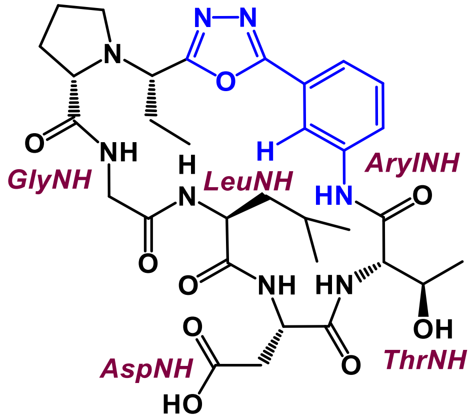

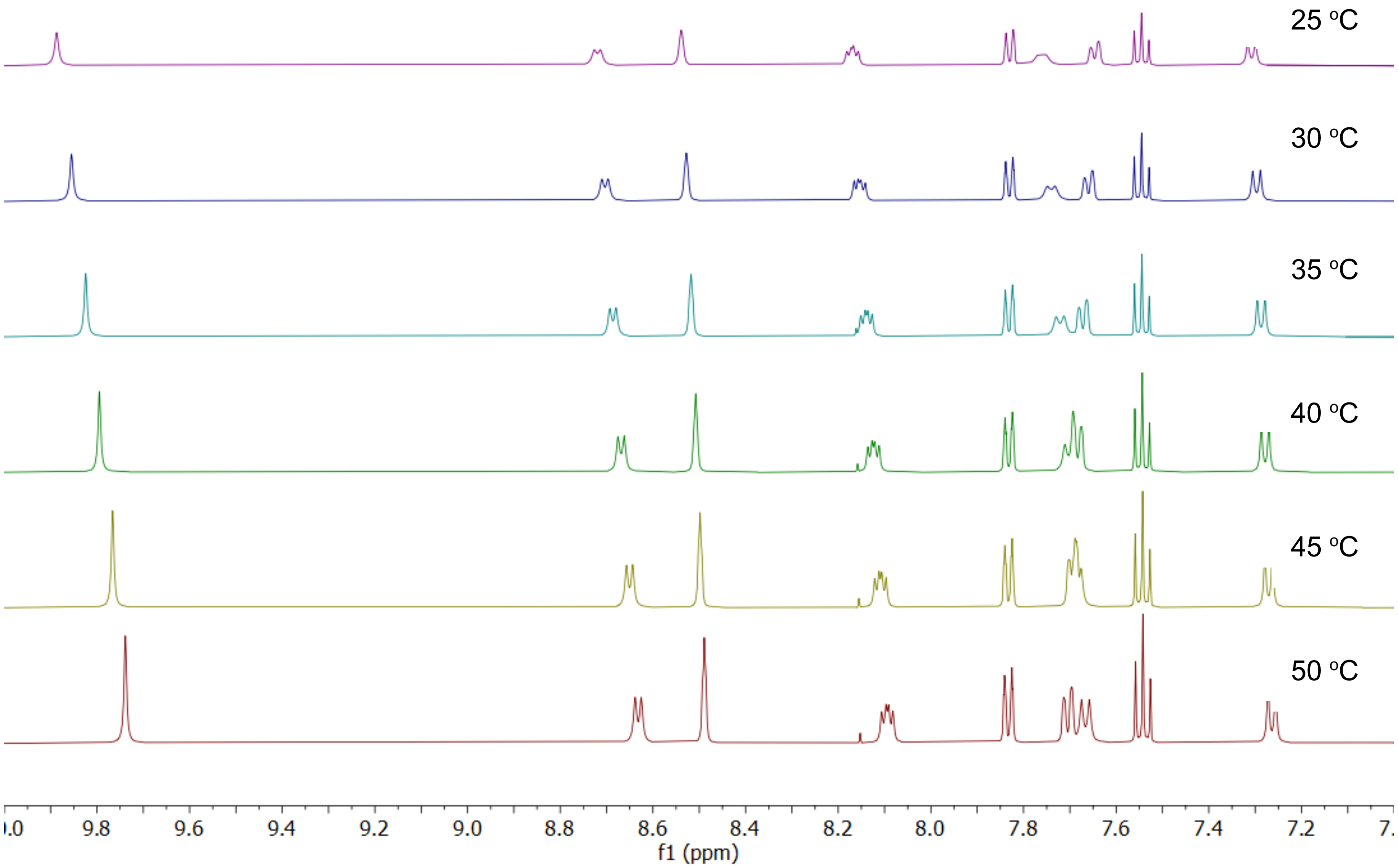

K	Shifts (ppm) *ArylNH*	*Thr*	*Asp*	*Leu*	*Gly*
**298**	9.88	7.31	8.72	7.76	8.17
**323**	9.76	7.26	8.63	7.67	8.10
Δppb/k					
**298-323**	**4.8**	**2**	**3.6**	**3.6**	**2.8**

Table 10: Full Characterization of compound **17b**

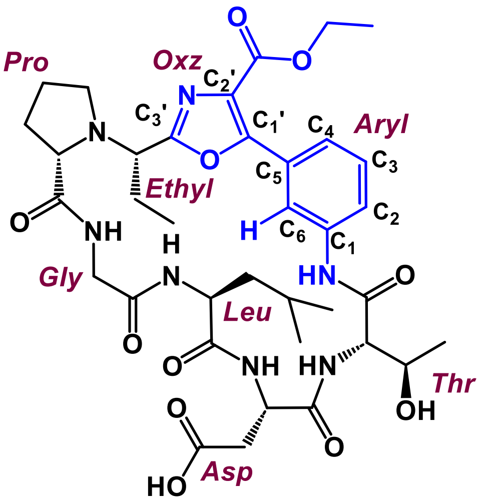

White solid obtained in a 21 % overall yield (based on 100 % resin loading).^{1}H NMR (500 MHz, DMSO-*d*6) δ 9.71 (s, 1H), 8.81 (s, 1H), 8.34 (s, 1H), 8.10 (d, *J* = 7.8 Hz, 2H), 7.64 (d, *J* = 7.7 Hz, 1H), 7.46 (t, *J* = 8.0 Hz, 1H), 7.22 (s, 1H), 5.13 (s, 1H), 4.39 – 4.32 (m, 5H), 4.14 – 4.08 (m, 2H), 3.74 (dd, *J* = 9.7, 5.7 Hz, 1H), 3.64 (dd, *J* = 15.7, 4.4 Hz, 1H), 2.86 – 2.80 (m, 1H), 2.70 – 2.62 (m, 3H), 2.04 – 1.93 (m, 2H), 1.88 – 1.79 (m, 1H), 1.74 – 1.52 (m, 6H), 1.30 (t, *J* = 7.1 Hz, 3H), 1.09 (d, *J* = 6.4 Hz, 3H), 0.88 (d, *J* = 6.4 Hz, 3H), 0.83 (d, *J* = 6.4 Hz, 3H), 0.77 (t, *J* = 7.3 Hz, 3H). HRMS (ESI+) m/z calc. for $C_{36}H_{50}N_7O_{11}^+$ $[M+H]^+$ = 756.3560, found 756.3563.

	**1H Shifts**
Ethyl	CH_3 (0.77), CH_2 (1.88 – 1.79, 2.04 – 1.93), αCH (3.72)
Thr	αCH (4.39 – 4.32), βCH (4.14 – 4.08), CH_3 (1.09), NH (7.23), OH (5.14)
Asp	αCH (4.39 – 4.32), $βCH_2$ (2.70 – 2.62), NH (8.81), CO_2H (n.d)
Leu	αCH (4.39 – 4.32), $βCH_2$ (1.74 – 1.52), γCH (1.74 – 1.52) CH_3s (0.83, 0.88), NH (7.97)
Gly	$αCH_2$ (3.63, 4.14 – 4.08), NH (8.11)
Pro	αCH (3.33), $βCH_2$ (2.04 – 1.93, 1.74 – 1.52), $γCH_2$ (1.74 – 1.52), $δCH_2$ (2.70 – 2.62, 2.86 – 2.80)
Aryl	NH (9.71), C_2H (7.63), C_3H (7.45), C_4H (8.09), C_6H (8.55)
Oxz	CH_2 (4.39 – 4.32), CH_3 (1.30)

	**C13 Shifts**
Ethyl	CH_3 (11.1), CH_2 (25.7), αCH (61.4)
Thr	αC (58.3), βC (67.0), CH_3 (20.5), C=O (168.9)
Asp	αC (52.0), βC (37.0), γC=O (171.9), C=O (170.6)
Leu	αC (58.9), βC (24.8), γC (24.5), CH_3s (22.2, 23.1), C=O (172.2)
Gly	αC (42.8), C=O (169.2)
Pro	αC (63.7), βC (30.6), γC (23.7), δC (50.3), C=O (172.1)
Aryl	C_1 (138.7), C_2 (121.7), C_3 (129.2), C_4 (124.2), C_5 (n.d), C_6 (118.8). C=O (n.d)
Oxz	C_1' (153.7), C_2' (n.d), C_3' (161.8), CH_2 (60.7), CH_3 (14.5), C=O (161.7)

**Signal at 3.33 ppm was determined by 2D NMR (Not observable in 1H due to overlapping signal with residual H_2O)

Figure S76:1H-NMR **17b** (500 MHz, DMSO-*d*6)

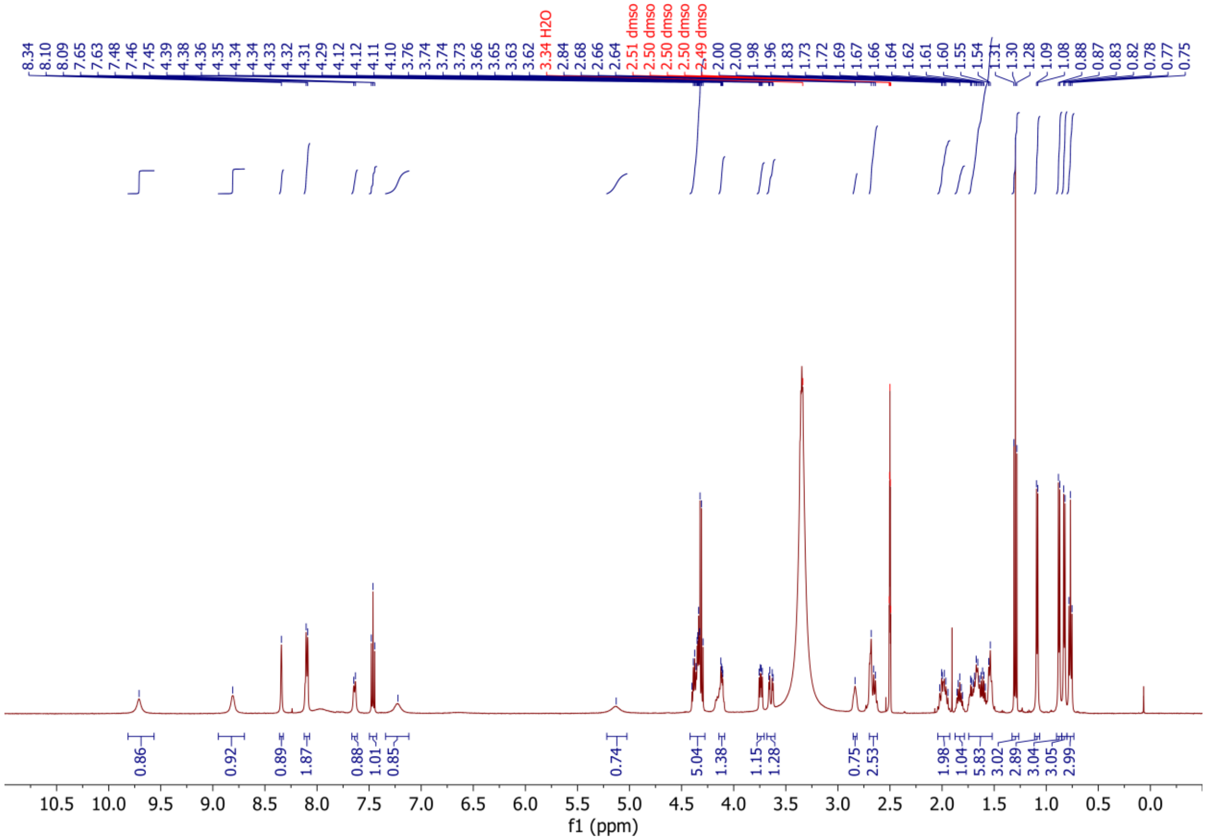

Figure S77: 2D-COSY Compound **17b** (500 MHz, DMSO-*d*6)

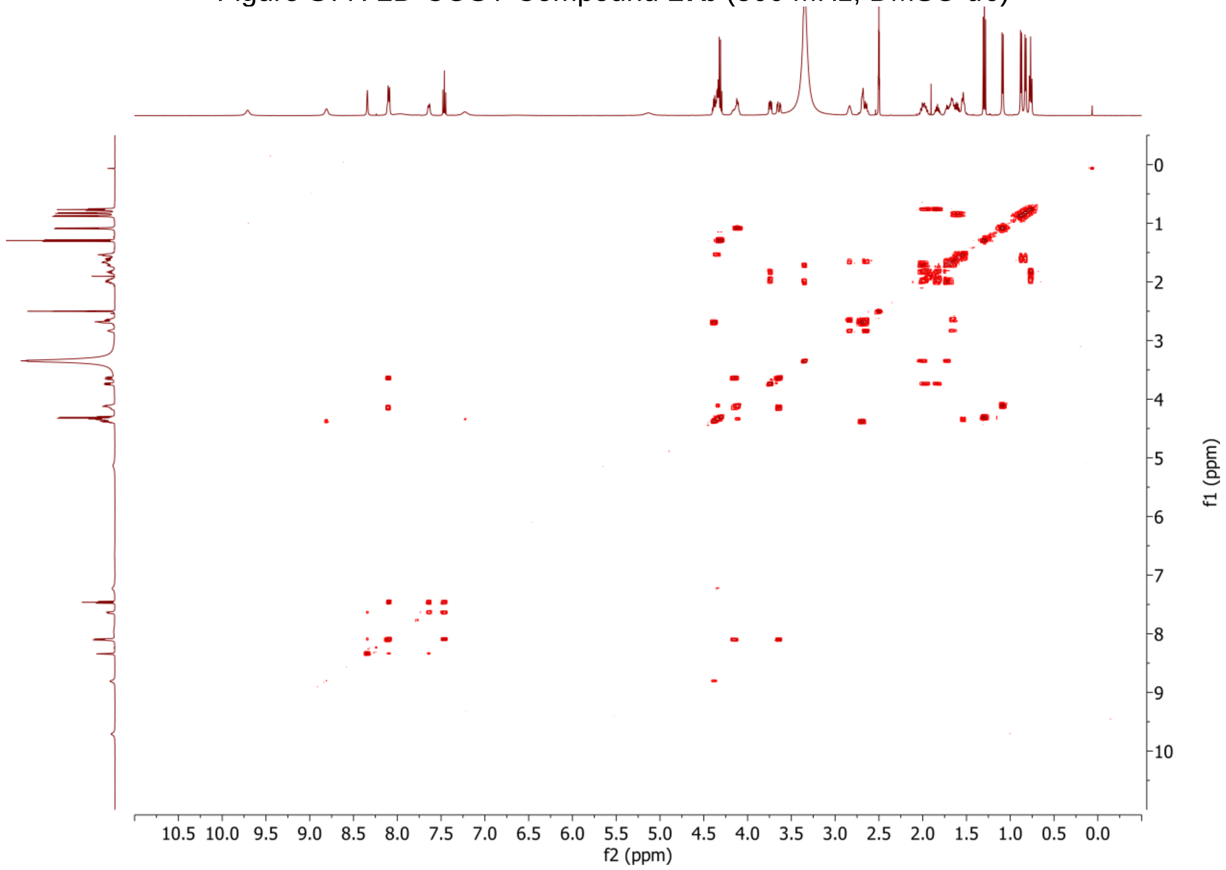

Figure S78: 2D-TOCSY Compound **17b** (500 MHz, DMSO-*d*6)

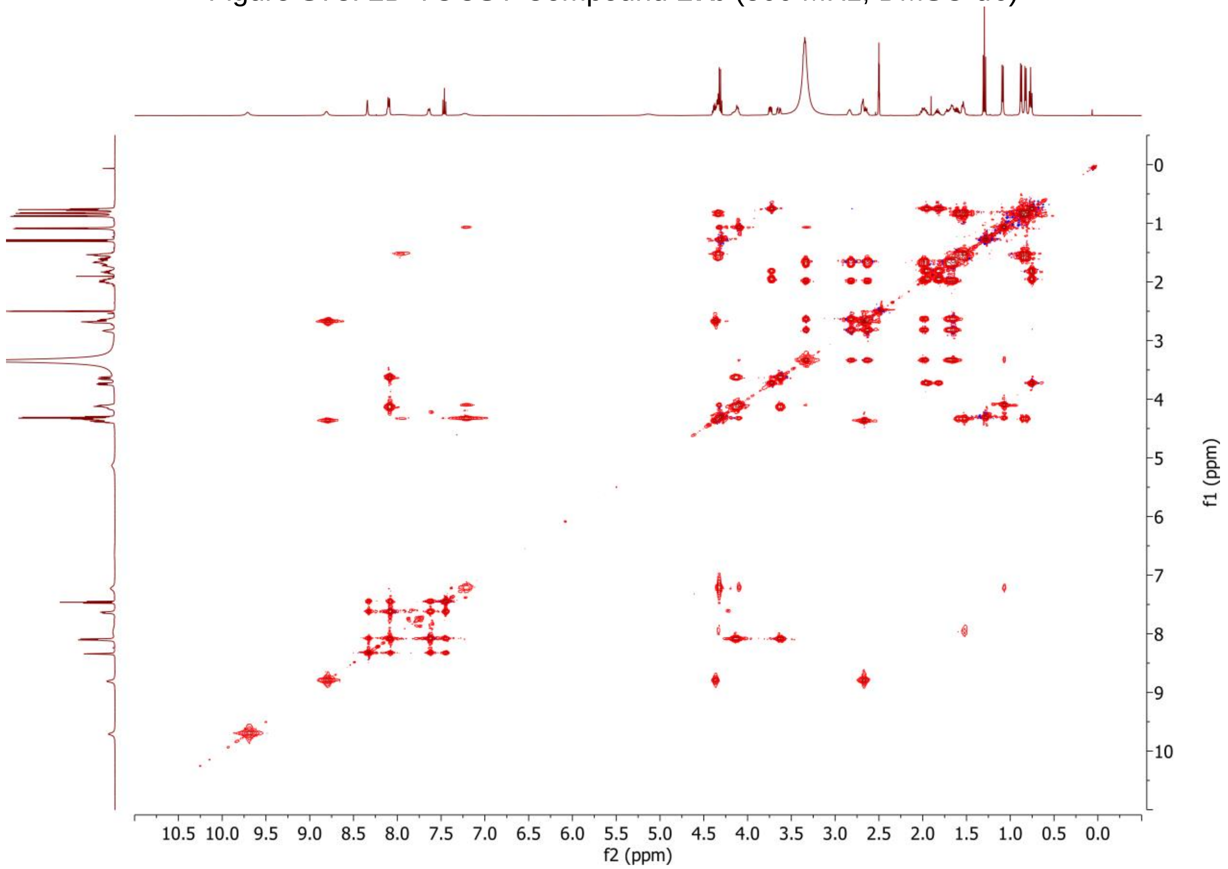

Figure S79: 2D-ROESY Compound **17b** (500 MHz, DMSO-*d*6)

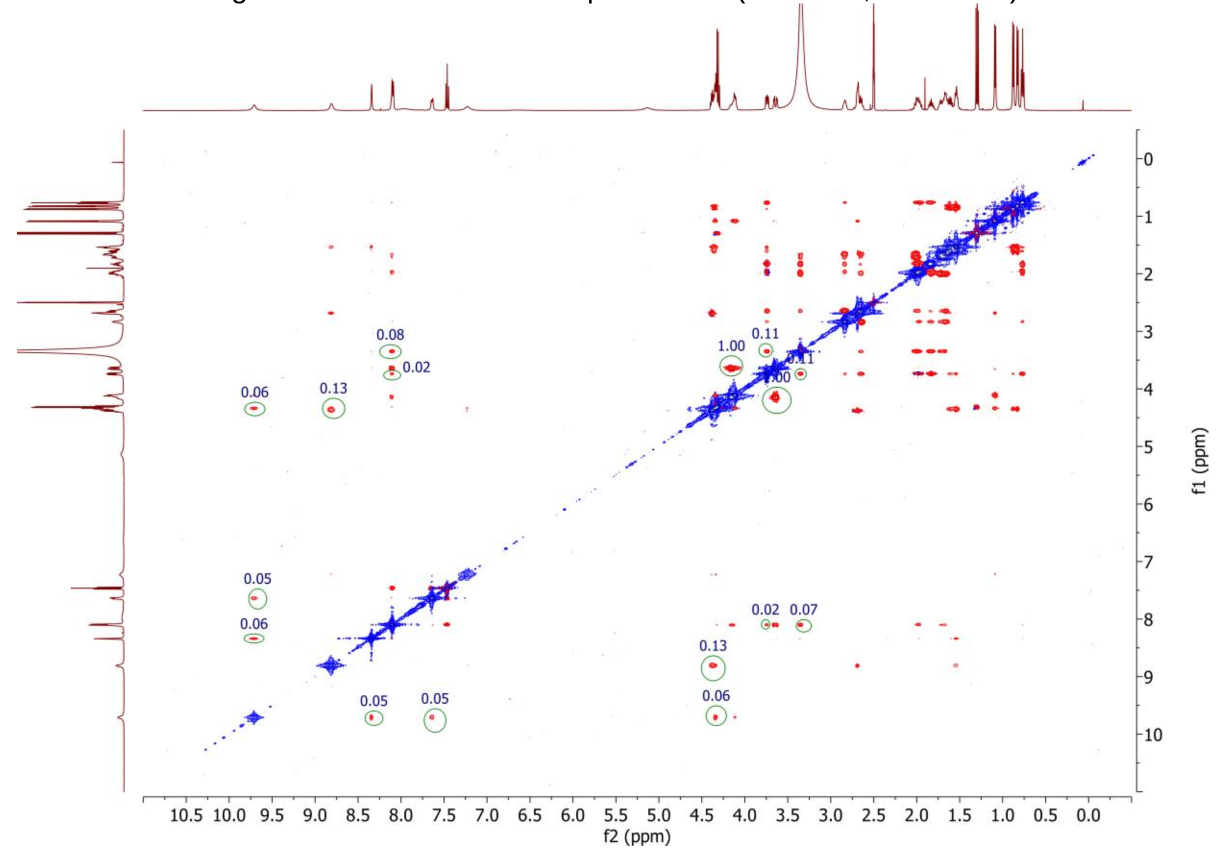

Figure S80: 2D-HSQC Compound **17b** (500 MHz, DMSO-*d*6)

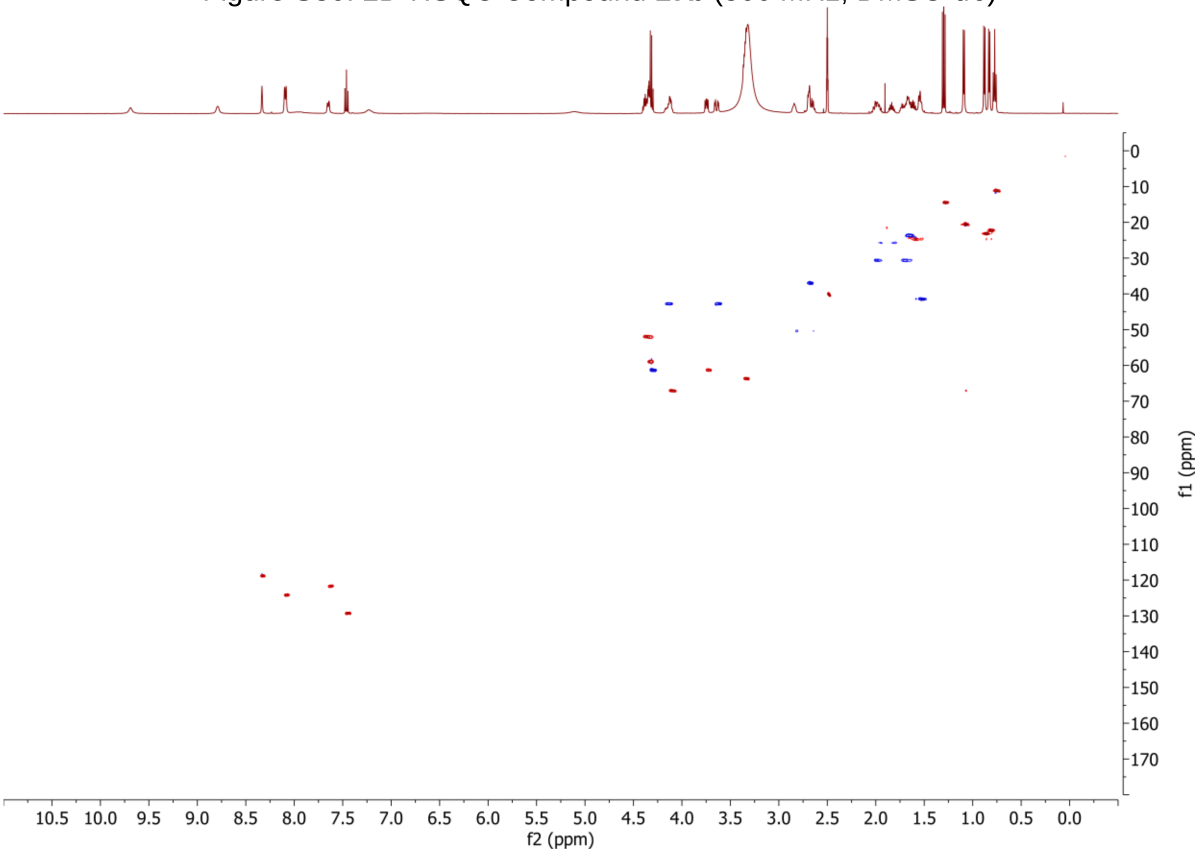

Figure S81: 2D-HMBC Compound **17b** (500 MHz, DMSO-*d*6)

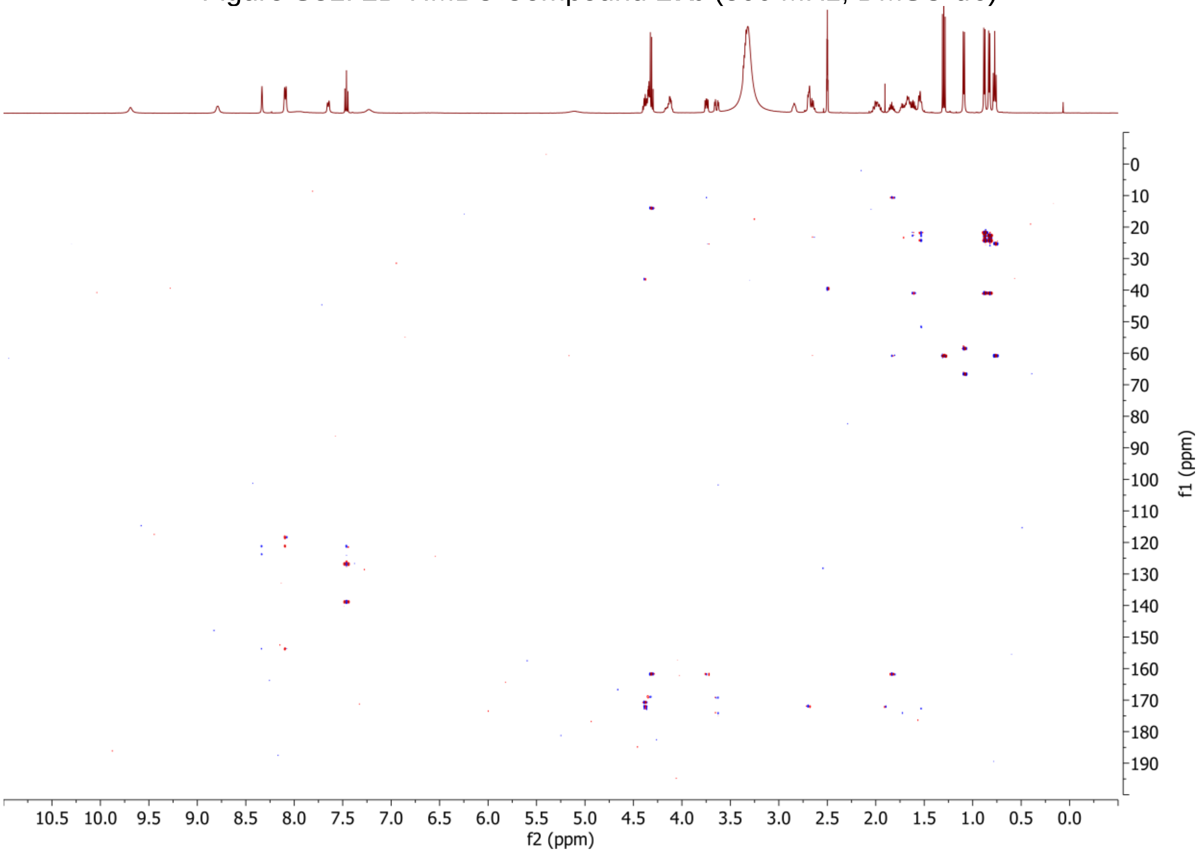

Figure S82: VT 1H-NMR Compound **17b** (500 MHz, DMSO-*d*6)

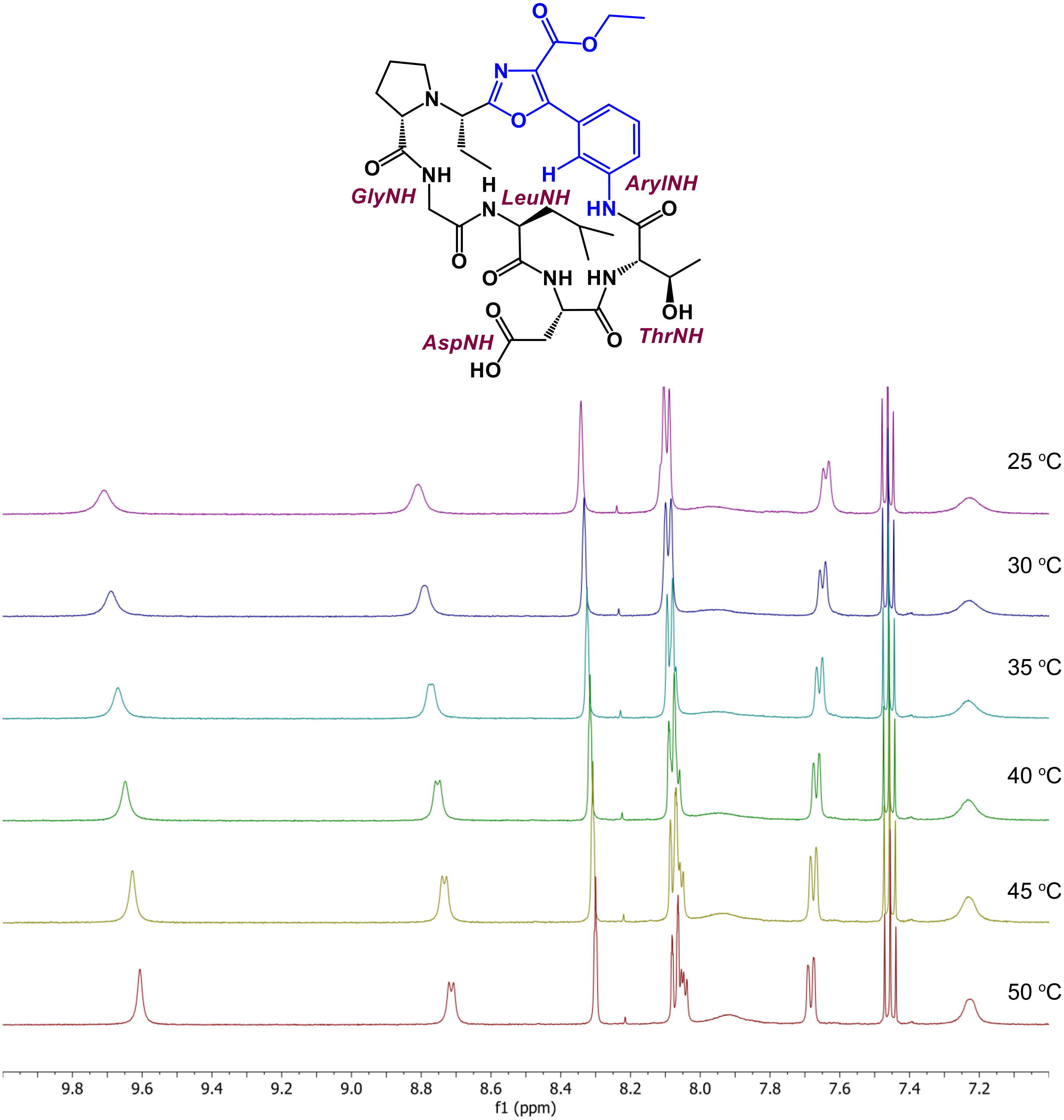

	Shifts (ppm)				
K	*ArylNH*	*Thr*	*Asp*	*Leu*	*Gly*
**298**	9.71	7.23	8.81	7.97	8.11
**323**	9.62	7.22	8.71	7.91	8.06
Δppb/k					
**298-323**	**3.6**	**0.4**	**4**	**2.4**	**2**

Compound S8: Isolated Enamine Byproduct

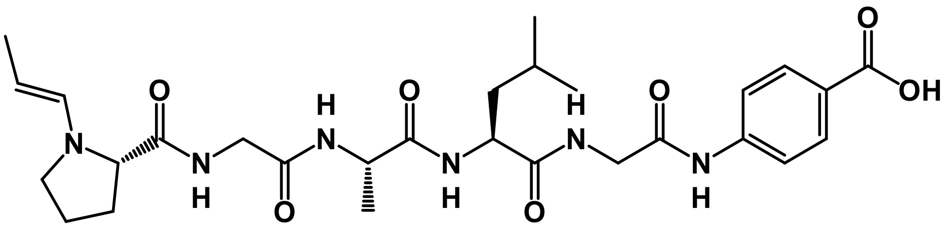

^{1}H NMR (500 MHz, DMSO-*d6*) δ 10.07 (d, *J* = 6.7 Hz, 1H), 8.36 – 8.24 (m, 2H), 8.20 (d, *J* = 7.2 Hz, 2H), 8.15 (dd, *J* = 7.7, 2.0 Hz, 2H), 7.88 (d, *J* = 8.5 Hz, 2H), 7.71 (d, *J* = 8.4 Hz, 2H), 4.48 (dd, *J* = 10.8, 3.8 Hz, 1H), 4.29 (pd, *J* = 7.2, 2.7 Hz, 2H), 3.96 (d, *J* = 16.7 Hz, 1H), 3.88 (d, 2H), 3.77 – 3.67 (m, 1H), 3.63 – 3.52 (m, 2H), 3.01 – 2.92 (m, 1H), 2.77 – 2.64 (m, 1H), 2.02 – 1.87 (m, 1H), 1.82 – 1.68 (m, 1H), 1.65 – 1.57 (m, 2H), 1.55 – 1.47 (m, 3H), 1.23 – 1.18 (m, 3H), 0.94 (t, *J* = 7.3 Hz, 2H), 0.91 – 0.87 (m, 3H), 0.86 – 0.81 (m, 3H). LCMS (ESI+) m/z calc. for $C_{28}H_{41}N_6O_7$ [M+H] = 573.3, found 573.3.

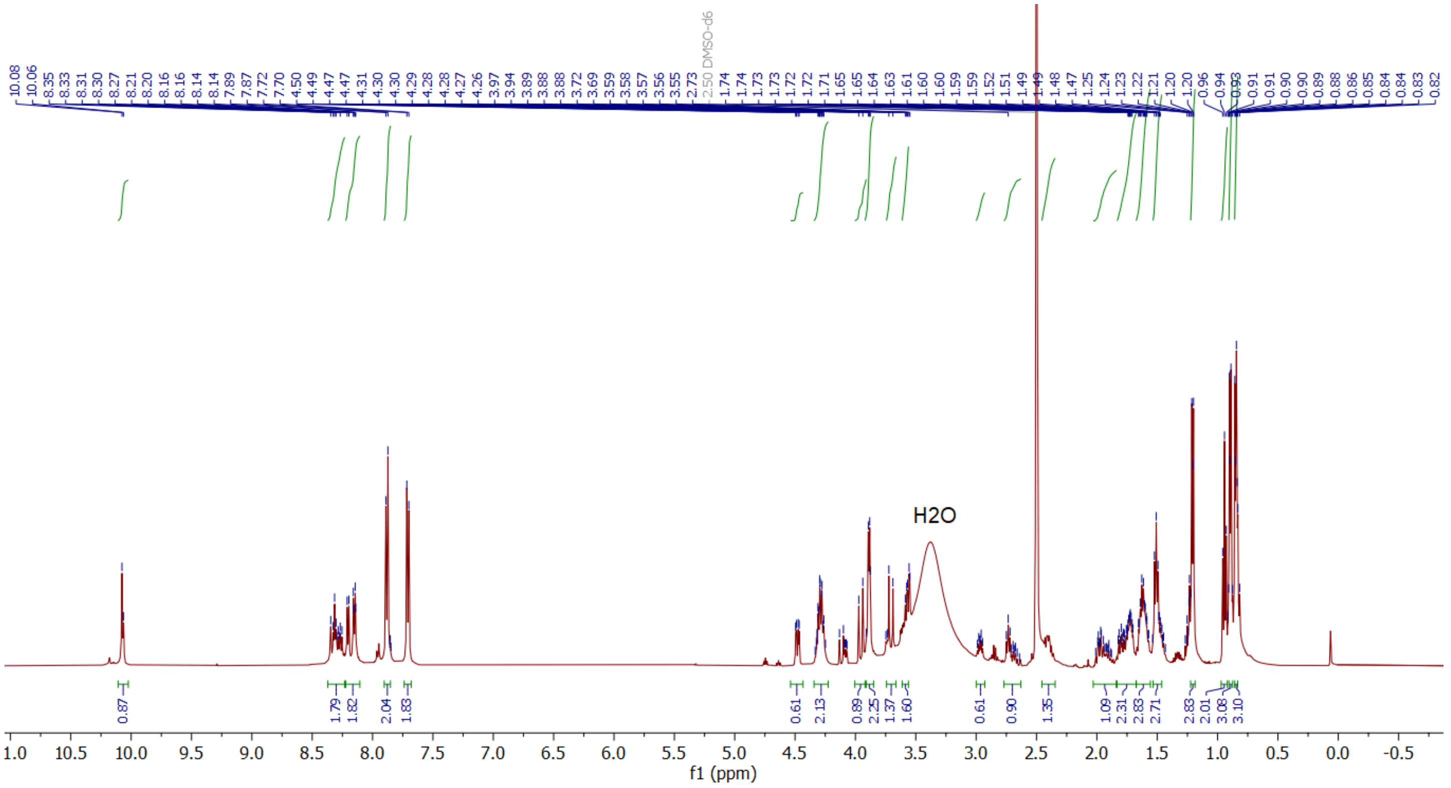

LC Traces

Compound 7a: Retention time = 6.60 min (5-95 %, MeCN/H_2O with 0.1 % formic acid over 15 min)

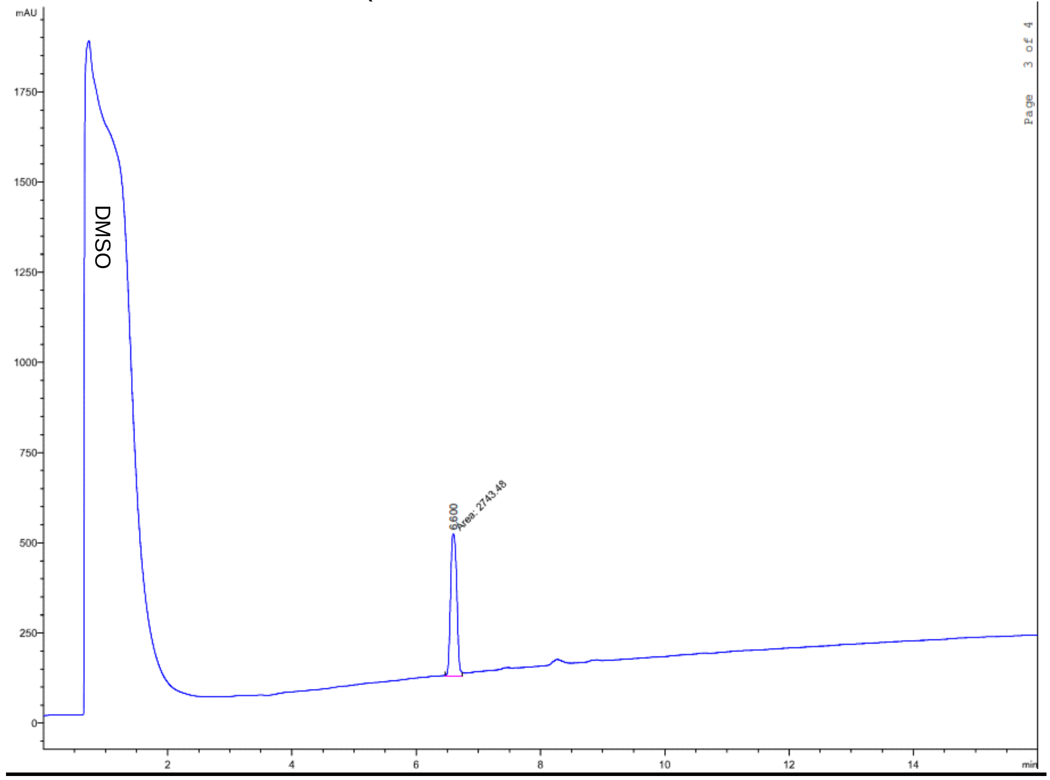

Compound 8a: Retention time = 6.84 min (5-95 %, MeCN/H_2O with 0.1 % formic acid over 15 min)

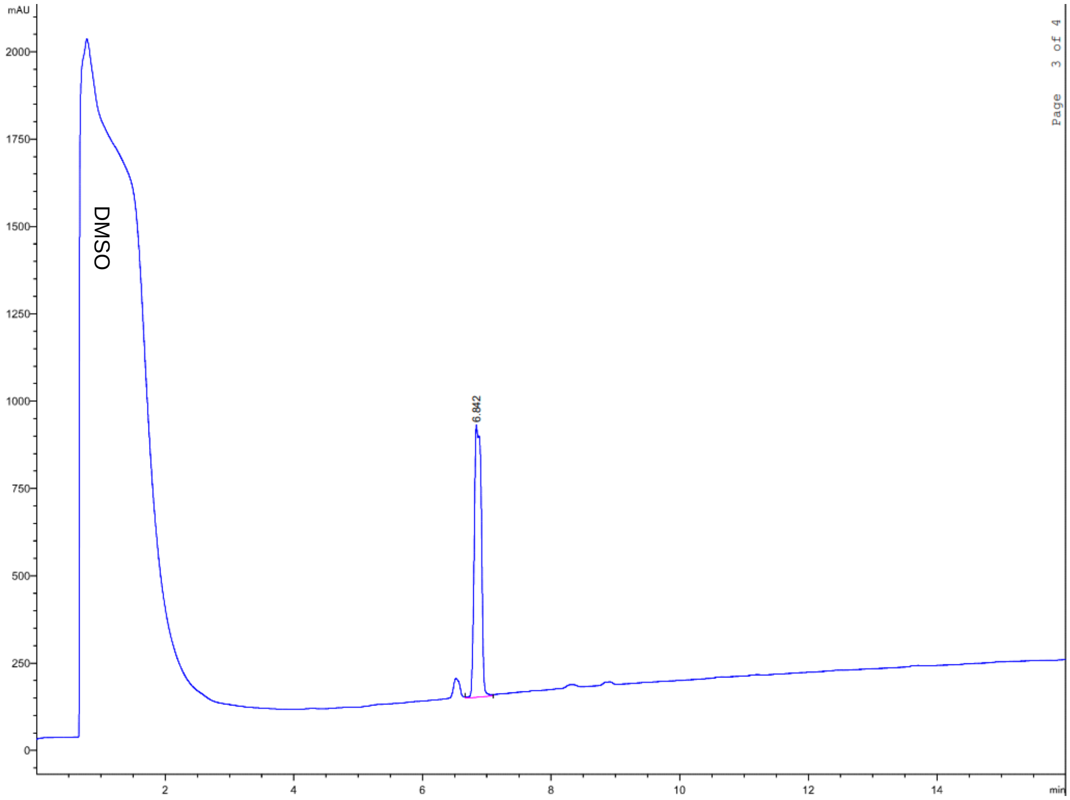

Compound 11a: Retention time = 5.75 min (5-95 %, MeCN/H_2O with 0.1 % formic acid over 15 min)

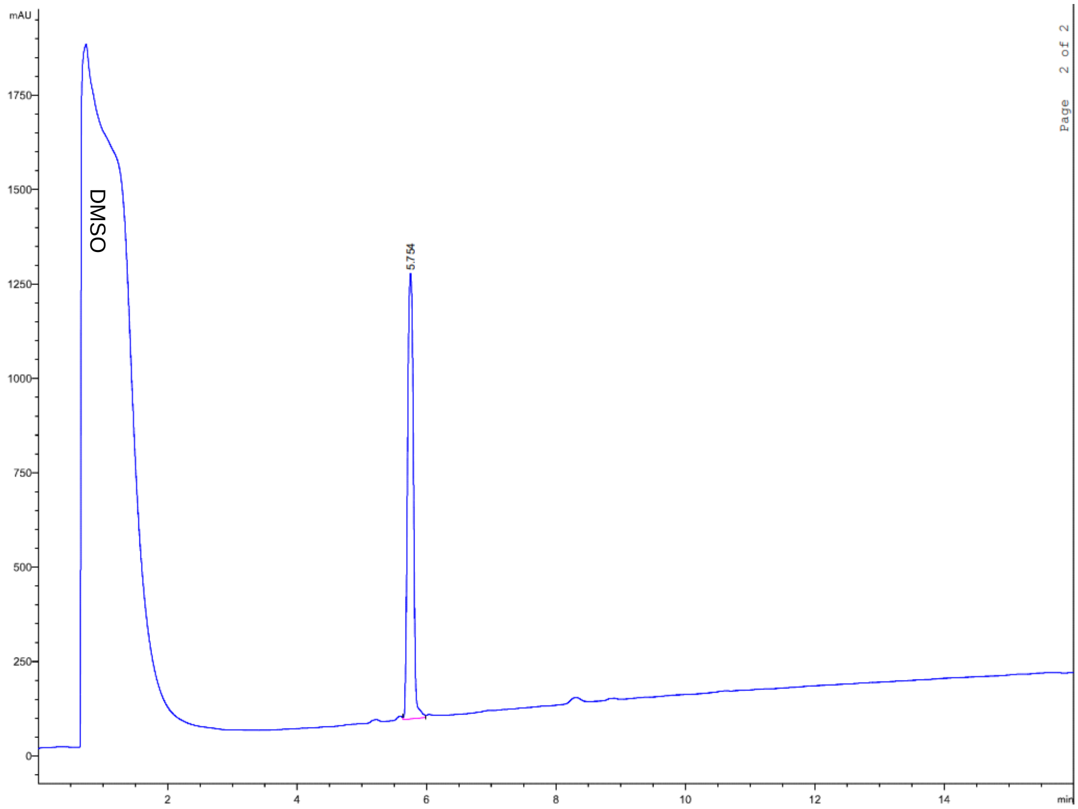

Compound 11b: Retention time = 6.35 min (5-95 %, MeCN/H_2O with 0.1 % formic acid over 15 min)

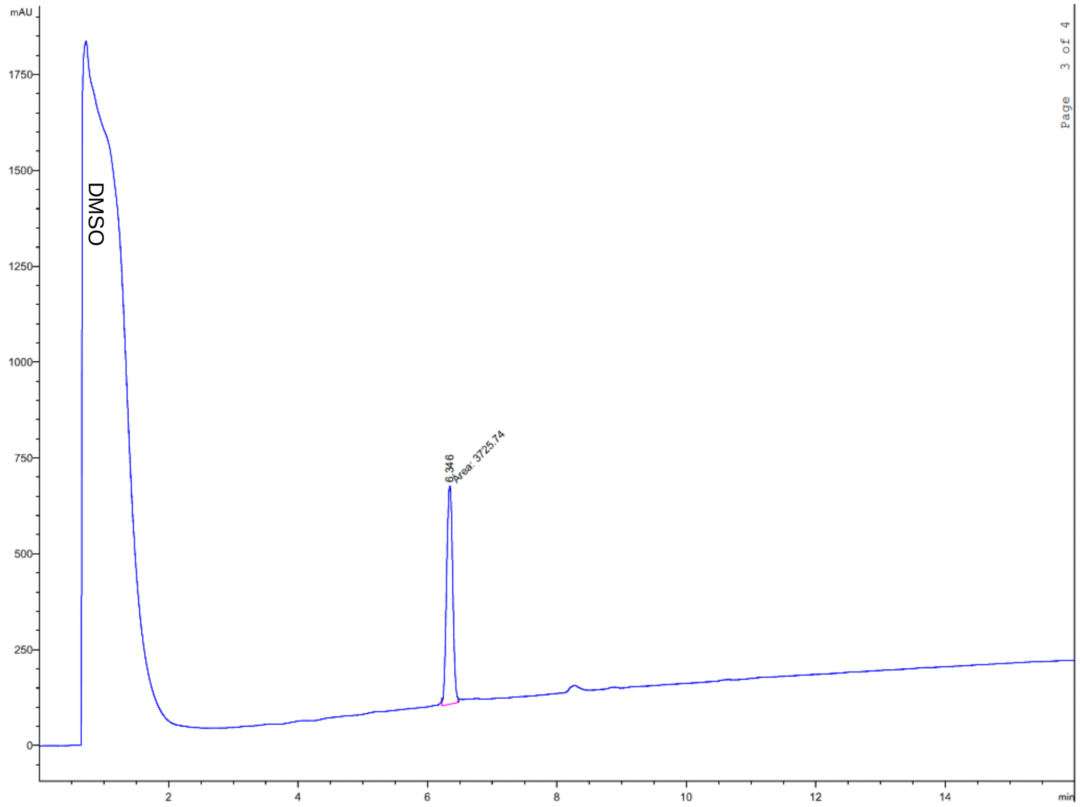

Compound 12a: Retention time = 5.30 min (5-95 %, MeCN/H_2O with 0.1 % formic acid over 15 min)

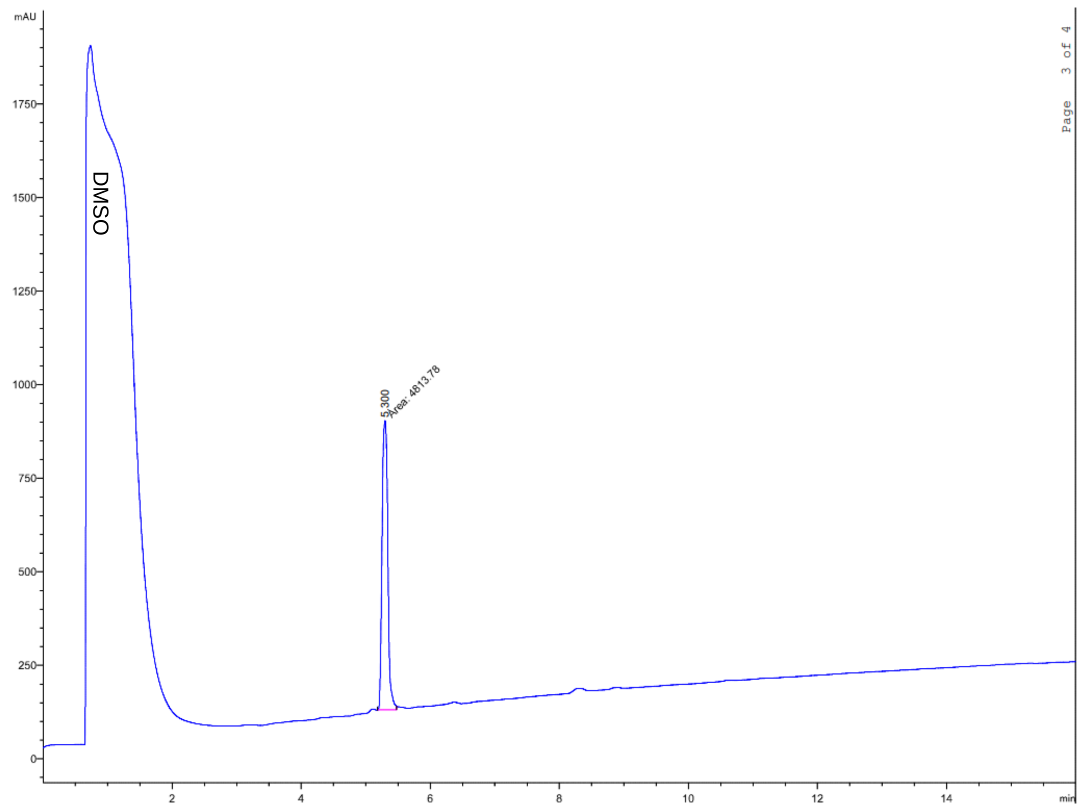

Compound 12b: Retention time = 5.84 min (5-95 %, MeCN/H_2O with 0.1 % formic acid over 15 min)

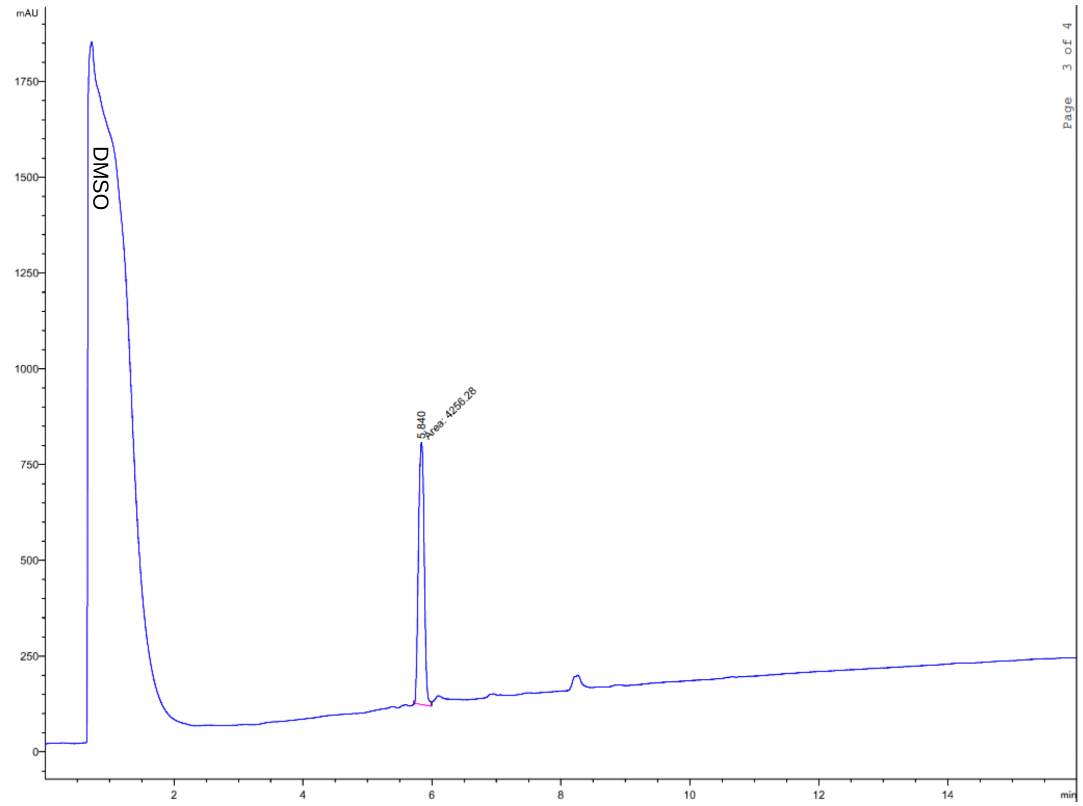

Compound 14a: Retention time = 4.73 min (5-95 %, MeCN/H_2O with 0.1 % formic acid over 15 min)

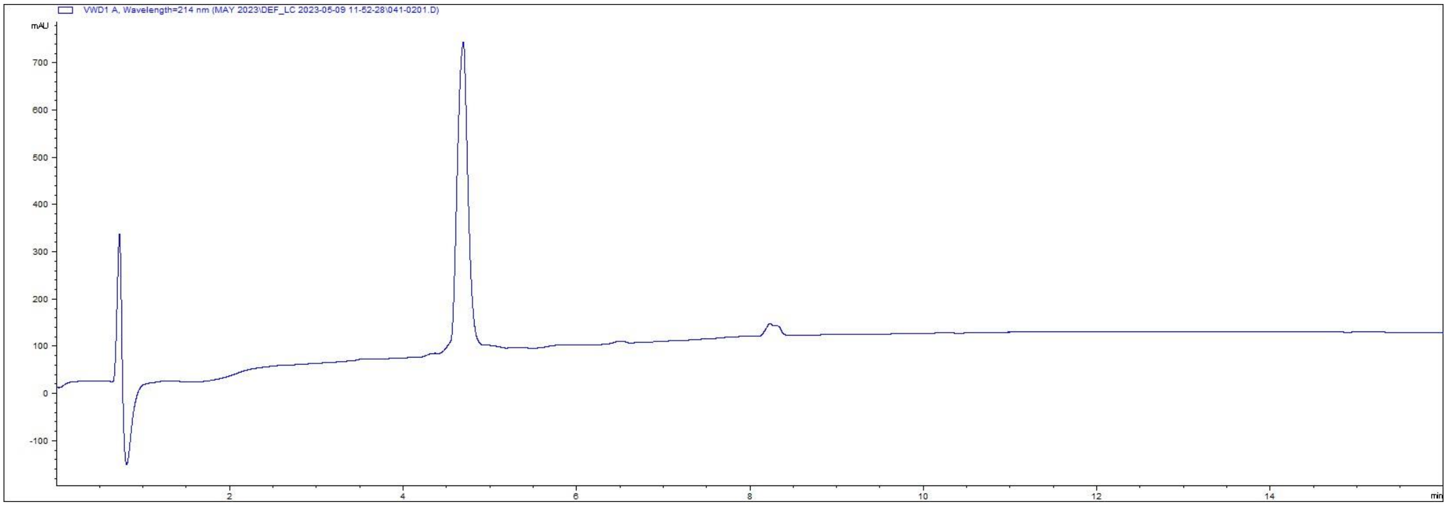

Compound 16a: Retention time = 5.25 min (5-95 %, MeCN/H_2O with 0.1 % formic acid over 15 min)

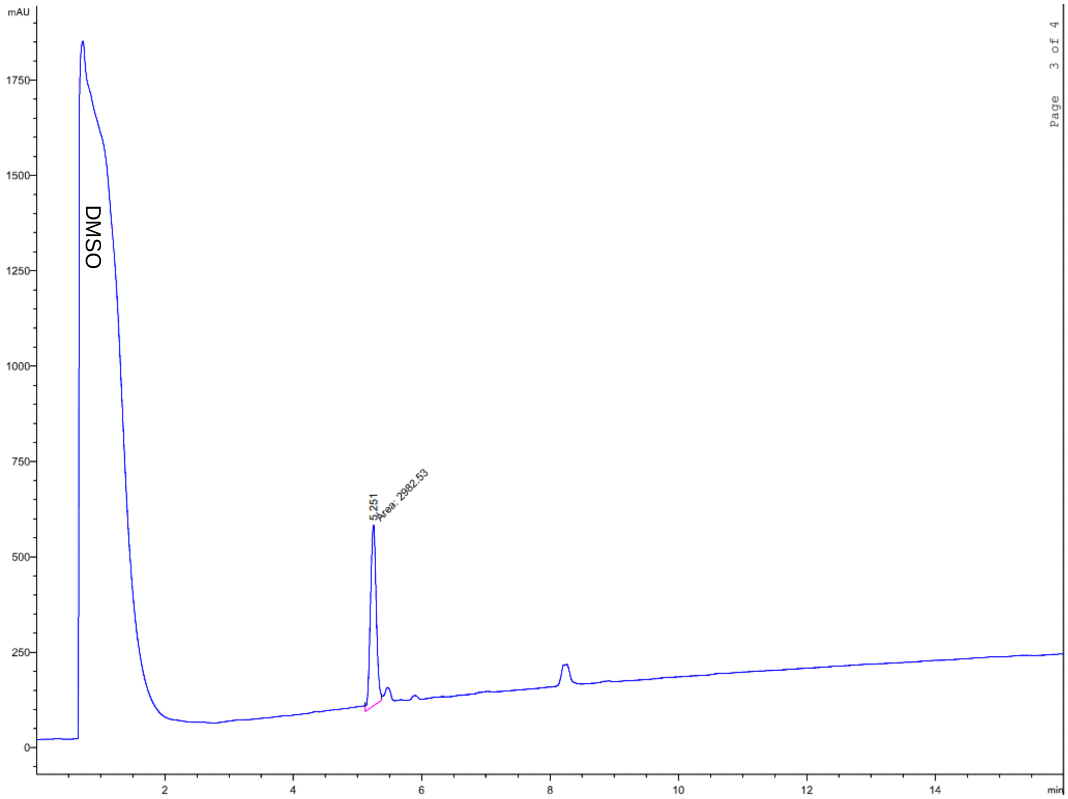

Compound 17a: Retention time = 5.71 min (5-95 %, MeCN/H_2O with 0.1 % formic acid over 15 min)

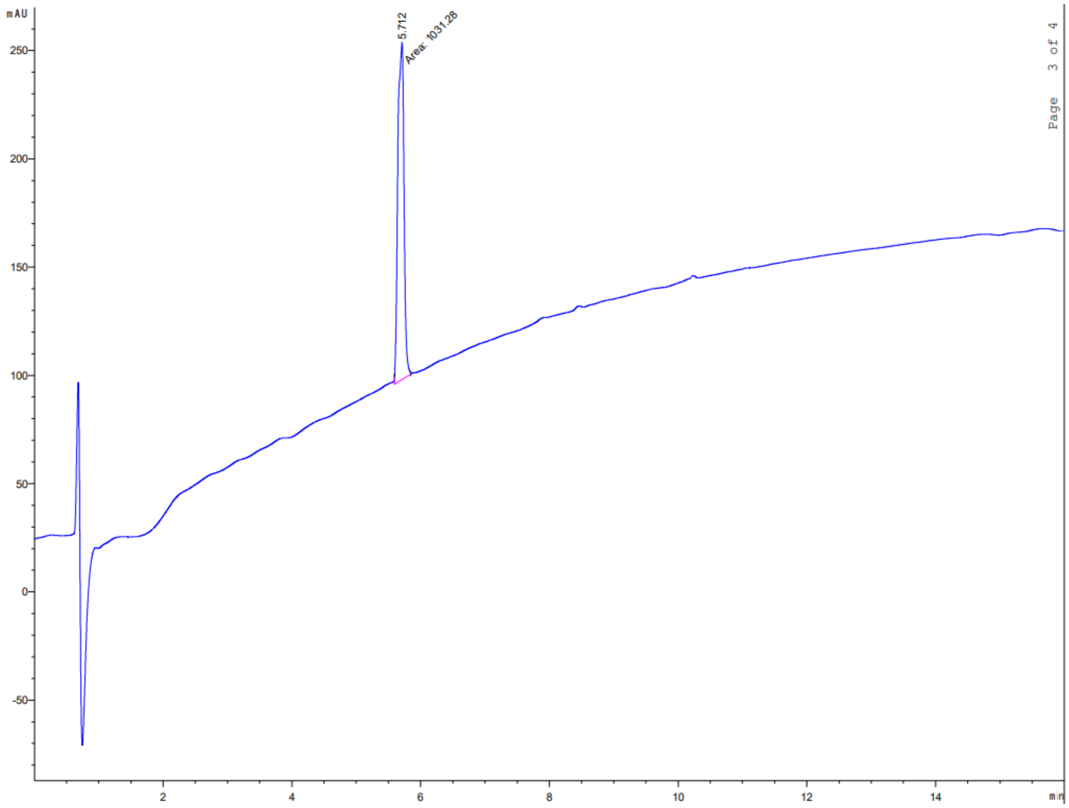

Compound 17b: Retention time = 6.34 min (5-95 %, MeCN/H_2O with 0.1 % formic acid over 15 min)

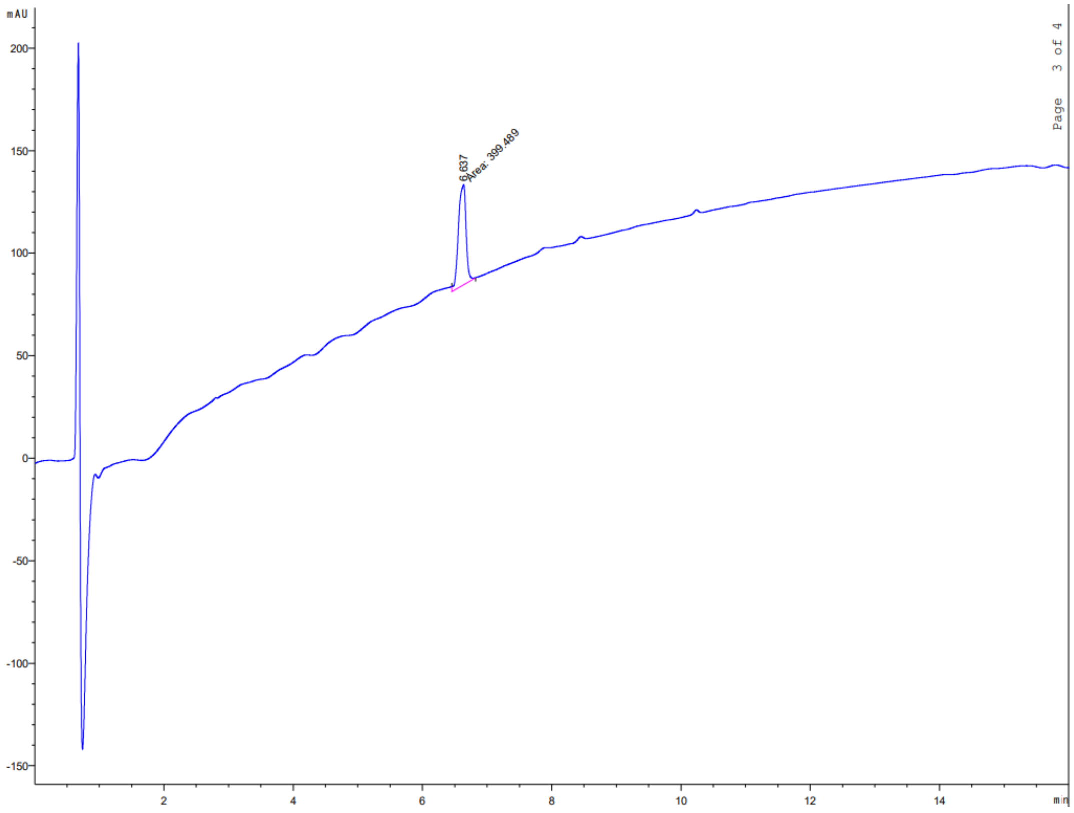

Compound 6b: Crude reaction of peptide **4** with isocyanide **2** (5-95 %, MeCN/H_2O over 6 min)

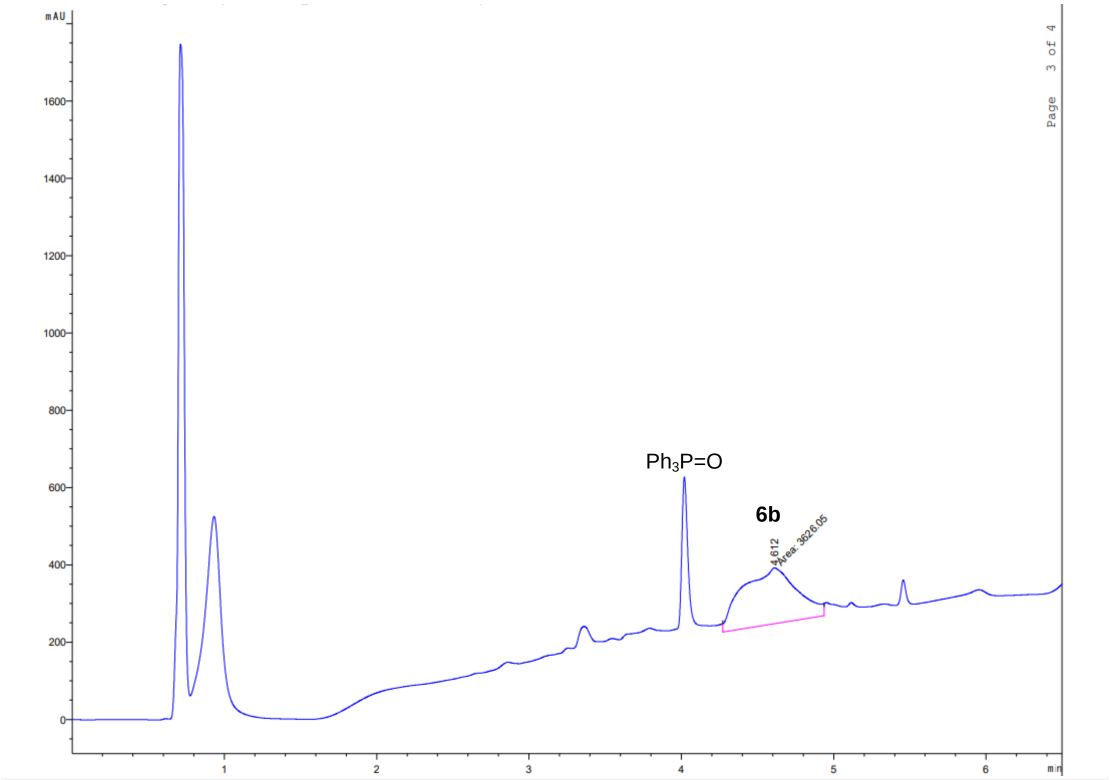

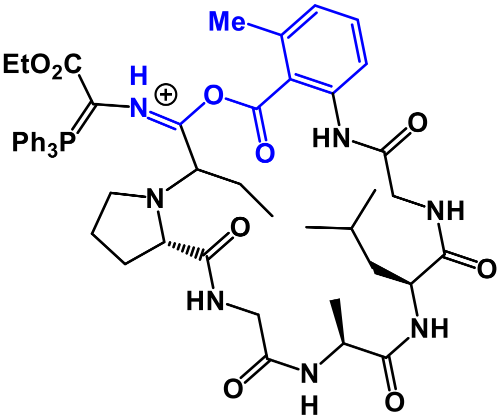

6b

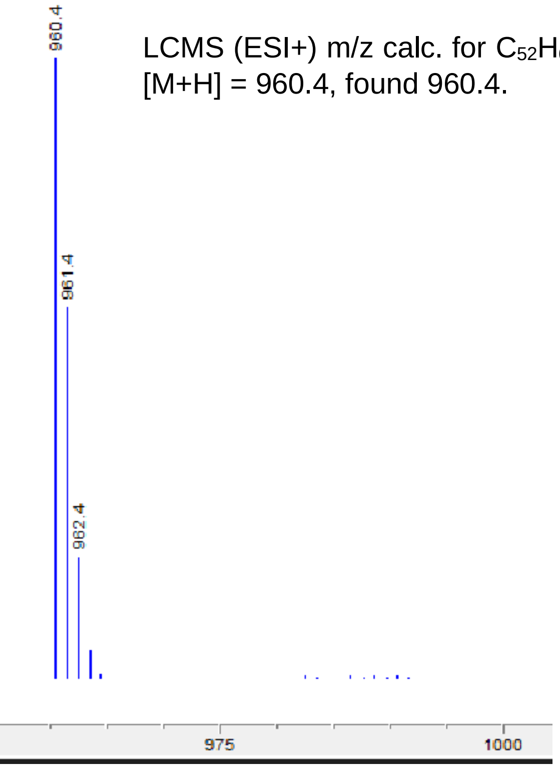

LCMS (ESI+) m/z calc. for $C_{52}H_{63}N_7O_9P^+$ [M+H] = 960.4, found 960.4.

Conformational Analysis

ROE-based restraint: The NMR structures were determined by NMR derived distance information. ROESY spectra were integrated by using MestreNova (v. 10.0.2, Mestrelab Research S.L.) software. Integrated volumes of ROE crosspeaks were converted to proton interatomic distances using an inverse sixth power relationship. A reference integral was calculated as the average integral between sets of geminal protons which was then set to the calculated geminal interproton distance of 1.78 Å. The calculated distances were adjusted upwards and downwards by 10% to give upper and lower bounds to account for uncertainty in interproton distances. 3 J coupling constants were recorded from the 1H spectrum. NH-CαH 3 J coupling constants of < 6 Hz were assigned phi dihedral values of -60° +/- 25°. NHCαH 3 J coupling constants of > 8 Hz were assigned phi values of -120° +/- 25°. Crude structures of macrocycles were generated by a restrained Monte Carlo low mode molecular mechanics conformational search with an implicit solvent model (DMSO) in Macromodel (Schrodinger LLC, v11.0). The structures were then checked for violations of the experimental distances and dihedral restraints. The lowest energy structure that satisfied these tests were passed for molecular dynamics study. Molecular dynamics: Solvent explicit molecular dynamics simulations were carried out with the Desmond Molecular Dynamics software module (D.E. Shaw, v4.4) running inside Maestro (Schodinger LLC, v2015-2). The OPLS3e force field was used for parameterization of the peptidomimetic macrocycle. The macrocycle representative structure was placed in an orthorhombic box solvent box (DMSO) with a minimum distance of 12 A between solute atoms and the box boundary. The solvated box was minimized then brought to 300 K from 10 K using a restrained dynamics regime. Coulombic interactions were grouped into near- and far interactions with a near interaction cutoff of 9 A. Bonds were constrained with the SHAKE algorithm and an integration time step of 2 fs was used. The final MD production run was 100 ns in length with energy value recording every 1.2 ps and trajectory recording every 4.8 ps. The trajectory run was clustered using the Trajectory Clustering script within Maestro with a 0.4 A RMSD cutoff for variation between backbone heavy atoms and a sampling frequency of 10%. The most populated cluster (with a hydrogen bond pattern agreeing with that determined by VT-NMR) was taken as the “preferred” structure. The stereochemistry of the ethyl group was determined to be (S) for each peptide according to the method previously described by Saunders and Yudin.[2] The average interproton distances were measured, compared to the experimental NMR derived distances and the violations were tabulated below:

Compound 7a

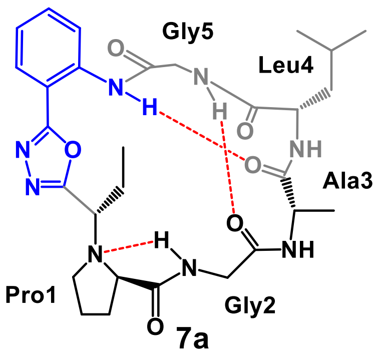

7a

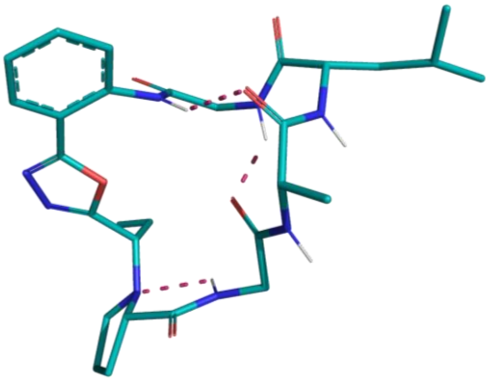

Residue 1	Atom 1	Residue 2	Atom 2	Calculated NOE (Å)	NOE Upper Bound (Å)	NOE Lower Bound (Å)	MD Average Distance (Å)	Violation (Å)
Aryl	*NH*	*Gly5*	*NH*	2.78	3.06	2.50	2.66	0.00
Aryl	*NH*	*Leu*	*NH*	3.20	3.52	2.88	3.97	0.09
Leu	*NH*	*Leu*	*αCH*	2.72	2.99	2.45	3.12	0.13
Leu	*NH*	*Ala*	*αCH*	2.85	3.14	2.56	2.94	0.00
Ala	*NH*	*Leu*	*NH*	2.72	2.99	2.45	3.03	0.04
Ala	*NH*	*Ala*	*αCH*	2.94	3.23	2.65	3.25	0.02
Gly2	*NH*	*Et*	*αCH*	3.42	3.76	3.08	3.25	0.00
Gly2	*NH*	*Pro*	*αCH*	2.42	2.66	2.18	2.41	0.00
Pro	*αCH*	*Et*	*αCH*	2.51	2.76	2.26	2.86	0.10

Compound 8a

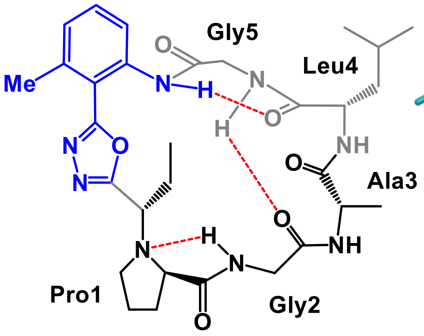

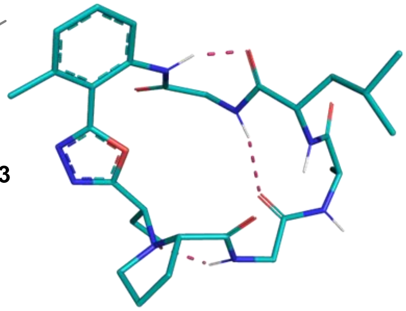

8b

Residue 1	Atom 1	Residue 2	Atom 2	Calculated NOE (Å)	NOE Upper Bound (Å)	NOE Lower Bound (Å)	MD Average Distance (Å)	Violation (Å)
Aryl	*NH*	*Gly2*	*NH*	2.67	2.94	2.40	2.77	0.00
Aryl	*NH*	*Aryl*	*C_6H*	2.65	2.91	2.38	2.64	0.00
Ala	*NH*	*Ala*	*αCH*	2.60	2.86	2.34	2.20	0.14
Leu	*NH*	*Ala*	*αCH*	2.83	3.11	2.55	3.45	0.34
Leu	*αCH*	*Leu*	*NH*	2.39	2.63	2.15	2.94	0.31

Compound 11a

11a

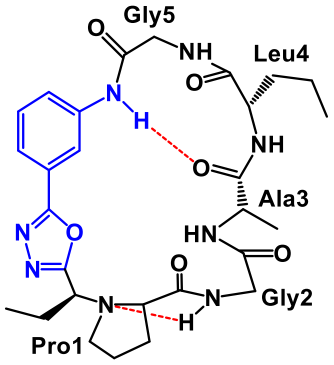

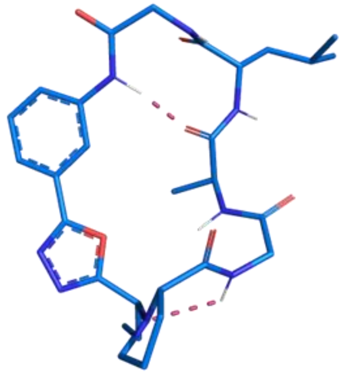

Residue 1	Atom 1	Residue 2	Atom 2	Calculated NOE (Å)	NOE Upper Bound (Å)	NOE Lower Bound (Å)	MD Average Distance (Å)	Violation (Å)
Aryl	*NH*	*Gly5*	*NH*	2.78	3.06	2.50	2.81	0.00
Gly5	*NH*	*Leu*	*αCH*	2.22	2.44	2.00	2.93	0.49
Leu	*NH*	*Ala*	*αCH*	2.40	2.64	2.16	2.21	0.00
Ala	*NH*	*Ala*	*αCH*	3.20	3.52	2.88	2.23	0.55
Ala	*NH*	*Et*	*αCH*	3.20	3.52	2.88	2.69	0.19
Gly2	*NH*	*Et*	*αCH*	2.94	3.23	2.65	2.70	0.00
Gly2	*NH*	*Pro*	*αCH*	2.54	2.79	2.29	2.96	0.17
Gly2	*NH*	*Ala*	*NH*	3.20	3.52	2.88	2.91	0.00
Pro	*αCH*	*Et*	*αCH*	2.72	2.99	2.45	2.62	0.00

Compound 11b

11b

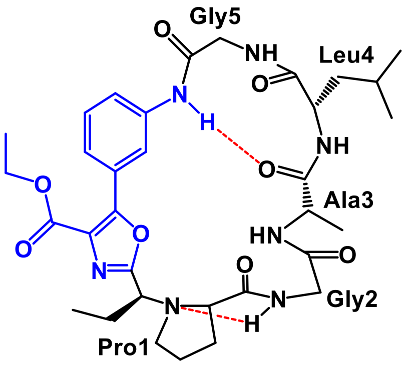

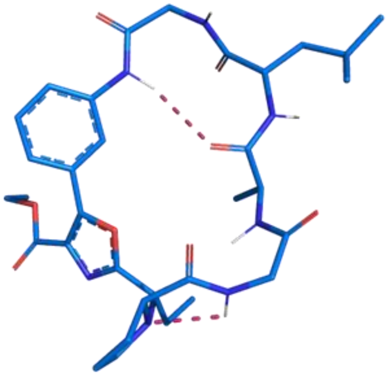

Residue 1	Atom 1	Residue 2	Atom 2	Calculated NOE (Å)	NOE Upper Bound (Å)	NOE Lower Bound (Å)	MD Average Distance (Å)	Violation (Å)
Aryl	*NH*	*Gly2*	*NH*	2.93	3.22	2.66	2.75	0.00
Gly5	*NH*	*Leu*	*αCH*	2.36	2.60	2.12	3.05	0.45
Leu	*NH*	*Ala*	*αCH*	2.41	2.65	2.17	2.33	0.00
Ala	*NH*	*Ala*	*αCH*	2.93	3.22	2.66	2.86	0.00
Leu	*NH*	*Leu*	*αCH*	2.71	2.98	2.44	2.28	0.16
Gly2	*NH*	*Pro*	*αCH*	2.41	2.65	2.17	2.91	0.26
Gly2	*NH*	*Et*	*αCH*	2.84	3.12	2.56	3.57	0.45
Pro	*αCH*	*Et*	*αCH*	2.53	2.78	2.28	2.70	0.00

Compound 12a

12a

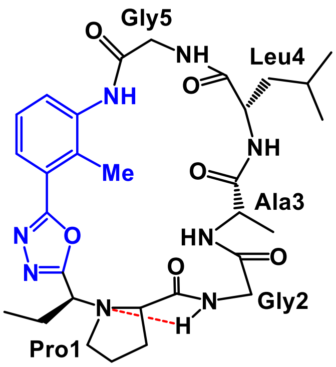

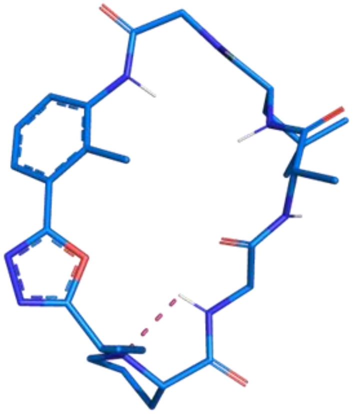

Residue 1	Atom 1	Residue 2	Atom 2	Calculated NOE (Å)	NOE Upper Bound (Å)	NOE Lower Bound (Å)	MD Average Distance (Å)	Violation (Å)
Aryl	*NH*	*Gly5*	*NH*	2.93	3.22	2.66	3.38	0.16
Gly5	*NH*	*Leu*	*NH*	3.41	3.75	3.07	4.03	0.28
Gly5	*NH*	*Leu*	*αCH*	2.24	2.46	2.02	2.46	0.00
Leu	*NH*	*Leu*	*αCH*	2.77	3.05	2.49	2.60	0.00
Leu	*NH*	*Ala*	*NH*	2.84	3.12	2.56	2.52	0.04
Ala	*NH*	*Ala*	*αCH*	2.98	3.28	2.68	2.93	0.00
Gly2	*NH*	*Pro*	*αCH*	2.77	3.05	2.49	3.30	0.25
Pro	*αCH*	*Et*	*αCH*	2.53	2.78	2.28	2.84	0.06

Compound 12b

12b

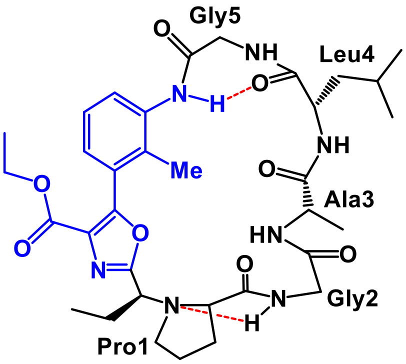

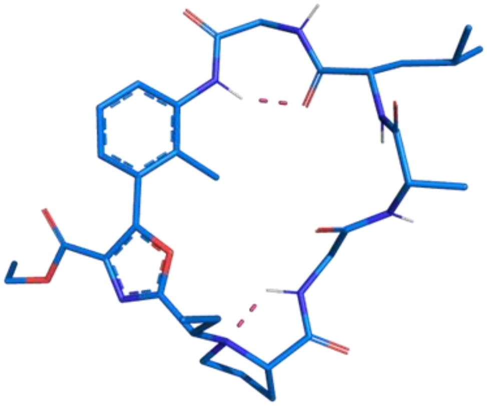

Residue 1	Atom 1	Residue 2	Atom 2	Calculated NOE (Å)	NOE Upper Bound (Å)	NOE Lower Bound (Å)	MD Average Distance (Å)	Violation (Å)
Aryl	*NH*	*Leu*	*NH*	3.04	3.34	2.74	2.84	0.00
Gly5	*NH*	*Leu*	*NH*	3.42	3.76	3.08	4.03	0.27
Gly5	*NH*	*Leu*	*αCH*	2.23	2.46	2.02	3.37	0.91
Ala	*NH*	*Ala*	*αCH*	2.93	3.28	2.68	3.33	0.05
Pro	*αCH*	*Gly2*	*NH*	2.77	3.05	2.49	2.35	0.14
Et	*αCH*	*Gly2*	*NH*	3.04	3.34	2.74	4.21	0.87
Et	*αCH*	*Pro*	*αCH*	2.50	2.75	2.25	2.46	0.00

Compound 14a

14a

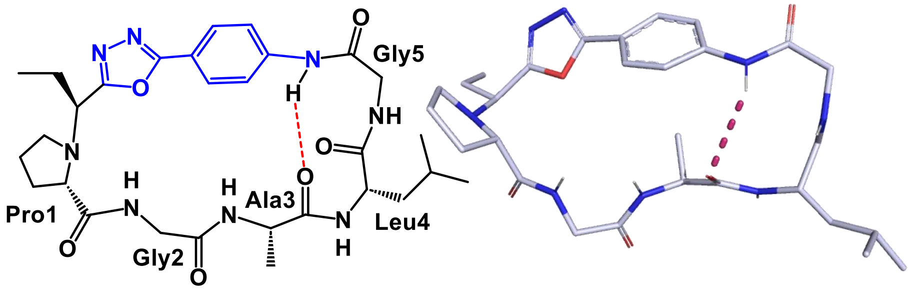

Residue 1	Atom 1	Residue 2	Atom 2	Calculated NOE (Å)	NOE Upper Bound (Å)	NOE Lower Bound (Å)	MD Average Distance (Å)	Violation (Å)
Aryl	*NH*	*Gly2*	*NH*	2.39	2.63	2.15	2.72	0.09
Ala	*αCH*	*Ala*	*NH*	2.47	2.72	2.22	2.82	0.10
Leu	*αCH*	*Gly2*	*NH*	2.15	2.36	1.93	2.31	0.00
Leu	*αCH*	*Leu*	*NH*	2.57	2.83	2.31	2.89	0.06
Pro	*αCH*	*Gly1*	*NH*	2.17	2.39	1.95	2.42	0.03

Compound 16a

16a

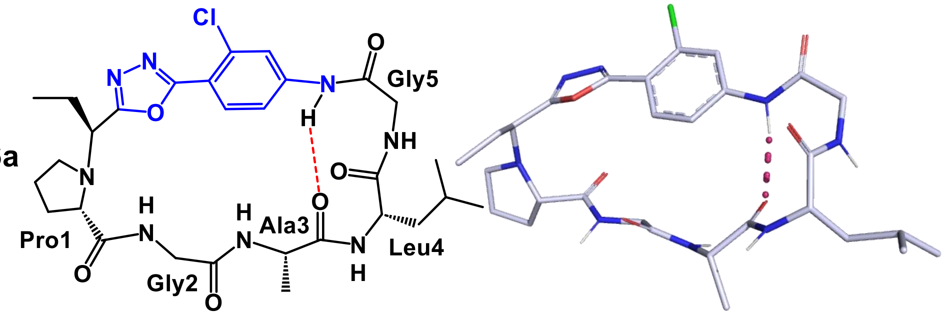

Residue 1	Atom 1	Residue 2	Atom 2	Calculated NOE (Å)	NOE Upper Bound (Å)	NOE Lower Bound (Å)	MD Average Distance (Å)	Violation (Å)
Aryl	*NH*	*Gly5*	*NH*	2.74	3.02	2.46	2.70	0.00
Gly5	*NH*	*Leu*	*αCH*	2.28	2.51	2.05	2.30	0.00
Leu	*NH*	*Leu*	*αCH*	2.71	2.98	2.44	2.79	0.00
Leu	*NH*	*Ala*	*αCH*	2.85	3.14	2.56	3.28	0.14
Gly	*NH*	*Pro*	*αCH*	2.33	2.56	2.10	2.91	0.45

Compound 17a

17a

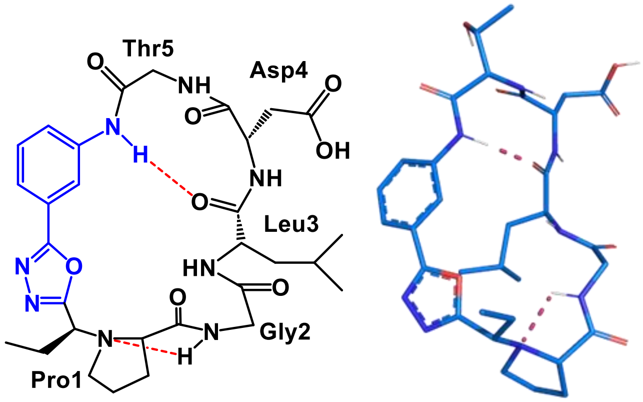

Residue 1	Atom 1	Residue 2	Atom 2	Calculated NOE (Å)	NOE Upper Bound (Å)	NOE Lower Bound (Å)	MD Average Distance (Å)	Violation (Å)
Aryl	*NH*	*Thr*	*NH*	3.19	3.52	2.87	3.62	0.10
Aryl	*NH*	*Thr*	*αCH*	2.53	2.78	2.28	2.28	0.00
Asp	*NH*	*Asp*	*αCH*	3.04	3.44	2.74	3.34	0.00
Asp	*NH*	*Leu*	*αCH*	2.66	2.93	2.39	2.81	0.00
Gly2	*NH*	*Et*	*αCH*	3.19	3.51	2.97	3.93	0.42
Gly2	*NH*	*Pro*	*αCH*	2.71	2.98	2.44	2.22	0.22
Pro	*αCH*	*Et*	*αCH*	2.71	2.98	2.44	3.29	0.31

Compound 17b

17b

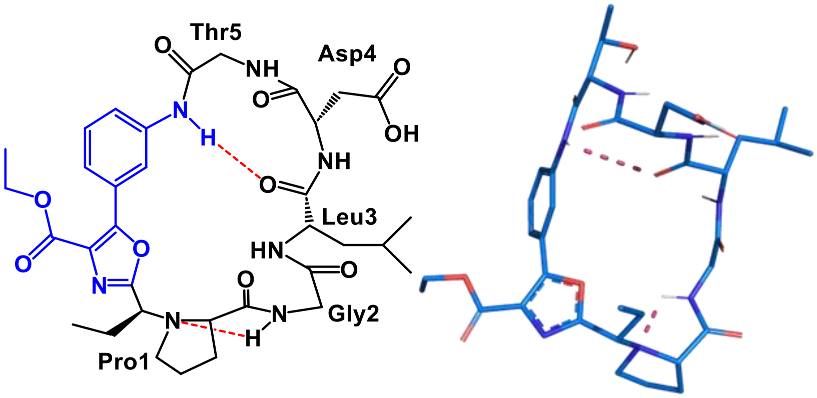

Residue 1	Atom 1	Residue 2	Atom 2	Calculated NOE (Å)	NOE Upper Bound (Å)	NOE Lower Bound (Å)	MD Average Distance (Å)	Violation (Å)
Aryl	*NH*	*Thr*	*αCH*	2.84	3.12	2.56	3.34	0.22
Asp	*NH*	*Leu*	*αCH*	2.50	2.75	2.25	2.22	0.03
Gly2	*NH*	*Et*	*αCH*	3.42	3.76	3.08	3.93	0.17
Gly2	*NH*	*Pro*	*αCH*	2.77	3.05	2.49	3.30	0.25
Pro	*αCH*	*Et*	*αCH*	2.57	2.83	2.31	2.81	0.00

Macrocycle Conformation Maps

ϕ / ψ torsion angles were extracted from the conformations of 17a and 17b using PyMOL and are tabulated below.

17a

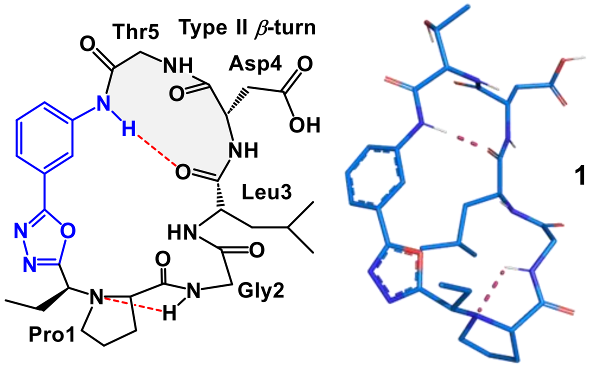

17b

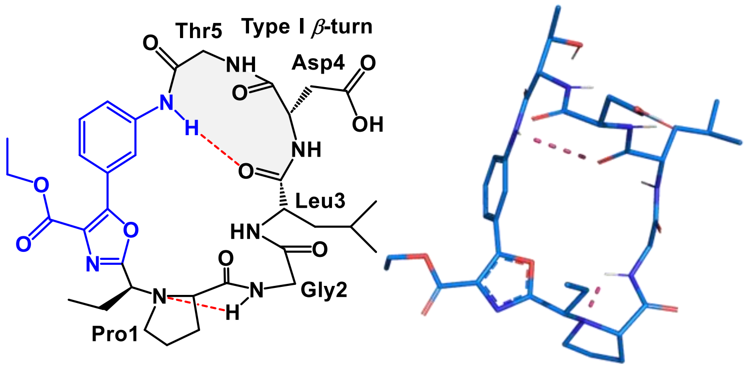

MCM Plot of LDT 17a and 17b					
	17a			***17b***	
	ϕ	ψ		ϕ	ψ
**Pro**	-101	11	**Pro**	-105	15
**Gly**	119	68	**Gly**	-115	-123
**Leu**	-109	142	**Leu**	-80	148
**Asp**	-48	91	**Asp**	-74	-20
**Thr**	57	37	**Thr**	-65	-2

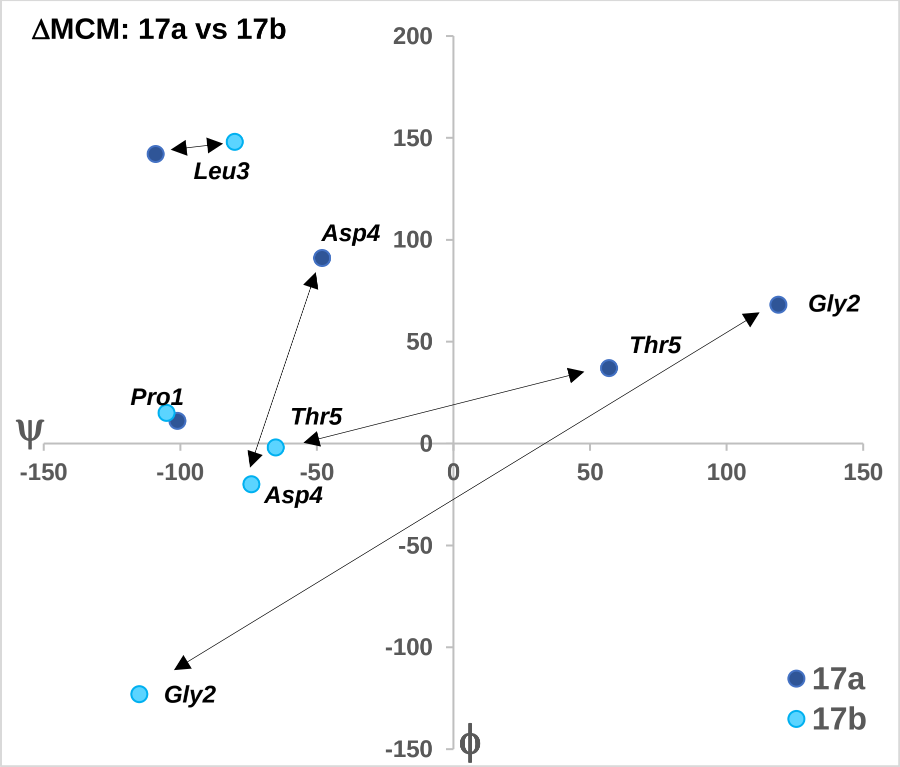

References:

1. Diamandas, M.; Moreira, R.; Taylor, S. D. Solid-Phase Total Synthesis of Dehydrotryptophan-Bearing Cyclic Peptides Tunicyclin B, Sclerotide A, CDA3a, and CDA4a using a Protected β-Hydroxytryptophan Building Block. *Org. Lett*. **2021**, *12* (8), https://doi.org/10.1021/acs.orglett.1c00717.
2. Saunders, G. J.; Yudin, A. K. Property-Driven Development of Passively Permeable Macrocyclic Scaffolds Using Heterocycles**. *Angewandte Chemie International Edition* **2022**, *61* (33), e202206866. https://doi.org/https://doi.org/10.1002/anie.202206866.
